# The Biosynthetic Gene Cluster for Sestermobaraenes—Discovery of a Geranylfarnesyl Diphosphate Synthase and a Multiproduct Sesterterpene Synthase from *Streptomyces mobaraensis*

**DOI:** 10.1002/anie.202010084

**Published:** 2020-08-31

**Authors:** Anwei Hou, Jeroen S. Dickschat

**Affiliations:** ^1^ Kekulé-Institute of Organic Chemistry and Biochemistry University of Bonn Gerhard-Domagk-Strasse 1 53121 Bonn Germany

**Keywords:** biosynthetic gene cluster, configuration determination, enzyme catalysis, *Streptomyces mobaraensis*, terpenes

## Abstract

A biosynthetic gene cluster from *Streptomyces mobaraensis* encoding the first cases of a bacterial geranylfarnesyl diphosphate synthase and a type I sesterterpene synthase was identified. The structures of seven sesterterpenes produced by these enzymes were elucidated, including their absolute configurations. The enzyme mechanism of the sesterterpene synthase was investigated by extensive isotope labeling experiments.

## Graphical abstract

11001_2_=5^2^=XXV. Not the answer to everything, but the number of carbons in a sesterterpene, for which we describe the first bacterial type I terpene synthase and geranylfarnesyl diphosphate synthase encoded by a gene cluster from *Streptomyces mobaraensis*. Seven sesterterpenes were obtained with the recombinant enzymes and their biosynthesis was analyzed by isotope labeling experiments.

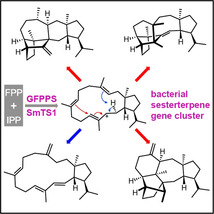

Terpenoids exhibit important physiological functions,^[1]^ are widely used in chemical and fragrance industry, and are an important resource for drug discovery and development attracting the interest of synthetic chemists.[^2^, ^3^] Their often highly complex skeletons are biosynthesized from two simple universal C_5_ precursors, dimethylallyl diphosphate (DMAPP) and isopentenyl diphosphate (IPP), that are assembled by prenyltransferases (PTs) into oligoprenyl diphosphates, followed by terpene synthase (TS) mediated cyclizations through cationic cascade reactions into terpene hydrocarbons or alcohols.[^4^, ^5^] Tailoring enzymes such as oxidases are often required to introduce bioactivity, and the extended knowledge about whole pathways allows for biotechnological approaches for compound production.[^6^, ^7^]

The bacterial farnesyl diphosphate (FPP) synthases (FPPS) from *Escherichia coli* and *Bacillus stearothermophilus* were already discovered three decades ago.[^8^, ^9^] More recently, bacterial geranylgeranyl diphosphate (GGPP) synthases (GGPPS) have been described whose genes are clustered with TS genes for the biosynthesis of secondary metabolites such as the labdanmycins from *Streptomyces* sp. KIB 015 and the venezuelaenes from *Streptomyces venezuelae*.[^10^, ^11^] No bacterial geranylfarnesyl diphosphate (GFPP) synthase (GFPPS) has been reported so far, and also the knowledge about bacterial sesterterpene synthases (StTSs) is scarce. The few known examples include the non‐canonical enzyme Bcl‐TS from *Bacillus clausii* that converts GFPP and hexaprenyl diphosphate into β‐geranylfarnesene (**1**) and β‐hexaprene, respectively,^[12]^ and StsC from *Streptomyces somaliensis*, a membrane protein belonging to the UbiA superfamily, that converts GFPP into somaliensenes A (**2**) and B (**3**) (Figure 1).^[13]^ The only type I TS from bacteria with StTS side activity that catalyzes the conversion of GFPP into prenylspata‐13,17‐diene (**4**) is spata‐13,17‐diene synthase (SpS) from *Streptomyces xinghaiensis*, but this enzyme functions naturally as a diterpene synthase.^[14]^ Starting from the ophiobolin F (**5**) synthase from *Aspergillus clavatus*, a few StTSs from fungi[^15^, ^16^, ^17^, ^18^, ^19^, ^20^, ^21^, ^22^, ^23^] and plants[^24^, ^25^, ^26^] were discovered recently. In fungi sesterterpene biosynthesis is always promoted by bifunctional enzymes with a GFPPS and a StTS domain,[^27^, ^28^] while in plants clustered genes for two discrete enzymes are found. Here we report on a two‐gene cluster from *Streptomyces mobaraensis* NBRC 13819 and characterization of the encoded enzymes as unprecedented bacterial representatives of a GFPPS and a type I StTS (SmTS1). The structures of seven products made by SmTS1 and the experimentally verified cyclization mechanism to these compounds are presented.

**Figure 1:**
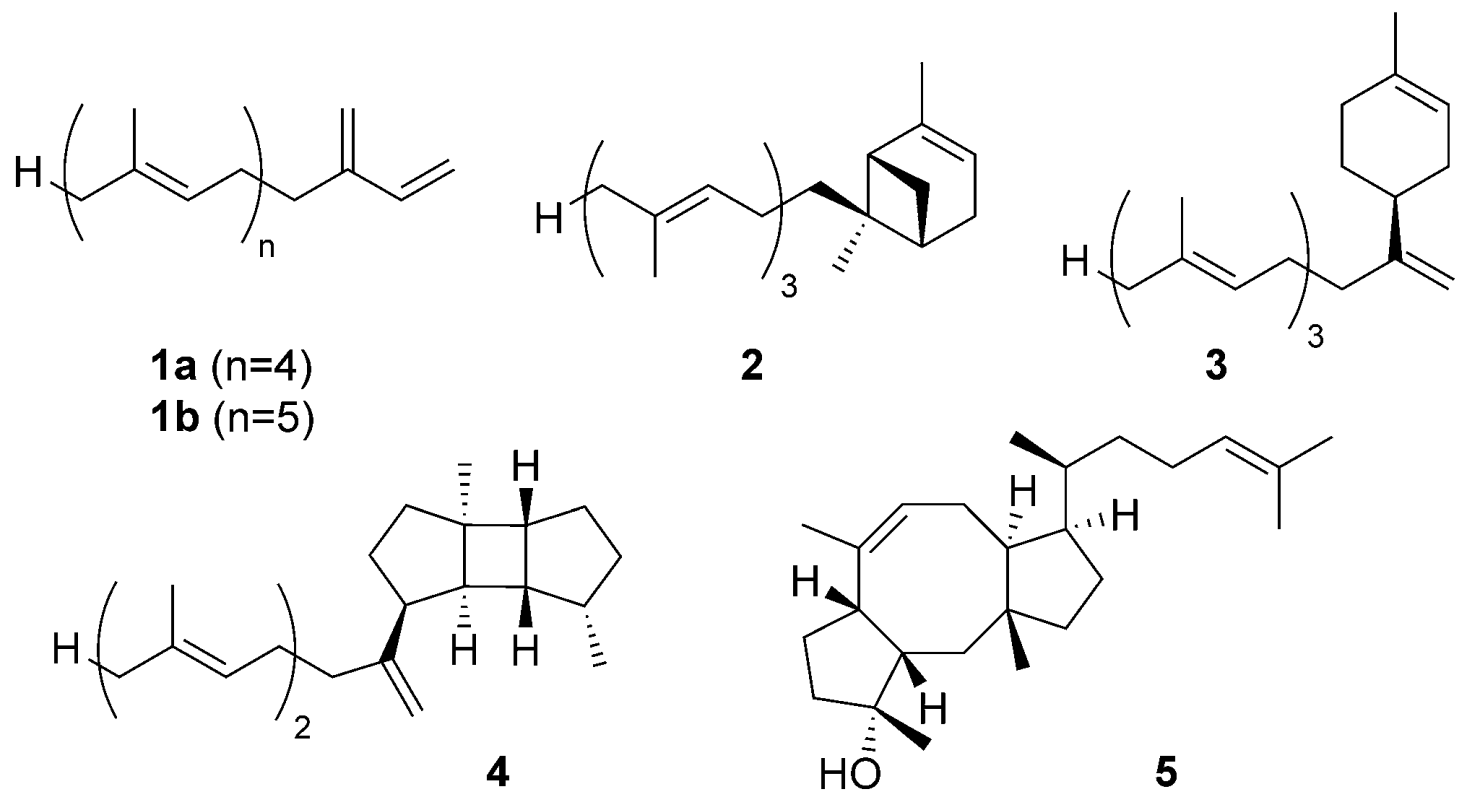
Bacterial sesterterpenes and the fungal compound ophiobolin F (**5**).

Volatiles can efficiently be analyzed by trapping on charcoal filters using a closed‐loop stripping apparatus, followed by extraction of the filters and GC/MS analysis of the obtained extracts.^[29]^ By application of this technique to bacterial cultures volatile mono‐ and sesquiterpenes are frequently observed,^[30]^ while reports about diterpenes are rare,^[31]^ likely because they are less volatile, but also because they occur less often in bacteria. During our continuous work on the analysis of volatiles from bacteria we made the remarkable observation that agar plate cultures of *S. mobaraensis* released several sesterterpenes (Figures S1 and S2). A BLAST search revealed that this bacterium encodes at least 10 type I TSs (SmTS1–SmTS10, Table S1, Figures S3 and S4), two of which showed close homology to the characterized 2‐methylisoborneol synthase and geosmin synthase from *Streptomyces coelicolor*.[^32^, ^33^] The other eight TS genes were cloned into the expression plasmid pYE‐Express by homologous recombination in yeast, gene expression in *E. coli* and protein purification that was successful for SmTS1 (Figure S5) and SmTS6–SmTS10 (SmTS4 and SmTS5 were insoluble proteins). All soluble proteins were tested with GFPP as substrate that was only converted by SmTS1, but not by the other enzymes, into a mixture of sesterterpene hydrocarbons and one sesterterpene alcohol (Figures S1 and S2), while geranyl diphosphate (GPP), FPP and GGPP were not accepted by SmTS1. Notably, the in vitro reaction with GFPP yielded all sesterterpenes observed in the headspace extracts, in addition to a few more compounds that were only obtained from the enzymatic reaction; they represent the less volatile compounds with higher retention times and one trace compound. A genome analysis through antiSMASH^[34]^ revealed that the gene of SmTS1 was clustered with a gene for an enzyme from the PT family and flanked by genes for a polyketide synthase (upstream) and a glycosyltransferase (downstream, Figure 2). The PT gene was suspected to encode a GFPPS; gene cloning and expression in *E. coli*, followed by incubation of the purified enzymes (Figure S5) with DMAPP, GPP, FPP and GGPP together with IPP confirmed this hypothesis by the observation of sesterterpenes in all cases (Figure S6).

**Figure 2:**
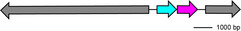
Clustered genes for a GFPPS (turquoise) and SmTS1 (pink) in *S. mobaraensis*.

A large scale incubation, followed by extensive compound purification by HPLC, standard and AgNO_3_ impregnated silica gel chromatography and structure elucidation by one‐ and two‐dimensional NMR spectroscopy (Tables S3–S9, Figures S7–S62) resulted in the structures of compounds **6**–**12** shown in the boxes of Scheme 1, for which we suggest the names sestermobaraenes A–F (**6**–**11**) and sestermobaraol (**12**). Their common biosynthesis can be explained from GFPP by 1,15‐14,18‐cyclization to cation **A**, followed by a 1,5‐hydride shift to **B**. These steps are also proposed for the cyclizations by some fungal and plant StTSs, but in most cases different stereoisomers of **A** and **B** are relevant.[^16^, ^20^, ^22^, ^23^, ^24^, ^25^, ^26^] Only for *Penicillium brasilianum* sesterbrasiliatriene synthase (PbSS) the cyclization cascade proceeds through the same intermediates **A** and **B**.^[22]^ Sestermobaraene E (**10**) with its unusual C3=C25 double bond localization can arise from **B** by a 1,5‐proton shift to **C** and deprotonation. Alternatively, **B** may undergo a 6,10‐cyclization to **D**, which is the direct precursor to sesterbrasiliatriene (**13**) in *P. brasilianum* (in brackets),^[22]^ but **13** was not observed as a product of the sestermobaraene synthase SmTS1. A 1,2‐hydride shift to **E**, 6,11‐2,12‐cyclization to **F** and deprotonation results in sestermobaraene F (**11**). From **D** in a folded conformation a 7,12‐cyclization to **G** and a 3,11‐cyclization to **H** are possible. In this secondary cation that may not be thermodynamically favored the hydrogen H12 is located directly underneath the cationic center which can thus undergo a possibly concerted 1,4‐hydride shift to **J**. Deprotonation then leads to the main product sestermobaraene A (**6**). From **H** an alternative (concerted) 1,2‐methyl migration to **K** and attack of water yields sestermobaraol (**12**). A 1,2‐hydride transfer from **G** to **L**, skeletal rearrangement to **M**, 2,7‐cyclization to **N** and deprotonation leads to sestermobaraene D (**9**). Cation **L** can also react by 2,12‐cyclization to **O** that yields sestermobaraene C (**8**) by deprotonation, or sestermobaraene B (**7**) by 1,4‐hydride shift to **P** and loss of a proton. This biosynthetic hypothesis was deeply investigated by enzymatic conversion of various isotopically labeled terpene precursors (Table S10). Specifically, all 25 isotopomers of (^13^C)GFPP, enzymatically prepared with GFPPS from the corresponding labeled GPP, FPP, GGPP or IPP isotopomers that were synthesized as shown in Scheme S6 or as reported previously,[^14^, ^35^, ^36^] resulted in the incorporation of the ^13^C‐label in the expected positions of compounds **6**–**12** in all cases (Figures S63–S87). These experiments supported the overall model, especially the proposed skeletal rearrangements from **H** to **K** and from **L** to **M**. The products obtained from (20‐^13^C) and (21‐^13^C)GFPP revealed a strict stereochemical course regarding the fate of the geminal methyl groups, without any distribution of labeling between these carbons, i. e. the 1,5‐hydride shift from **A** to **B** proceeds with high face selectivity at C19.

**Scheme 1:**
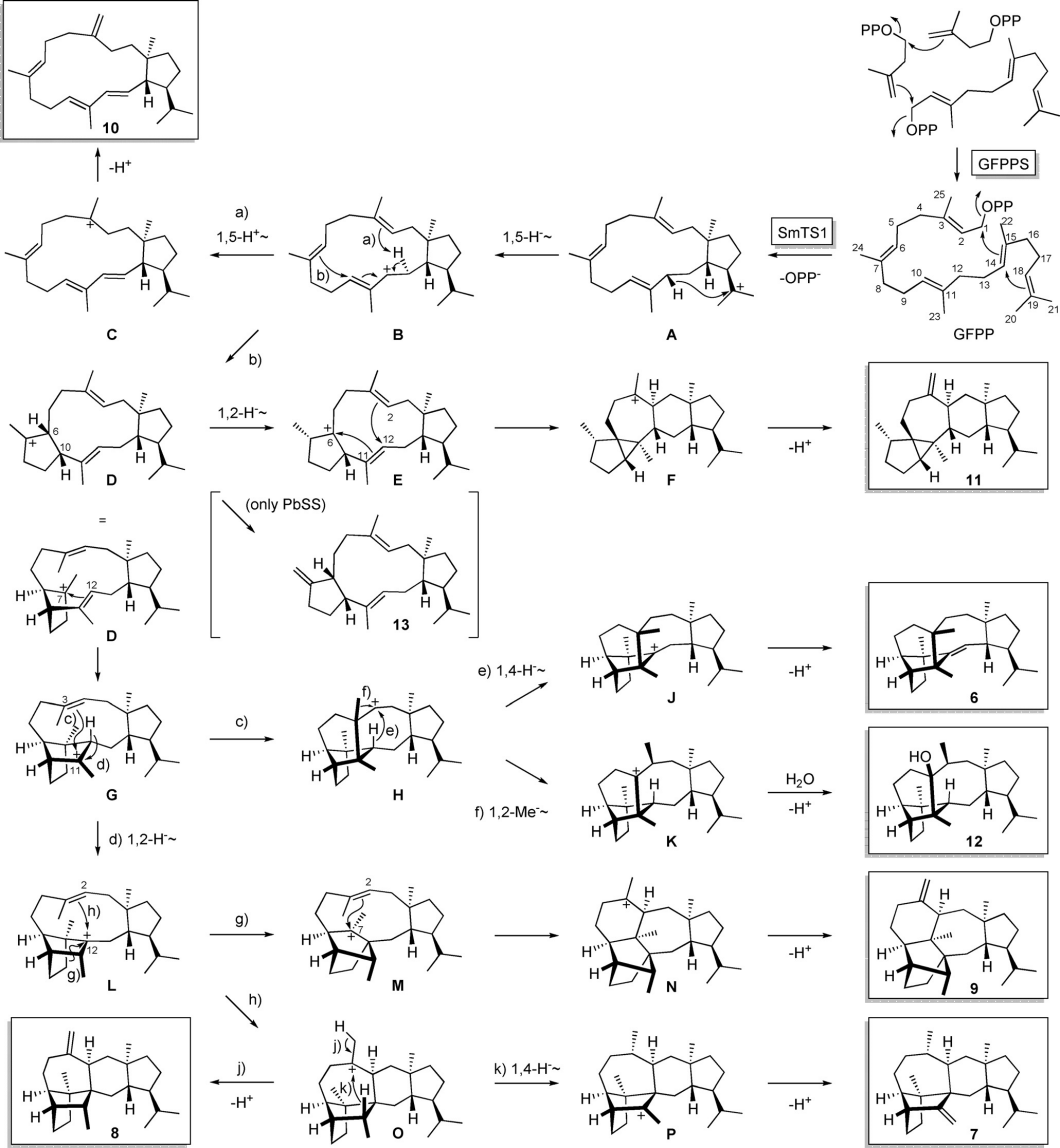
Biosynthesis of sestermobaraenes and sestermobaraol by SmTS1 in *S. mobaraensis*.

To follow the hydrogen migrations in the biosynthesis of **6**–**12**, experiments with stereoselectively deuterated and ^13^C‐labeled compounds were performed. The interpretation of the results made use of the known stereochemical course of the prenyltransferase reaction with inversion of configuration at C1 of the elongated oligoprenyl diphosphate and attack at C4 of IPP from the *Si* face.^[37]^ The 1,5‐hydride shift from **A** to **B** was investigated by conversion of (7‐^13^C)GPP and (*E*)‐ or (*Z*)‐(4‐^13^C,4‐^2^H)IPP^[38]^ with GFPPS and SmTS1 (Scheme S1). For the main compounds **6**–**8** the specific migration of deuterium from (*E*)‐(4‐^13^C,4‐^2^H)IPP to C19 was evident from an upfield shifted triplet by ^13^C‐^2^H spin coupling in the ^13^C‐NMR spectrum (Figures S88–S90). For the minor compounds **9**–**12** no such triplet signals were observable (Figures S91–S94), because for ^13^C connected to deuterium the transversal relaxation time for spin‐spin relaxation is increased, resulting in strongly reduced signal intensities in comparison to the intensities for carbons connected to protium. Furthermore, the nuclear quadrupole moment of deuterium causes line broadening. Indirect evidence for **9**–**12** was obtained by the clearly observable singlet for C19 in the experiment with (*Z*)‐(4‐^13^C,4‐^2^H)IPP that was not detected with (*E*)‐(4‐^3^C,4‐^2^H)IPP as a result of deuterium binding, suggesting that the 1,5‐hydride shift is also relevant for these compounds.

The 1,5‐proton shift from **B** to **C** towards compound **10** was investigated using (*R*)‐ and (*S*)‐(1‐^13^C,1‐^2^H)GPP^[36]^ with (2‐^13^C)IPP,^[14]^ GFPPS and SmTS1 (Scheme S2). While for (*S*)‐(1‐^13^C,1‐^2^H)GPP a singlet was observed for C2 in the ^13^C‐NMR spectrum, no signal was detected with (*R*)‐(1‐^13^C,1‐^2^H)GPP (Figure S95). Analysis of the enzyme products by GC/MS revealed a strongly diminished production of **10** from (*R*)‐(1‐^13^C,1‐^2^H)GPP caused by a deuterium kinetic isotope effect. Product analysis by the more sensitive HSQC spectroscopy in comparison to ^13^C NMR demonstrated that with (*S*)‐(1‐^13^C,1‐^2^H)GPP the crosspeak for H13 was vanished, while with (*R*)‐(1‐^13^C,1‐^2^H)GPP no crosspeak for H2β was detected. Taken together, these data support the 1,5‐proton shift from **B** to **C** with specific migration of the 1‐*pro*‐*R* proton of GPP into the H2 position of **10**. The same experiment also demonstrated the specific loss of the 1‐*pro*‐*S* proton of GPP in the deprotonation step to **6**, as was concluded based on GC/MS and ^13^C‐NMR data (Figure S96).

The 1,2‐hydride shift from **D** to **E** was shown by conversion of (3‐^13^C,2‐^2^H)GGPP^[36]^ and IPP with GFPPS and SmTS1 (Scheme S3), resulting in an upfield triplet for C7 of **11** in the ^13^C‐NMR (Figure S97). Along similar lines, the 1,4‐hydride transfer from **H** to **J** towards **6** was shown by a two‐step enzymatic transformation first of GPP and (*Z*)‐(4‐^2^H)IPP^[39]^ with *Streptomyces coelicolor* FPPS,^[40]^ followed by the addition of (2‐^13^C)IPP,^[14]^ GFPPS and SmTS1 (Scheme S4 a), resulting in a triplet for C2 of **6** (Figure S98). The formation of other isotopomers, for example, from unreacted GPP after the first step (Scheme S4 b), could not completely be suppressed and gave rise to additional ^13^C‐NMR signals for C2 of **6**.

The 1,2‐hydride migration from **G** to **L** in the biosynthesis of **8** and **9** (Scheme S5) was followed by incubation of GPP and (3‐^13^C,4,4‐^2^H_2_)IPP, synthesized as in Scheme S7, with GFPPS and SmTS1. For C11 of compound **8** an upfield shifted triplet was observed, while the signals for the other labeled carbons were clearly visible and appeared as doublets because of ^13^C‐^13^C spin coupling (Figure S99). The upfield shift of these signals is a result of two deuterium atoms bound to neighboring carbons. For the minor product **9** it was impossible to detect a triplet for C11. For compound **7**, the same hydrogen is passed on in the 1,4‐hydride shift from **O** to **P**, as was shown by the triplet for C3 obtained from the same incubation experiment (Figure S100). Here the unusually large upfield shift is caused by the simultaneous effect of one directly bound deuterium atom and two deuterium atoms in the neighboring position.

The absolute configurations of **6**–**12** were determined by an enantioselective labeling strategy with enzymatic conversion of GPP and (*R*)‐ or (*S*)‐(1‐^13^C,1‐^2^H)IPP^[41]^ (Scheme 2). As a consequence of the ^13^C‐labels (black dots), the introduced stereoselective deuterations at C1, C5 and C9 can efficiently be monitored by HSQC spectroscopy (Figures S101–S107). The relative orientation of the natural stereogenic centers in **6**–**12** to the stereogenic anchors at the deuterated carbons can be deduced by NOESY and gives rise to the absolute configurations of all seven sesterterpenes (red and blue arrows show key NOESY correlations). Similar experiments with GPP and (*E*)‐ or (*Z*)‐(4‐^13^C,4‐^2^H)IPP^[38]^ gave additional stereochemical labels at C4 and C8 (the information at C12 is lost), allowing for the same conclusions on the absolute configurations of **6**–**12**.

**Scheme 2:**
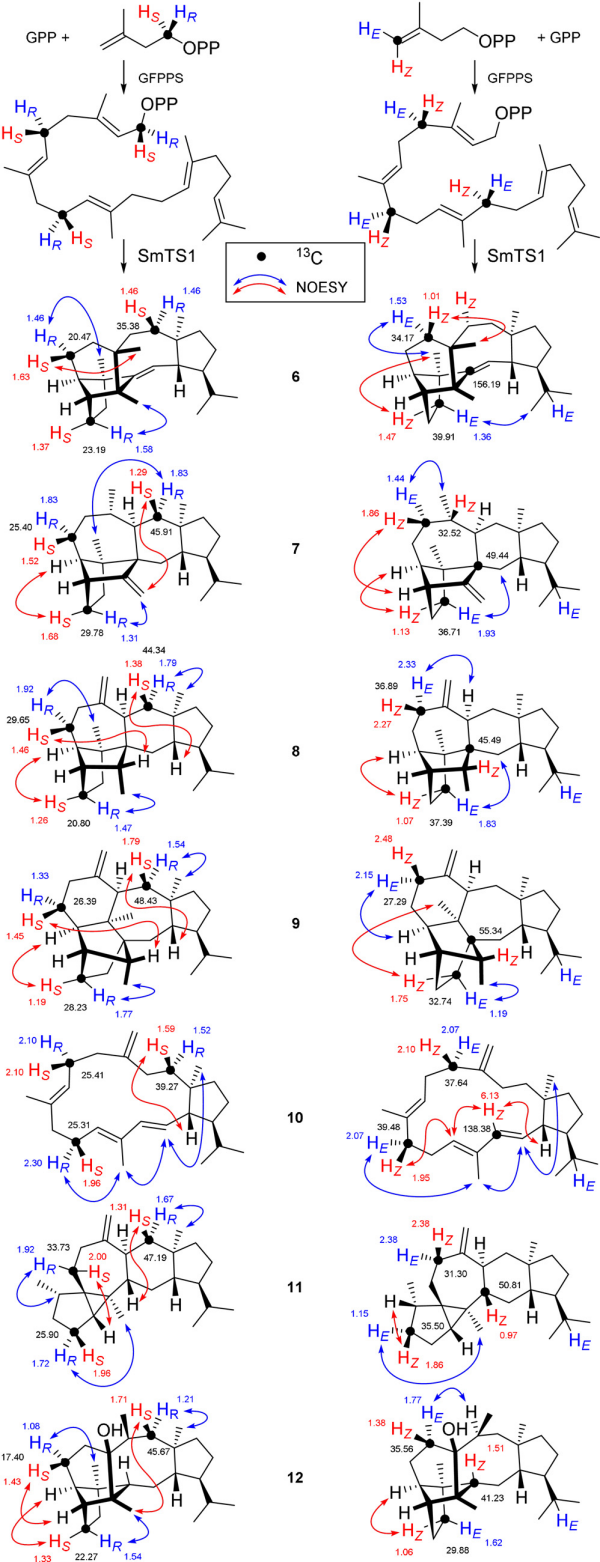
The absolute configurations of **6**–**12**, by enantioselective deuteration (H_*R*_=^2^H or H_*S*_=^2^H; H_*E*_=^2^H or H_*Z*_=^2^H). Numbers at C and H atoms indicate chemical shifts for unlabeled **6**–**12**.

In summary, we have identified a biosynthetic gene cluster in *S. mobaraensis* encoding the showcase representatives of a bacterial GFPPS and a type I StTS. The remarkable structures of seven new sesterterpenes were elucidated, including the absolute configurations by an enantioselective deuteration approach. The complex cyclization mechanism from GFPP to all seven identified products was investigated in a series of labeling experiments that allowed to follow every elementary step including skeletal rearrangements, hydride and proton migrations, and a stereospecific deprotonation. Whether the polyketide synthase encoded by a gene adjacent to the StTS and GFPPS genes is functionally related and for example, involved in the biosynthesis of a bacterial meroterpenoid, an important natural product class in *Streptomyces*,^[42]^ is currently unknown. However, no candidate compound for such a hypothetical biosynthetic pathway has been described, and the detection of the same sesterterpenes in the headspace of *S. mobaraensis* cultures as in the in vitro incubations performed in this study disfavors this hypothesis.

## Conflict of interest

The authors declare no conflict of interest.

## Supporting information

As a service to our authors and readers, this journal provides supporting information supplied by the authors. Such materials are peer reviewed and may be re‐organized for online delivery, but are not copy‐edited or typeset. Technical support issues arising from supporting information (other than missing files) should be addressed to the authors.

Supplementary
