# Supplementary material for: The Biosynthetic Gene Cluster for Sestermobaraenes—Discovery of a Geranylfarnesyl Diphosphate Synthase and a Multiproduct Sesterterpene Synthase from Streptomyces mobaraensis
Source: Angew Chem Int Ed Engl. 2020 Aug 31;59(45):19961–5. doi: 10.1002/anie.202010084 (PMC7693059; doi:10.1002/anie.202010084)
Supplement: Supplementary file 1 — Supplementary [file ANIE-59-19961-s001.pdf]

Angewandte Chemie
Eine Zeitschrift der Gesellschaft Deutscher Chemiker

Supporting Information

The Biosynthetic Gene Cluster for Sestermobaraenes—Discovery of a Geranylfarnesyl Diphosphate Synthase and a Multiproduct Sesterterpene Synthase from *Streptomyces mobaraensis*

*Anwei Hou and Jeroen S. Dickschat**

anie_202010084_sm_miscellaneous_information.pdf

Author Contributions

A.H. Investigation: Lead; Writing - Original Draft: Supporting; Writing - Review & Editing: Supporting.

Table of Contents

Strains, culture conditions, CLSA and GC/MS analysis	S1
GC/MS analysis of CLSA extracts and enzyme products from SmTS1	S2
Type I terpene synthase homologs from *S. mobaraensis*	S5
Phylogenetic tree for bacterial terpene synthase homologs	S6
Amino acid sequences of SmTS1 – SmTS10	S7
Gene cloning and expression	S9
Enzyme purification	S11
Incubation experiments with unlabeled substrates	S12
Compound purification procedure and analytical data for **6** – **12**	S14
NMR data of sestermobaraene A (**6**)	S16
NMR data of sestermobaraene B (**7**)	S25
NMR data of sestermobaraene C (**8**)	S34
NMR data of sestermobaraene D (**9**)	S43
NMR data of sestermobaraene E (**10**)	S52
NMR data of sestermobaraene F (**11**)	S61
NMR data of sestermobaraol (**12**)	S70
List of labeling experiments performed in this study	S79
Enzymatic conversion 25 isotopomers of (^{13}C)GFPP	S80
Enzymatic conversion (7-^{13}C)GPP and (*E*)- or (*Z*)-(4-^{13}C,4-^{2}H)IPP	S105
Enzymatic conversion (*R*)- or (*S*)-(1-^{13}C,1-^{2}H)GPP and (2-^{13}C)IPP	S113
Enzymatic conversion (3-^{13}C,2-^{2}H)GGPP and IPP	S116
Enzymatic conversion GPP, (*Z*)-(4-^{2}H)IPP and (2-^{13}C)IPP	S118
Enzymatic conversion GPP and (3-^{13}C,4-$^{2}H_2$)IPP	S121
Determination of the absolute configurations of **6** – **12**	S124
Synthesis of (5-^{13}C)IPP	S131
Synthesis of (3-^{13}C,4-$^{2}H_2$)IPP	S134
References	S137

Strains and culture conditions

The strain *Streptomyces mobaraensis* NBRC 13819 (=NRRL B-3729) was obtained from the NRRL culture collection and cultured in SFM medium (2% mannitol, 2% soja flour, pH 7.2) or medium 65 GYM (0.4% glucose, 0.4% yeast extract, 1% malt extract, pH 7.2) at 28 °C. *Saccharomyces cerevisiae* FY834 was cultured in YPAD medium (1% yeast extract, 2% peptone, 2% glucose, 0.04% adenine sulphate) or SM-URA medium (0.17% yeast nitrogen base, 0.5% ammonium sulphate, 2% glucose, 0.077% nutritional supplement minus uracil). *E. coli* BL21 (DE3) was cultured in LB medium (1% tryptone, 0.5% yeast extract, 0.5% NaCl). For agar plate cultures, 1.5% agar was added.

CLSA headspace extraction

The volatile organic compounds from an agar plate culture of *Streptomyces mobaraensis* were collected by a closed loop stripping apparatus (CLSA).[1] The emitted compounds were absorbed on charcoal for 24 hours and eluted with dichloromethane (30 μL). The obtained sample was immediately analyzed by GC/MS.

GC/MS

GC/MS analyses were performed on a 5977A GC/MSD system (Agilent, Santa Clara, CA, USA) with a 7890B GC and a 5977A mass selective detector. The GC was equipped with a HP5-MS fused silica capillary column (30 m, 0.25 mm i. d., 0.50 μm film). Specific GC settings were 1) inlet pressure: 77.1 kPa, He at 23.3 mL min^{-1}, 2) injection volume: 2 μL, 3) temperature program: 5 min at 50 °C increasing at 5 °C min^{-1} to 320 °C, 4) 60 s valve time, and 5) carrier gas: He at 1.2 mL min^{-1}. MS settings were 1) source: 230 °C, 2) transfer line: 250 °C, 3) quadrupole: 150 °C and 4) electron energy: 70 eV. Retention indices (*I*) were determined from retention times in comparison to the retention times of of *n*-alkanes (C_7-C_{40}).

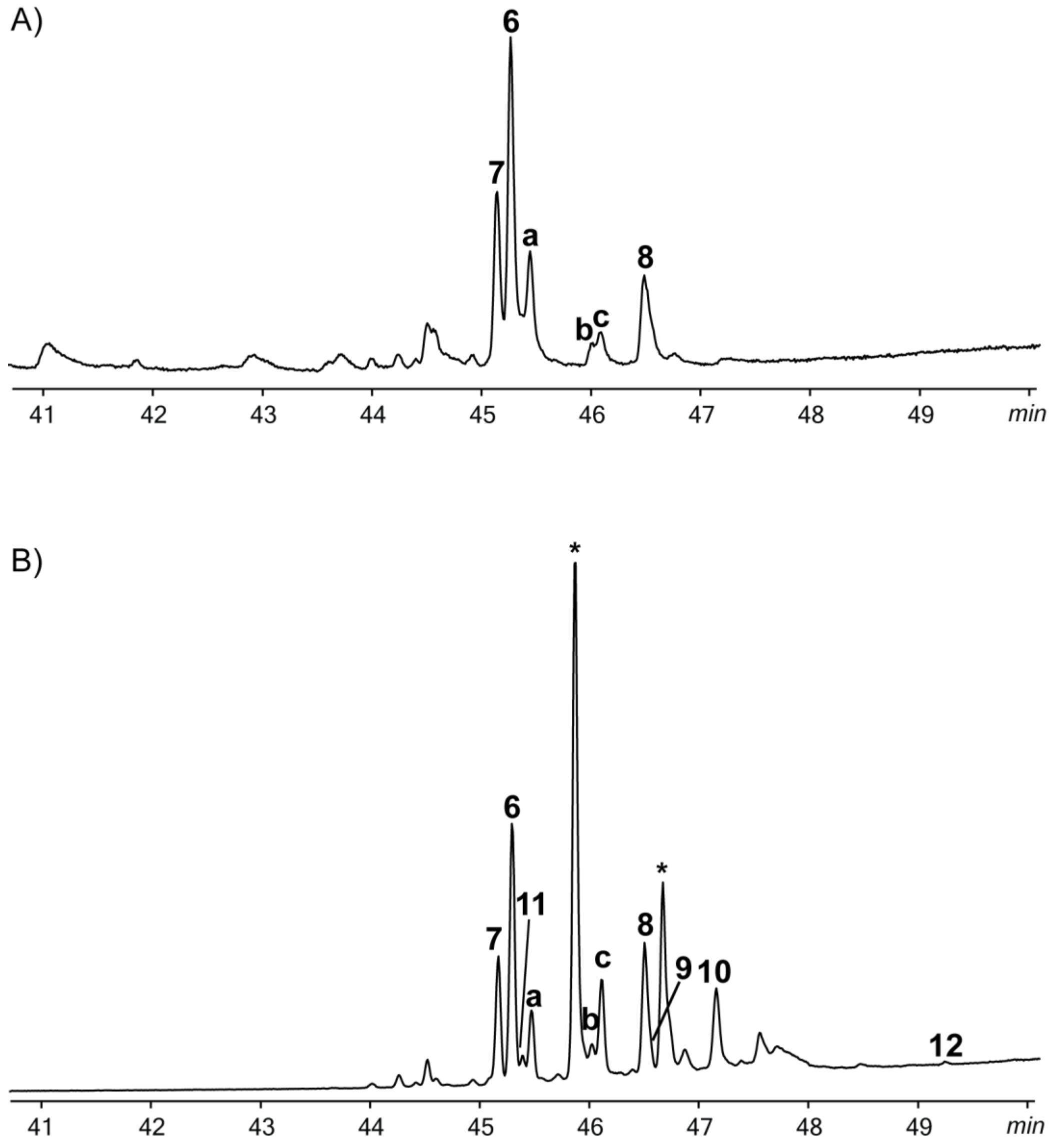

Figure S1. Total ion chromatograms of A) a CLSA headspace extract from an agar plate culture of *S. mobaraensis* NBRC 13819, and B) an extract of an incubation of GFPP with SmTS1. The asterisks indicate non-enzymatic degradation products from GFPP.

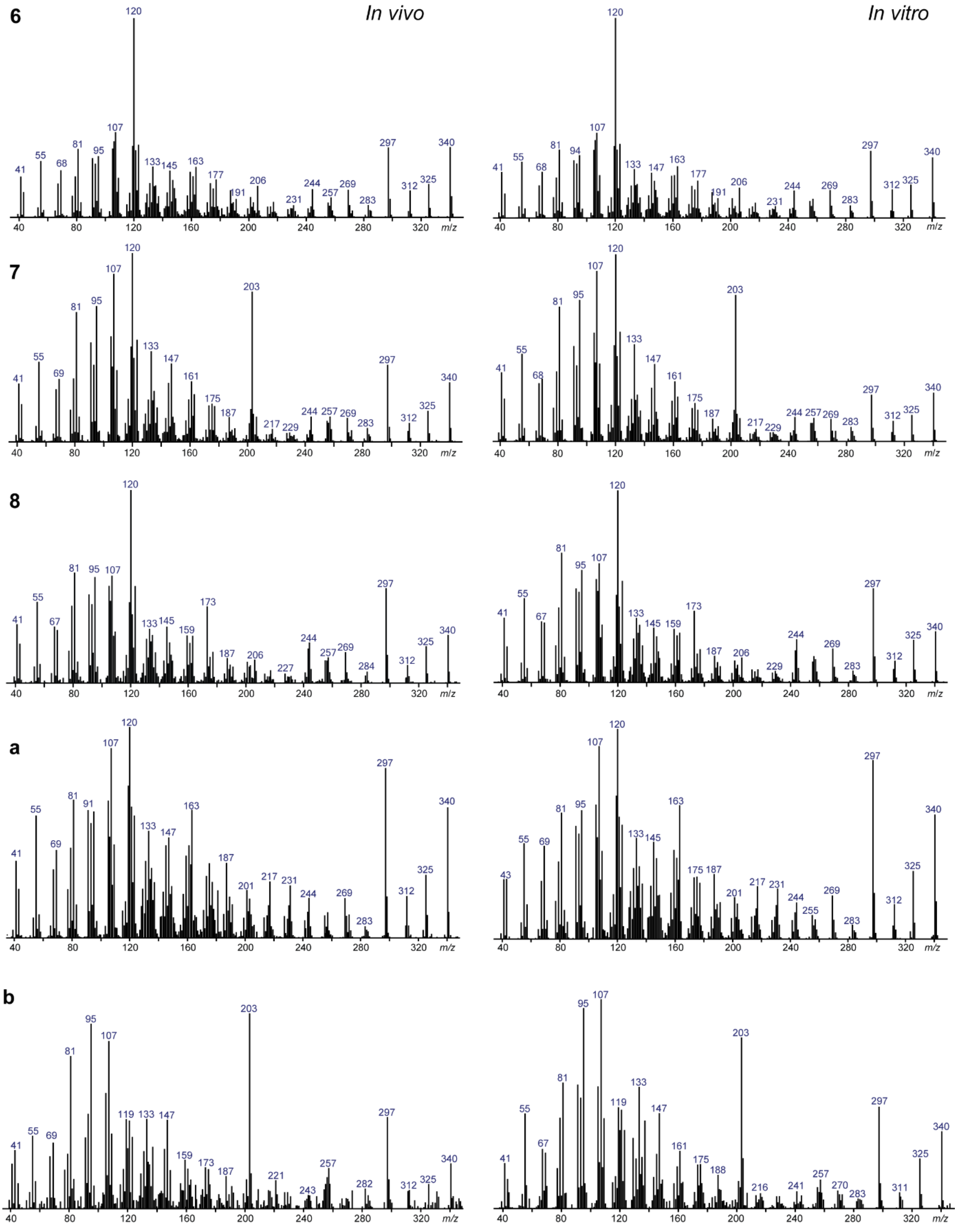

Figure S2. EI mass spectra of A) sestermobaraene A (**6**), B) sestermobaraene B (**7**), C) sestermobaraene C (**8**), D) sestermobaraene D (**9**), sestermobaraene E (**10**), F) sestermobaraene F (**11**), G) sestermobaraol (**12**), and further unknown compounds (**a**, **b**, **c**). The mass spectra on the left are from compounds in CLSA headspace extracts and the mass spectra on the right are from enzyme products.

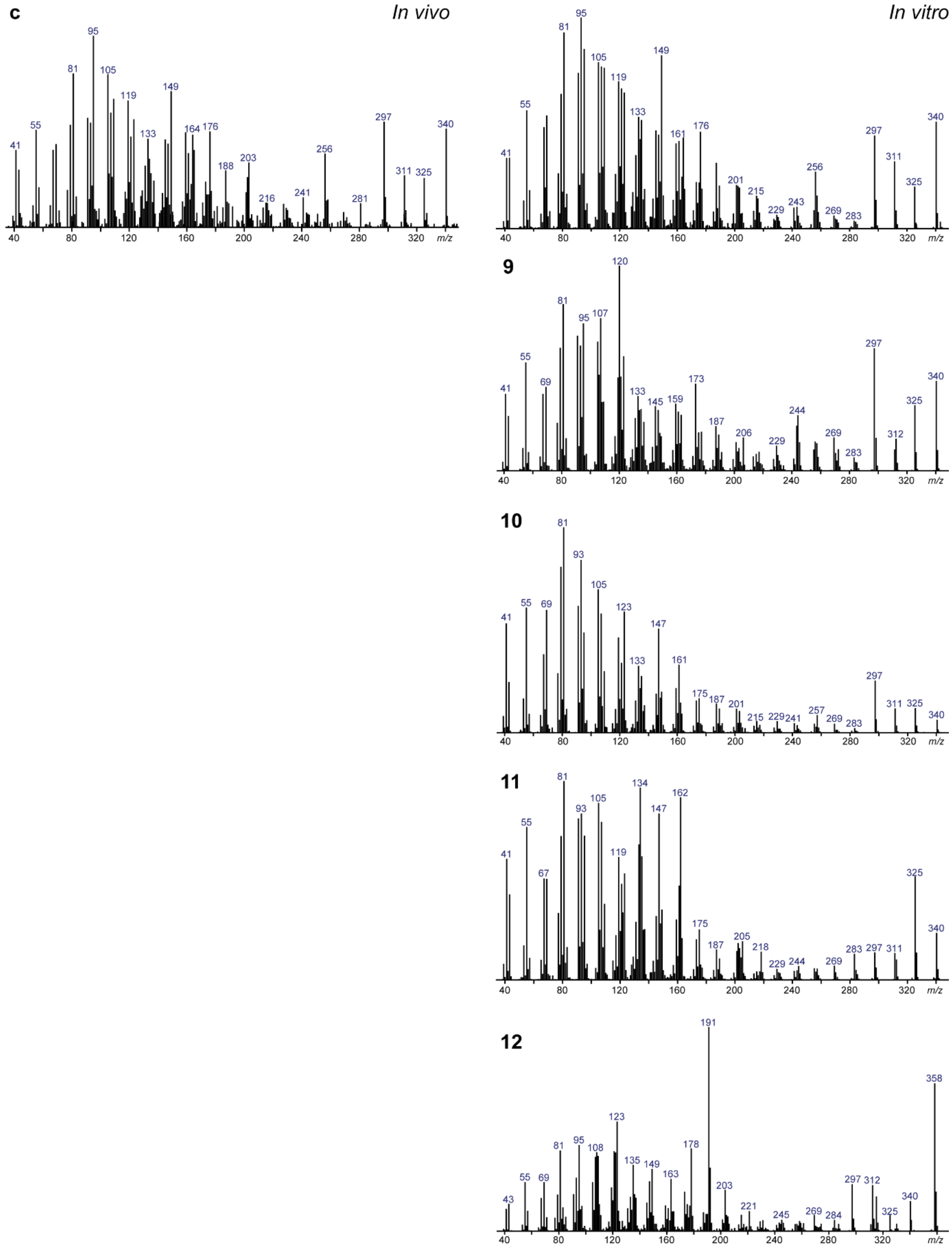

Figure S2 (continued). EI mass spectra of A) sestermobaraene A (**6**), B) sestermobaraene B (**7**), C) sestermobaraene C (**8**), D) sestermobaraene D (**9**), sestermobaraene E (**10**), F) sestermobaraene F (**11**), G) sestermobaraol (**12**), and further unknown compounds (**a**, **b**, **c**). The mass spectra on the left are from compounds in CLSA headspace extracts and the mass spectra on the right are from enzyme products.

Table S1. Type I terpene synthase homologs from *S. mobaraensis*.

accession number	TS no.	(predicted) function
WP_004941320	SmTS1	sestermobaraene synthase
WP_004945508	SmTS2	2-MIB synthase
EME96605	SmTS3	geosmin synthase
WP_004939181	SmTS4	(insoluble)
WP_004942276	SmTS5	(insoluble)
WP_004954463	SmTS6	sesquiterpene/diterpene synthase
WP_004952180	SmTS7	no activity
WP_004952004	SmTS8	sesquiterpene/diterpene synthase (low activity)
WP_004954462	SmTS9	sesquiterpene/diterpene synthase (low activity)
WP_004954459	SmTS10	sesquiterpene synthase (low activity)

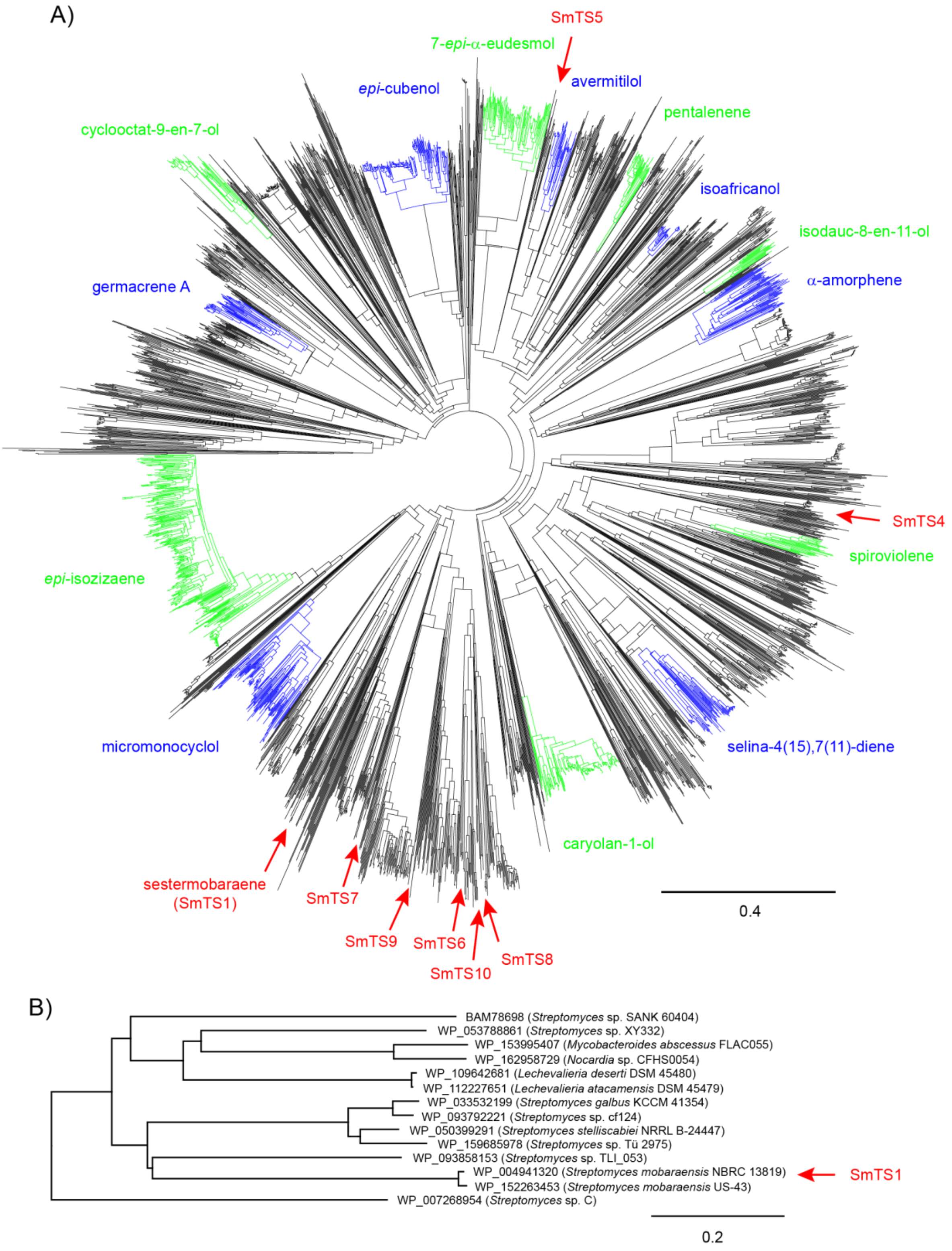

Figure S3. A) Phylogenetic tree constructed from 3267 amino acid sequences of bacterial terpene synthase homologs using the tree builder function of Geneious (alignment type: global alignment with free end gaps, cost matrix: Blosum45, genetic distance model: Jukes-Cantor, tree build method: neighbor-joining, gap open panelty: 8, gap extension penalty: 2). The largest branches representing functionally characterized enzymes[2-14] and their closest relatives with likely the same function are shown in green and blue. B) Expansion for the branch containing SmTS1 and its closest relatives. The red arrows indicate the terpene synthases from *S. mobaraensis*. The scale bar indicates the number of substitutions per site.

SmTS1

MTLNPVPDDFLTFYCPIPGEVGPDGDKRVERTLAWVRSYDFGSGDDMANTMYAHTGVTLVTHLFPHAT
GDLAQALDDYNTWAFLANDLTVPDHRTVRTTDAVRLIARWTQILRIPHIFDDTSPGEAALGDALSRLR
QLTTPVQFDRFAKGQARWLWGQAWEAHVREHDSRMTVNEHLTLGYAVGGPEATPPIVEVAEGIEVPER
ELASLPVRAAVDAAMTTAVFDNQRYSYFKESAHAQPKRSMFDTILHNNPGRTLQEAMHEGVAIRDRAL
ACYLRLRDRILPHASPQLRQYLAGLDLVLSGHLTFAAKALRYLTPGHAVTITPTPPPHLPTEPLPYPA
VAWWWDQIDPHSARQRDLG

SmTS2

MPDPGARGPSVPAPPSEPAGTVPDVPTPSAPDLPIPAAAFALAAMPRPPVAHPFPPSTGTPPAPPSEP
AAVPTAARVLGGPTGLGTTALSLAGAGAVAPSEPASVSEPAGVSEPASVSEPVIVSAPAIAPDPQRVL
GGPSGLGTAALSLASLALTSHRPPPEPAPAPPAEGQAVPGLYHHPVPEPDPVRVEEVSRRIKRWAEDE
VRLYPEEWEGQFDGFSIGRYMVGCHPDAPTVDHLMIAARLMVAENAVDDCYCEDHGGSPVGLGGRLLL
AHTALDPLHTTQEYAPAWEESLGSDPPRRAYRSAMDYFVRMATPSQADRFRHDMARLHLGYLAEAAWA
ETDHDPEVWEYLAMRQFNNFRPCPTITDTVGGYELPADLHARPEMQRVIALAGNATTIVNDLYSYTKE
LASPGKHLNLPVVLAERRQLSERDAYLKAVEIHNELMHSFEAAAAELAAHCPLPSVVRFLKGVAAWVD
GNHDWHRTNTYRYSLPDFW

SmTS3

MTQPFTLPAFYMNHPARLNPHLEEARAHAREWARGMGMLEGSGVWDQADLDAHDYALLCAYTHPDCDG
PALSLVTDWYVWVFFFDDHFLELFKRTQDREGGRAYLDRLPAFMPMDPGAAVPEPTHPVEAGLADLWA
RTVPHMSADWRVRFAESTRNLLDESLWELSNINEGRIANPVEYIEMRRKVGGAPWSAGLVEYAAGAEV
PASVAGTRPMRVLSDTFADAVHLRNDIFSYQREVEDEGELSNGILVLETFLDCGTQEAAESVNNLLTS
RMQQFEHTVVTELPQLFVDHALGPDDCRAVLAYAKGLQDWQAGGHEWHMRSSRYMNKEAAPIGLPGPT
GPGTSGLDLRHLFAATGAQRLRSLTHVPHQHVGPSELPDFDVPFTLRLNPHLERSRPNTVAWAREMGM
LAPQPGVPTSYIWDERKLYGYDFPLCAAGLHPDATPEELDLASQWLTWGTYGDDYYPVVYGRPRDLAG
AKLAHARLLTLMPLDPAGAPAPVPPTALERGLADLWERTAGPMDAAGRSQLRDAVEVMLESWLWELQN
QAEHRVPEPVDYIEMRRATFGSDLTIALCRVGHGRAVPDEVYRSGTVRSLENAAIDYATLLNDVFSYQ
KEIEYEGEVHNAVLVVQTFFGCDRRAALAIVDDLMRGRMSQFRHVAMHELPVLYEDAGLGPVARAALE
GYVKELENWLAGILNWHRGCRRYRQEDLERNFRFLPAAEPSAFAPAVPWGASR

SmTS4

MRVVLAPRYCPFASPAHPDAWRMTERGGRWLNGFGLGLREPERSRMLNNDCGEFYGRIMPRAPAGRLQ
LAVDWCTLMFAFDDAHCDEGAAGVRPGDFARLASRVLRVLEVPRAVMGPPGDVLLAAVRDLAERGRTW
STAAQWARVVEEHRGWFYGVLWEFACRAQDRTPALNDYAHMRQHTAAGPATLCWAEIVDGVEIPERDM
ASPAVRALRELAFTTAAFDDDLFSYGKERWLAARSAHATGCRLNLVDILAQERGLPVHKALEEAVALT
NRLTARFARLREQVLPTASGPLRQHLDHLSTLLRGNAEWGLRAGRYSDPDGRHPGAVVTTGSFTDTAP
PAGAPGIAALSWLWELPGYG

SmTS5

MPVDARIRFPAGFRFAVSPHEEEAREGNRAWARAHRMADGPEATALYDSWDLARVSACMYPYATAPGL
RLVTDHMGFWYPFDDQFDSPLGLDPAATARACEELIAVVHHGTTGPRPSAAARAFADIWRRAADGMPP
AWRARAAHDWEYYFASYAQESAARLAGTVPDFPTFLRLRLGVSAMSVVCDLVERIDGYEVPAVAFHSL
PLIELRRLTEELPCLANDVYTLDREEPRGDVVNLVLVLERDGHRPRAAAIDTAYALVNDRLRRFDALR
RGVPALARTLGLDARRRAAVERYARDLEFLVSGYMAWGADTRRHFPETVVPPPDRPGYPENLLLPTTP

Figure S4. Amino acid sequences of SmTS1 – SmTS10. Highly conserved motifs are marked by yellow background.

SmTS6
MTGSGEPSTGGWKNHLGVPVPRYPWPWVPSPFKDHVLSAECHWYDTDYTFLSADTLEKYKRHGLTQVT
AYIFPVDDVEAVLLASRLLIFHTVFDDYFELCPATEMAAIRDHLIAVLLGEPPTPTDLGLFRQVAAVR
DECRKAGMPDFWFERLADSFHRYITYGVMEEVPYKLAGVFPSLAYGLSIHDAAIGMRPHFTMAELVND
CLLPEVVYRHPMLQRLLDVHHRLFVVQNDLFSLDREIHRETEVINHILALRHWEGMSLHDACADVMRM
NDRYVKEAADLHAALLEVPSFAPFRETIDRYARSLETILTGMNQWYQEGRSIRYDASGGYPEPEYASP
RPSRRDT

SmTS7
MTLLEPQRMRDHQPTDALRLYCPLPRHEYPDHKGLVEATQEWSRPYISYSSDRDQDILDSCAYSAYTC
IPPRVPHDLRVWFSCVSAWTLLSDDSFDRGTLQQRQQRYAEIAPQELHALSTPLAIPEPGRLHPLADY
IWALRTYGEEHAPLPPVRMLMDAIDRALRETETEIGYAVRSELCGADEYIRRLMSGAKYSGQFAILPE
ICTRATIPEHEGQHPIMQGLRYLTGTLCHLHLDFFCWANDGPTEAYNIINAVAQDLGIPPYEAGAPAL
ELTNRIMELFLRLREQLKREVGEASRTLLDDLDWMVRGNLDWGLYTTRYASDNNPMRPFLPEEQPTPL
APPALTSAPCPALAHLFGLLD

SmTS8
MPDTLPVPELSYPFPTVVSPFETDESFHDEREWYETYYGFLPEGDRAKYKEHDLAQGAAYMSPTVTDP
ARLRPMARWFVFLTQIDDYHEFHTVPELVAVRDRVRDILSGEEPHDGEPGHLRFLARMRAEFQEFMPH
LWLERLAHSFHEFFTYGVMEEAPYKLGKRSGPPSLAHYLLIHEYSIAMRPYGDLVEPAMGGVLPESVF
RHPVIQRLRSLICRLMSVQNDLHSLAKEEARPSECVNIVPVLCHELGCTRDEAVAEAVRILDSFSAEI
LALGRQLPDFGPYQEGVREYFRQMQLMVTGLERWYRYSRSTRYRIPGAFPDVAPEARRRP

SmTS9
MDPSEMARLRYPFPSLMNPYAEALQEHTDRAWIDGEWAGIVPPEIAEGFKKVKTAYMTAFFFPSATWE
RLIPLGRMMLFSLYQDDVYERATPDLVRHLRQRTVAVARGEITPREAGVPLARAVAQIRTDALSFIPP
TSVARWADDLDLYFQGLEAETRHLAAGTVPGIDEYMAMREKALMIHPFLALKEIETGTVLPEEIHDHP
VIRRLKSLTVRITGWFNEFQSYDKDMRTGMGNVNLINVLAHHQRIRVDQAREEMFALHDRELDEFVRL
QRSLPDFGSWTDAVAQHVHHFSFVISGWRGVDRHVHRYDPEYYPDQDALRATAGKEEDLTSmTS10

SmTS10
MTDADSQDPTAPGQSDTGEELVIPRLTYPWPTIDSPLADALDEETFRWYDEDYADMFPDPADRERYRK
QLLSRVTPYMFPTTDDIDRLRPAARWMNYITLMDDFFDLTPAEEIAPLRDRIYQVMTGRDDPGPDELG
LLRQMAAAREEFRRYAPQSWIERMALSYWQYIHYGLMEEIAFRRQGVYPSIYRCRMIRMHSIGMRPFA
DQLEPVTGLLLPADVFHHPVIQRLRDLQACVIYLQNDISSLYKELALGQNEVVNQILTIRHHRQVSLQ
RAVDEVVAMHDRDVEEMWSLQQCLPDFGPHQAAASNYVRHLGIQVVGLQNWYDEIGRQFRYDRGGFVT
AQYGREEIEIKTKTSYVNPDGTPYRPPRIHGPGWEERRPAEVIDNTQRYVDDNGTPWQGQG

Figure S4 (continued). Amino acid sequences of SmTS1 – SmTS10. Highly conserved motifs are marked by yellow background.

Gene cloning and expression

S. mobaraensis was grown in medium 65 (0.4% glucose, 0.4% yeast extract, 1% malt extract, pH 7.2) at 28°C for 1 week. The cells were collected by centrifugation and the genomic DNA was extracted by using a standard phenol/chloroform extraction protocol.[15] The obtained gDNA, the primers according to Table S2, and Q5®-polymerase (NEB, Ipswich, MA, USA) were used for PCR. The standard protocol for PCR was: initial denaturation at 98 °C for 40 sec, followed by 30 cycles of a 3 steps program (denaturation at 98 °C for 10 sec, annealing at 60 – 67 °C for 30 sec, elongation at 72 °C for 45 sec) and final elongation at 72 °C for 2 min. The primers indicate the target sequence (accession number) in their names. Short primers were used to amplify the gene from the gDNA, then the longer primers were used in a second PCR with the first PCR product as a template to attach the homology arms (underlined) for homologous recombination in yeast with the linearized pYE-Express vector (EcoRI and HindIII digestion).[16] All PCR products were analyzed by gel electrophoresis and purified by using the Wizard® SV Gel and PCR Clean-Up System (Promega, Madison, WI, USA). Homologous recombination was performed by the PEG/LiOAc method.[17] After culturing the yeast for three days, the plasmid containing the integrated gene was isolated from the yeast by using the Zymoprep™ Yeast Plasmid Miniprep II kit (Zymoresearch, Irvine, CA, USA), followed by introduction to *E. coli* BL21 (DE3) through electroporation. The transformants were cultured on LB agar plates (1% tryptone, 0.5% yeast extract, 0.5% NaCl, 1.5% agar) with kanamycin (50 μg/mL) at 37 °C overnight. Single colonies were selected and grown in liquid LB medium overnight for plasmid DNA isolation by using PureYield™ Plasmid Miniprep System (Promega, Madison, WI, USA). The sequences of the cloned genes were verified by DNA sequencing.

The *E. coli* transformants harbouring a correct plasmid were pre-cultured in liquid LB medium and grown at 37 °C overnight. The preculture was used to inoculate an expression culture (0.1%) and incubated at 37 °C until an OD_{600} of 0.4 – 0.6 was reached. The expression culture was then cooled to 18 °C and IPTG (0.4 mM) was added to induce protein expression, followed by incubation at 18 °C for 16 – 18 h. The grown cells were collected by centrifugation and used for protein purification immediately or stored at –80 °C.

Table S2. Primers used in this study.

Primer name	Target	Nucleotide sequence (5’ → 3’, homology arms are underlined)
AH005f_WP004941320	(SmTS1)	GTGACGCTCAACCCGGTC
AH005r_WP004941320	(SmTS1)	CTATCCCAGGTCTCGTTGTCG
AH006f_WP004941320	(SmTS1)	GGCAGCCATATGGCTAGCATGACTGGTGGAGTGACGCTCAACCCGGTC
AH006r_WP004941320	(SmTS1)	TCTCAGTGGTGGTGGTGGTGGTGCTCGAGTCTATCCCAGGTCTCGTTGTCG
AH012f_WP004941318	GFPPS	ATGGACGGACCTGCCG
AH012r_WP004941318	GFPPS	TCACTGGTCGCGTTCGG
AH013f_WP004941318	GFPPS	GGCAGCCATATGGCTAGCATGACTGGTGGAATGGACGGACCTGCCG
AH013r_WP004941318	GFPPS	TCTCAGTGGTGGTGGTGGTGGTGCTCGAGTTCACTGGTCGCGTTCGG
AH001f_WP004939181	(SmTS4)	GTGCGGGTCGTGCTCG
AH001r_WP004939181	(SmTS4)	TCAGCCGTACCCGGGGAG
AH003f_WP004939181	(SmTS4)	GGCAGCCATATGGCTAGCATGACTGGTGGAGTGCGGGTCGTGCTCGC
AH003r_WP004939181	(SmTS4)	TCTCAGTGGTGGTGGTGGTGGTGCTCGAGTTCAGCCGTACCCGG
PR028f_WP004942276	(SmTS5)	ATGCCCGTCGACGCCAGGAT
PR028r_WP004942276	(SmTS5)	TCAGGGTGTGGTGGGCAGCA
PR027f_WP004942276	(SmTS5)	GGCAGCCATATGGCTAGCATGACTGGTGGAATGCCCGTCGACGCCAGGAT
PR027r_WP004942276	(SmTS5)	TCTCAGTGGTGGTGGTGGTGGTGCTCGAGTTCAGGGTGTGGTGGGCAGCA
AH004f_WP004954463	(SmTS6)	ATGACCGGTTCCGGTGAACCATC
JR124r_WP004954463	(SmTS6)	TCAGGTGTCGCGCCTGC
AH002f_WP004954463	(SmTS6)	GGCAGCCATATGGCTAGCATGACTGGTGGAATGACCGGTTCCGGTGAACCATC
JR125r_WP004954463	(SmTS6)	TCTCAGTGGTGGTGGTGGTGGTGCTCGAGTTCAGGTGTCGCGCCTGC
AH007f_WP004952180	(SmTS7)	ATGACACTTCTCGAACCGCAG
AH007r_WP004952180	(SmTS7)	TCAGTCGAGCAGGCCG
AH008f_WP004952180	(SmTS7)	GGCAGCCATATGGCTAGCATGACTGGTGGAATGACACTTCTCGAACCGCAG
AH008r_WP004952180	(SmTS7)	TCTCAGTGGTGGTGGTGGTGGTGCTCGAGTTCAGTCGAGCAGGCCG
PR030f_WP004952004	(SmTS8)	ATGCCCGACACGTTGCCGGT
PR030r_WP004952004	(SmTS8)	TCAAGGGCGCCGGCGGGCTT
PR029f_WP004952004	(SmTS8)	GGCAGCCATATGGCTAGCATGACTGGTGGAATGCCCGACACGTTGCCGGT
PR029r_WP004952004	(SmTS8)	TCTCAGTGGTGGTGGTGGTGGTGCTCGAGTTCAAGGGCGCCGGCGGGCTT
JR122f_WP004954462	(SmTS9)	ATGGACCCGAGCGAGATGG
JR122r_WP004954462	(SmTS9)	TCACGTCAGGTCCTCTTCCTTTCC
JR123f_WP004954462	(SmTS9)	GGCAGCCATATGGCTAGCATGACTGGTGGAATGGACCCGAGCGAGATGG
JR123r_WP004954462	(SmTS9)	TCTCAGTGGTGGTGGTGGTGGTGCTCGAGTTCACGTCAGGTCCTCTTCCTTTCC
PR032f_WP004954459	(SmTS10)	GTGACCGACGCCGACAGCCA
PR032r_WP004954459	(SmTS10)	TCAGCCTTGCCCCTGCCACG
PR031f_WP004954459	(SmTS10)	GGCAGCCATATGGCTAGCATGACTGGTGGAGTGACCGACGCCGACAGCCA
PR031r_WP004954459	(SmTS10)	TCTCAGTGGTGGTGGTGGTGGTGCTCGAGTTCAGCCTTGCCCCTGCCACG

Enzyme purification

The collected cell pellets (from 200 mL culture) were suspended in binding buffer (5 mL; 20 mM Na_2HPO_4, 500 mM NaCl, 20 mM imidazole, 1 mM $MgCl_2$, pH 7.4, 4 °C) and lysed by ultra-sonification (5 x 1 min). The lysate was centrifuged (14000 x g, 7 min) to remove the cell debris. The supernatant was loaded onto a Ni^{2+}-NTA affinity chromatography column (Super Ni-NTA, Generon, Slough, UK), followed by washing with binding buffer (2 x 2 mL) and elution of the target portein with elution buffer (2 mL; 20 mM Na_2HPO_4, 500 mM NaCl, 500 mM imidazole, 1 mM $MgCl_2$, pH 7.4, 4 °C). The protein purity and concentration in eluted fractions were analyzed by SDS-PAGE and Bradford assay.[18] Typical protein concentrations were 0.5 mg/mL for SmTS1 and 1.2 mg/mL for GFPPS.

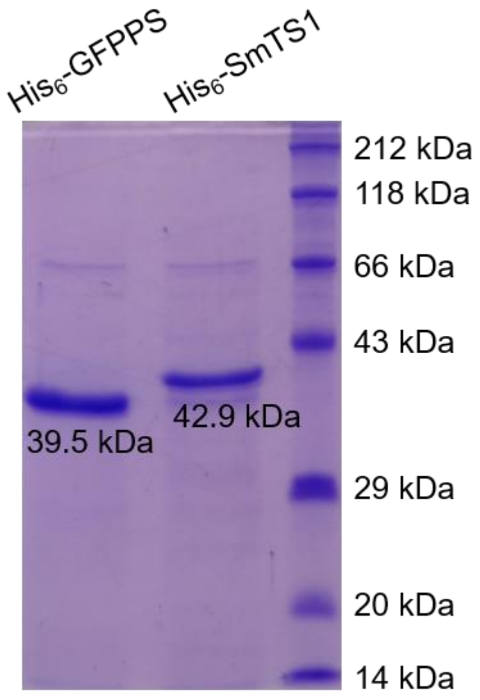

Figure S5. SDS-PAGE of purified His-tagged SmTS1 and GFPPS.

Incubation experiments

For incubations with GFPP, purified SmTS1 (0.2 mL; ca. 0.5 mg/mL), a solution of GFPP (0.2 mL; 1.0 mg/mL in 25 mM NH_4HCO_3) and incubation buffer (0.5 mL; 20 mM Na_2HPO_4, 4 mM $MgCl_2$, 10% glycerol, pH 7.4) were mixed and incubated at 28 °C overnight. The enzyme products were extracted with hexane (0.2 mL), and the obtained extract was dried with $MgSO_4$ and analyzed by GC/MS.

For incubations with GGPP, FPP, GPP or DMAPP, a solution of each substrate (1 mL; 1.5 mg/mL in 25 mM NH_4HCO_3), purified GFPPS (2 mL; ca. 1.2 mg/mL), SmTS1 (2 mL; ca. 0.5 mg/mL) and a solution of IPP (1 mL; 2.0 mg/mL in 25 mM NH_4HCO_3) were mixed with incubation buffer (5 mL) and incubated at 28 °C overnight. The products were extracted with hexane (0.6 mL), and the obtained extract was dried with $MgSO_4$ and analyzed by GC/MS.

For preparative scale incubation, solutions of GFPPS (45 mL) and SmTS1 (45 mL), each obtained from 4 L *E. coli* culture, were used to set up 30 small scale reactions. The small scale reaction system was: GFPPS (1.5 mL), SmTS1 (1.5 mL), FPP (0.8 mL; 1.5 mg/mL in 25 mM NH_4HCO_3), IPP (0.8 mL; 2.0 mg/mL in 25 mM NH_4HCO_3) and incubation buffer (5 mL). After incubation at 28 °C overnight, the reaction mixtures of all small scale reactions were combined. The obtained products were extracted with hexane (3 x 50 mL) and concentrated under reduced pressure.

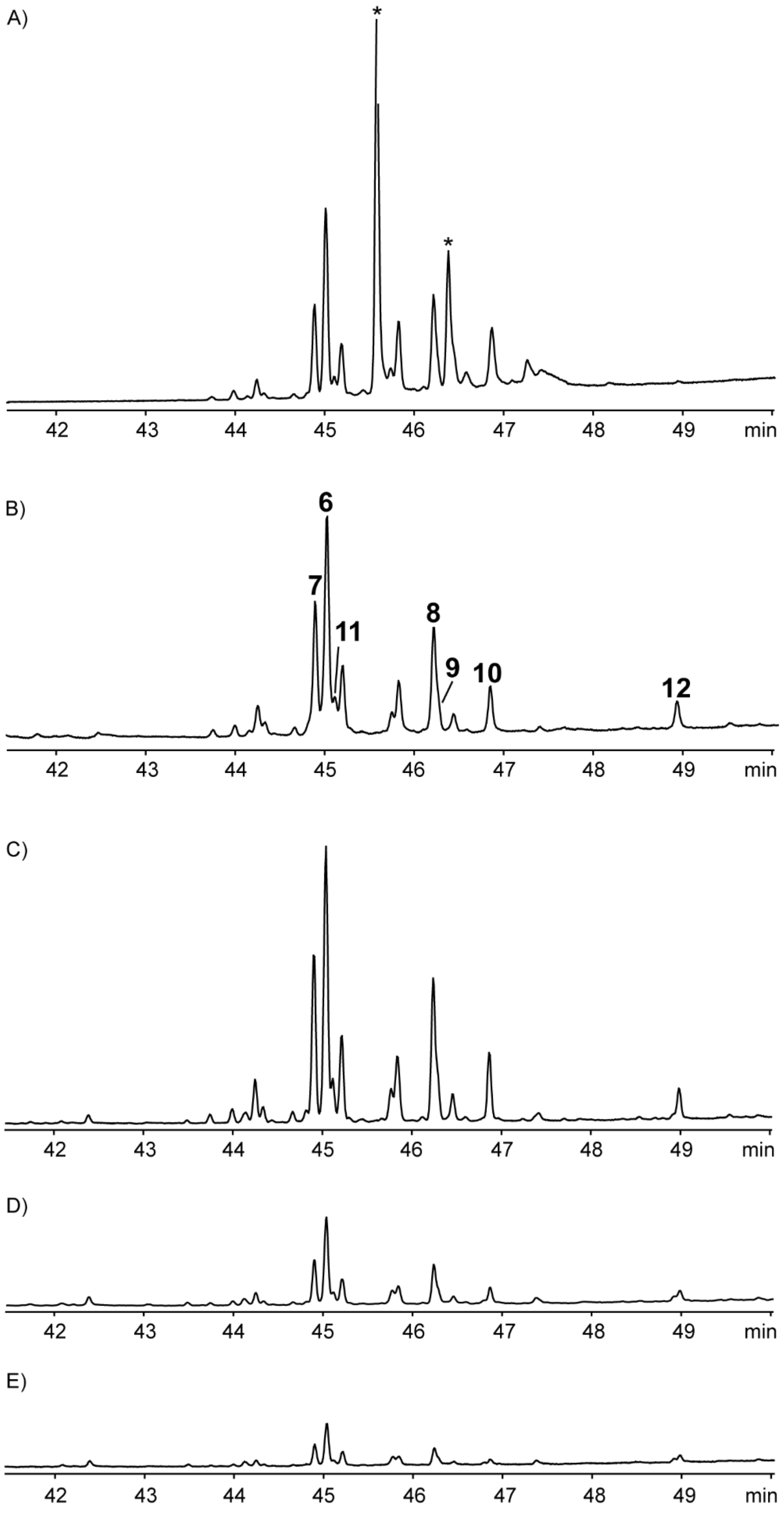

Figure S6. Incubation experiments with SmTS1. Products obtained from A) GFPP, B) GGPP with IPP and GFPPS, C) FPP with IPP and GFPPS, D) GPP with IPP and GFPPS, and E) DMAPP with IPP and GFPPS. The asterisks indicate non-enzymatic degradation products from GFPP. For comparison, the Y-axis of all five chromatograms was set to the same absolute scaling.

Compound purification
The crude products obtained from the preparative scale enzyme incubation were purified via silica gel chromatography to afford pure **10** (0.4 mg) and **12** (0.7 mg). Compound **6** (0.9 mg) was obtained via HPLC purification. HPLC purifications were performed on a Smartline series HPLC system (Knauer, Berlin, Germany), equipped with a UV/Vis-Detector S-2550 (190–1000 nm) and a Knauer Eurospher II 100-5 C18P column (5 μm; 8 × 250 mm). Elution was performed with acetonitrile at 6 mL/min (86 bar). The UV/Vis absorption was monitored at 205 nm.
The other compounds were purified via repeated preparative TLC using $AgNO_3$ coated TLC plates. The $AgNO_3$ coated TLC plates were obtained by treatment of commercial TLC plates (TLC Silica gel 60 F_{254}, 20 x 20 cm, Merck, Darmstadt, Germany) with a solution of $AgNO_3$ in methanol (5 g/100 mL) for 10 min, followed by drying at 65 °C for 40 min. After TLC separation with a mixture solvent of cyclohexane and ethyl acetate, a small stripe of the TLC plate was cut off and stained with molybdophosphoric acid in EtOH (10 g/100 mL). The silica of regions containing the target compounds was scratched off and extracted with diethyl ether. After evaporation of the solvent, the pure compounds **7** (1.5 mg), **8** (1.5 mg), **9** (0.5 mg), **11** (0.8 mg) were obtained.

NMR spectroscopy
NMR spectra were recorded on a Bruker (Billerica, MA, USA) Avance I (300 MHz), Avance I (400 MHz), Avance I (500 MHz), Avance III HD Prodigy (500 MHz) or an Avance III HD Cryo (700 MHz) NMR spectrometer. Spectra were measured in C_6D_6 and referenced against solvent signals (^{1}H-NMR, residual proton signal: δ = 7.16; ^{13}C-NMR: δ = 128.06).[19]

GC/MS-QTOF
GC/MS-QTOF analyses were performed on a 7890B GC connected to a 7200 accurate-mass Q-TOF detector (Agilent) equipped with a HP5-MS fused silica capillary column (30 m, 0.25 mm i. d., 0.50 μm film). MS parameters were 1) inlet pressure: 83.2 kPa, He at 24.6 mL min^{-1}, 2) transfer line: 250 °C, 3) electron energy 70 eV. GC parameters were 1) temperature program: 5 min at 50 °C increasing at 5 °C min^{-1} to 320 °C, 2) injection volume: 1 μL, 3) split ratio: 50:1, 60 s valve time, and 4) carrier gas: He at 1 mL min^{-1}.

IR spectroscopy and optical rotations
IR spectra were recorded on an ALPHA II FTIR Spectrometer (Bruker Optics, MA, USA), and the scan range was set to 500 to 4000 cm^{-1}. Optical rotations were recorded on a MCP 150 Modular Circular Polarimeter (Anton Paar GmbH, Graz, Austria).

(–)-Sestermobaraene A ((3*S*,3a*R*,5a*R*,6*R*,7*R*,11a*R*,12*S*,*Z*)-3-isopropyl-5a,9a,11a,12-tetramethyl-2,3,3a,5a,6,7,8,9,9a,10,11,11a-dodecahydro-1*H*-5,7,6-(epipropane[1,1,3]triyl)-benzo[*a*]cyclopenta[*e*][8]annulene, **6**). TLC (pentane): R_f = 0.95. Optical rotation: $[\alpha]_D^{20}$ = –4.4 (*c* 0.09, CH_2Cl_2). HRMS (ToF): *m*/*z* = 340.3122 (calc. for $[C_{25}H_{40}]^{+\cdot}$ 340.3124). GC (HP-5MS): *I* = 2358. MS (EI, 70 eV): Figure S2. IR (diamond ATR): $\tilde{\nu}$ / cm^{-1} = 2591 (s), 2924 (s), 2857 (m), 1736 (m), 1718 (m), 1458 (m), 1376 (m), 1366 (m), 1228 (w), 1206(w), 1094 (w), 1028 (w), 800 (w), 543 (w). NMR data are given in Table S3 and Figures S7–S14.

(–)-Sestermobaraene B ((3*R*,3a*R*,6*S*,6a*R*,7a*R*,10*S*,10a*S*,11a*S*,11b*S*)-10-isopropyl-6,7a,11b-trimethyl-12-methylenehexadecahydro-3,11a-methanoindeno[5,6-*e*]azulene, **7**). TLC ($AgNO_3$ coated, cyclohexane): R_f = 0.45. Optical rotation: $[\alpha]_D^{20}$ = –21.3 (*c* 0.15, DCM). HRMS (ToF): *m*/*z* = 340.3130 (calc. for $[C_{25}H_{40}]^{+\cdot}$ 340.3124). GC (HP-5MS): *I* = 2350. MS (EI, 70 eV): Figure S2. IR (diamond ATR): $\tilde{\nu}$ / cm^{-1} = 2948 (s), 2924 (s), 2895 (m), 2869 (m), 1645 (w), 1460 (m),

1375 (m), 1260 (m), 1086 (m), 1016 (m), 876 (m), 800 (m). NMR data are given in Table S4 and Figures S15–S22.

(–)-Sestermobaraene C ((3*S*,3a*R*,6a*R*,7a*R*,10*S*,10a*S*,11a*R*,11b*S*)-10-isopropyl-7a,11b,12-trimethyl-6-methylenehexadecahydro-3,11a-methanoindeno[5,6-*e*]azulene, **8**). TLC [$AgNO_3$ coated, cyclohexane/ethyl acetate (40/1)]: R_f = 0.38. Optical rotation: $[\alpha]_D^{20}$ = –14.7 (*c* 0.15, CH_2Cl_2). HRMS (ToF): *m*/*z* = 340.3129 (calc. for $[C_{25}H_{40}]^{+\cdot}$ 340.3124). GC (HP-5MS): *I* = 2432. MS (EI, 70 eV): Figure S2. IR (diamond ATR): $\tilde{\nu}$ / cm^{-1} = 2946 (s), 2925 (s), 2872 (m), 1628 (w), 1466 (m), 1385 (w), 1373 (w), 1260 (m), 1092 (m), 1076 (m), 1019 (m), 888 (m), 801 (m). NMR data are given in Table S5 and Figures S23–S30.

(–)-Sestermobaraene D ((3*S*,3a*R*,3a^1*S*,6a*R*,7a*R*,10*S*,10a*S*,11a*S*)-10-isopropyl-3a^1,7a,12-trimethyl-6-methylenetetradecahydro-1*H*,4*H*-3,11a-methanonaphtho[1,8-*fg*]azulene, **9**). TLC ($AgNO_3$ coated, cyclohexane): R_f = 0.27. Optical rotation: $[\alpha]_D^{20}$ = –42 (*c* 0.05, CH_2Cl_2). HRMS (ToF): *m*/*z* = 340.3128 (calc. for $[C_{25}H_{40}]^{+\cdot}$ 340.3124). GC (HP-5MS): *I* = 2435. MS (EI, 70 eV): Figure S2. IR (diamond ATR): $\tilde{\nu}$ / cm^{-1} = 2956 (m), 2923 (s), 2872 (m), 2852 (m), 1738 (w), 1659 (w), 1633 (m), 1466 (m), 1435 (w), 1377 (m), 1260 (w), 1090 (w), 1019 (m), 879 (m), 798 (m). NMR data are given in Table S6 and Figures S31–S38.

(–)-Sestermobaraene E ((1*S*,3a*R*,9*E*,13*E*,15*E*,16a*S*)-1-isopropyl-3a,10,14-trimethyl-6-methylene-1,2,3,3a,4,5,6,7,8,11,12,16a-dodecahydrocyclopenta[15]annulene **10**). TLC (pentane): R_f = 0.42. Optical rotation: $[\alpha]_D^{20}$ = –37.5 (*c* 0.04, CH_2Cl_2). HRMS (ToF): *m*/*z* = 340.3124 (calc. for $[C_{25}H_{40}]^{+\cdot}$ 340.3124). GC (HP-5MS): *I* = 2470. MS (EI, 70 eV): Figure S2. IR (diamond ATR): $\tilde{\nu}$ / cm^{-1} = 2953 (m), 2923 (s), 2854 (m), 1738 (w), 1644 (w), 1438 (m), 1377 (m), 1367 (m), 1260 (m), 1217 (w), 1064 (m), 1020 (m), 967 (m), 884 (m), 800(m). NMR data are given in Table S7 and Figures S39–S46.

(+)-Sestermobaraene F ((3*S*,3a*S*,6a*S*,7a*R*,10*S*,10a*S*,11a*S*,11b*S*,11c*S*)-10-isopropyl-3,7a,11b-trimethyl-6-methylenehexadecahydro-1*H*-cyclopenta[2',3']cyclopropa[1',2':3,4]cyclohepta[1,2-*f*]indene **11**). TLC [$AgNO_3$ coated, cyclohexane/ethyl acetate (40/1)]: R_f = 0.42. Optical rotation: $[\alpha]_D^{20}$ = +15 (*c* 0.08, CH_2Cl_2). HRMS (ToF): *m*/*z* = 340.3123 (calc. for $[C_{25}H_{40}]^{+\cdot}$ 340.3124). GC (HP-5MS): *I* = 2362. MS (EI, 70 eV): Figure S2. IR (diamond ATR): $\tilde{\nu}$ / cm^{-1} = 2948 (s), 2925 (s), 2870 (m), 1738 (w), 1658 (w), 1465 (m), 1376 (m), 1260 (w), 1229 (w), 1217 (w), 1093 (m), 1019 (m), 888 (m), 803 (m). NMR data are given in Table S8 and Figures S47–S54.

(–)-Sestermobaraol ((3*S*,3a*S*,5*S*,5a*S*,6*R*,7*R*,9a*S*,10*S*,11a*R*,12*S*)-3-isopropyl-5a,10,11a,12-tetramethyltetradecahydro-9a*H*-5,7,6-(epipropane[1,1,3]triyl)benzo[*a*]cyclopenta[*e*][8]-annulen-9a-ol **12**). TLC [cyclohexane/ethyl acetate (5/1)]: R_f = 0.55. Optical rotation: $[\alpha]_D^{20}$ = –35.7 (*c* 0.07, CH_2Cl_2). HRMS (ToF): *m*/*z* = 358.3234 (calc. for $[C_{25}H_{42}O]^{+\cdot}$ 358.3230). GC (HP-5MS): *I* = 2608. MS (EI, 70 eV): Figure S2. IR (diamond ATR): $\tilde{\nu}$ / cm^{-1} = 3274 (w), 2953 (s), 2924 (s), 2855 (m), 1735 (w), 1672 (w), 1652 (w), 1463 (m), 1375 (m), 1345 (m), 1261 (m), 1092 (m), 1016 (m), 798 (m), 544 (m). NMR data are given in Table S9 and Figures S55–S62.

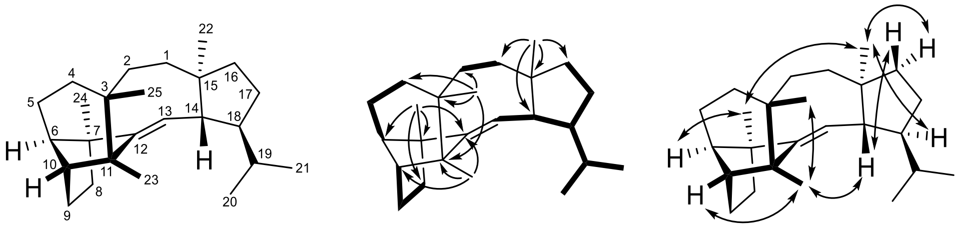

Figure S7. Structure elucidation of **6**. Bold: ^{1}H,^{1}H-COSY correlations, single-headed arrows: key HMBC correlations, and double-headed arrows: key NOESY correlations.

Table S3. NMR data of sestermobaraene A (**6**) in C_6D_6 recorded at 298 K.

C[a]		^{13}C[b]	^{1}H[b]
1	CH_2	35.39	1.47 (m, 2H)
2	CH_2	34.69	1.47 (m, 1H, H_β)
			1.39 (m, 1H, H_α)
3	C_q	43.28	–
4	CH_2	34.17	1.53 (m, 1H, H_α)
			1.02 (m, 1H, H_β)
5	CH_2	20.47	1.64 (m, 1H, H_β)
			1.47 (m, 1H, H_α)
6	CH	49.68	1.41 (m, 1H)
7	C_q	52.31	–
8	CH_2	39.91	1.47 (m, 1H, H_α)[c]
			1.37 (m, 1H, H_β)[c]
9	CH_2	23.19	1.59 (m, 1H, H_β)[c]
			1.38 (m, 1H, H_α)[c]
10	CH	49.03	1.90 (d, *J* = 4.0, 1H)
11	C_q	50.63	–
12	C_q	156.19	–
13	CH	120.18	5.15 (d, *J* = 9.3, 1H)
14	CH	48.38	2.28 (dd, *J* = 10.9, 9.6, 1H)
15	C_q	49.09	–
16	CH_2	39.65	1.43 (m, 1H, H_β)
			1.30 (m, 1H, H_α)
17	CH_2	25.88	1.80 (m, 1H, H_α)
			1.35 (m, 1H, H_β)
18	CH	50.30	1.66 (m, 1H)
19	CH	33.09	1.56 (m, 1H)
20	CH_3	22.24	0.98 (d, *J* = 6.9, 3H)
21	CH_3	20.65	0.92 (d, *J* = 6.6, 3H)
22	CH_3	21.33	0.87 (s, 3H)
23	CH_3	20.12	1.30 (s, 3H)
24	CH_3	17.04	1.14 (s, 3H)
25	CH_3	25.47	0.94 (s, 3H)

[a] Carbon numbering as shown in Figure S7. [b] Chemical shifts δ in ppm, multiplicity: s = singlet, d = doublet, m = multiplet, coupling constants *J* are given in Hertz. [c] For assignment of H_α and H_β cf. Figure S101.

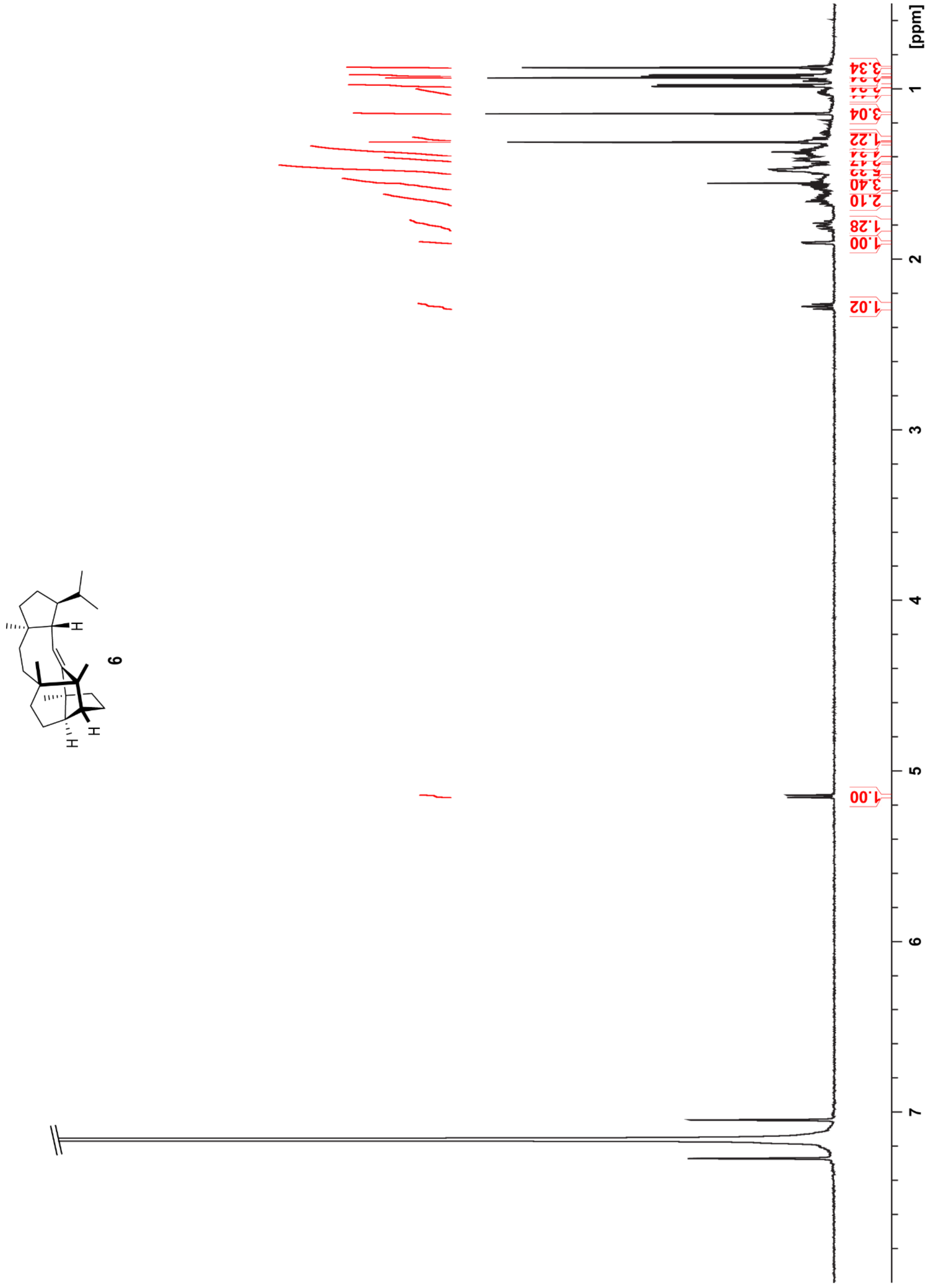

Figure S8. ^{1}H-NMR spectrum (700 MHz, C_6D_6) of **6**.

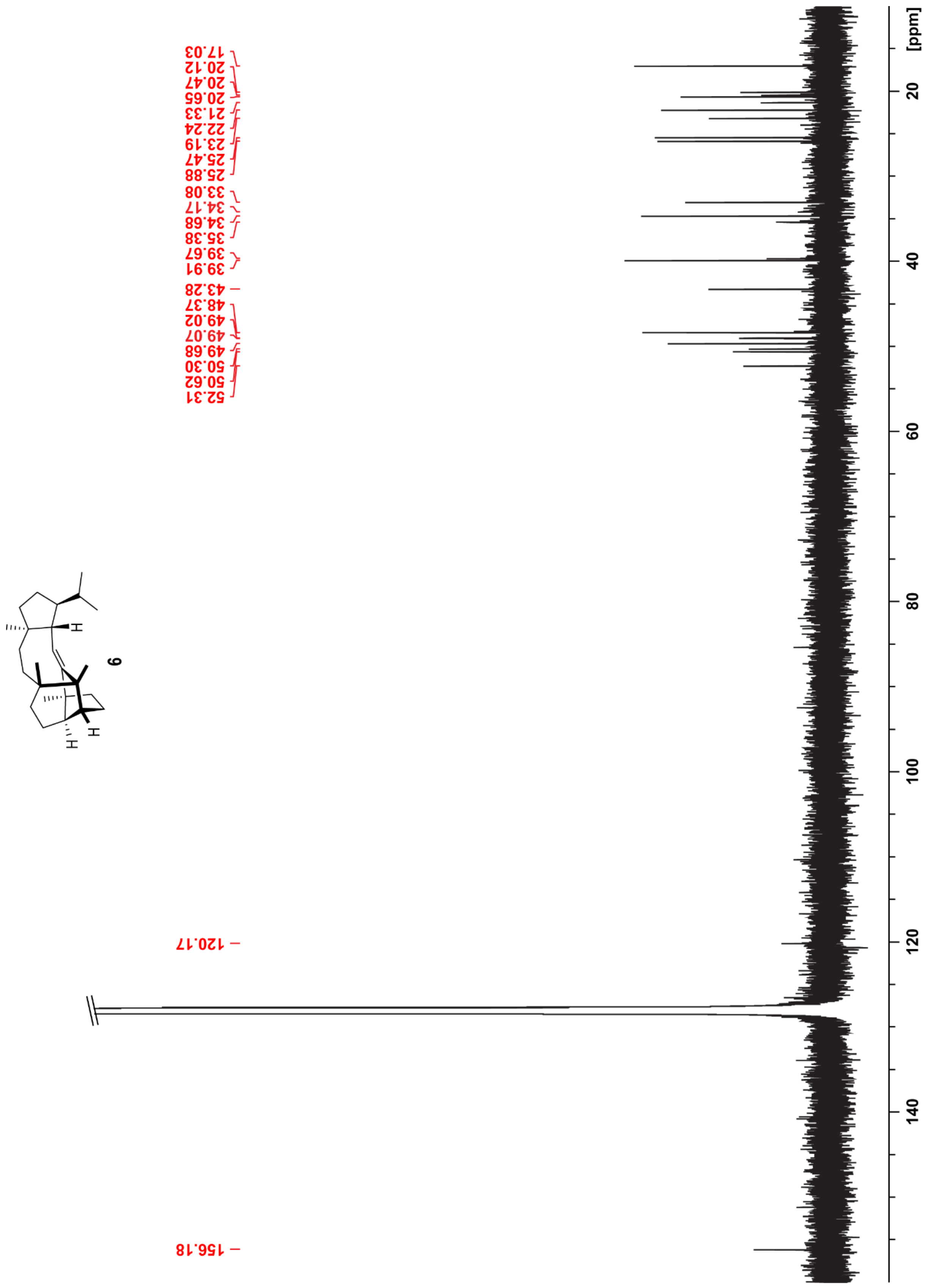

Figure S9. ^{13}C-NMR spectrum (176 MHz, C_6D_6) of **6**.

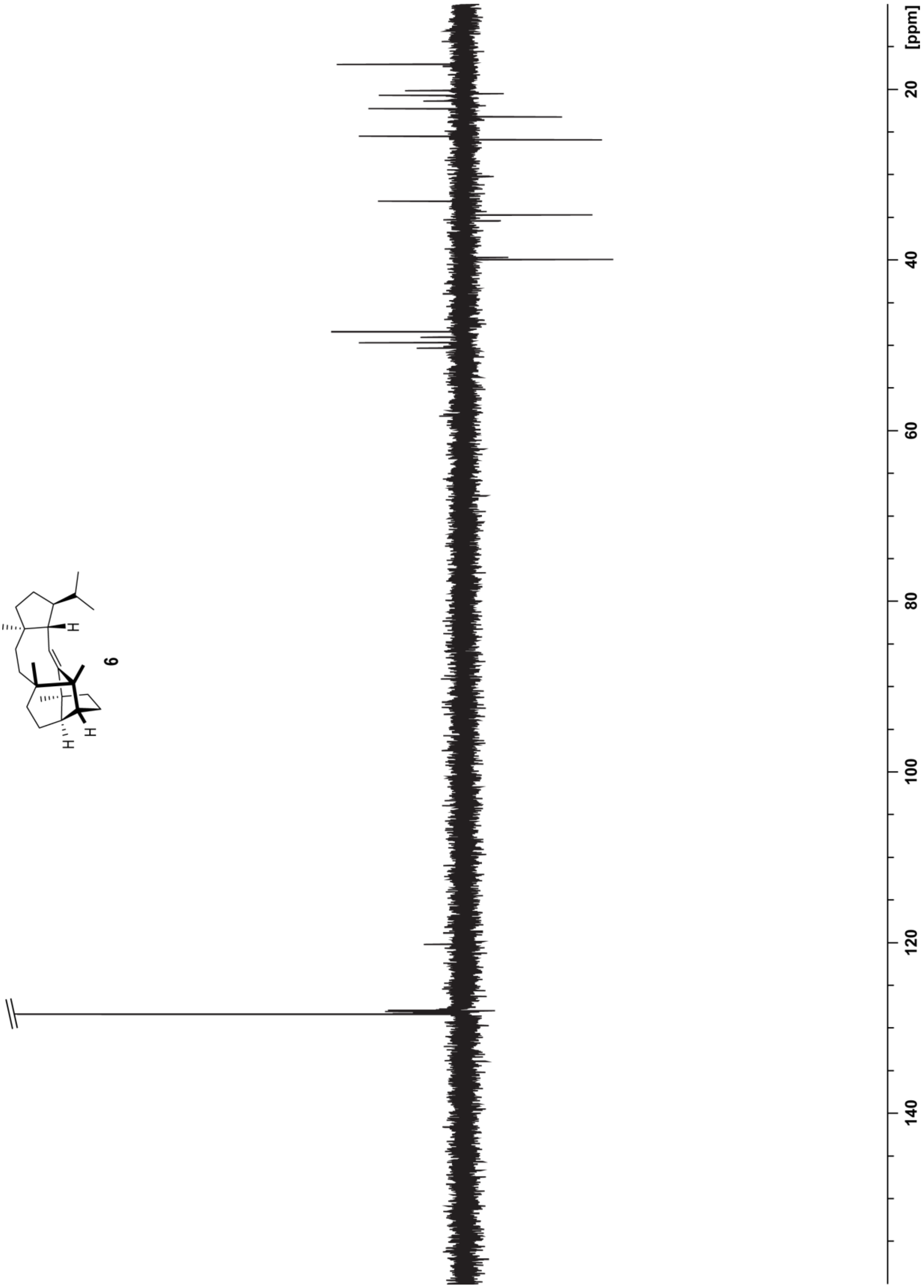

Figure S10. ^{13}C-DEPT135 spectrum (176 MHz, C_6D_6) of **6**.

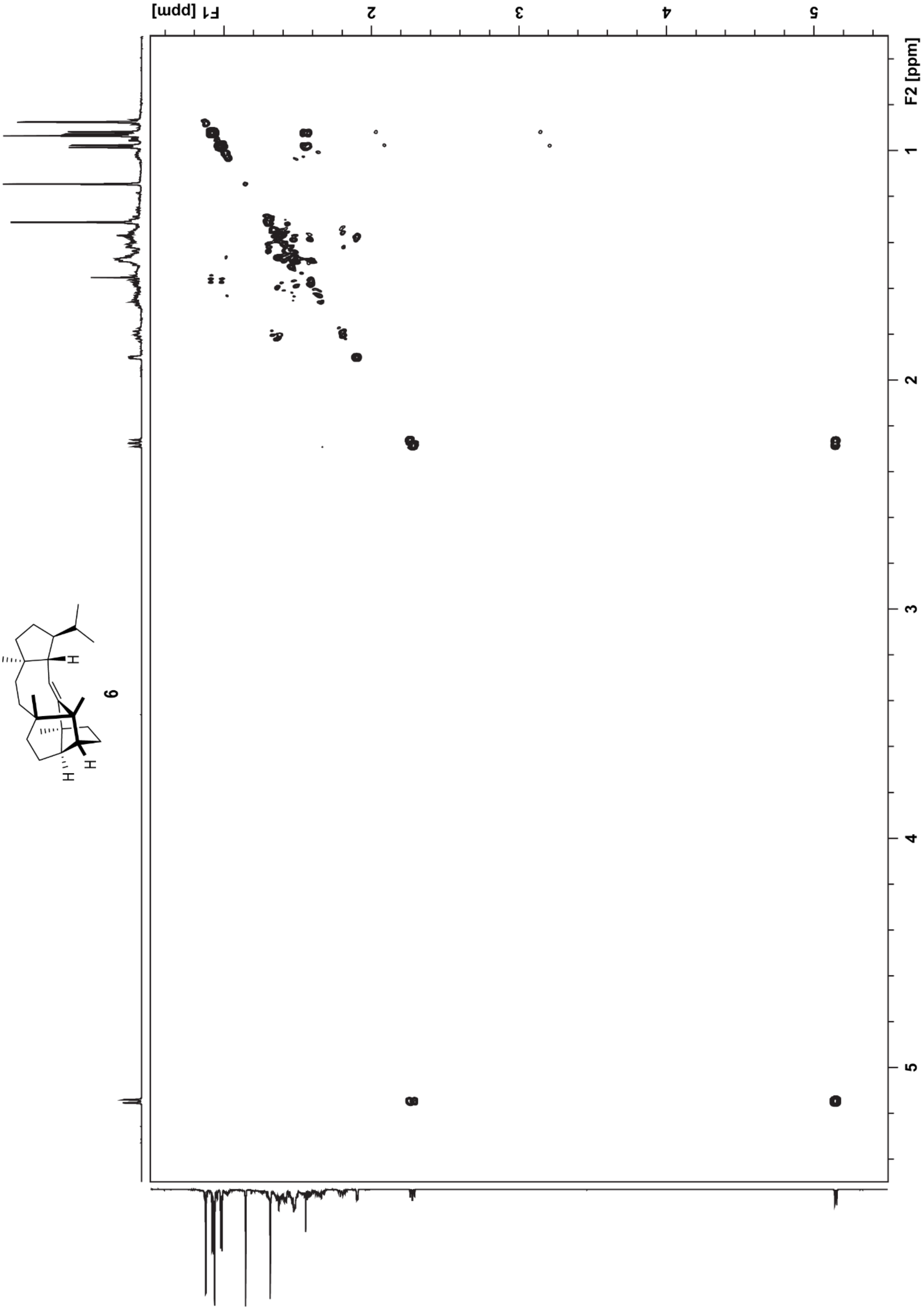

Figure S11. $^{1}H,^{1}H$-COSY spectrum (C_6D_6) of **6**.

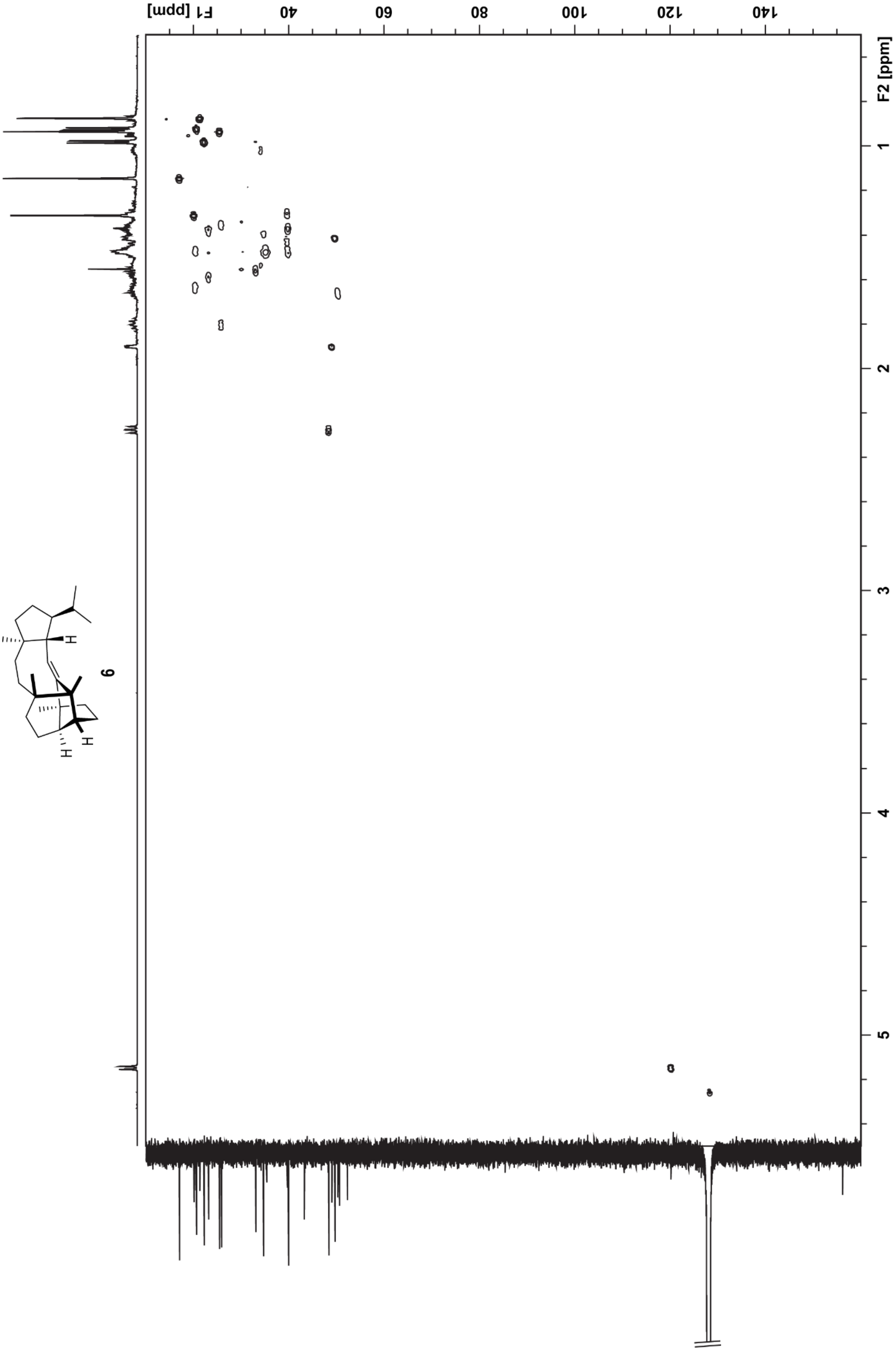

Figure S12. HSQC spectrum (C_6D_6) of **6**.

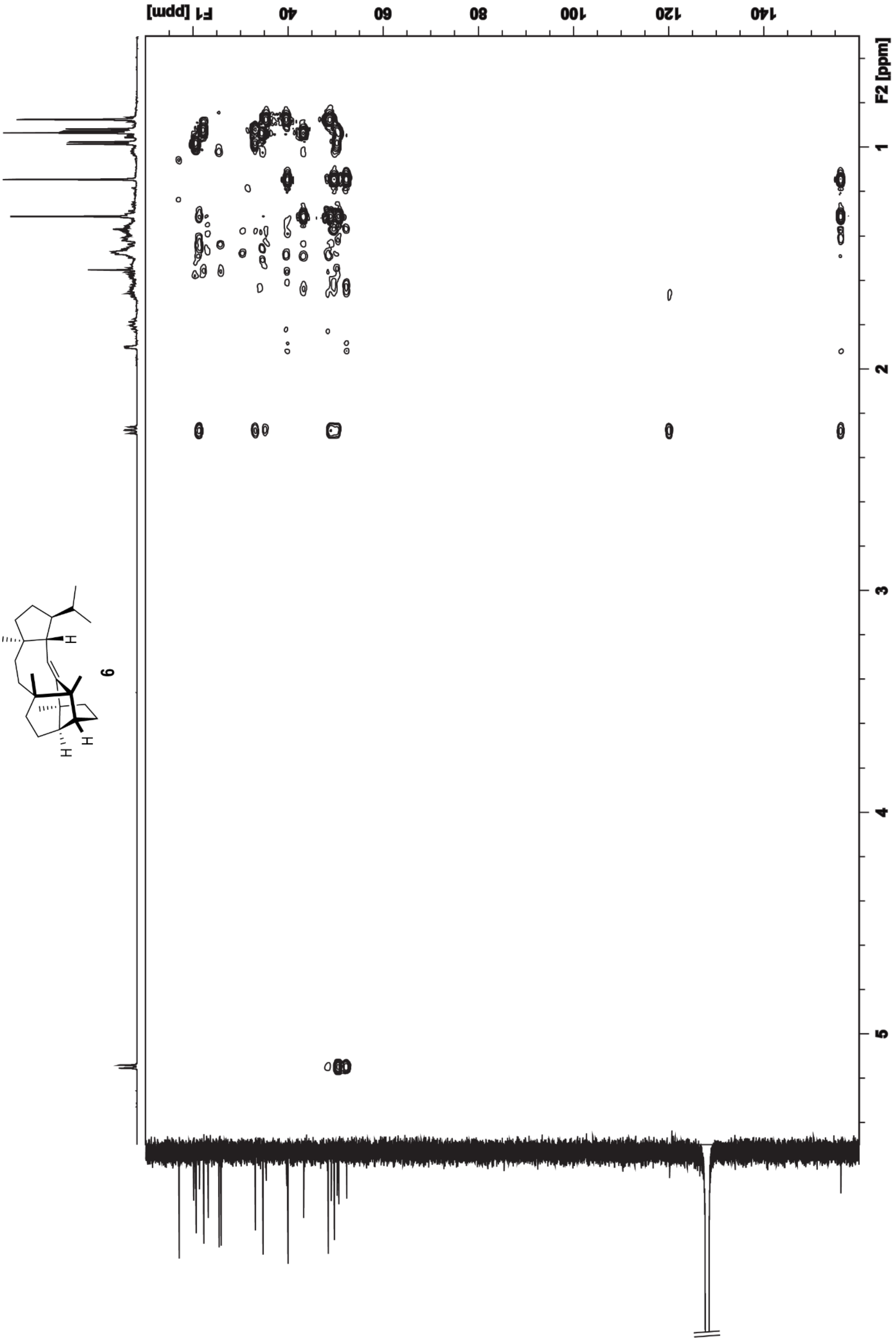

Figure S13. HMBC spectrum (C_6D_6) of **6**.

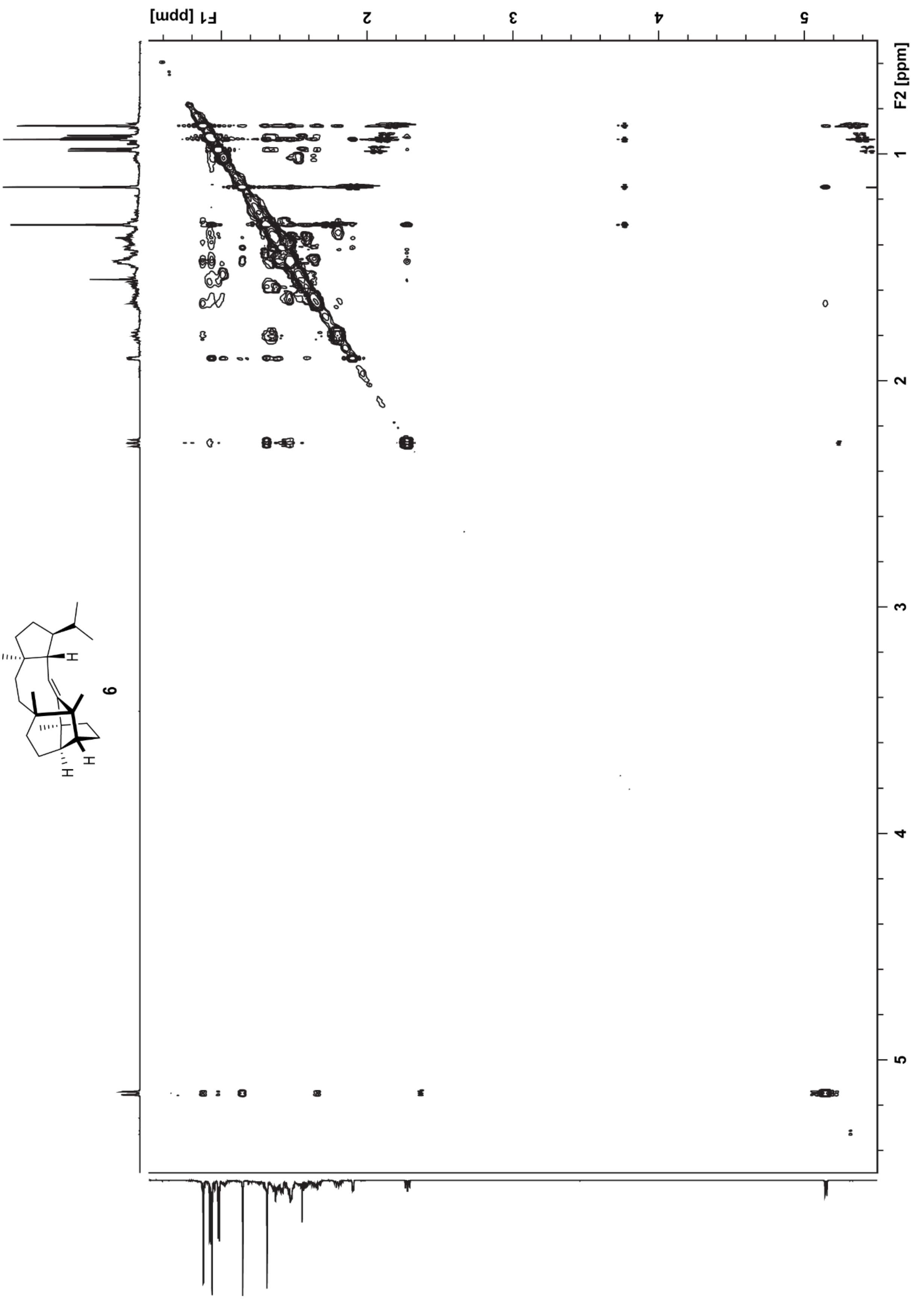

Figure S14. NOESY spectrum (C_6D_6) of **6**.

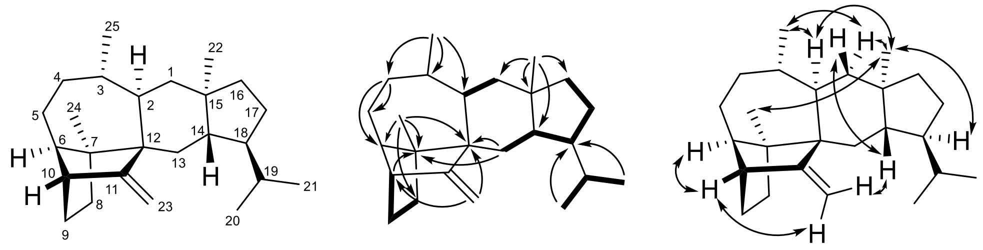

Figure S15. Structure elucidation of **7**. Bold: $^{1}H,^{1}H$-COSY correlations, single-headed arrows: key HMBC correlations, and double-headed arrows: key NOESY correlations.

Table S4. NMR data of sestermobaraene B (**7**) in C_6D_6 recorded at 298 K.

C[a]		13C[b]	1H[b]
1	CH_2	45.93	1.84 (m, 1H, H_α)
			1.30 (m, 1H, H_β)
2	CH	42.54	1.51 (m, 1H)
3	CH	38.37	1.49 (m, 1H)
4	CH_2	32.54	1.87 (m, 1H, H_β)
			1.45 (m, 1H, H_α)
5	CH_2	25.42	1.84 (m, 1H, H_α)
			1.53 (m, 1H, H_β)
6	CH	51.91	1.53 (m, 1H)
7	C_q	54.43	–
8	CH_2	36.73	1.93 (ddd, *J* = 12.5, 9.1, 3.0, 1H, H_β)[c]
			1.14 (m, 1H, H_α)[c]
9	CH_2	29.80	1.69 (m, 1H, H_α)[c]
			1.32 (m, 1H, H_β)[c]
10	CH	56.26	2.33 (dd, *J* = 5.0, 1.1, 1H)
11	C_q	165.27	–
12	C_q	49.46	–
13	CH_2	35.13	1.54 (m, 1H, H_β)
			1.23 (t, *J* = 13.0, 1H, H_α)
14	CH	46.08	1.59 (m, 1H)
15	C_q	41.38	–
16	CH_2	39.91	1.49 (m, 1H, H_α)
			1.18 (m, 1H, H_β)
17	CH_2	23.85	1.72 (m, 1H, H_α)
			1.42 (m, 1H, H_β)
18	CH	46.83	1.50 (m, 1H)
19	CH	29.64	1.73 (m, 1H)
20	CH_3	22.21	0.97 (d, *J* = 6.9, 3H)
21	CH_2	17.99	0.89 (d, *J* = 6.8, 3H)
22	CH_3	17.79	0.75 (s, 3H)
23	CH_2	103.51	4.98 (d, *J* = 1.1, 1H, H_Z)[d]
			4.90 (d, J = 1.0, 1H, H_E)[d]
24	CH_3	16.98	1.08 (s, 3H)
25	CH_3	23.32	1.00 (d, *J* = 6.4, 3H)

[a] Carbon numbering as shown in Figure S15. [b] Chemical shifts δ in ppm, multiplicity: s = singlet, d = doublet, t = triplet, m = multiplet, coupling constants *J* are given in Hertz. [c] For assignment of H_α and H_β cf. Figure S102. [d] Assignment according to CIP priority rules.

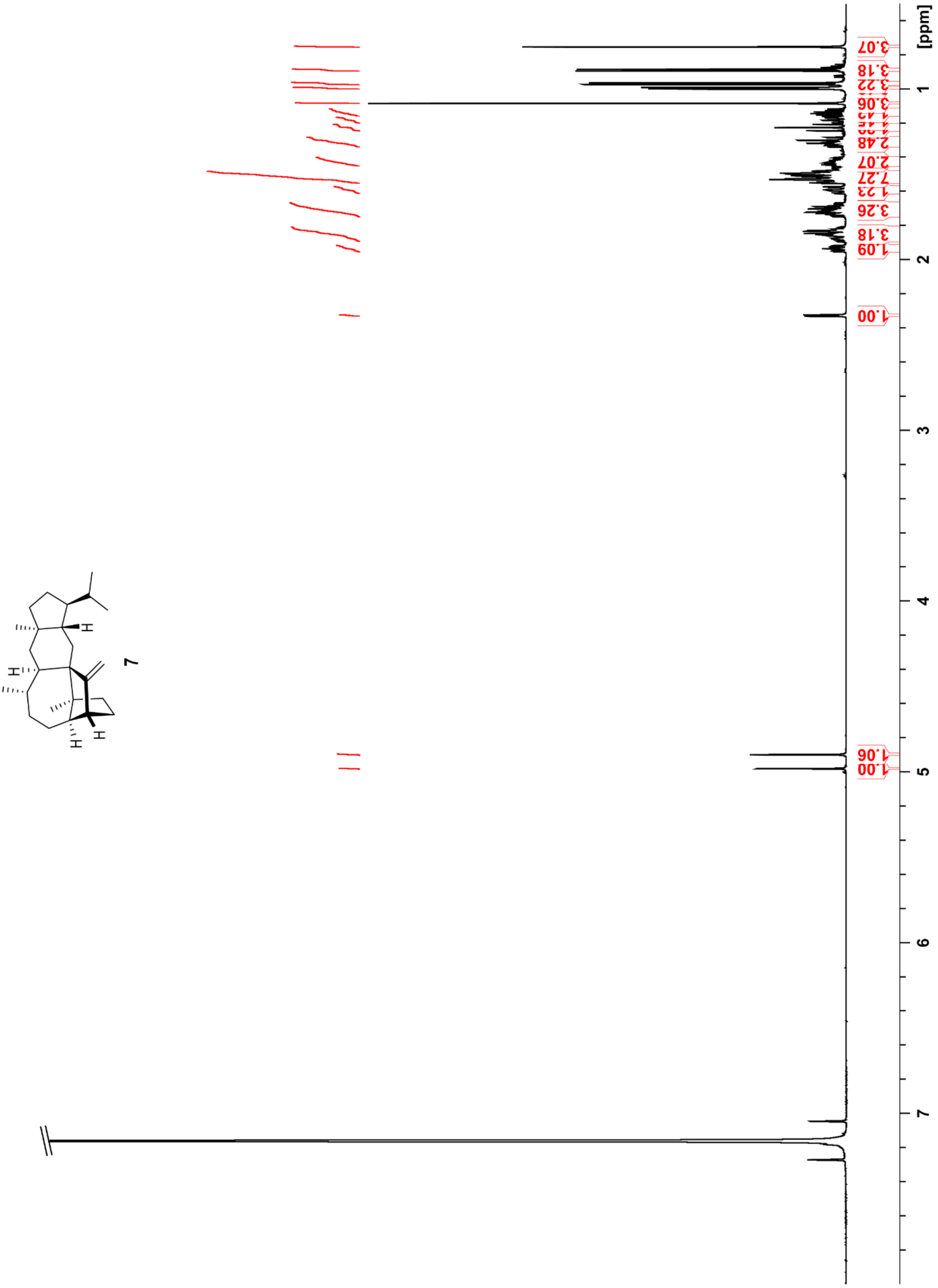

Figure S16. ^{1}H-NMR spectrum (700 MHz, C_6D_6) of **7**.

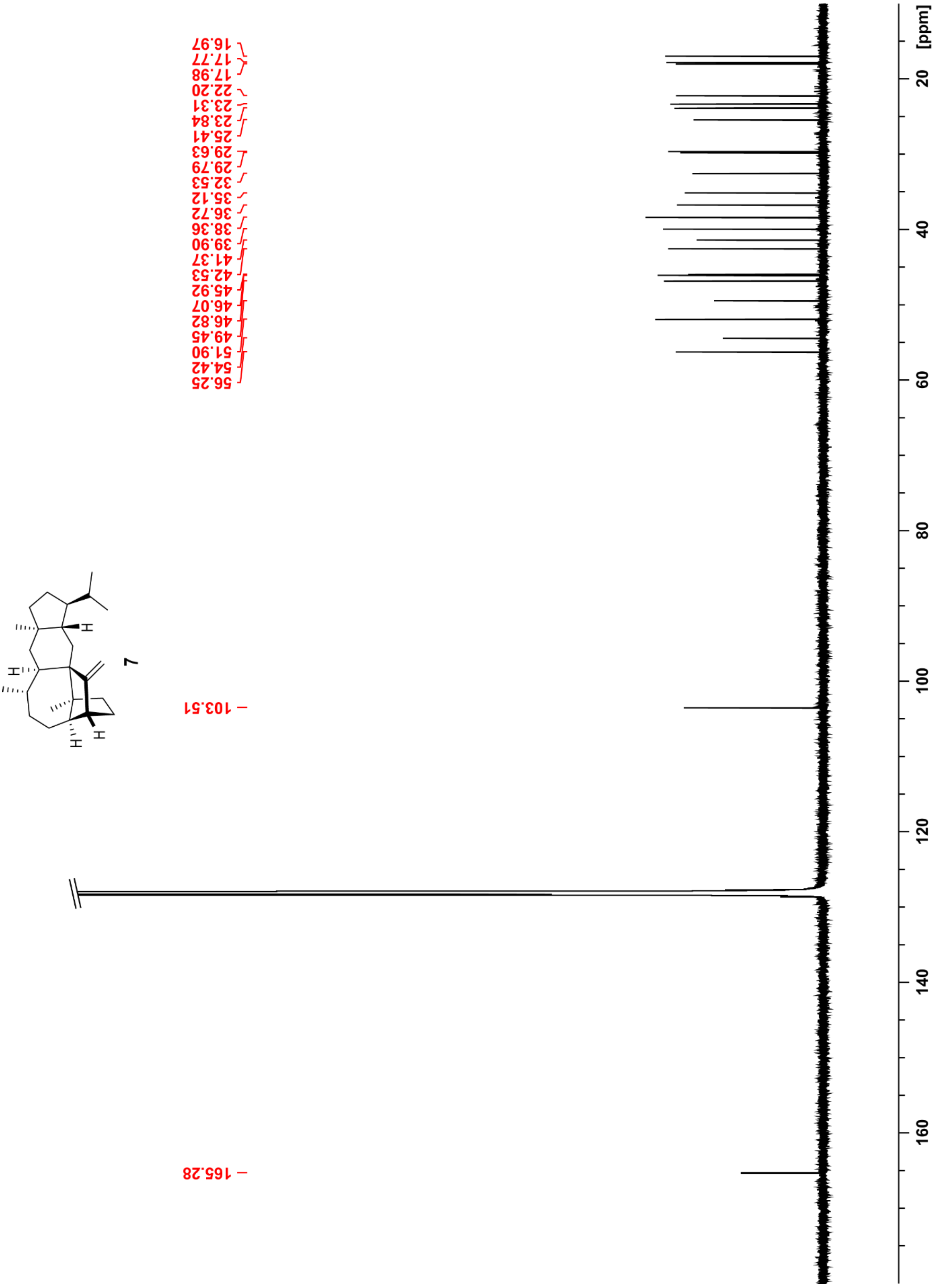

Figure S17. ^{13}C-NMR spectrum (176 MHz, C_6D_6) of **7**.

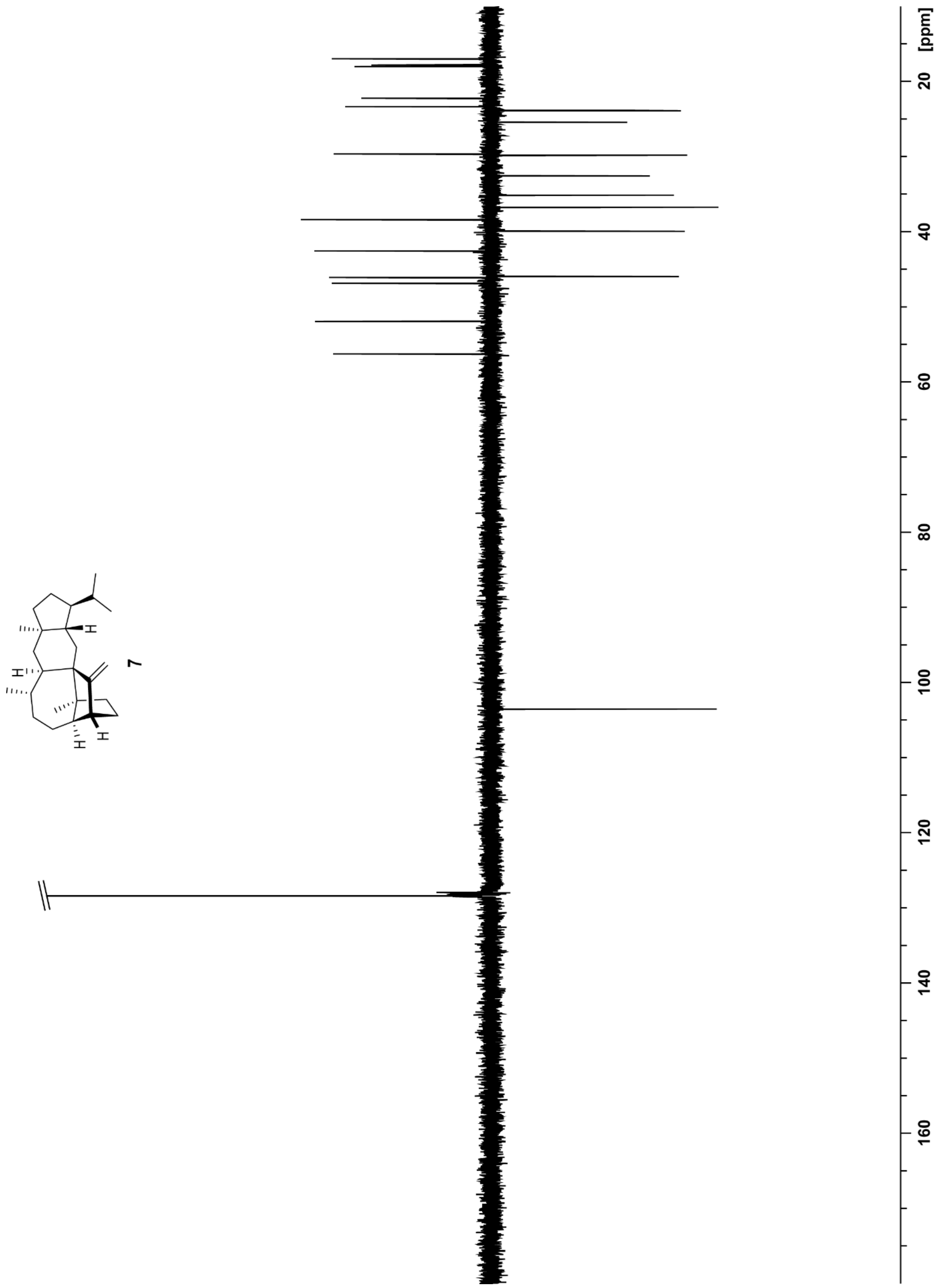

Figure S18. ^{13}C-DEPT135 spectrum (176 MHz, C_6D_6) of **7**.

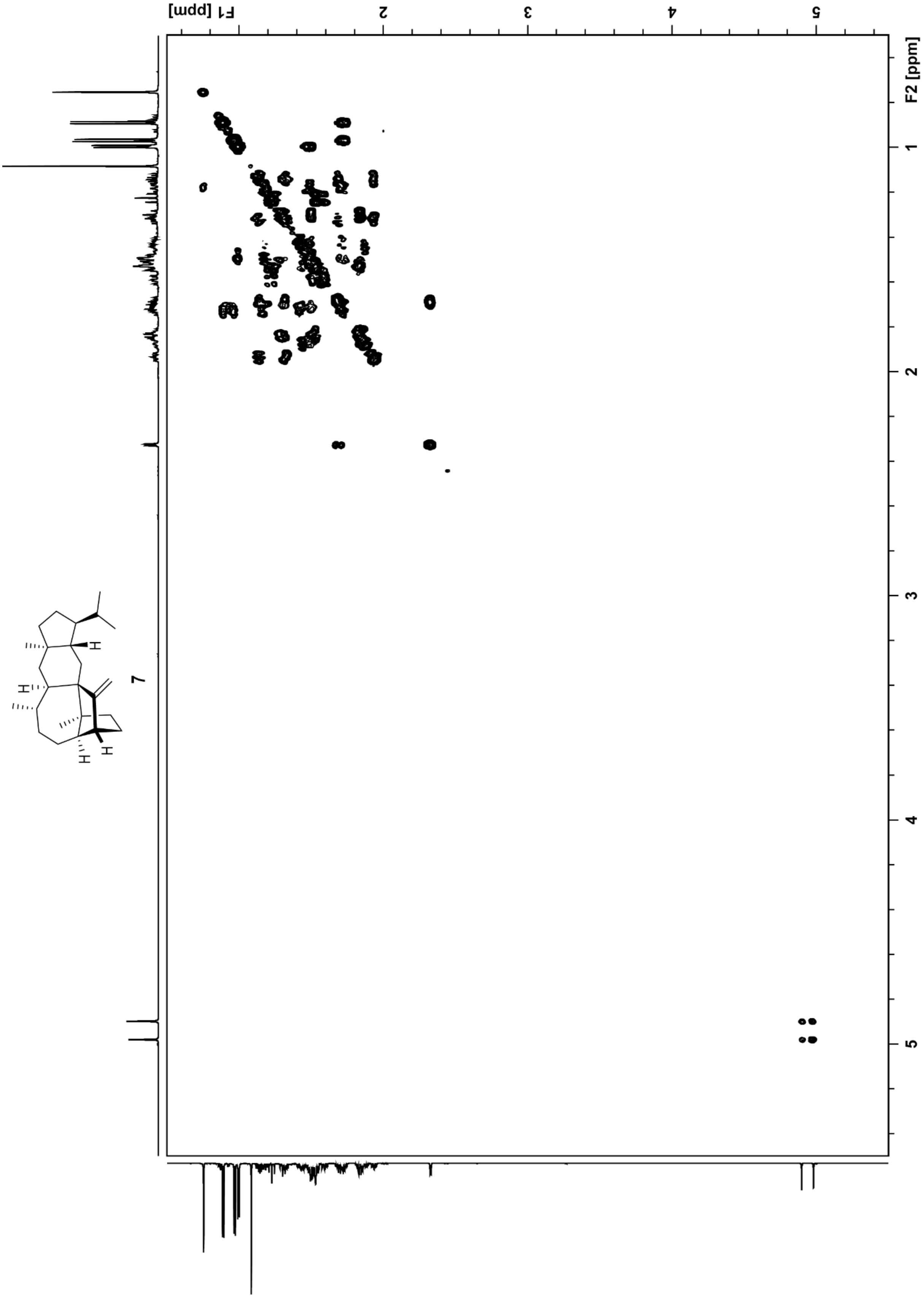

Figure S19. $^{1}H,^{1}H$-COSY spectrum (C_6D_6) of **7**.

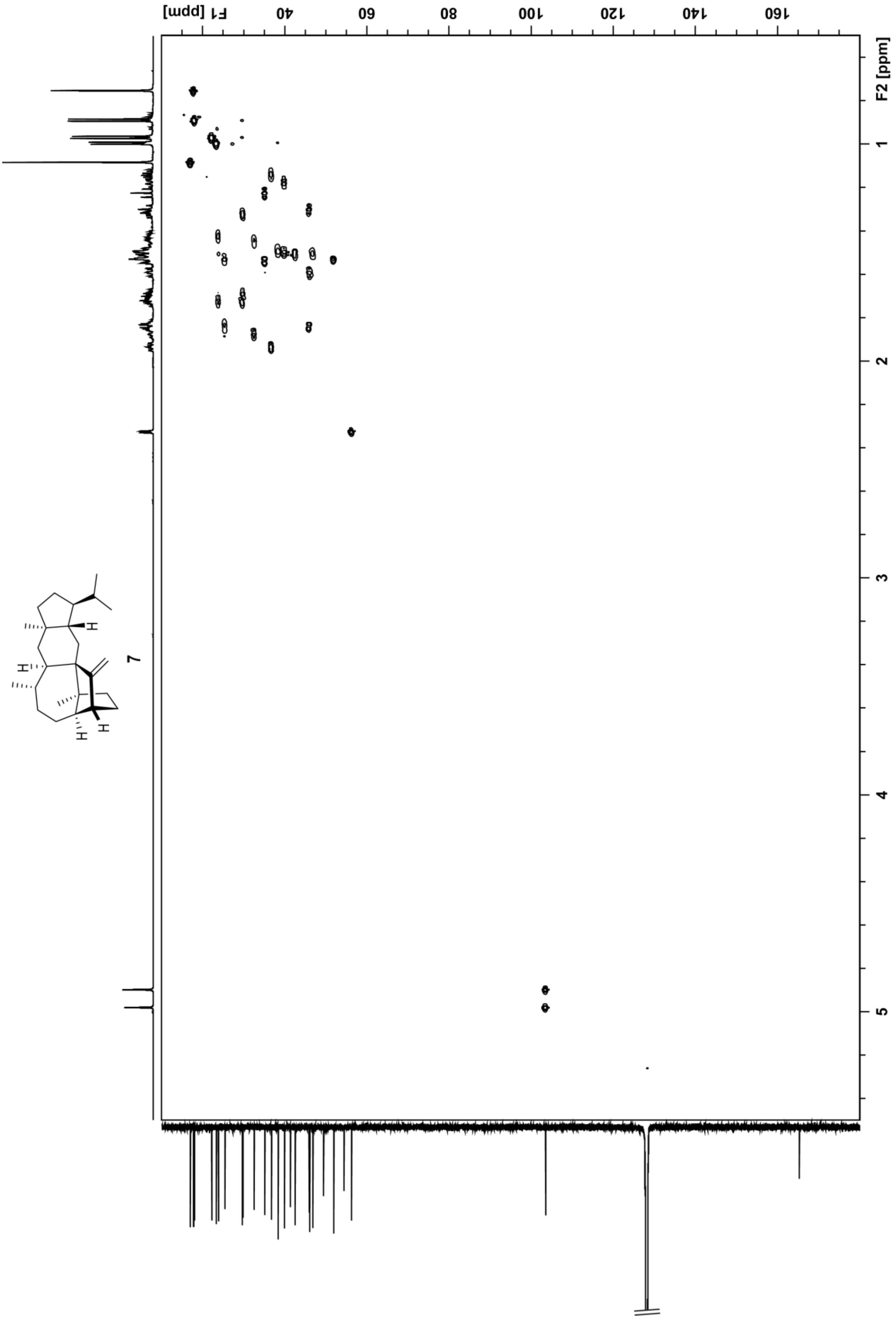

Figure S20. HSQC spectrum (C_6D_6) of **7**.

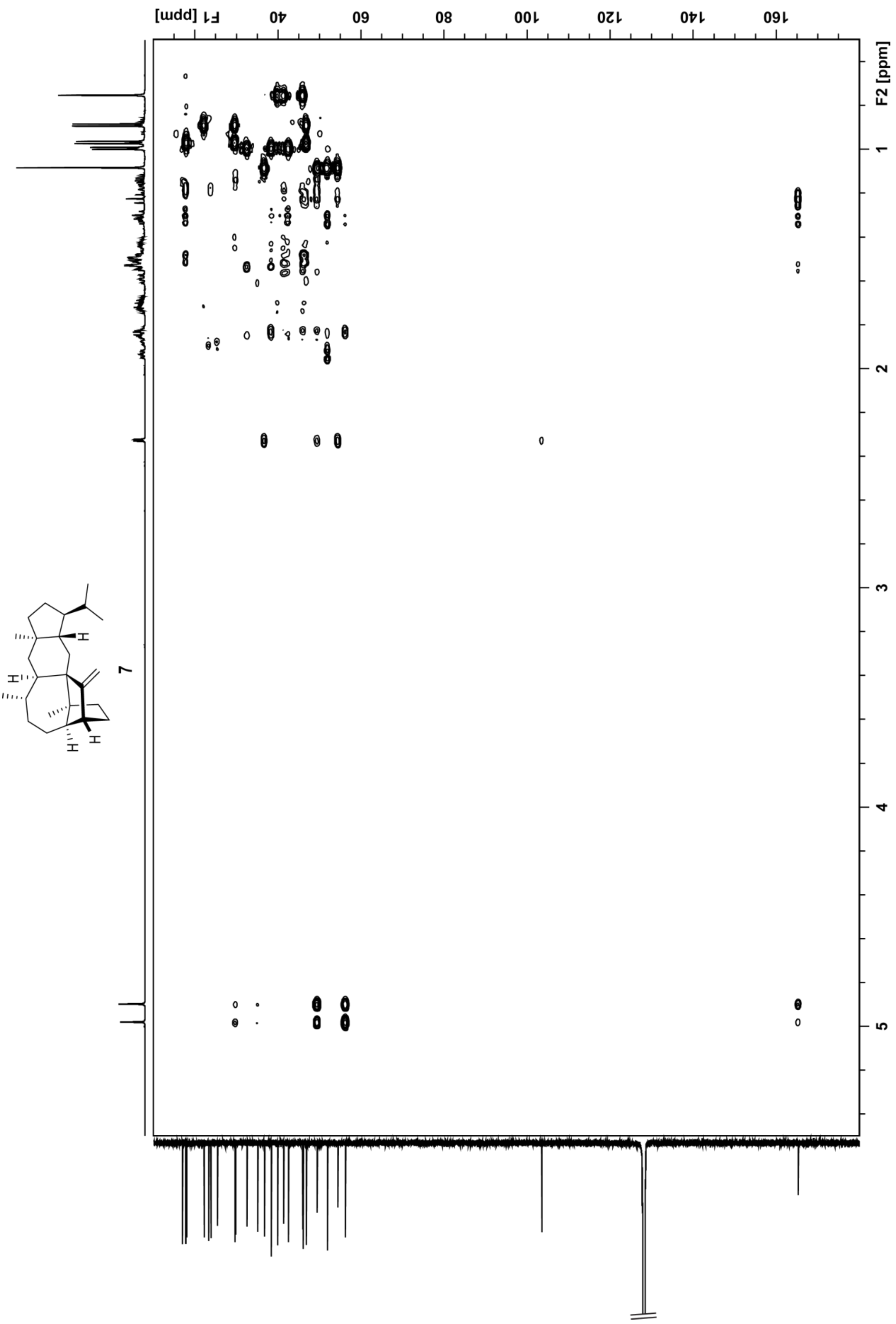

Figure S21. HMBC spectrum (C_6D_6) of **7**.

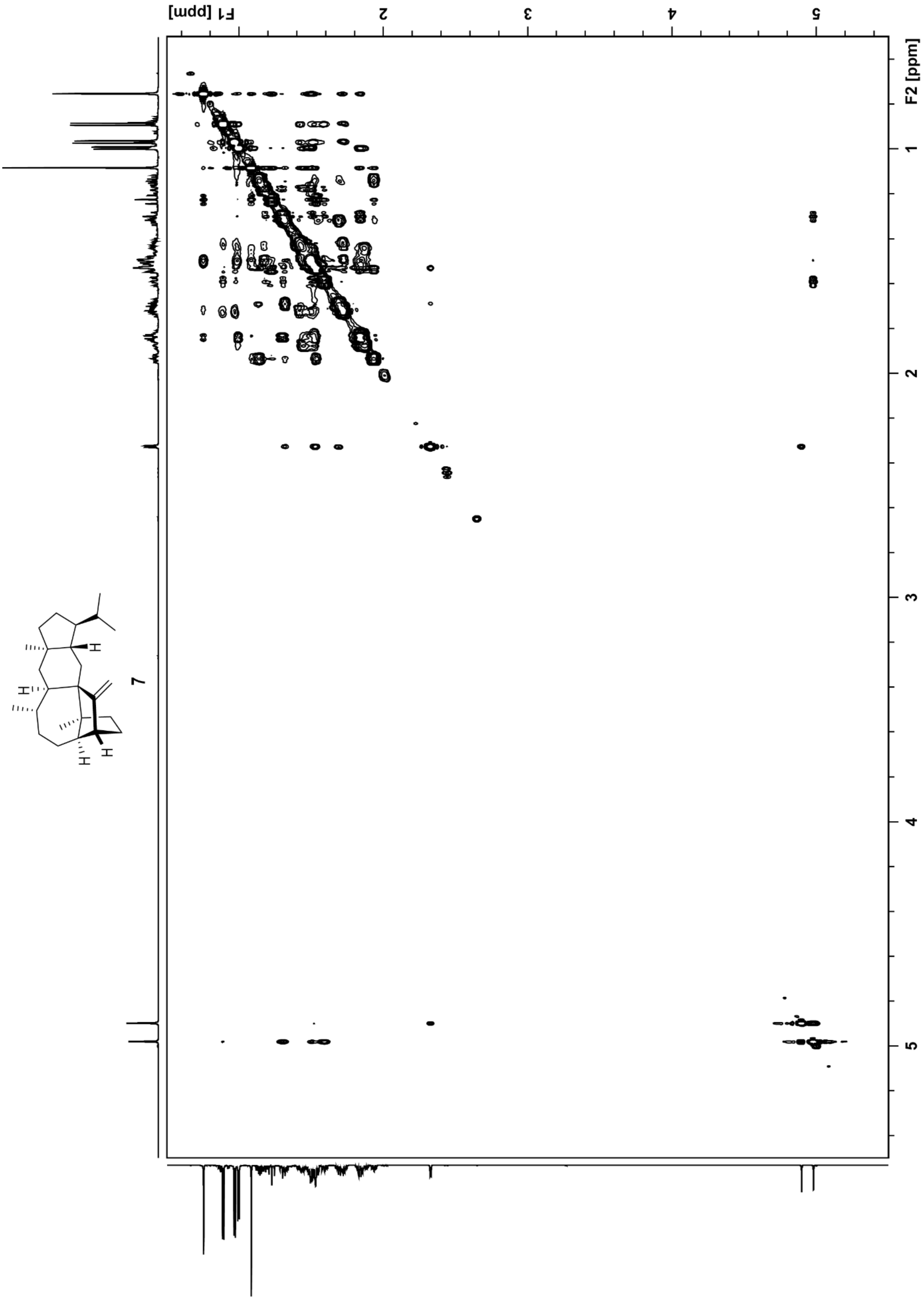

Figure S22. NOESY spectrum (C_6D_6) of **7**.

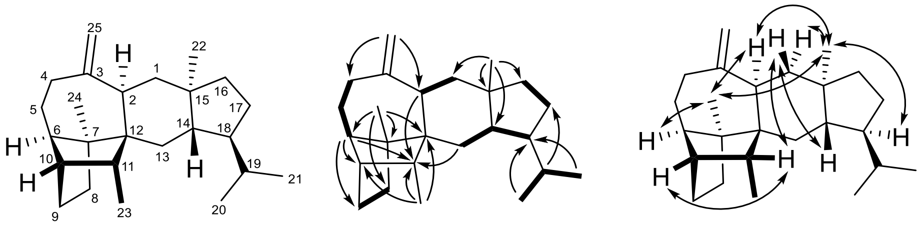

Figure S23. Structure elucidation of **8**. Bold: ^{1}H,^{1}H-COSY correlations, single-headed arrows: key HMBC correlations, and double-headed arrows: key NOESY correlations.

Table S5. NMR data of sestermobaraene C (**8**) in C_6D_6 recorded at 298 K.

C[a]		^{13}C[b]	^{1}H[b]
1	CH_2	44.34	1.80 (dd, J = 12.4, 3.3, 1H, H_α)
			1.38 (t, J = 12.6, 1H, H_β)
2	CH	44.45	2.52 (dm, J = 12.6, 1H)
3	C_q	156.75	–
4	CH_2	36.89	2.35 (m, 1H, H_α)
			2.28 (m, 1H, H_β)
5	CH_2	29.65	1.93 (dddd, J = 14.4, 10.1, 5.7, 4.6, 1H, H_α)
			1.46 (m, 1H, H_β)
6	CH	51.42	1.62 (dt, J = 9.9, 2.0, 1H)
7	C_q	53.78	–
8	CH_2	37.39	1.84 (ddd, J = 12.6, 9.2, 3.2, 1H, H_β)[c]
			1.08 (m, 1H, H_α)[c]
9	CH_2	20.80	1.47 (m, 1H, H_β)[c]
			1.27 (m, 1H, H_α)[c]
10	CH	52.33	1.50 (m, 1H)
11	CH	36.32	2.32 (m, 1H)
12	C_q	45.49	–
13	CH_2	29.30	1.68 (m, 1H, H_β)
			1.05 (m, 1H, H_α)
14	CH	47.00	1.19 (m, 1H)
15	C_q	41.32	–
16	CH_2	39.72	1.46 (m, 1H, H_α)
			1.10 (m, 1H, H_β)
17	CH_2	22.94	1.66 (m, 1H, H_α)
			1.40 (m, 1H, H_β)
18	CH	47.52	1.49 (m, 1H)
19	CH	28.71	1.76 (m, 1H)
20	CH_3	22.65	0.98 (d, J = 6.8, 3H)
21	CH_3	17.38	0.88 (d, J = 6.8, 3H)
22	CH_3	17.57	1.17 (s, 3H)
23	CH_3	15.62	1.09 (d, J = 7.2, 3H)
24	CH_3	17.61	0.76 (d, J = 0.9, 3H)
25	CH_2	112.33	4.97 (m, 1H, H_E)[d]
			4.85 (t, J = 1.8, 1H, H_Z)[d]

[a] Carbon numbering as shown in Figure S23. [b] Chemical shifts δ in ppm, multiplicity: s = singlet, d = doublet, t = triplet, m = multiplet, coupling constants J are given in Hertz. [c] For assignment of H_α and H_β cf. Figure S103. [d] Assignment according to CIP priority rules.

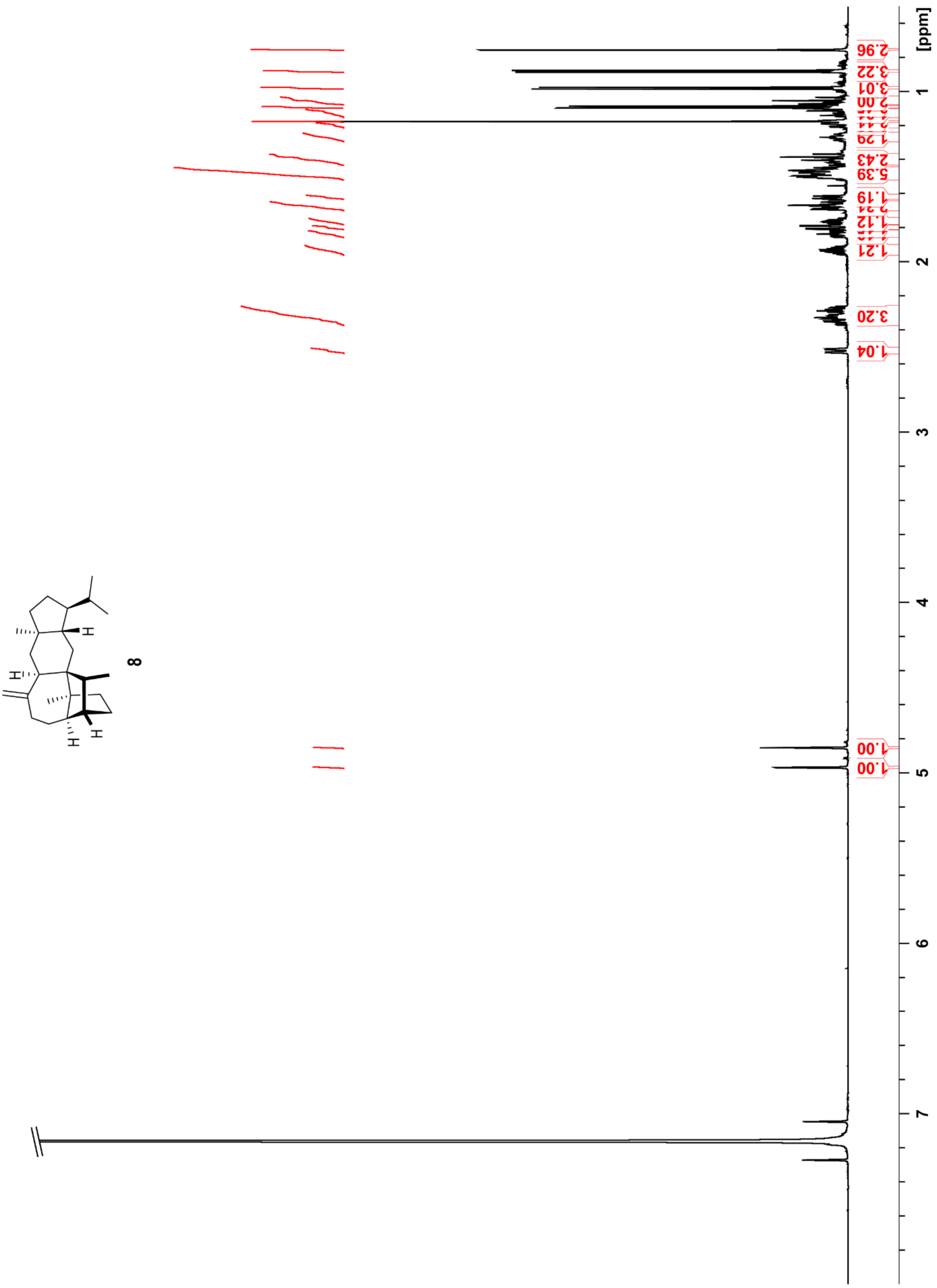

Figure S24. ^{1}H-NMR spectrum (700 MHz, C_6D_6) of **8**.

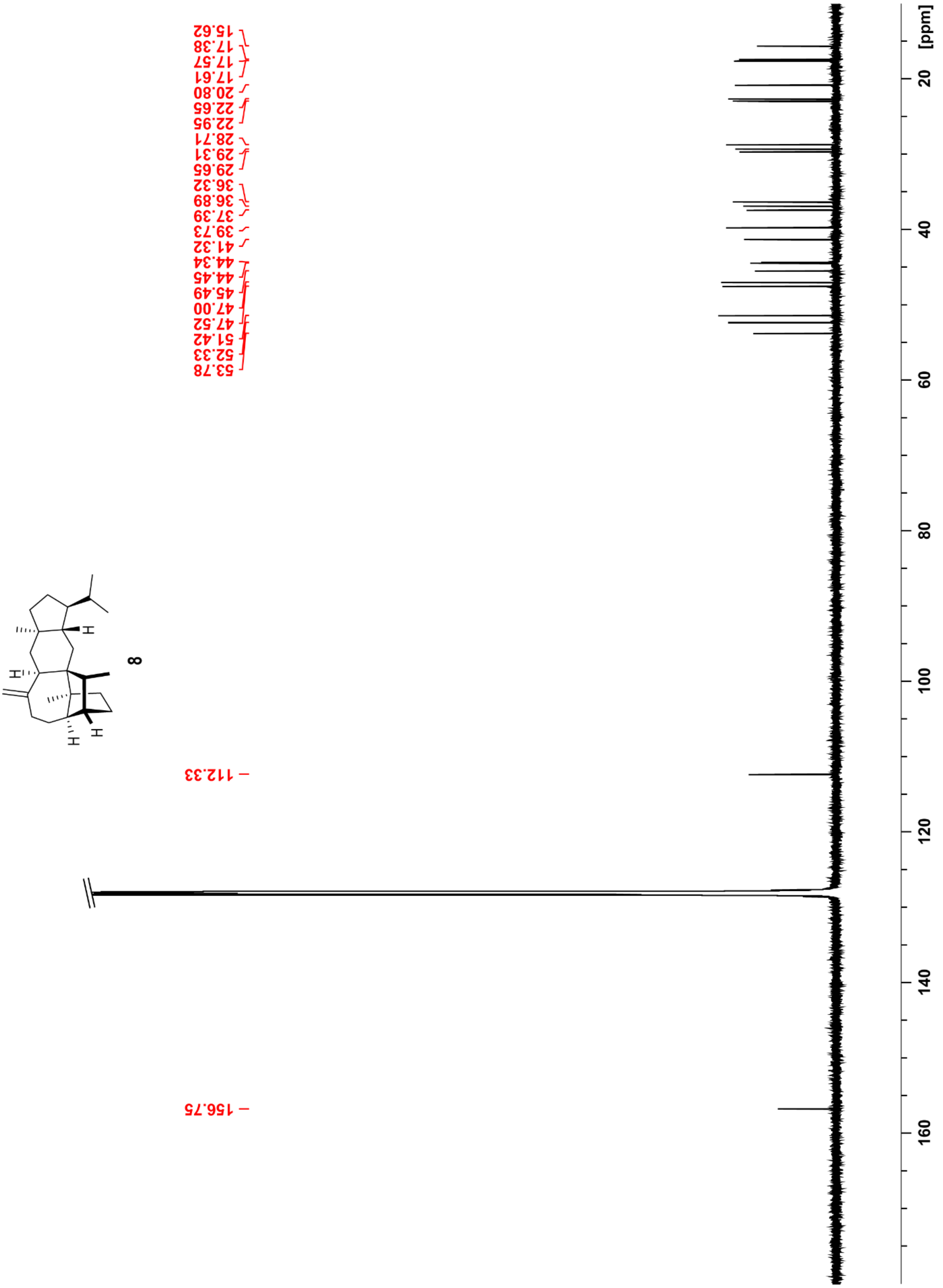

Figure S25. ^{13}C-NMR spectrum (176 MHz, C_6D_6) of **8**.

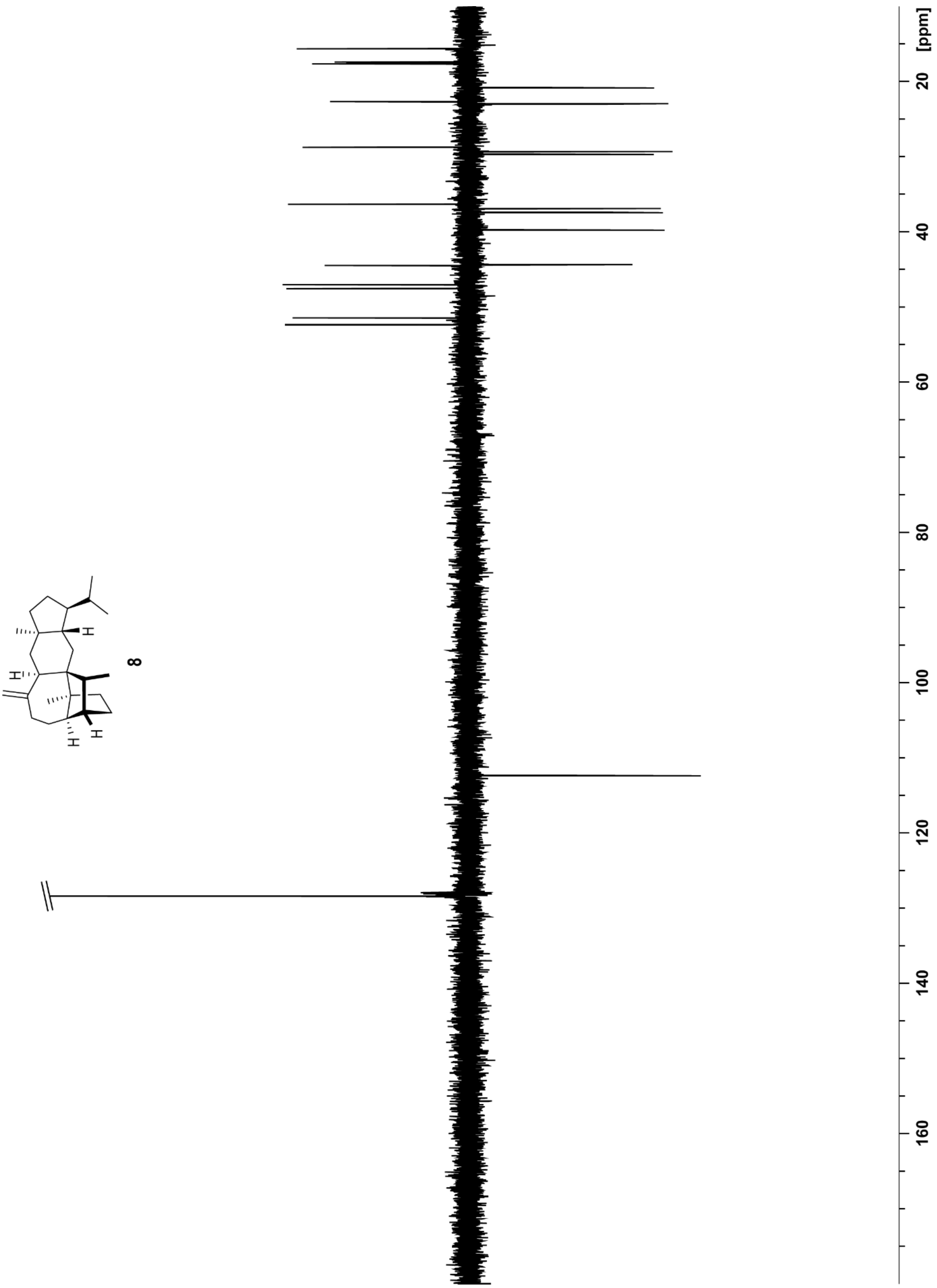

Figure S26. ^{13}C-DEPT135 spectrum (176 MHz, C_6D_6) of **8**.

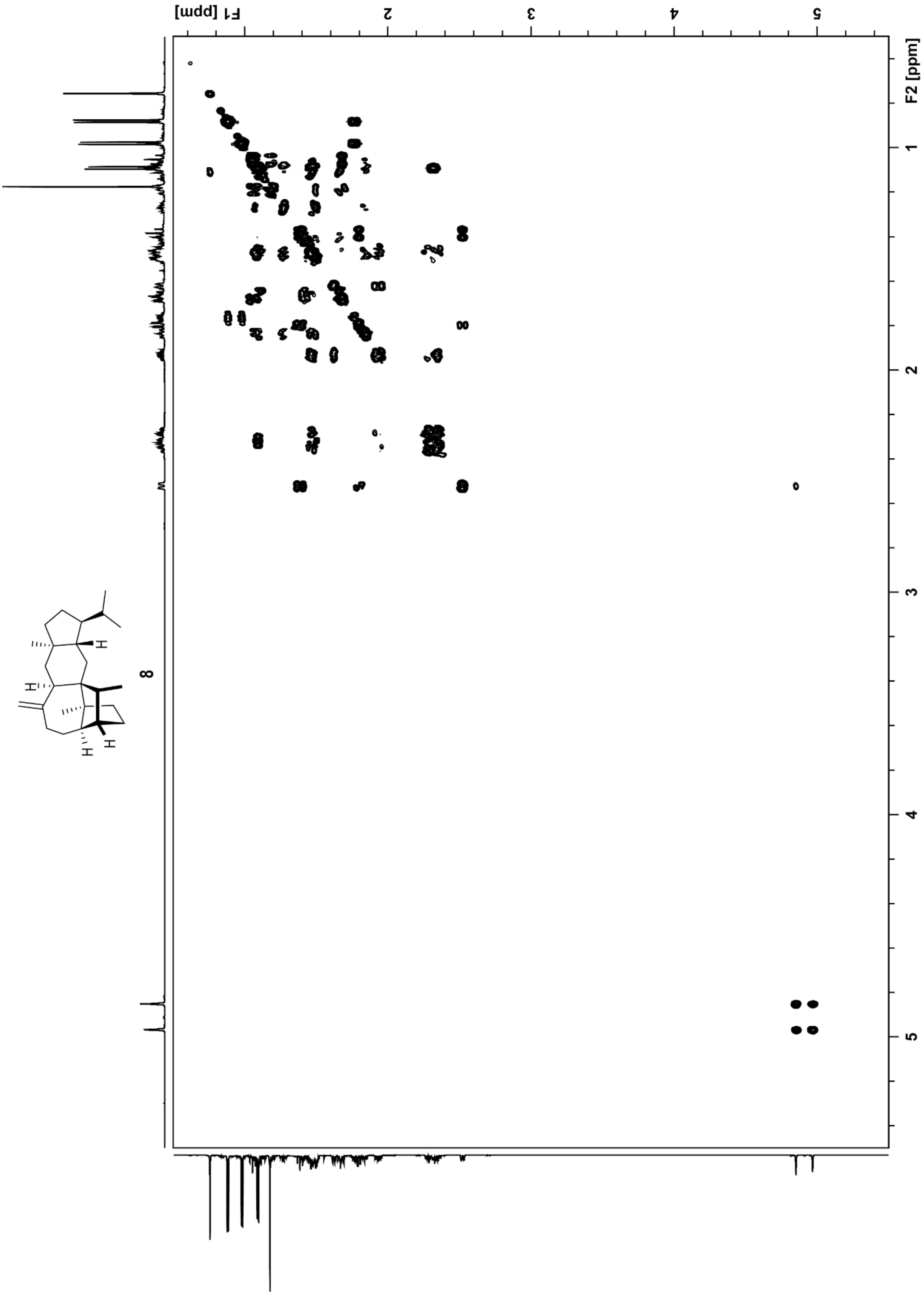

Figure S27. $^{1}H,^{1}H$-COSY spectrum (C_6D_6) of **8**.

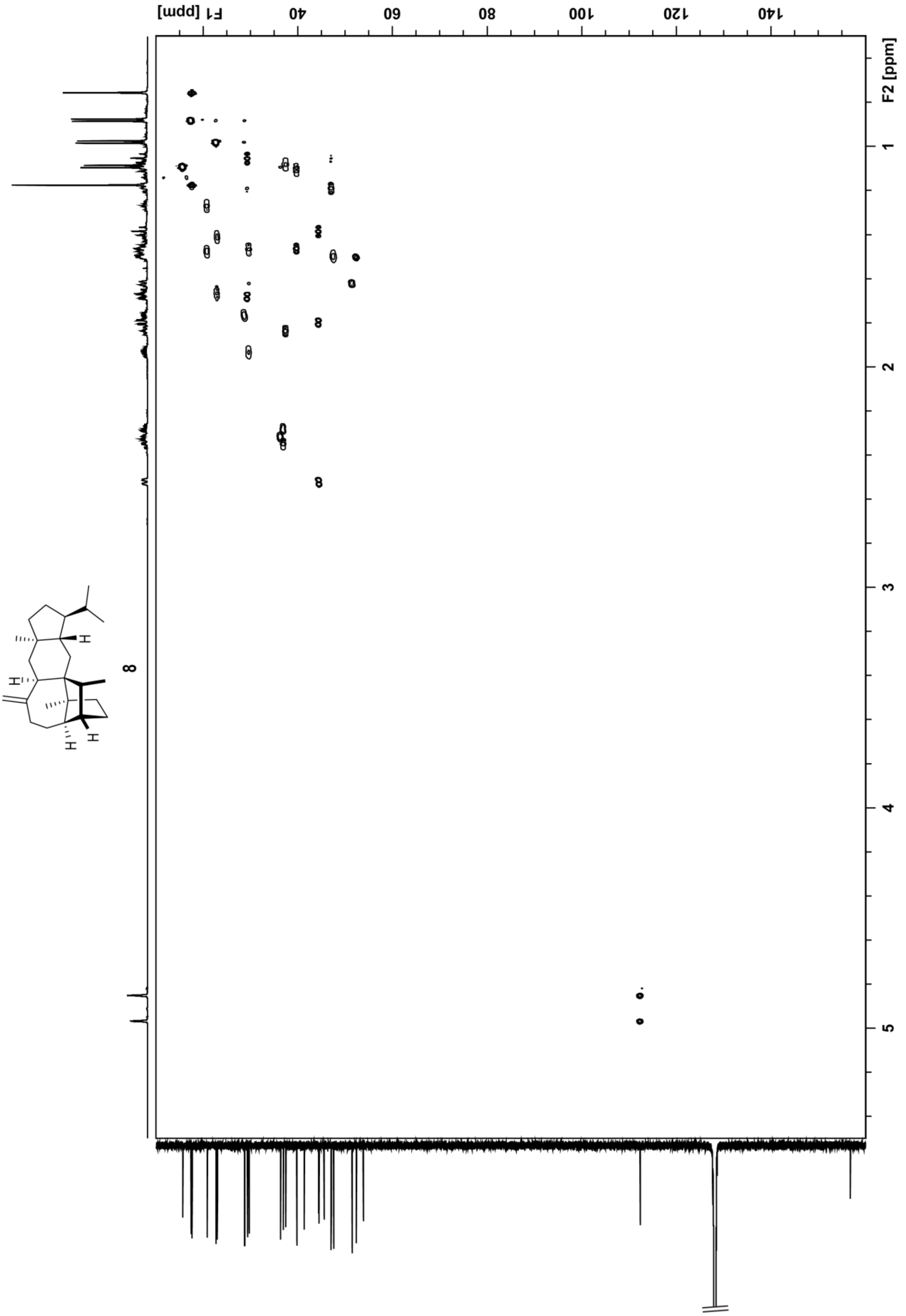

Figure S28. HSQC spectrum (C_6D_6) of **8**.

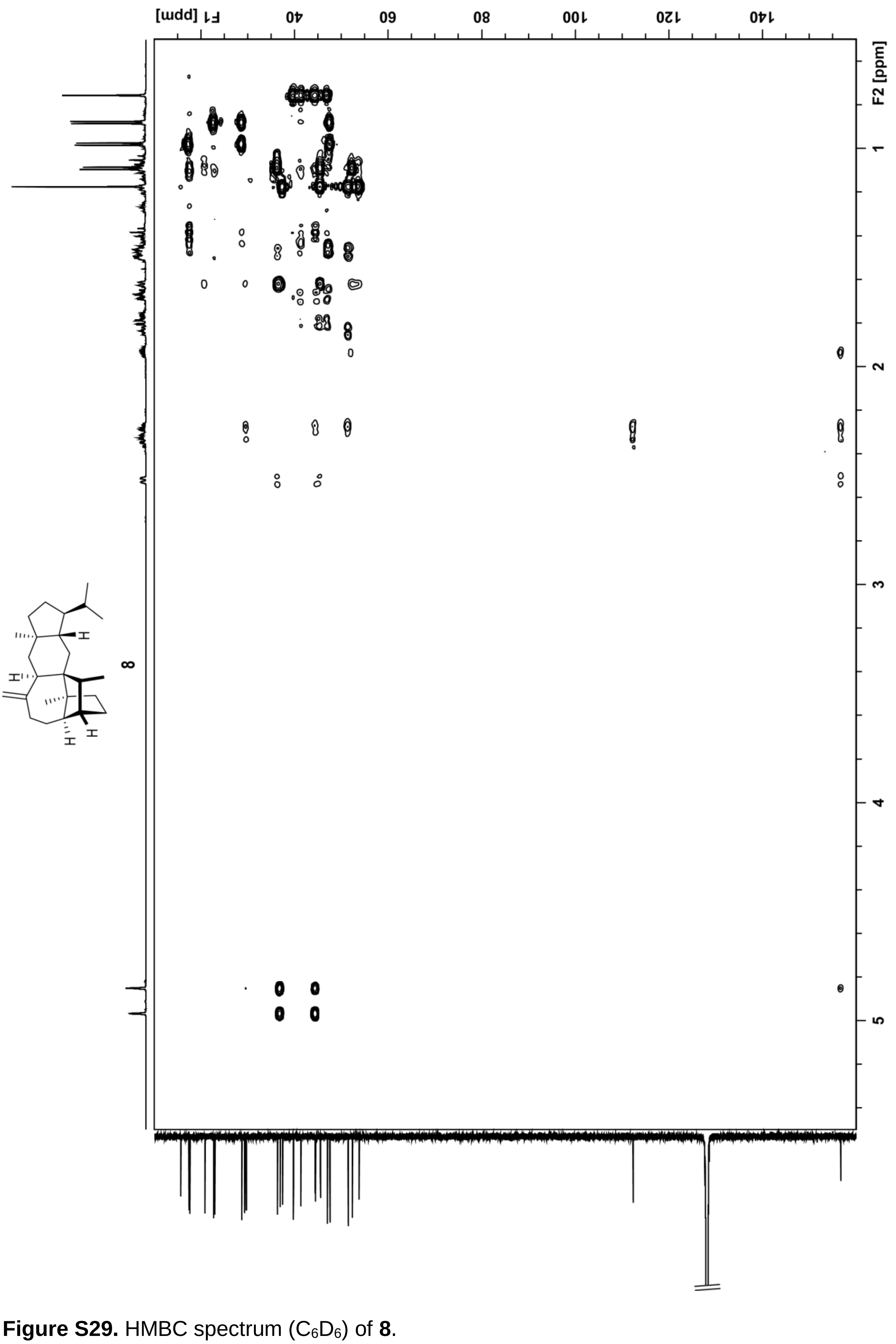

Figure S29. HMBC spectrum (C_6D_6) of **8**.

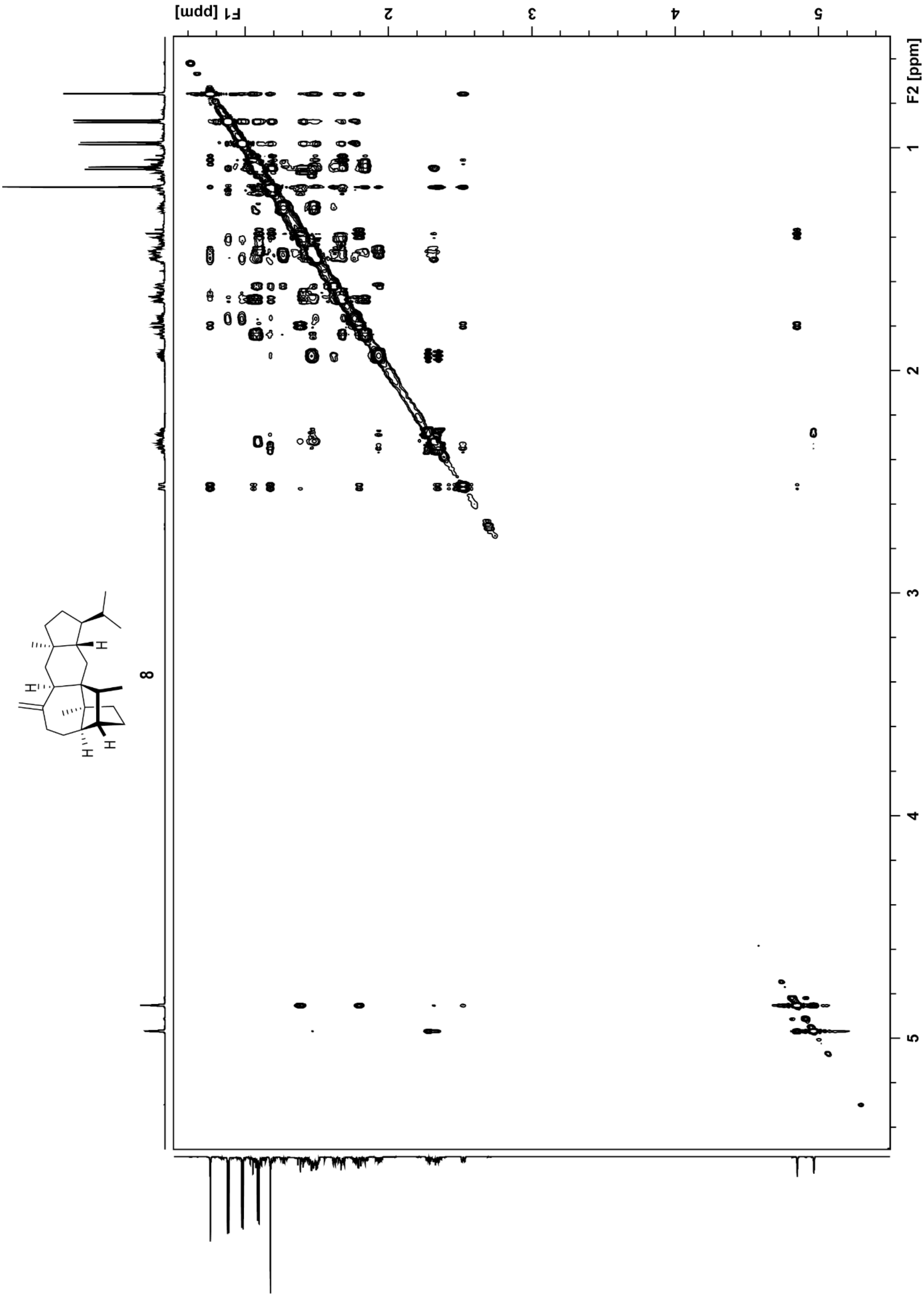

Figure S30. NOESY spectrum (C_6D_6) of **8**.

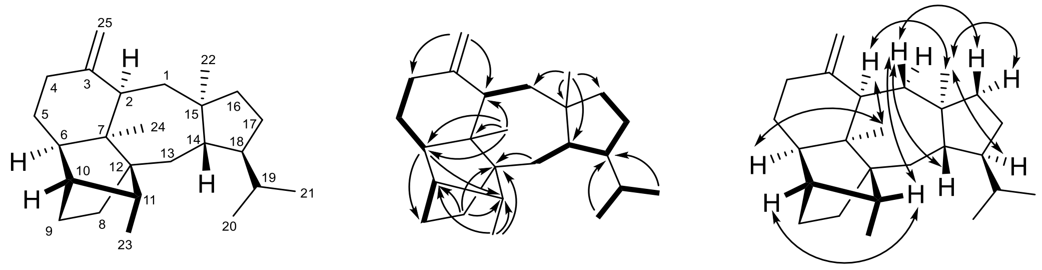

Figure S31. Structure elucidation of **9**. Bold: $^{1}H,^{1}H$-COSY correlations, single-headed arrows: key HMBC correlations, and double-headed arrows: key NOESY correlations.

Table S6. NMR data of sestermobaraene D (**9**) in C_6D_6 recorded at 298 K.

C[a]		^{13}C[b]	^{1}H[b]
1	CH_2	48.45	1.80 (m, 1H, H_β)
			1.55 (m, 1H, H_α)
2	CH	49.93	2.25 (d, *J* = 12.7, 1H)
3	C_q	153.80	–
4	CH_2	27.31	2.48 (m, 1H, H_β)
			2.16 (m , 1H, H_α)
5	CH_2	26.41	1.46 (m, 1H, H_β)
			1.34 (m, 1H, H_α)
6	CH	52.86	1.00 (dd, *J* = 13.6, 5.1, 1H)
7	C_q	46.08	–
8	CH_2	32.76	1.76 (m, 1H, H_α)[c]
			1.19 (m, 1H, H_β)[c]
9	CH_2	28.26	1.78 (m, 1H, H_β)[c]
			1.20 (m, 1H, H_α)[c]
10		47.17	1.50 (br d, *J* = 4.9, 1H)
11	CH	43.12	2.28 (q, *J* = 6.8, 1H)
12	C_q	55.36	–
13	CH_2	27.24	1.29 (m, 1H, H_α)
			1.20 (m, 1H, H_β)
14	CH	49.88	1.30 (m, 1H)
15	C_q	44.91	–
16	CH_2	40.92	1.32 (m, 1H, H_α)
			1.13 (m, 1H, H_β)
17	CH_2	21.43	1.61 (m, 1H, H_α)
			1.45 (m, 1H, H_β)
18	CH	47.79	1.52 (m, 1H)
19	CH	27.31	1.78 (m, 1H)
20	CH_3	16.03	0.89 (d, *J* = 6.7, 3H)
21	CH_3	23.60	0.95 (d, *J* = 6.9, 3H)
22	CH_3	18.41	0.83 (s, 3H)
23	CH_3	10.87	0.86 (d, *J* = 6.6, 3H)
24	CH_3	29.20	1.07 (s, 3H)
25	CH_2	109.88	4.88 (q, *J* = 2.3, 1H, H_Z)[d]
			4.83 (q, *J* = 2.2, 1H, H_E)[d]

[a] Carbon numbering as shown in Figure S31. [b] Chemical shifts δ in ppm, multiplicity: s = singlet, d = doublet, q = quartet, br =broad, m = multiplet, coupling constants *J* are given in Hertz. [c] For assignment of H_α and H_β cf. Figure S104. [d] Assignment according to CIP priority rules.

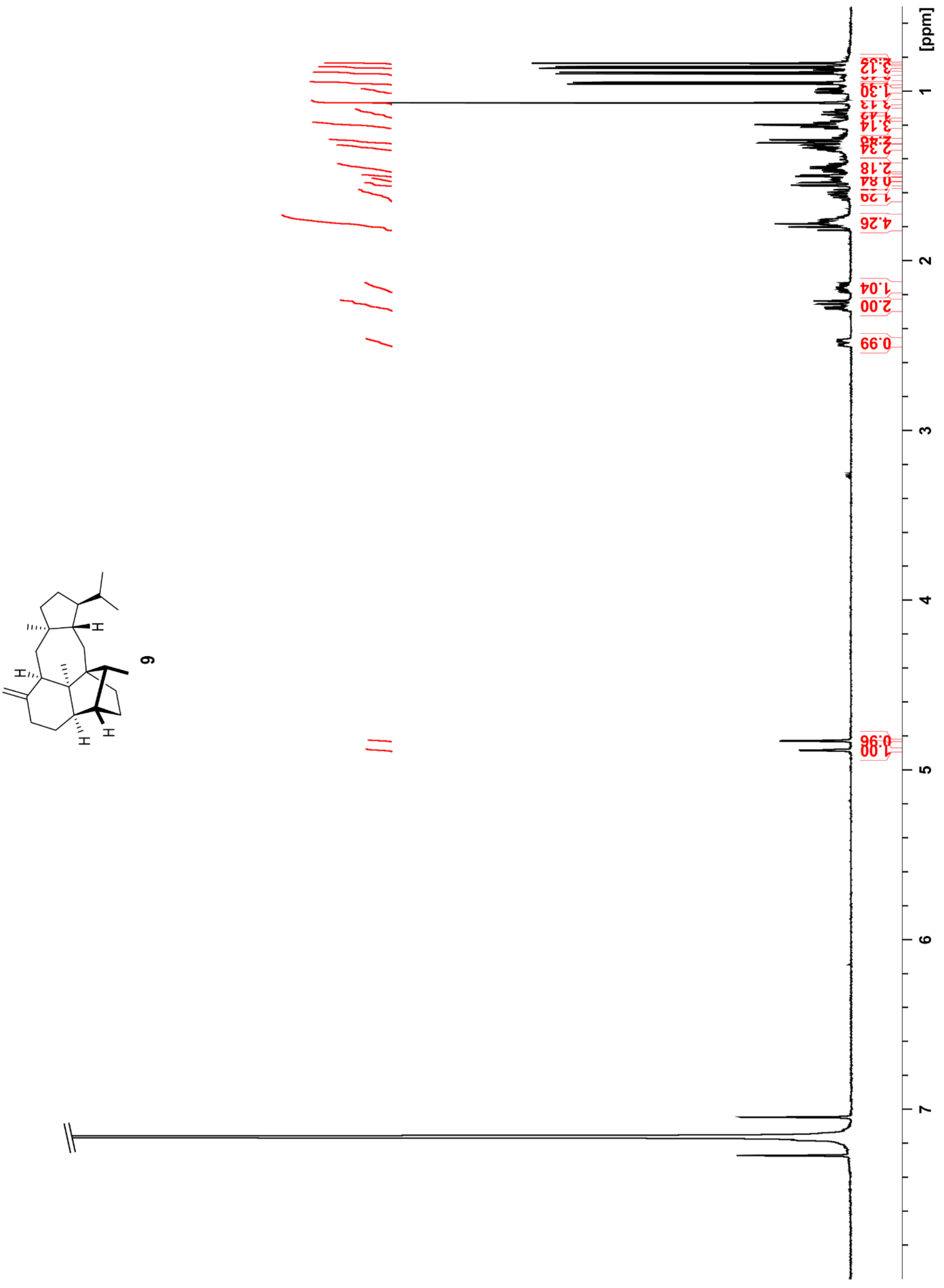

Figure S32. ^{1}H-NMR spectrum (700 MHz, C_6D_6) of **9**.

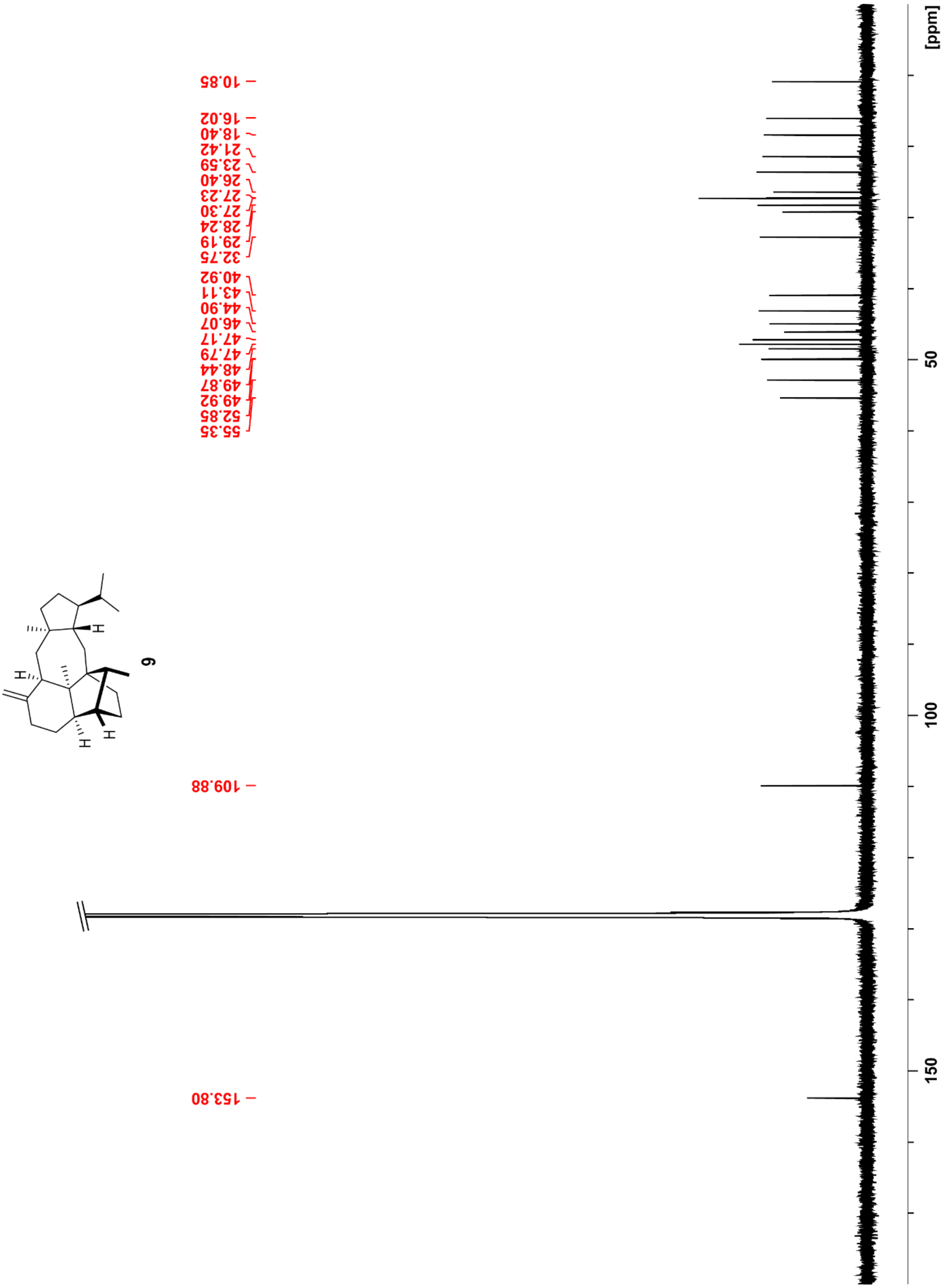

Figure S33. ^{13}C-NMR spectrum (176 MHz, C_6D_6) of **9**.

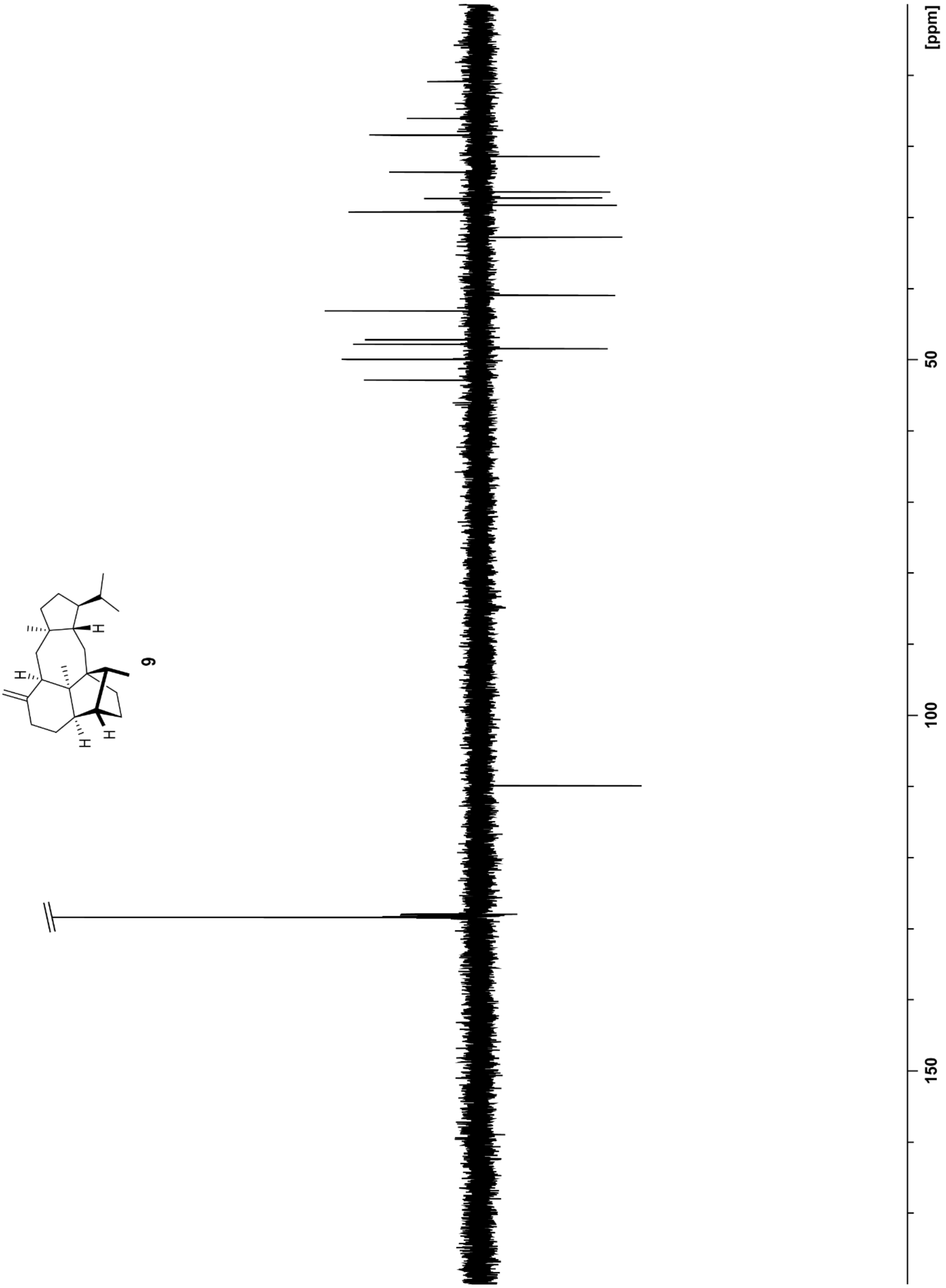

Figure S34. ^{13}C-DEPT135 spectrum (176 MHz, C_6D_6) of **9**.

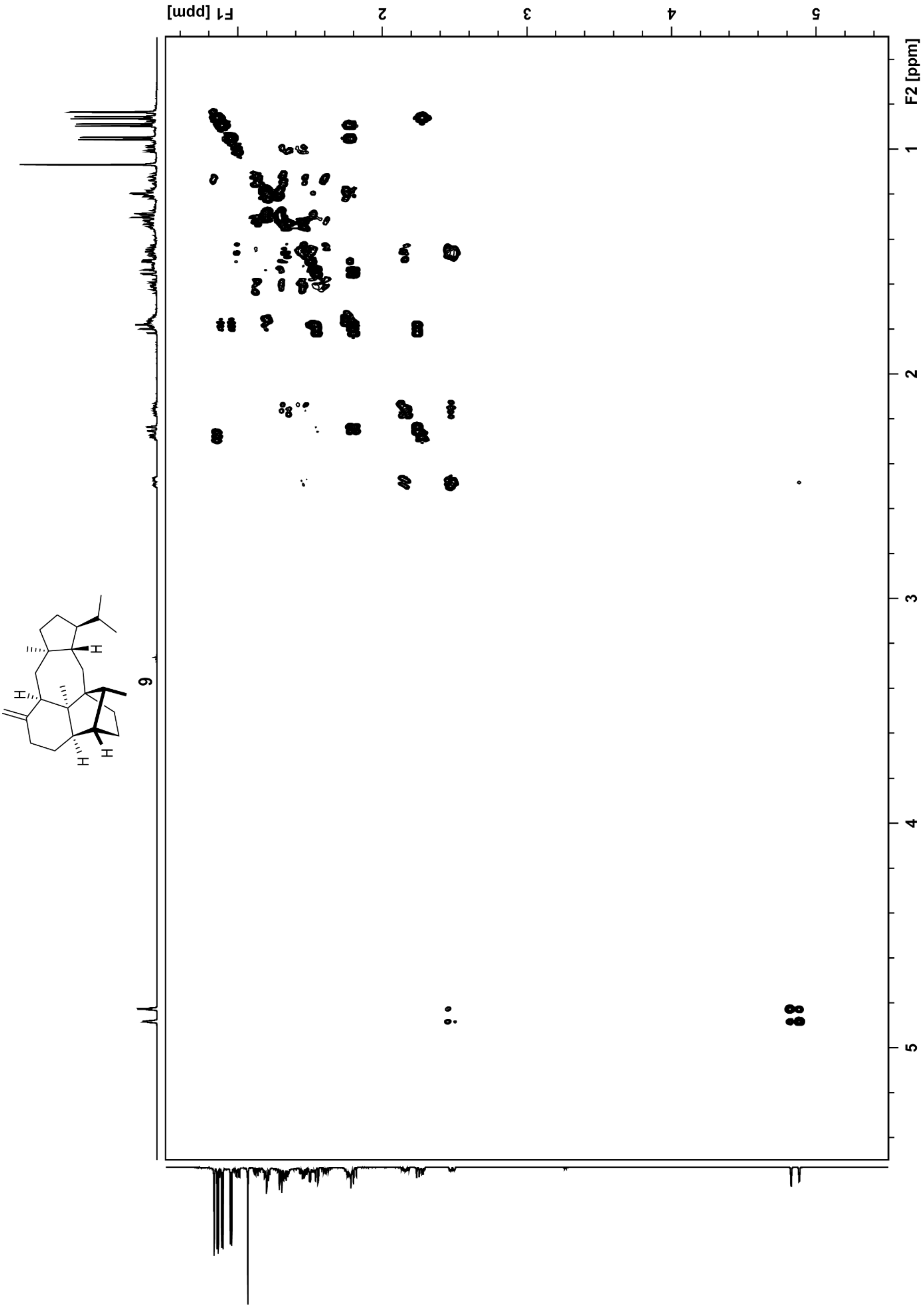

Figure S35. $^{1}H,^{1}H$-COSY spectrum (C_6D_6) of **9**.

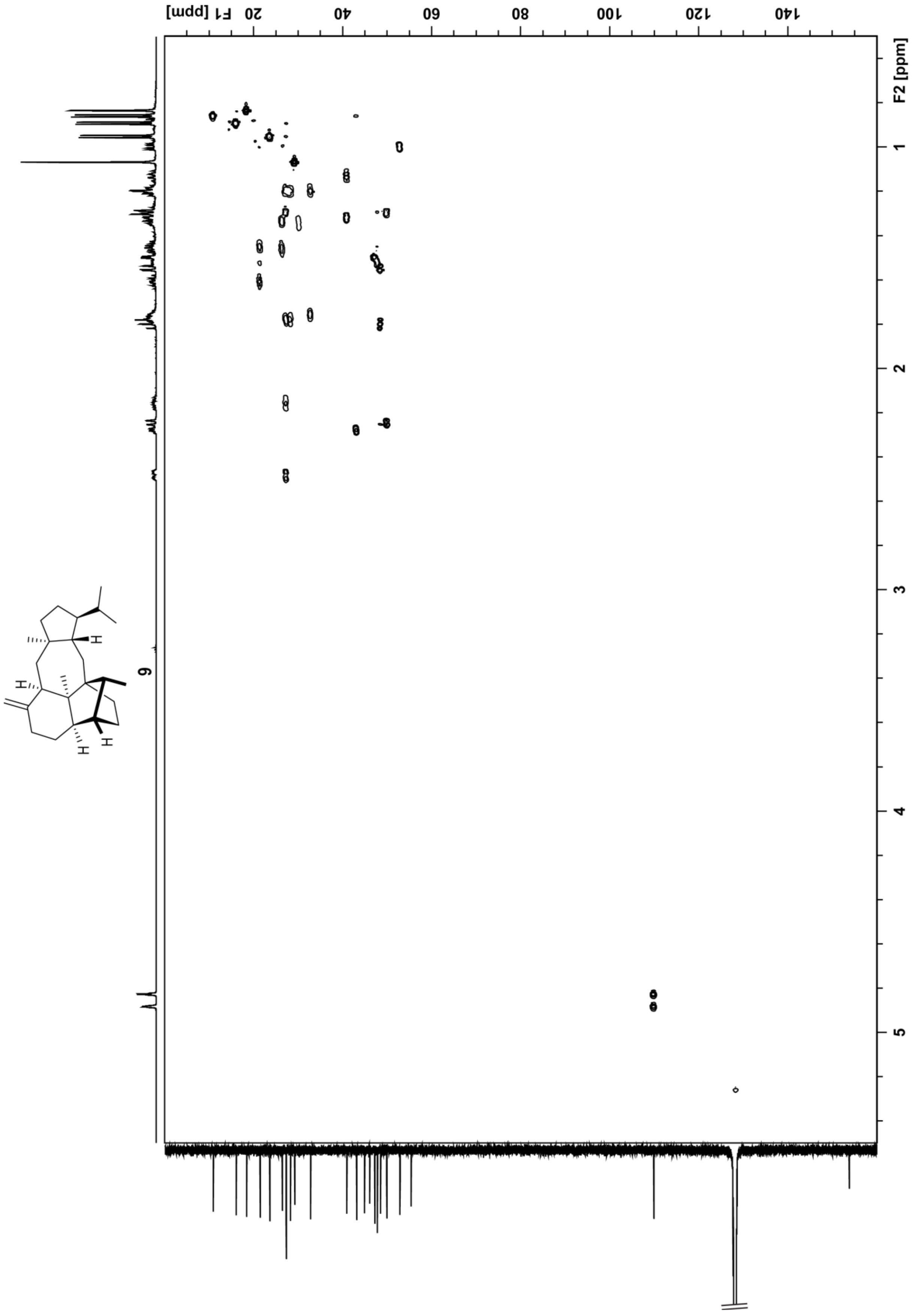

Figure S36. HSQC spectrum (C_6D_6) of **9**.

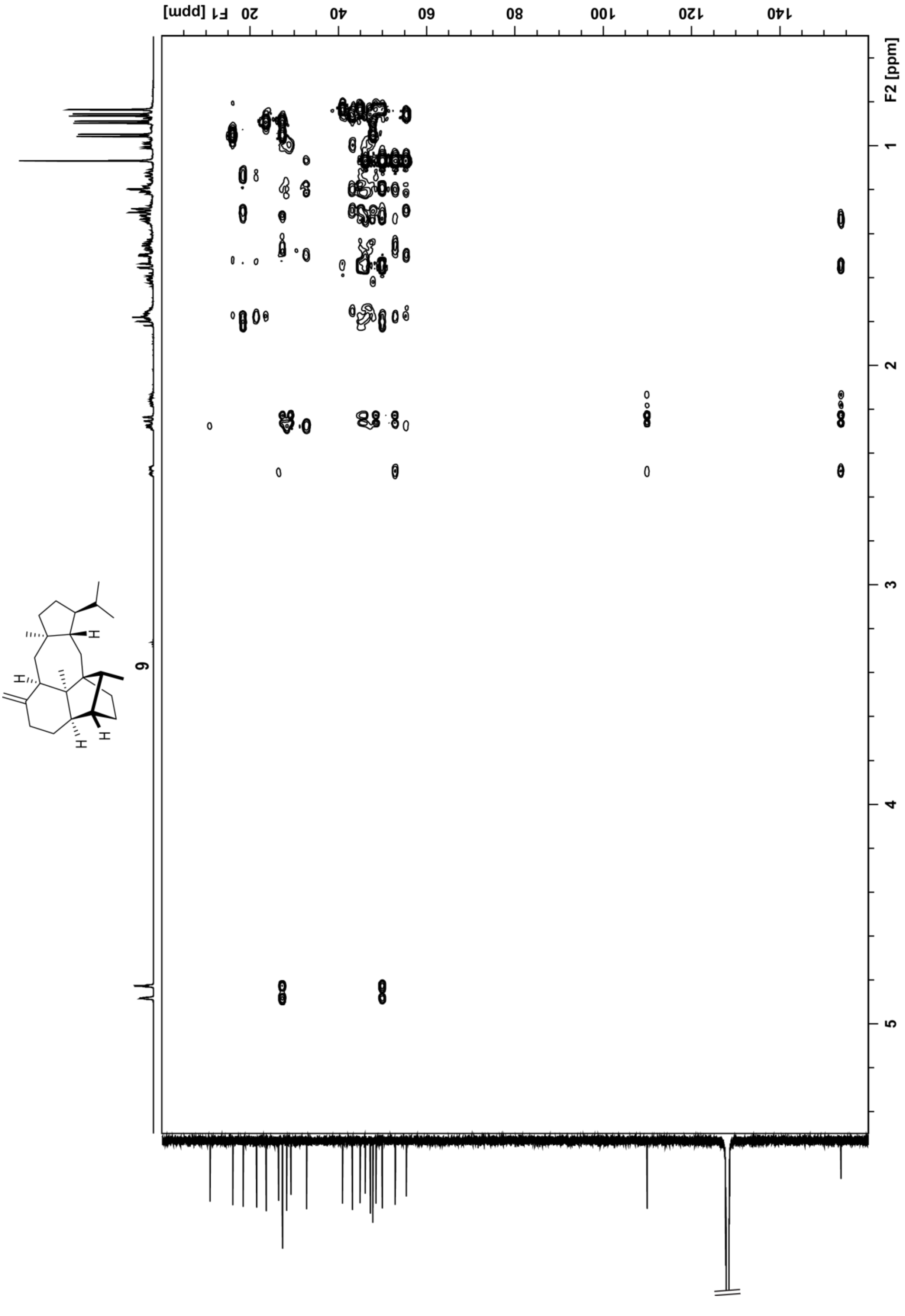

Figure S37. HMBC spectrum (C_6D_6) of **9**.

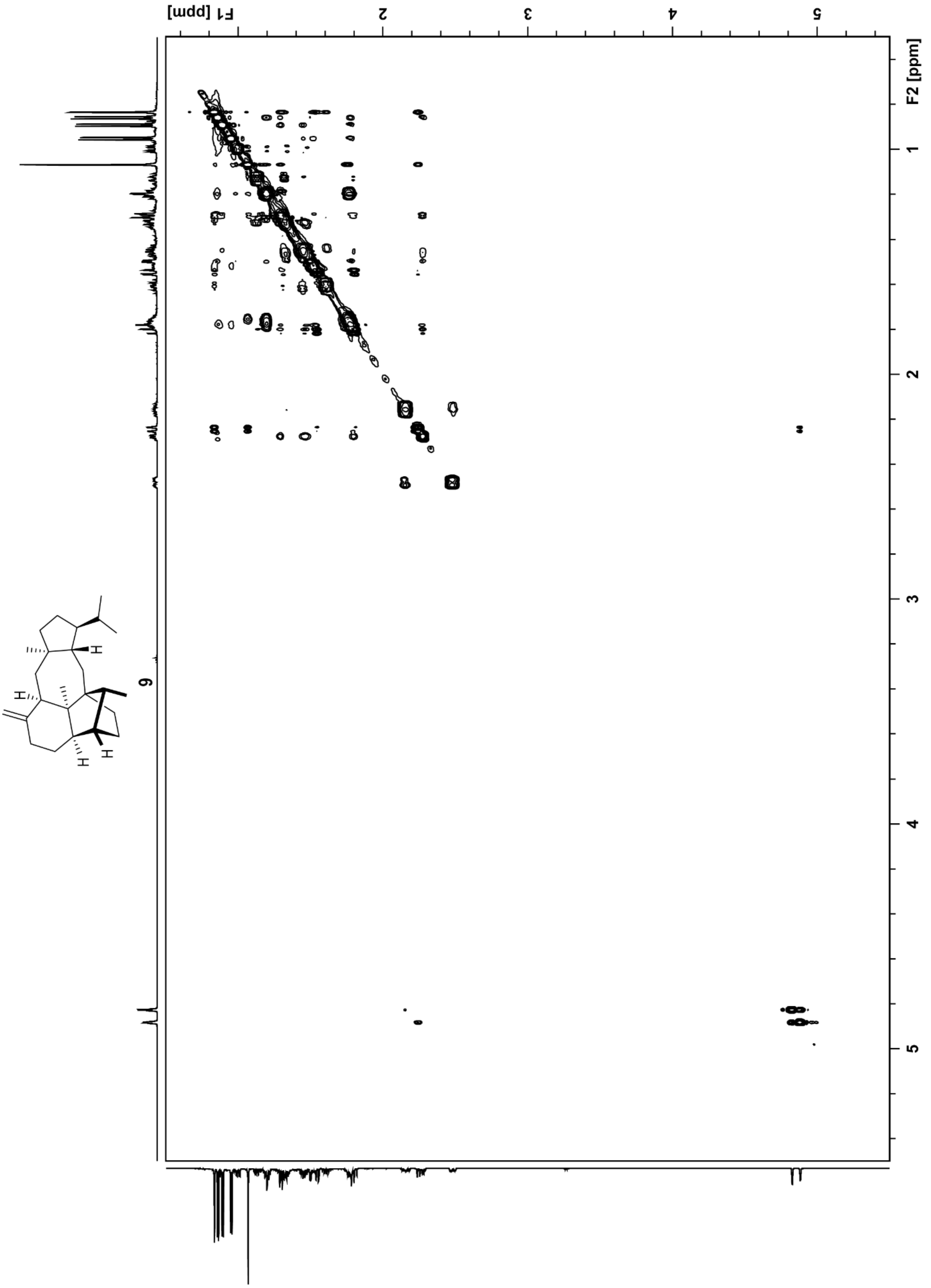

Figure S38. NOESY spectrum (C_6D_6) of **9**.

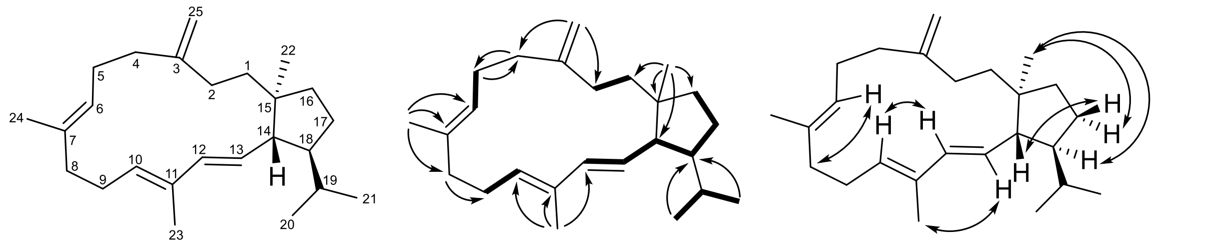

Figure S39. Structure elucidation of **10**. Bold: $^{1}H,^{1}H$-COSY correlations, single-headed arrows: key HMBC correlations, and double-headed arrows: key NOESY correlations.

Table S7. NMR data of sestermobaraene E (**10**) in C_6D_6 recorded at 298 K.

C[a]		^{13}C[b]	^{1}H[b]
1	CH_2	39.27	1.59 (m, 1H, H_β)
			1.52 (m, 1H, H_α)
2	CH_2	30.66	2.17 (m, 1H)
			1.80 (m, 1H)
3	C_q	150.12	–
4	CH_2	37.64	2.10 (m, 1H, H_β)
			2.07 (m, 1H, H_α)
5	CH_2	25.41	2.10 (m, 2H)
6	CH	126.31	5.06 (m, 1H)
7	C_q	132.97	–
8	CH_2	39.48	2.07 (m, 1H, H_α)
			1.95 (m, 1H, H_β)
9	CH_2	25.31	2.30 (m, 1H, H_α)
			1.96 (m, 1H, H_β)
10	CH	131.57	5.09 (dd, J = 10.9, 3.2, 1H)
11	C_q	133.76	–
12	CH	138.38	6.13 (d, J = 15.7, 1H)
13	CH	127.91	5.38 (dd, J = 15.4, 9.8, 1H)
14	CH	58.54	1.98 (m, 1H)
15	C_q	46.25	–
16	CH_2	40.00	1.38 (m, 1H, H_β)
			1.34 (m, 1H, H_α)
17	CH_2	23.96	1.71 (m, 1H, H_α)
			1.38 (m, 1H, H_β)
18	CH	49.06	1.85 (m, 1H)
19	CH	30.59	1.70 (m, 1H)
20	CH_3	22.32	0.95 (d, J = 6.9, 3H)
21	CH_3	18.44	0.89 (d, J = 6.8, 3H)
22	CH_3	19.65	0.83 (s, 3H)
23	CH_3	12.45	1.62 (s, 3H)
24	CH_3	15.30	1.41 (s, 3H)
25	CH_2	108.09	4.93 (br s, 1H, H_Z)[c]
			4.92 (br s, 1H, H_E)[c]

[a] Carbon numbering as shown in Figure S39. [b] Chemical shifts δ in ppm, multiplicity: s = singlet, d = doublet, br = broad, m = multiplet, coupling constants J are given in Hertz. [c] Assignment according to CIP priority rules.

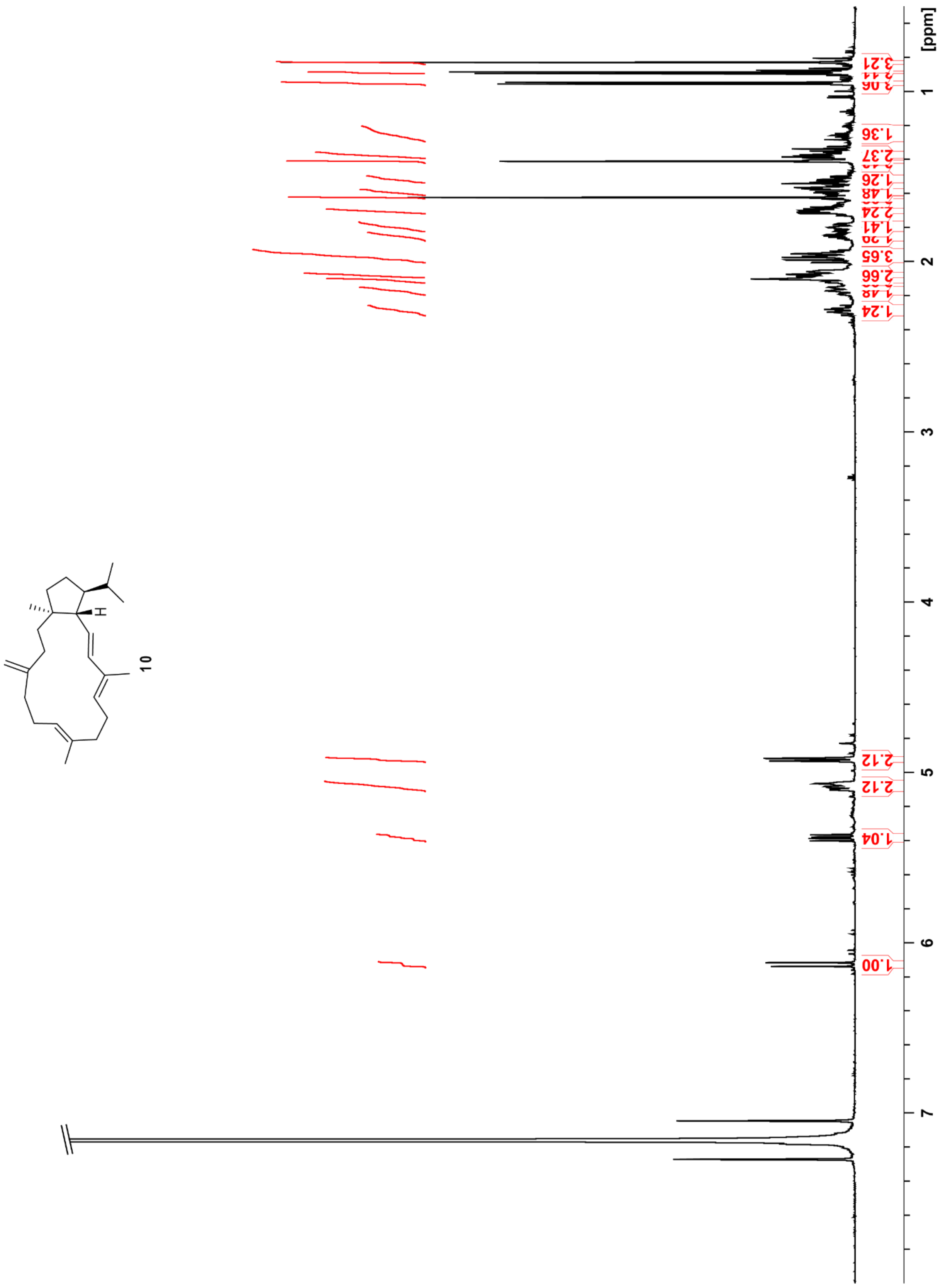

Figure S40. ^{1}H-NMR spectrum (700 MHz, C_6D_6) of **10**.

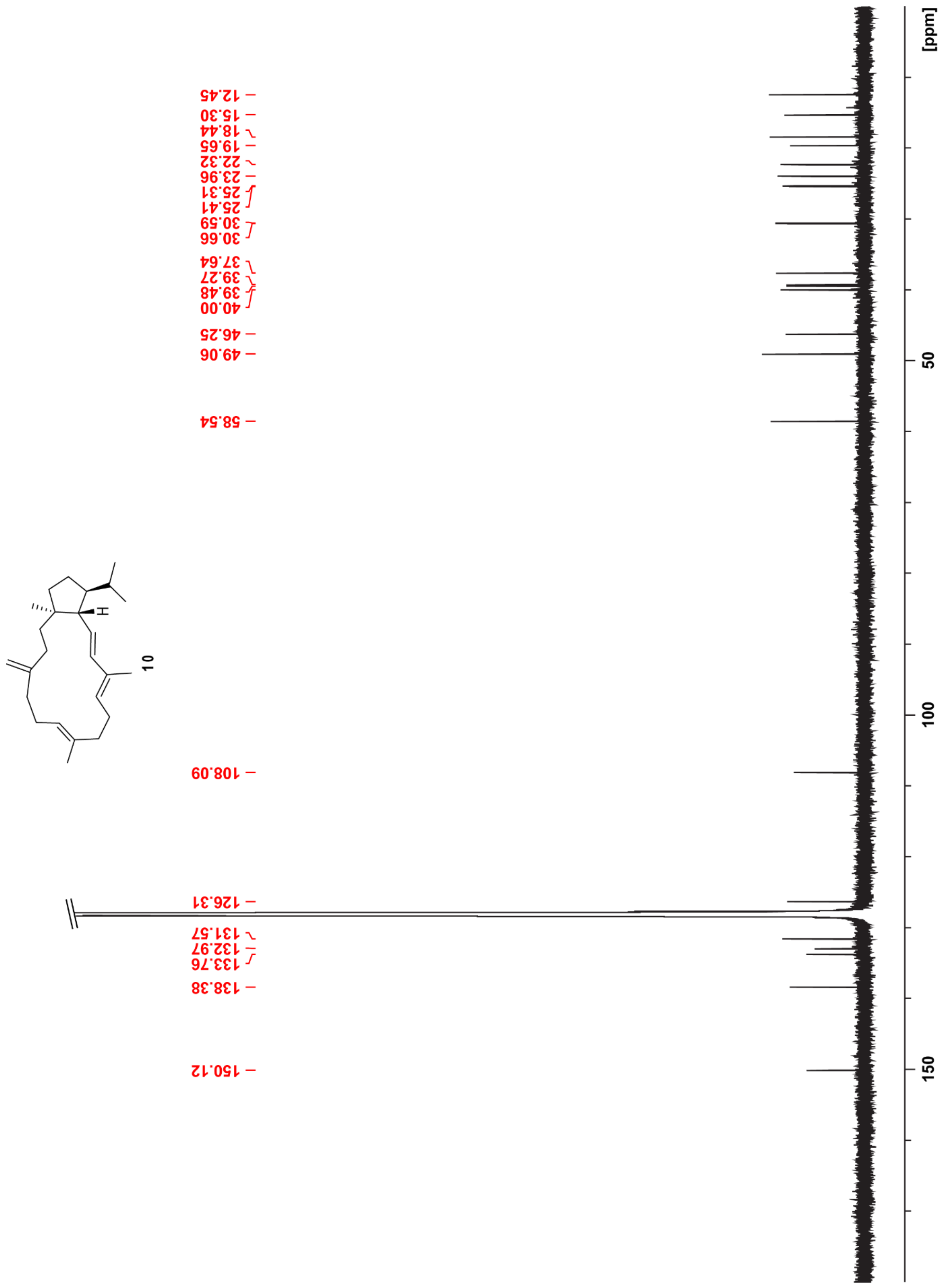

Figure S41. ^{13}C-NMR spectrum (176 MHz, C_6D_6) of **10**.

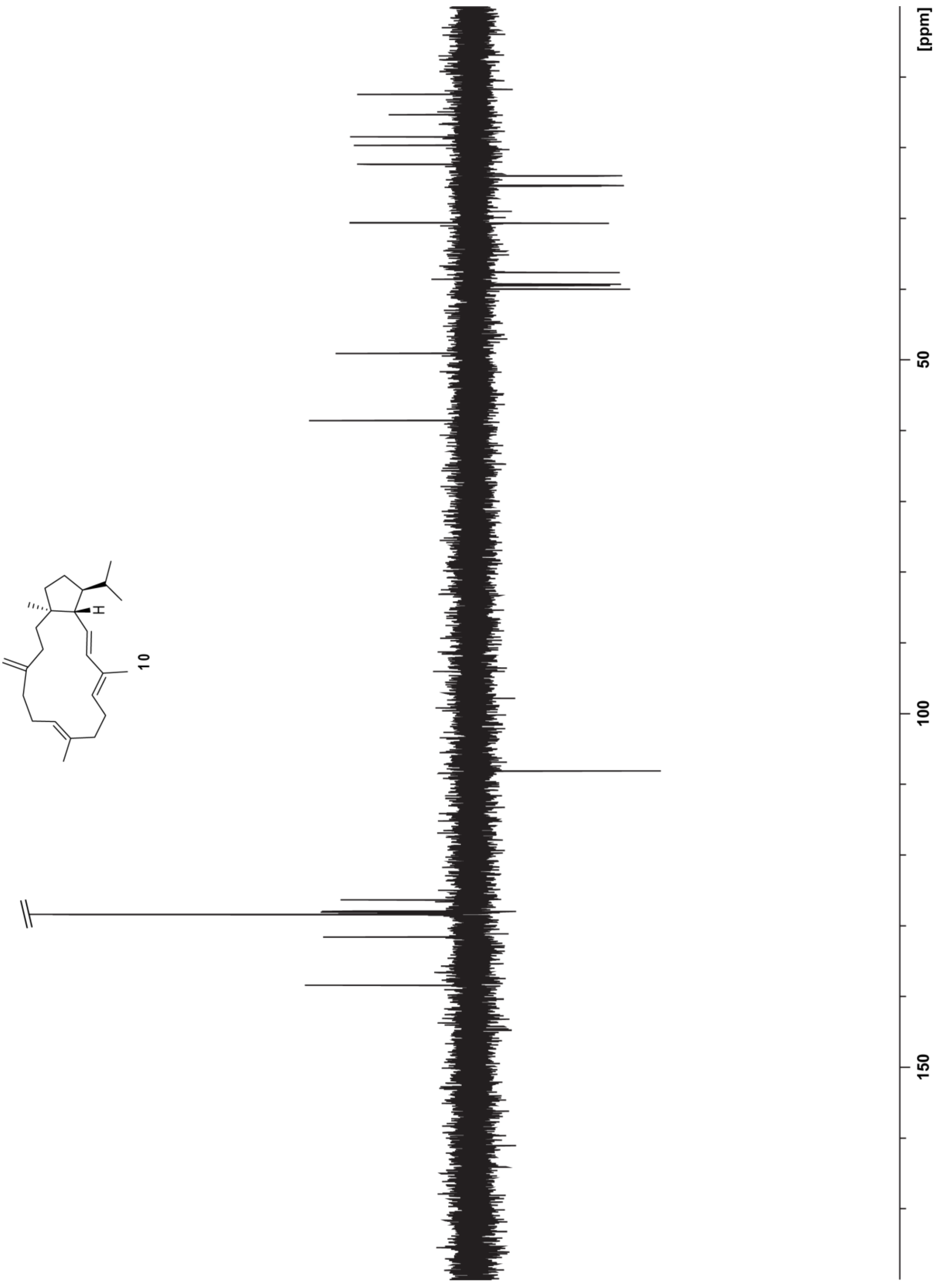

Figure S42. ^{13}C-DEPT135 spectrum (176 MHz, C_6D_6) of **10**.

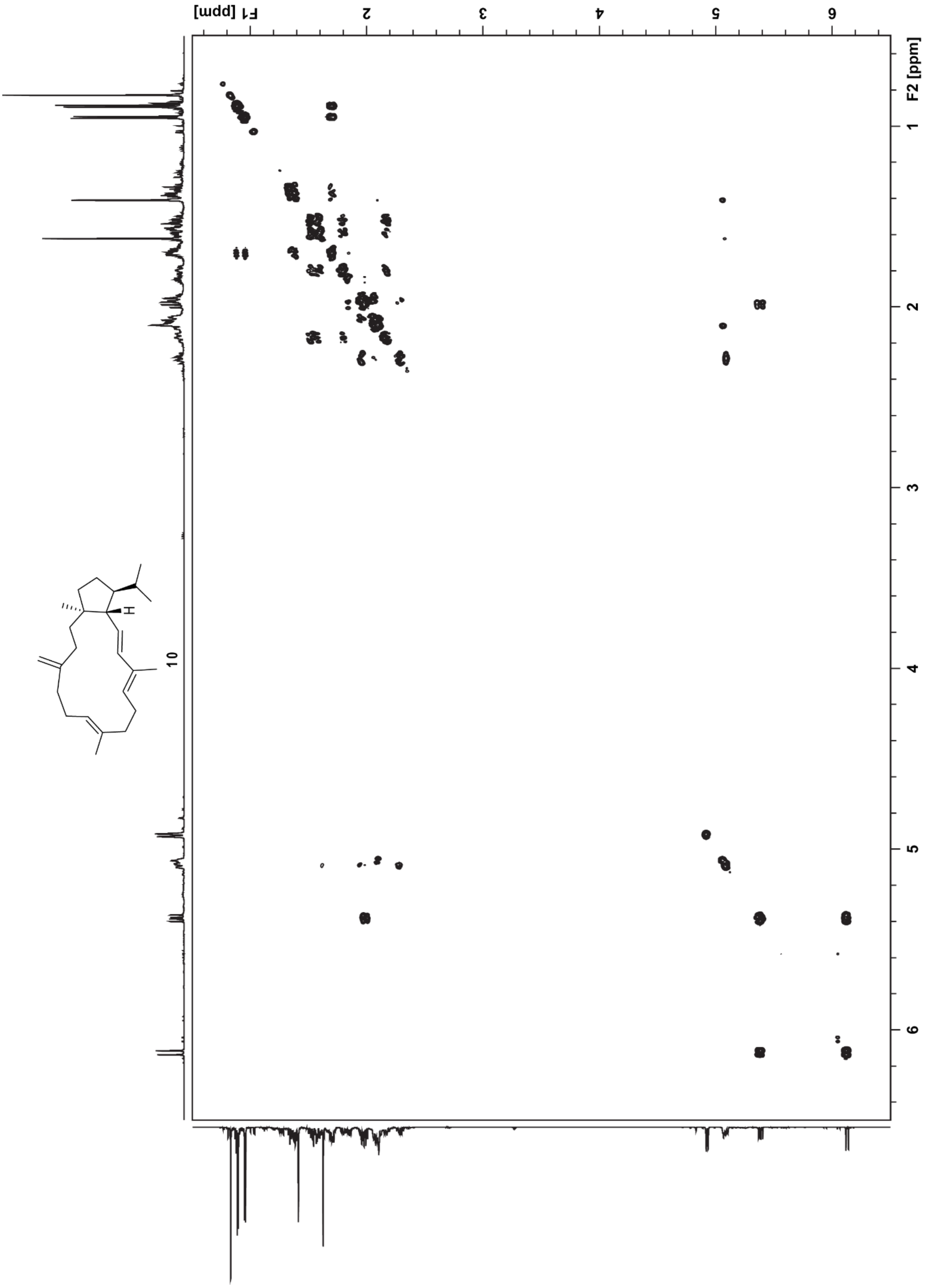

Figure S43. ^{1}H,^{1}H-COSY spectrum (C_6D_6) of **10**.

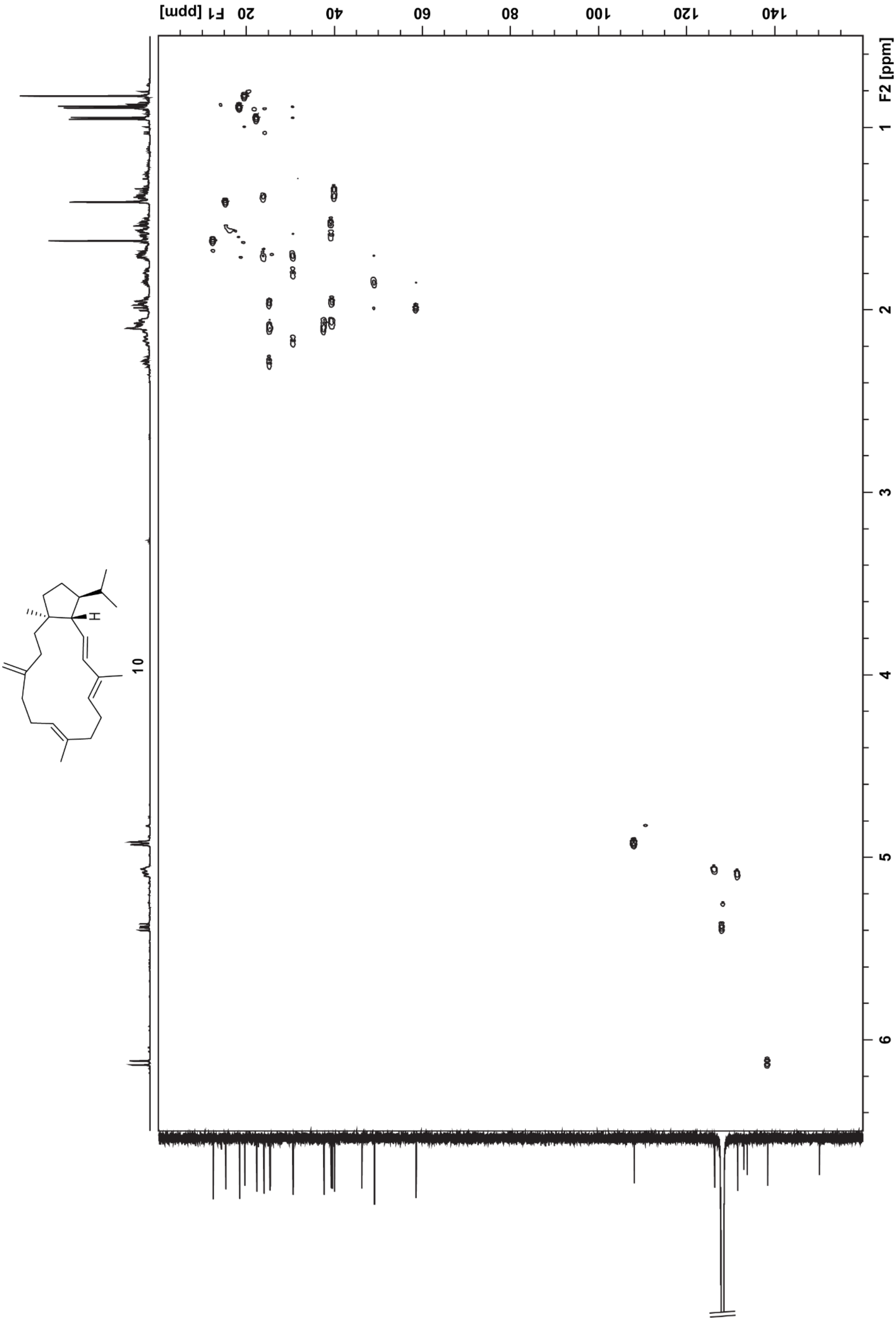

Figure S44. HSQC spectrum (C_6D_6) of **10**.

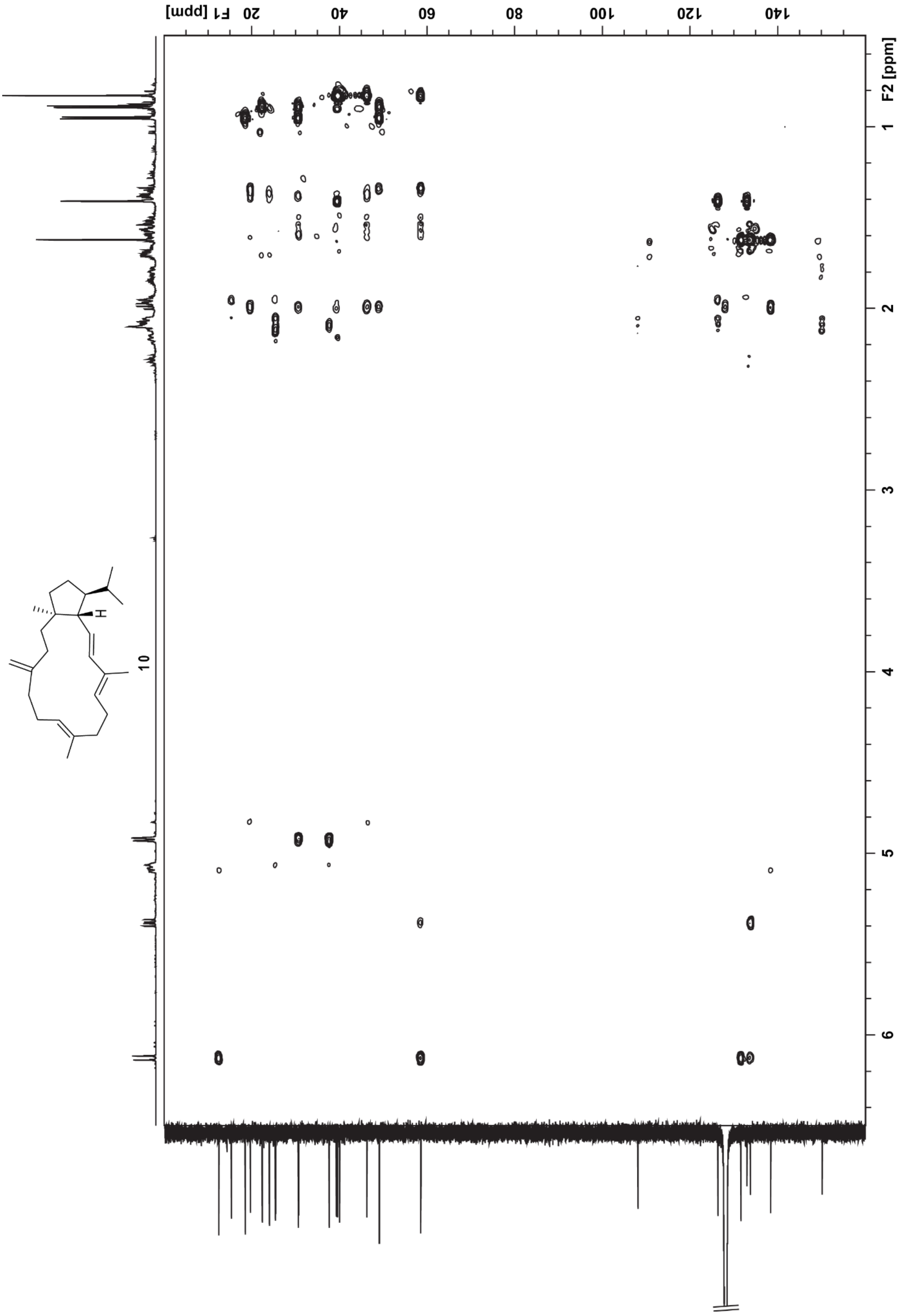

Figure S45. HMBC spectrum (C_6D_6) of **10**.

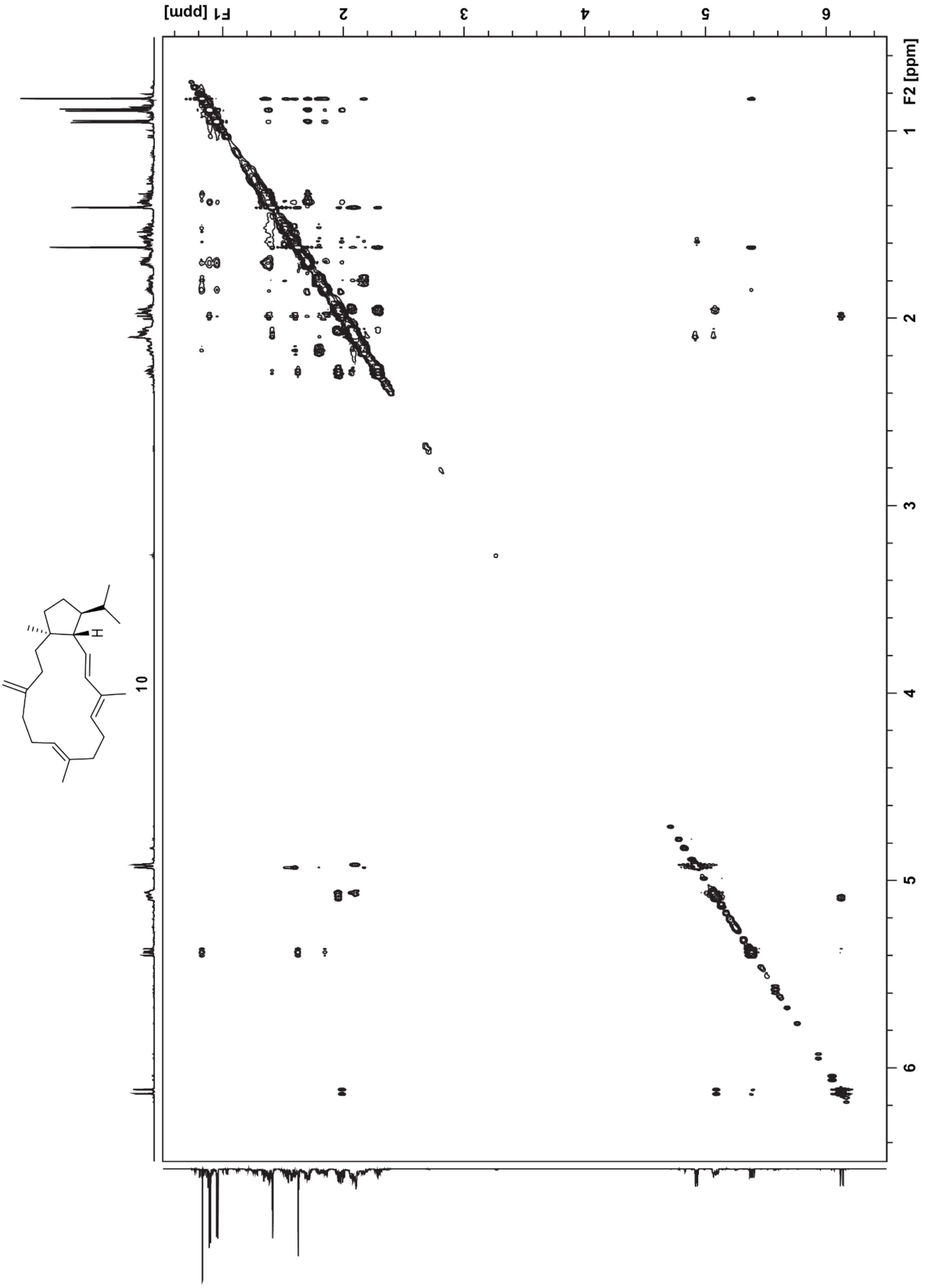

Figure S46. NOESY spectrum (C_6D_6) of **10**.

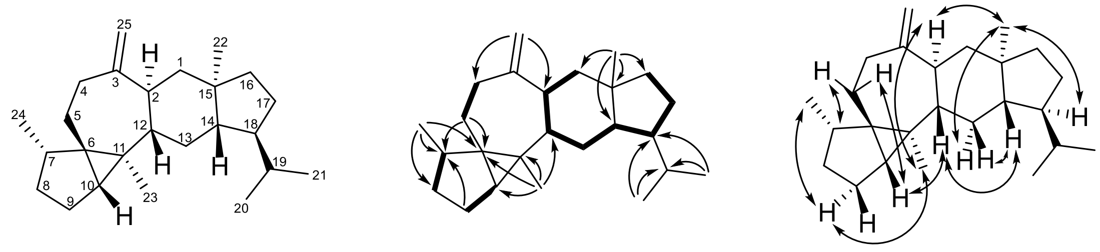

Figure S47. Structure elucidation of **11**. Bold: $^{1}H,^{1}H$-COSY correlations, single-headed arrows: key HMBC correlations, and double-headed arrows: key NOESY correlations.

Table S8. NMR data of sestermobaraene F (**11**) in C_6D_6 recorded at 298 K.

C[a]		^{13}C[b]	^{1}H[b]
1	CH_2	47.20	1.67 (m, 1H, H_α)
			1.30 (m, 1H, H_β)
2	CH	45.99	2.71 (td, *J* = 12.2, 3.7, 1H)
3	C_q	153.51	–
4	CH_2	31.30	2.38 (m, 2H)
5	CH_2	33.74	1.99 (m, 1H, H_β)
			1.92 (m, 1H, H_α)
6	C_q	39.05	–
7	CH	45.42	2.20 (m, 1H)
8	CH_2	35.51	1.86 (m, 1H, H_β)
			1.14 (m, 1H, H_α)
9	CH_2	25.92	1.96 (m, 1H, H_β)
			1.72 (m, 1H, H_α)
10	CH	37.64	0.62 (d, *J* = 5.7, 1H)
11	C_q	30.64	–
12	CH	50.82	0.98 (m, 1H)
13	CH_2	27.21	1.61 (m, 1H, H_β)
			1.25 (m, 1H, H_α)
14	CH	51.90	1.04 (m, 1H)
15	C_q	42.62	–
16	CH_2	39.43	1.43 (m, 1H, H_α)
			1.10 (m, 1H, H_β)
17	CH_2	23.68	1.68 (m, 1H, H_α)
			1.36 (m, 1H, H_β)
18	CH	46.29	1.55 (m, 1H)
19	CH	29.52	1.69 (m, 1H)
20	CH_3	22.43	0.95 (d, *J* = 6.9, 3H)
21	CH_3	17.97	0.83 (d, *J* = 6.8, 3H)
22	CH_3	19.25	0.85 (s, 3H)
23	CH_3	11.55	1.11 (s, 3H)
24	CH_3	16.53	1.14 (d, *J* = 7.1, 3H)
25	CH_2	112.68	4.91 (d, *J* = 2.6, 1H, H_Z)[c]
			4.82 (m, 1H, H_E)[c]

[a] Carbon numbering as shown in Figure S47. [b] Chemical shifts δ in ppm, multiplicity: s = singlet, d = doublet, t = triplet, m = multiplet, coupling constants *J* are given in Hertz. [c] Assignment according to CIP priority rules.

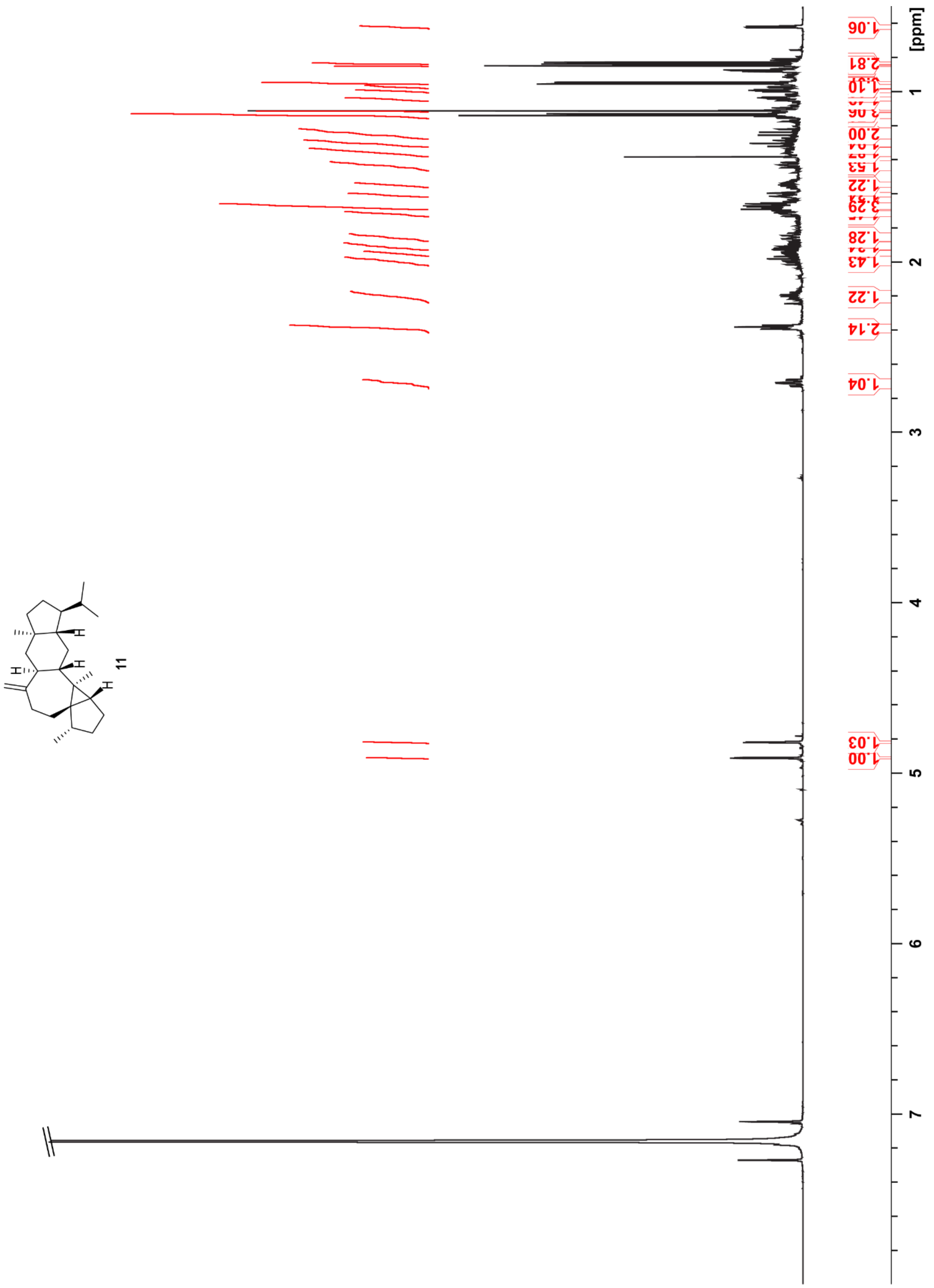

Figure S48. ^{1}H-NMR spectrum (700 MHz, C_6D_6) of **11**.

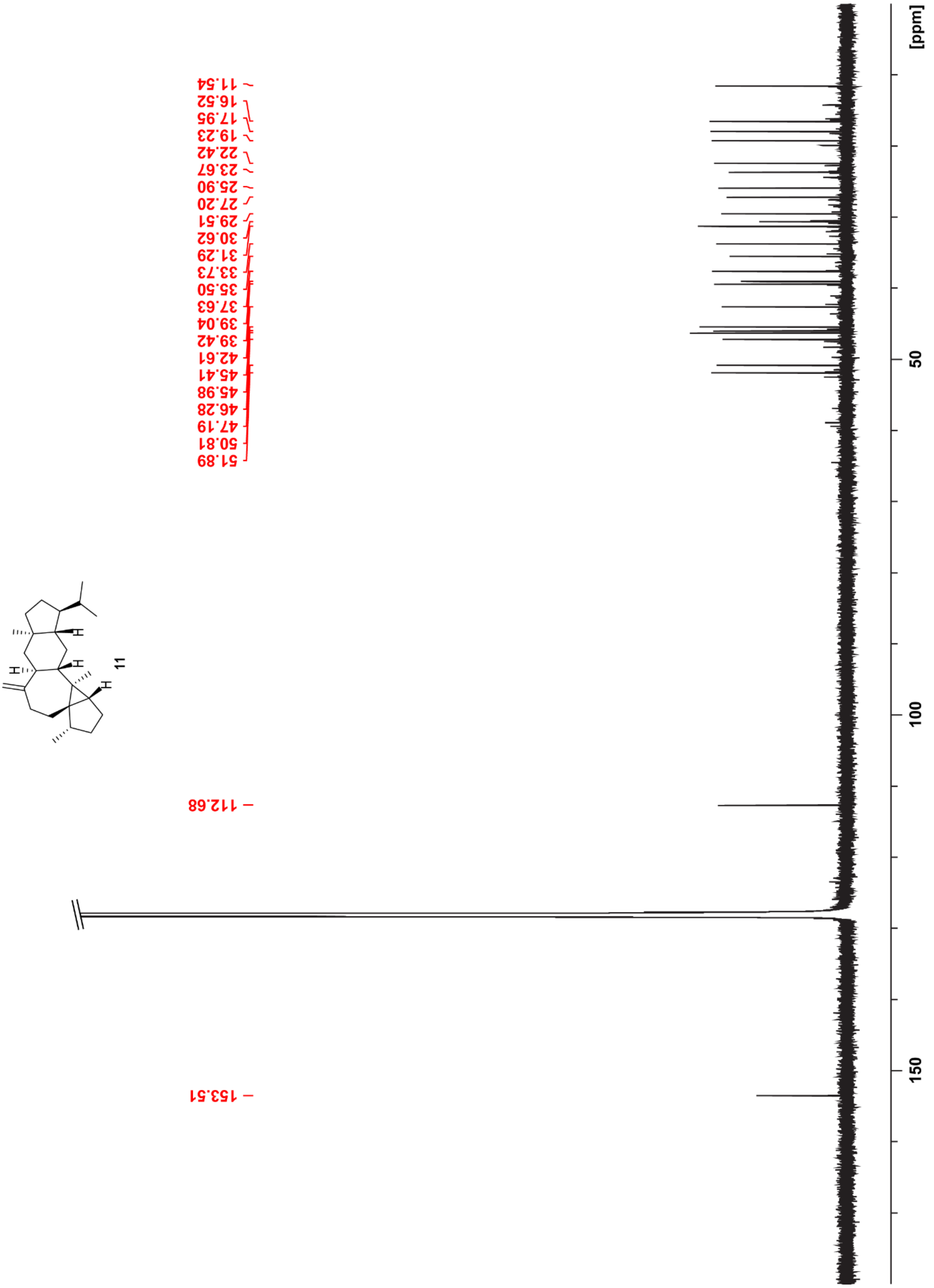

Figure S49. ^{13}C-NMR spectrum (176 MHz, C_6D_6) of **11**.

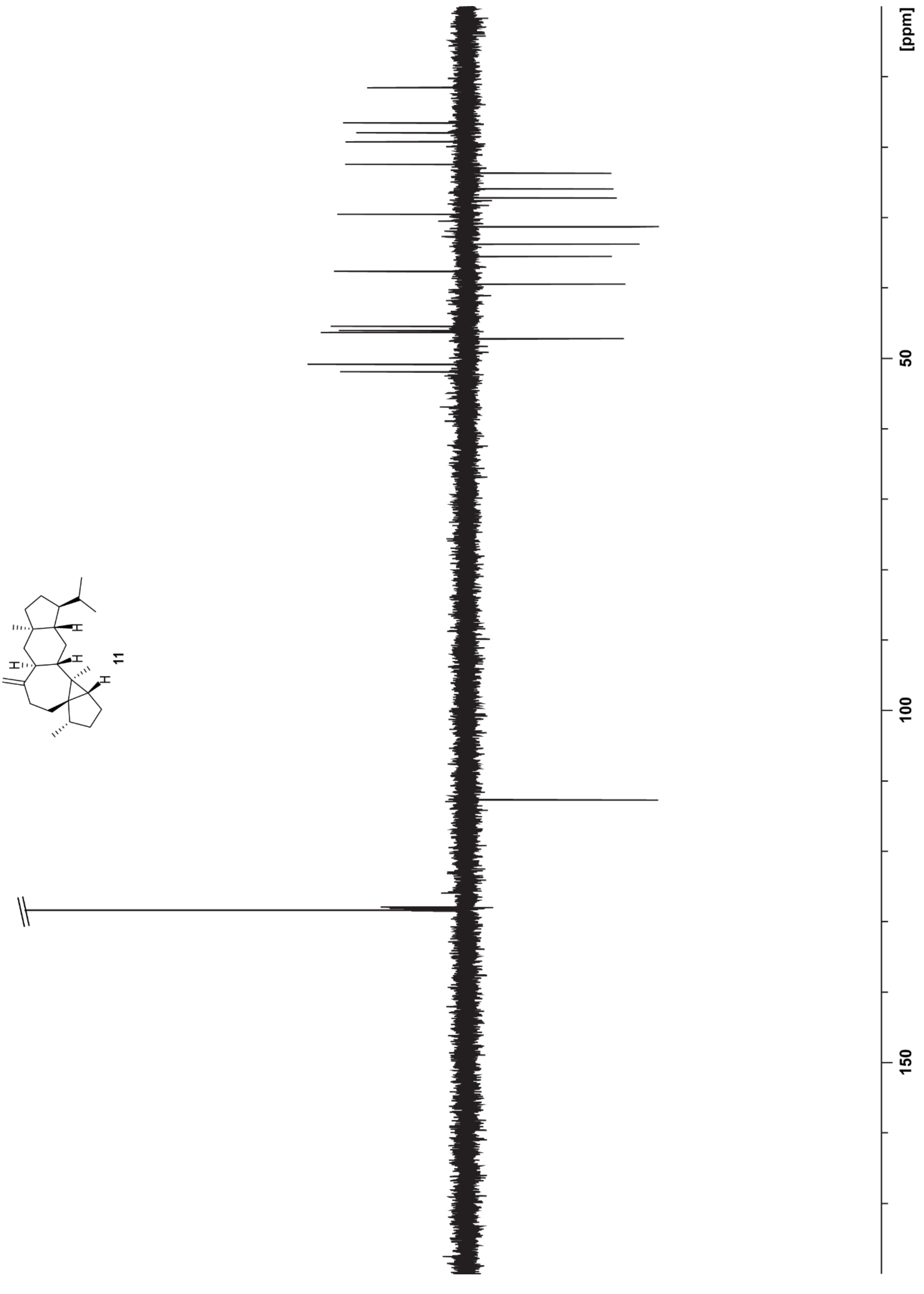

Figure S50. ^{13}C-DEPT135 spectrum (176 MHz, C_6D_6) of **11**.

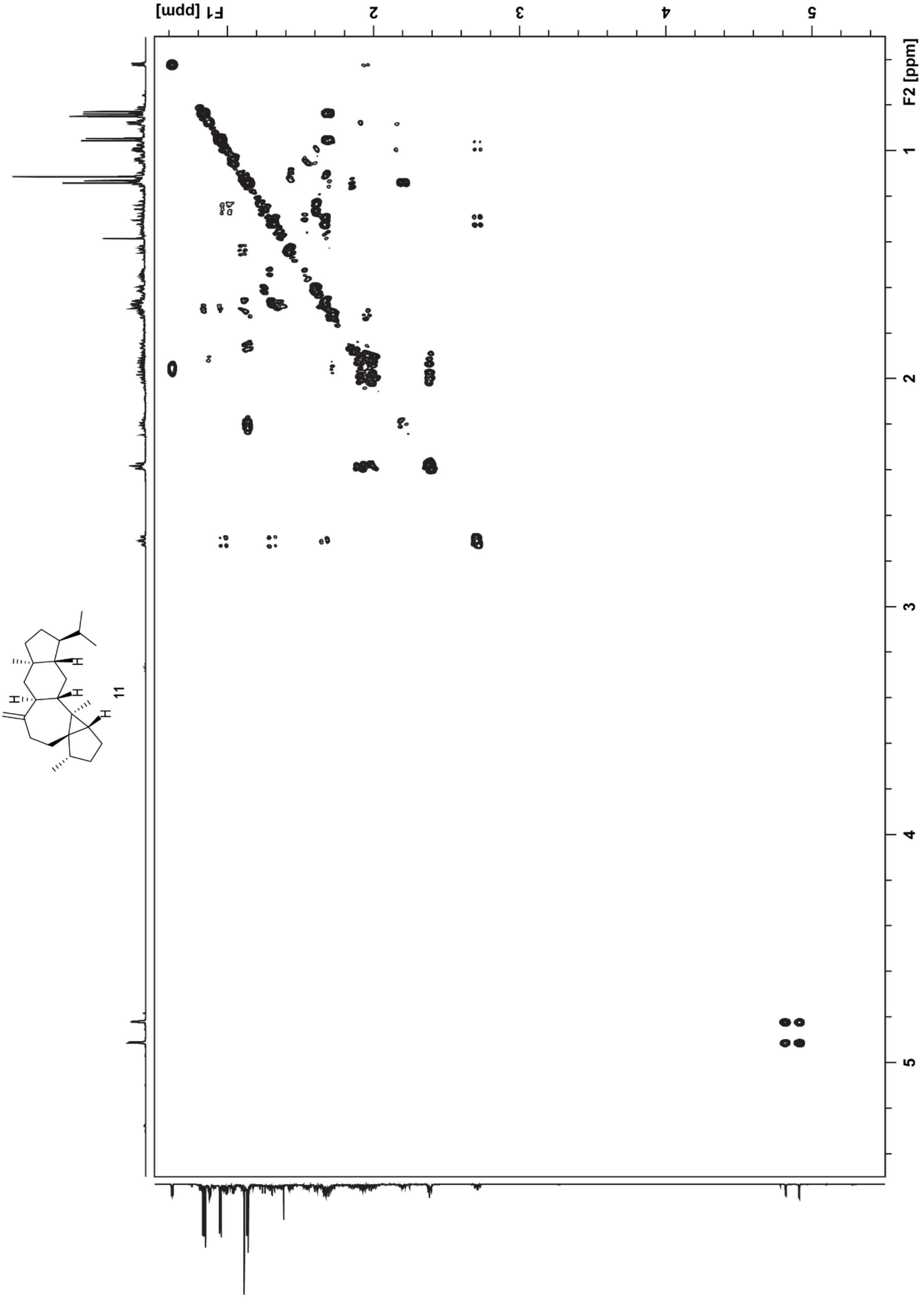

Figure S51. ^{1}H,^{1}H-COSY spectrum (C_6D_6) of **11**.

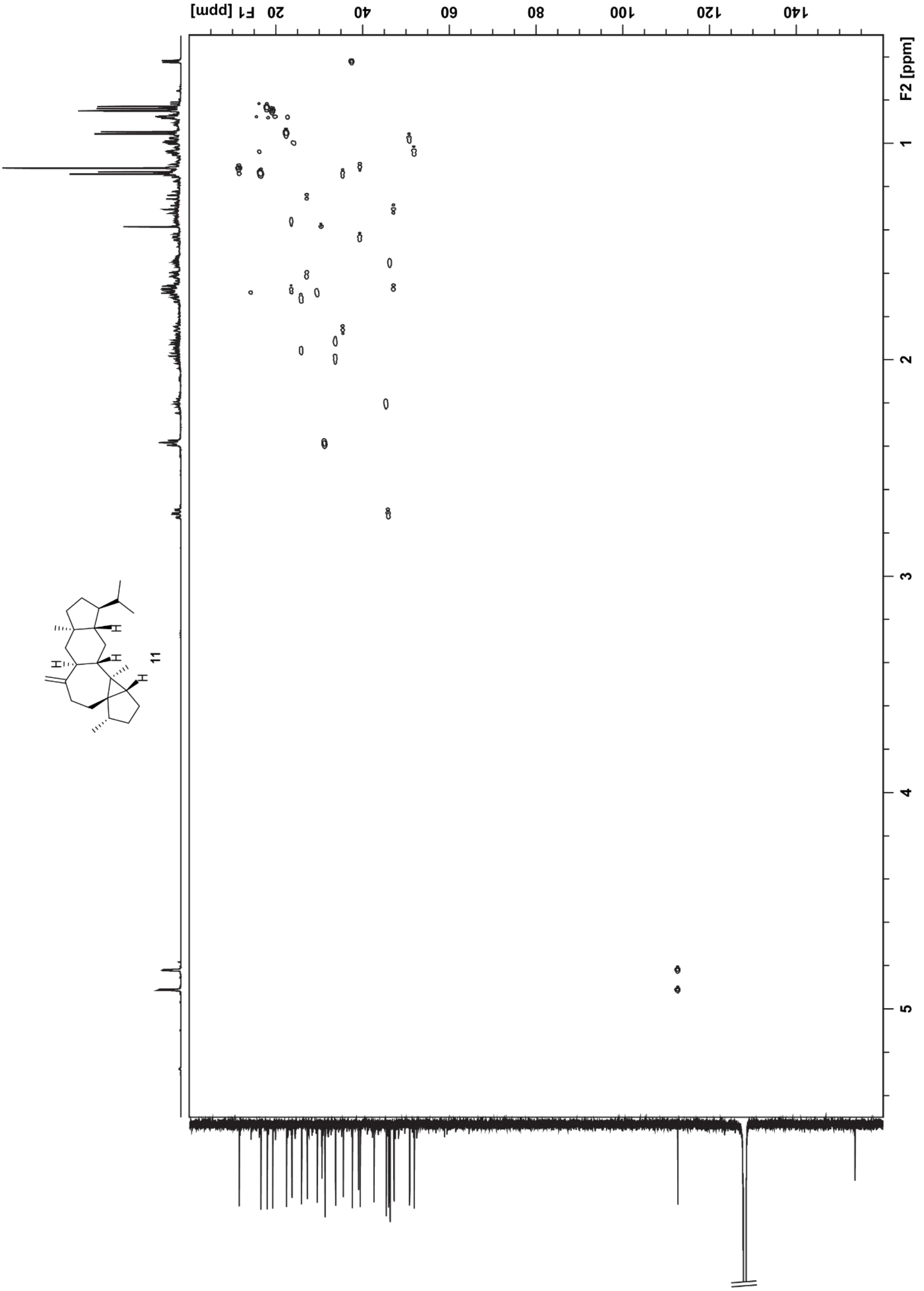

Figure S52. HSQC spectrum (C_6D_6) of **11**.

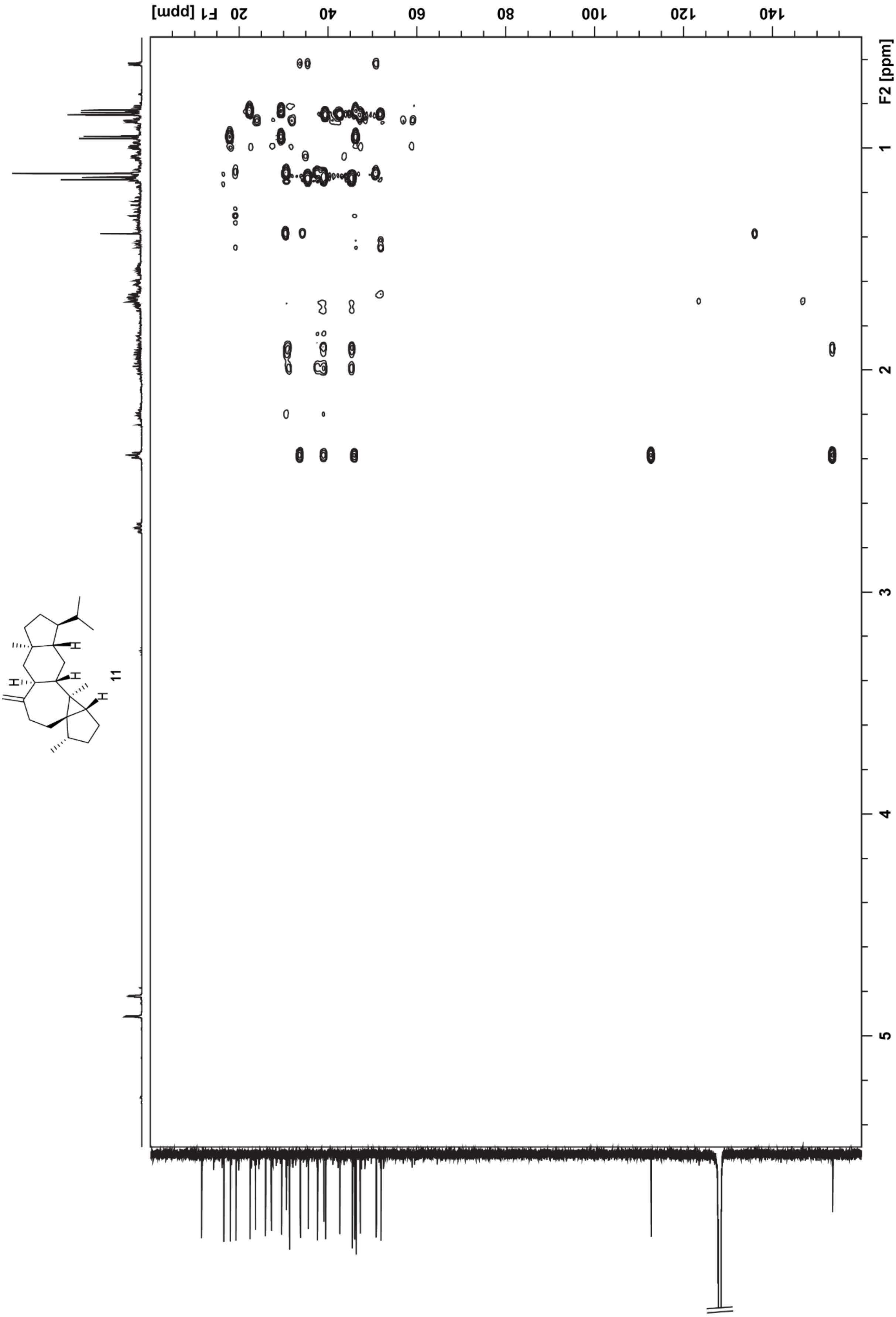

Figure S53. HMBC spectrum (C_6D_6) of **11**.

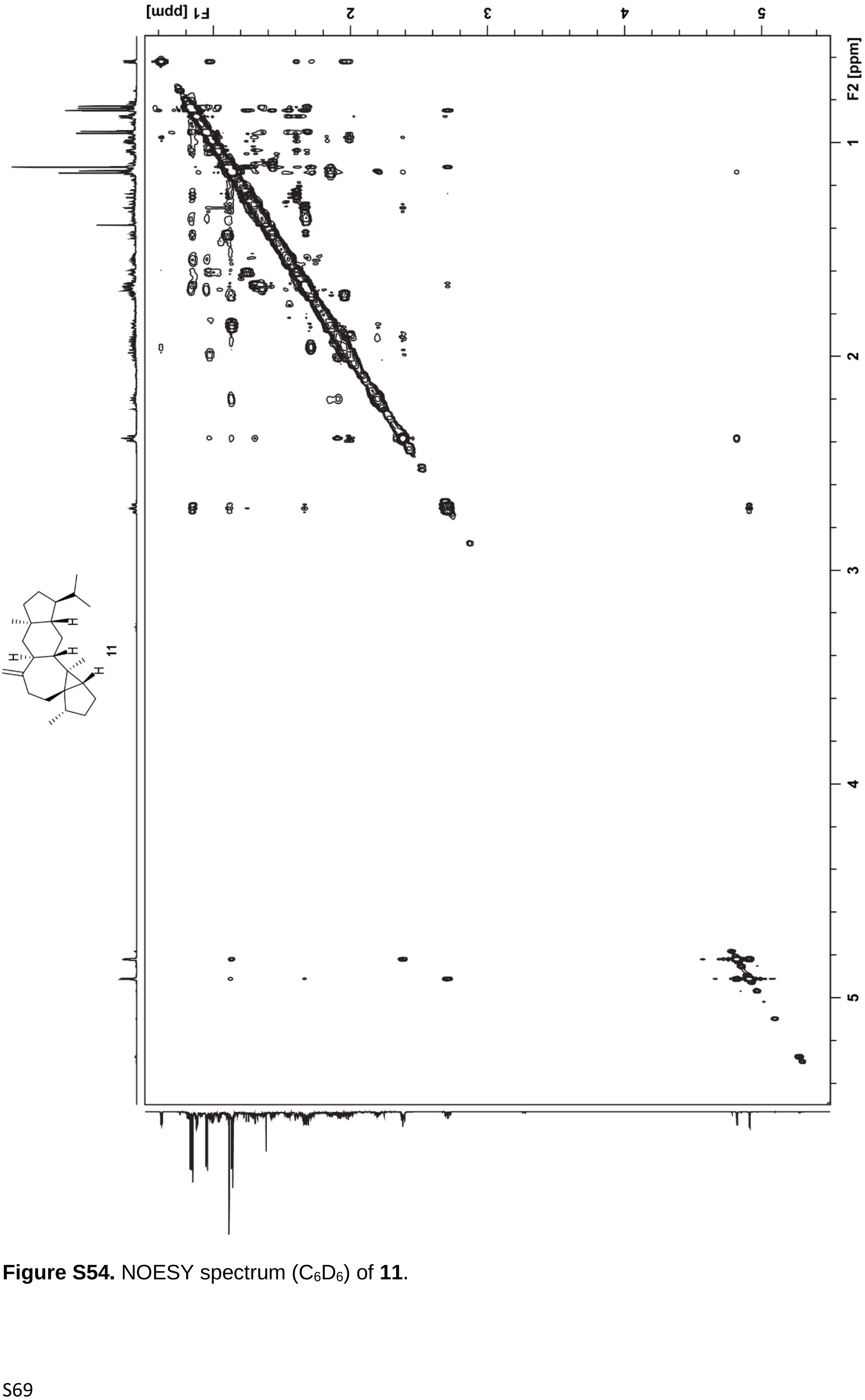

Figure S54. NOESY spectrum (C_6D_6) of **11**.

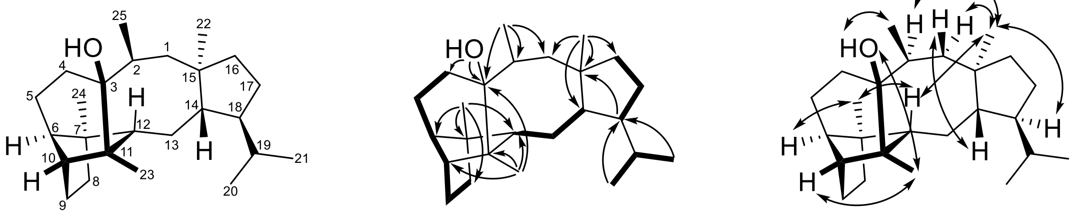

Figure S55 Structure elucidation of **12**. Bold: $^{1}H,^{1}H$-COSY correlations, single-headed arrows: key HMBC correlations, and double-headed arrows: key NOESY correlations.

Table S9. NMR data of sestermobaraol (**12**) in C_6D_6 recorded at 298 K.

C[a]		^{13}C[b]	^{1}H[b]
1	CH_2	45.68	1.71 (dd, J = 15.7, 9.9, 1H, H_β)
			1.21 (d, J = 15.6, 1H, H_α)
2	CH	34.58	2.02 (dq, J = 10.0, 6.8, 1H)
3	C_q	79.59	–
4	CH_2	35.57	1.77 (m, 1H, H_α)
			1.40 (m, 1H, H_β)
5	CH_2	17.42	1.43 (m, 1H, H_β)
			1.09 (m, 1H, H_α)
6	CH	49.05	1.51 (m, 1H)
7	C_q	49.33	–
8	CH_2	29.89	1.62 (m, 1H, H_β)[c]
			1.06 (m, 1H, H_α)[c]
9	CH_2	22.28	1.54 (m, 1H, H_β)[c]
			1.33 (m, 1H, H_α)[c]
10	CH	46.94	1.95 (dd, J = 4.8, 1.8, 1H)
11	C_q	48.56	–
12	CH	41.24	1.51 (m, 1H)
13	CH_2	26.13	1.64 (m, 1H, H_β)
			0.94 (m, 1H, H_α)
14	CH	46.28	1.63 (m, 1H)
15	C_q	45.35	–
16	CH_2	45.81	1.30 (m, 2H)
17	CH_2	21.29	1.55 (m, 1H, H_α)
			1.30 (m, 1H, H_β)
18	CH	53.62	1.65 (m, 1H)
19	CH	28.93	1.76 (m, 1H)
20	CH_3	22.55	0.93 (d, J = 6.9, 3H)
21	CH_3	16.41	0.83 (d, J = 6.8, 3H)
22	CH_3	17.84	0.87 (s, 3H)
23	CH_3	17.84	0.89 (s, 3H)
24	CH_3	17.92	1.12 (s, 3H)
25	CH_3	21.87	0.97 (d, J = 6.7, 3H)

[a] Carbon numbering as shown in Figure S55. [b] Chemical shifts δ in ppm, multiplicity: s = singlet, d = doublet, q = quartet, m = multiplet, coupling constants J are given in Hertz. [c] For assignment of H_α and H_β cf. Figure S107.

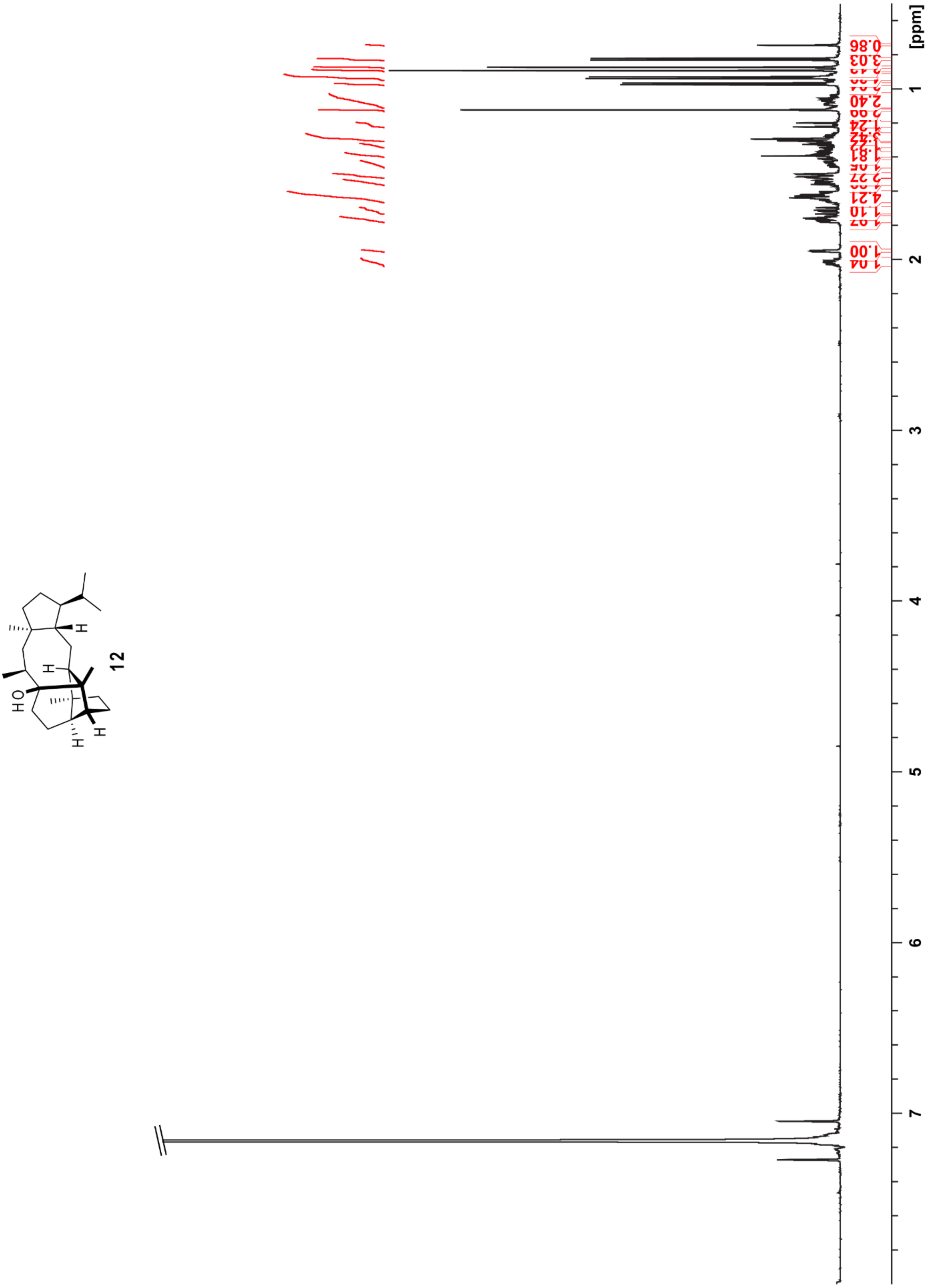

Figure S56. ^{1}H-NMR spectrum (700 MHz, C_6D_6) of **12**.

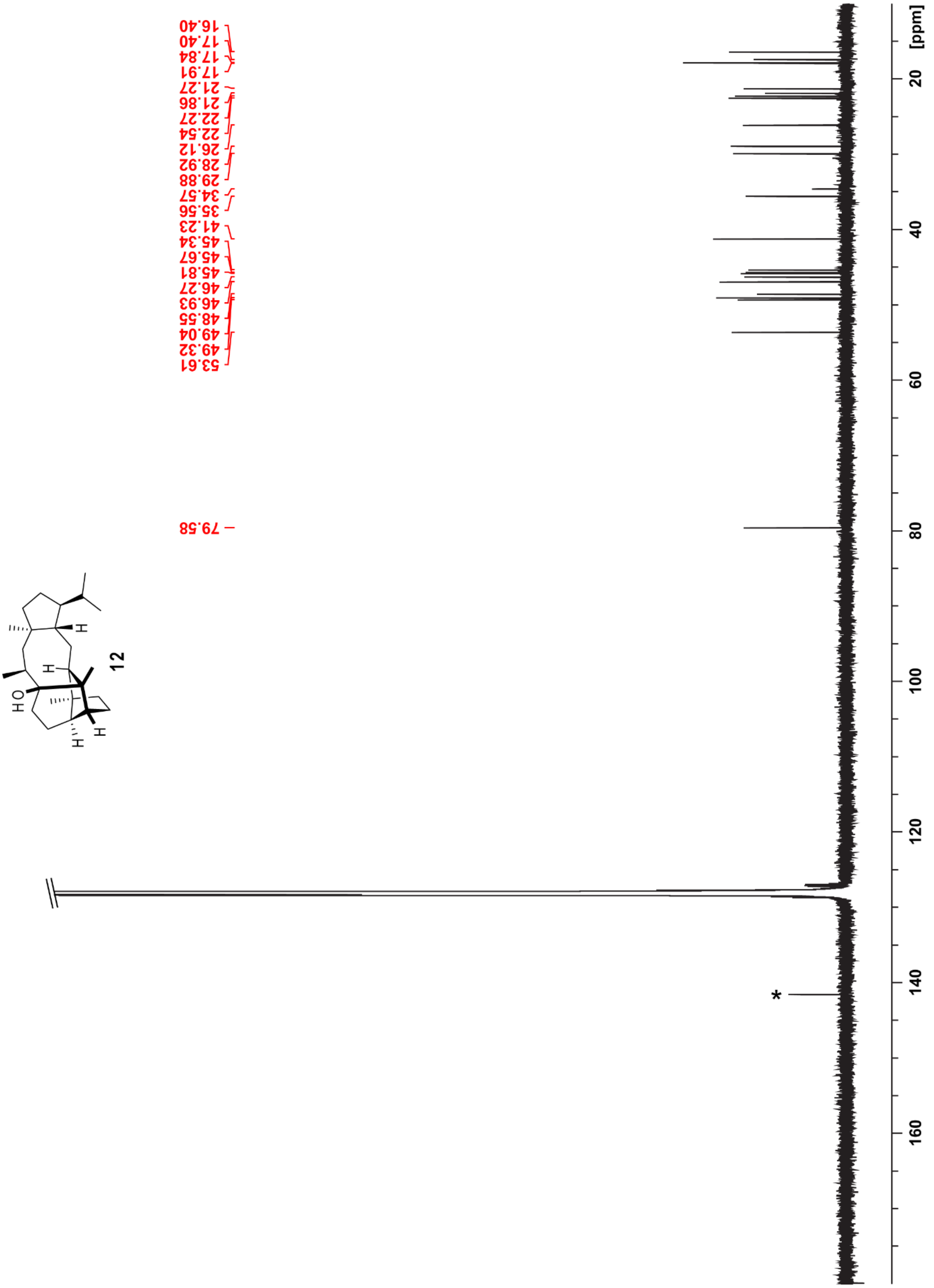

Figure S57. ^{13}C-NMR spectrum (176 MHz, C_6D_6) of **12** (asterisk indicates an impurity from commercial C_6D_6).

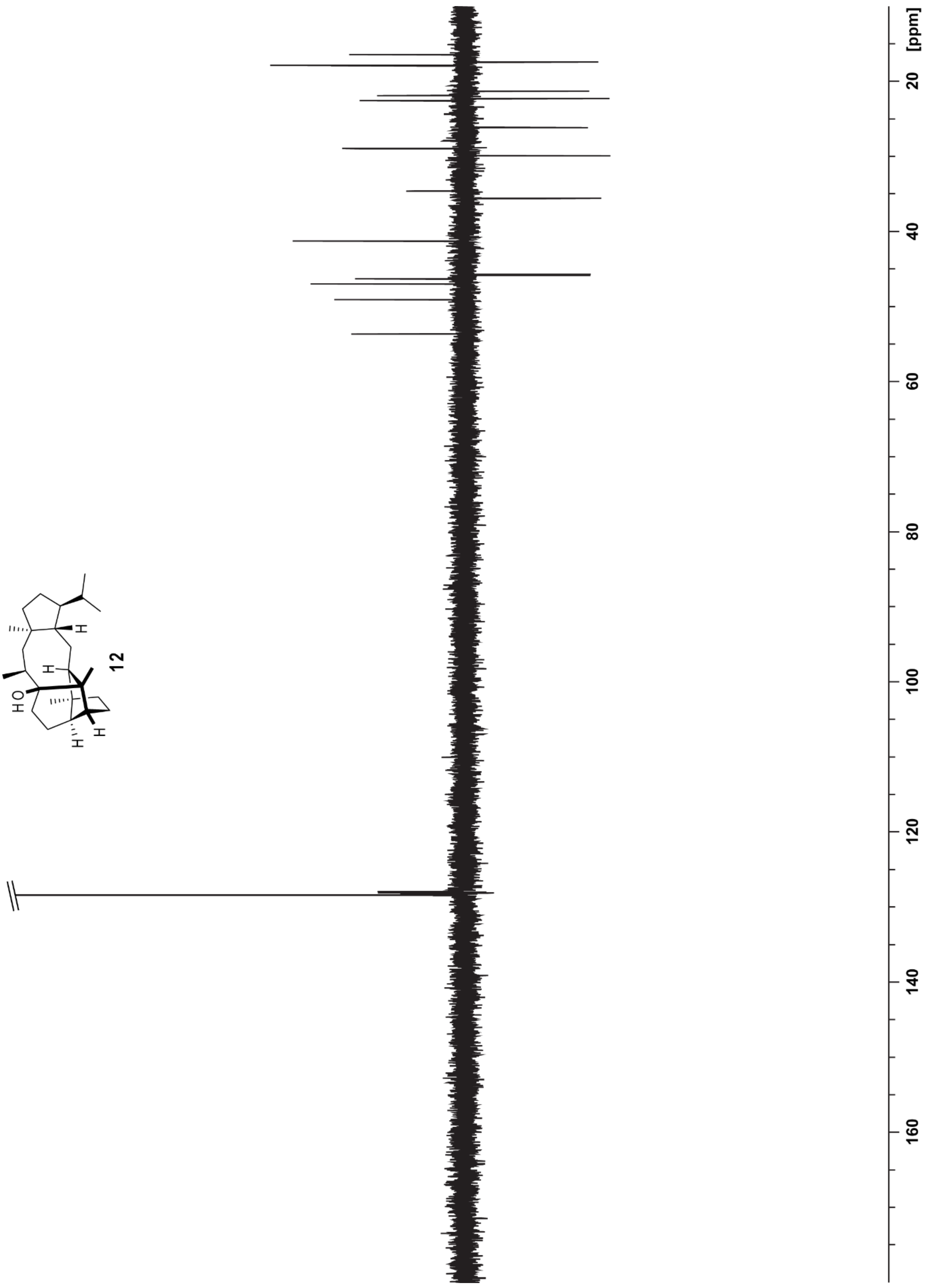

Figure S58. ^{13}C-DEPT135 spectrum (176 MHz, C_6D_6) of **12**.

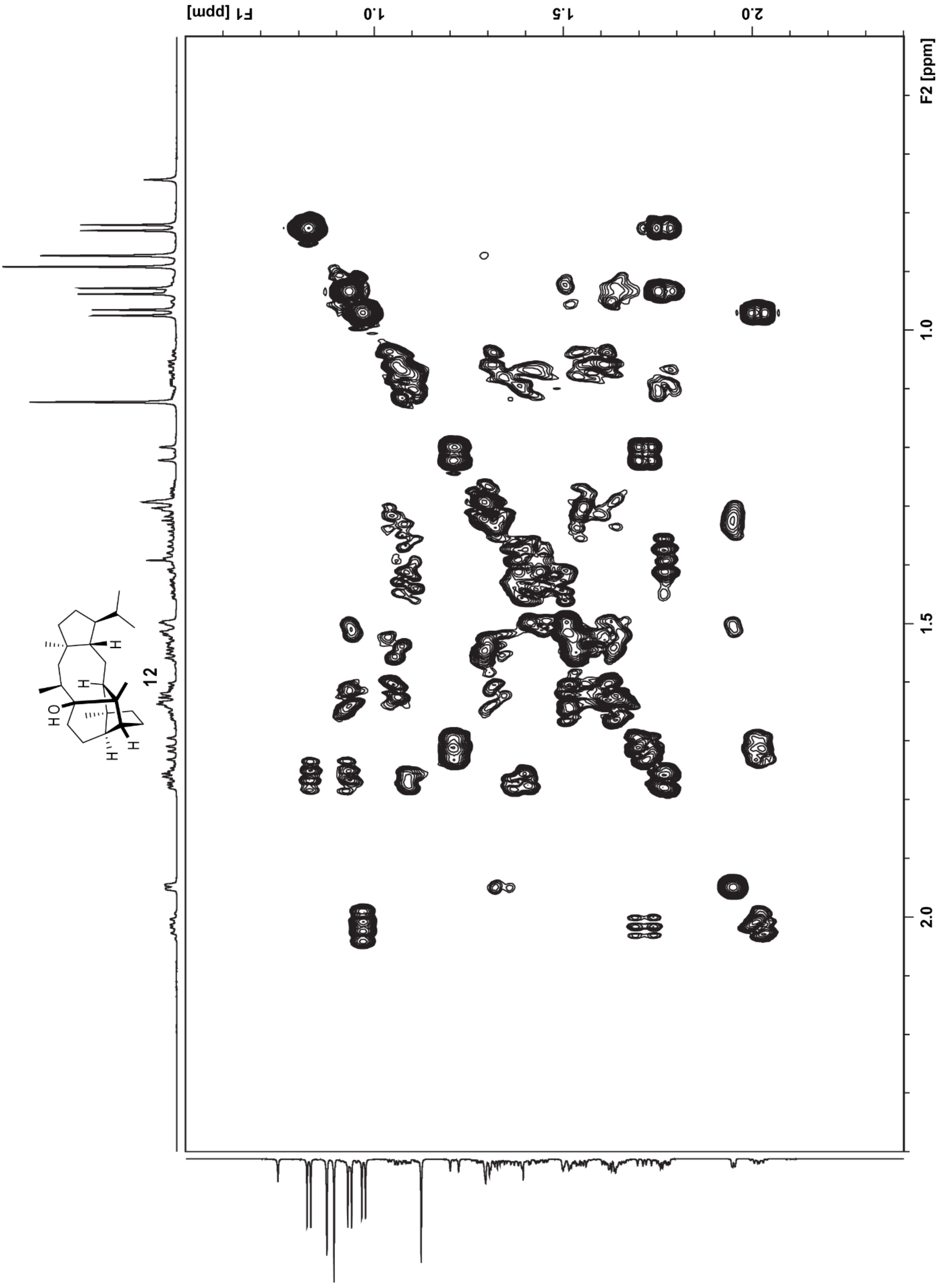

Figure S59. $^{1}H,^{1}H$-COSY spectrum (C_6D_6) of **12**.

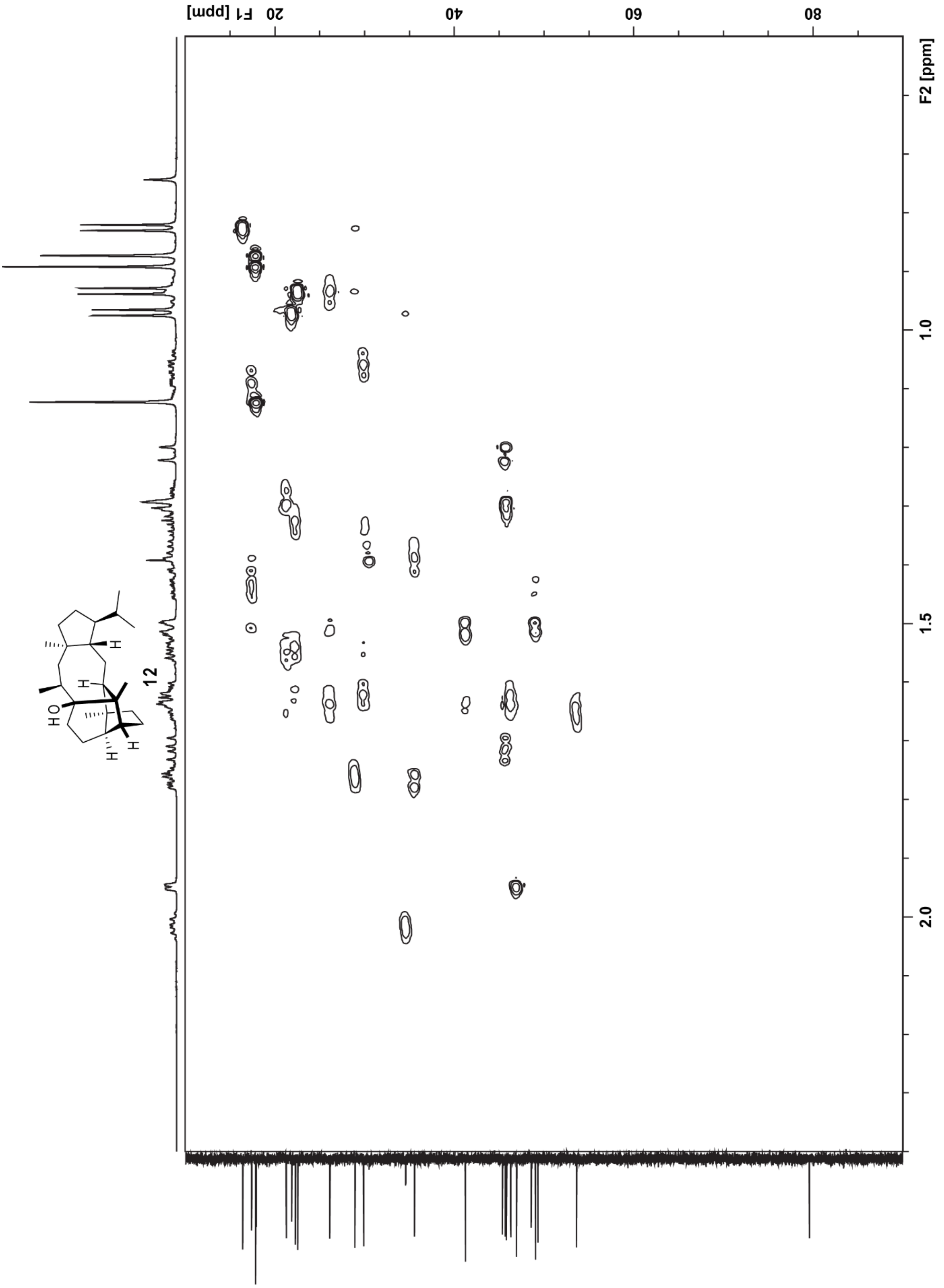

Figure S60. HSQC spectrum (C_6D_6) of **12**.

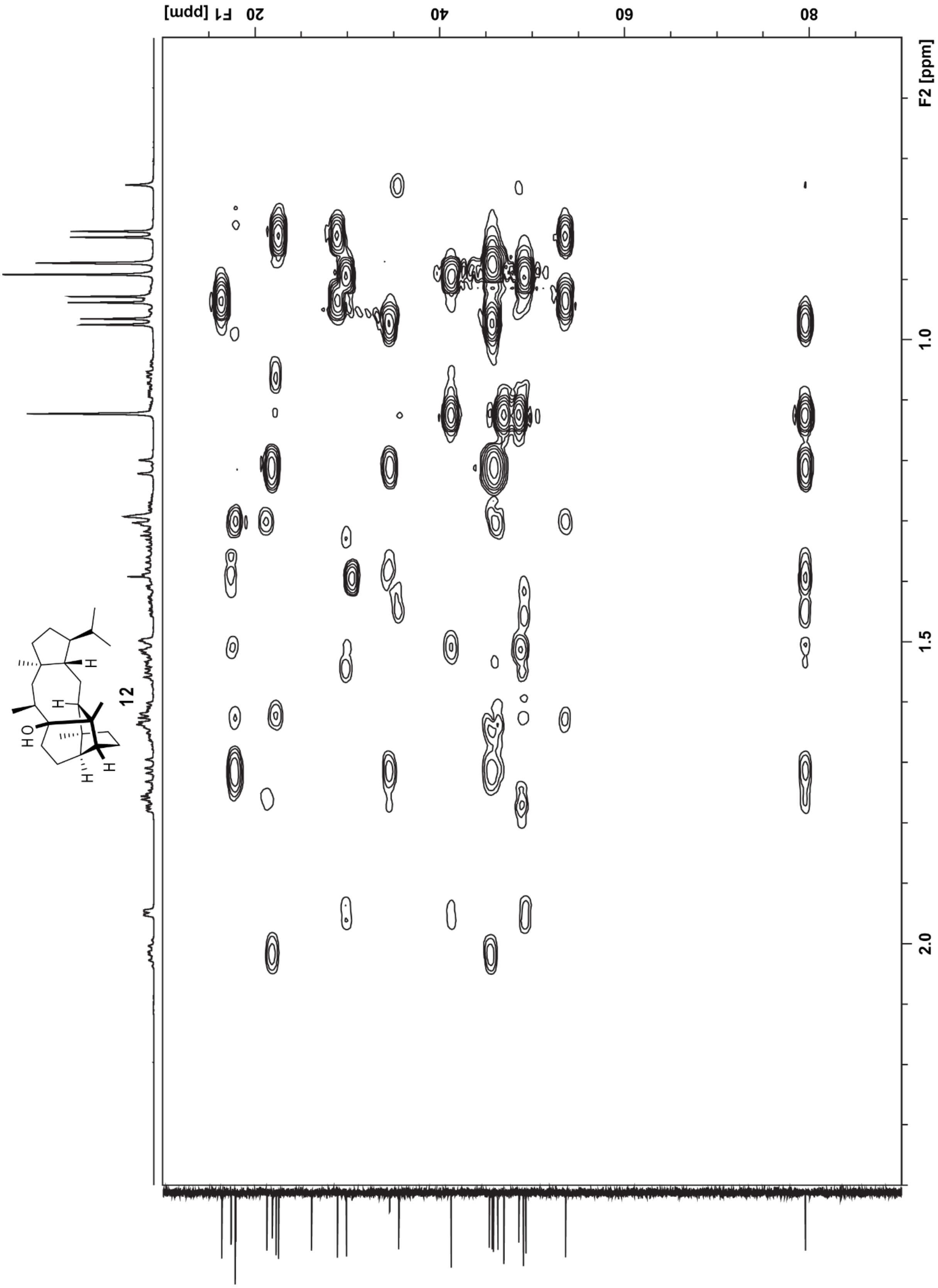

Figure S61. HMBC spectrum (C_6D_6) of **12**.

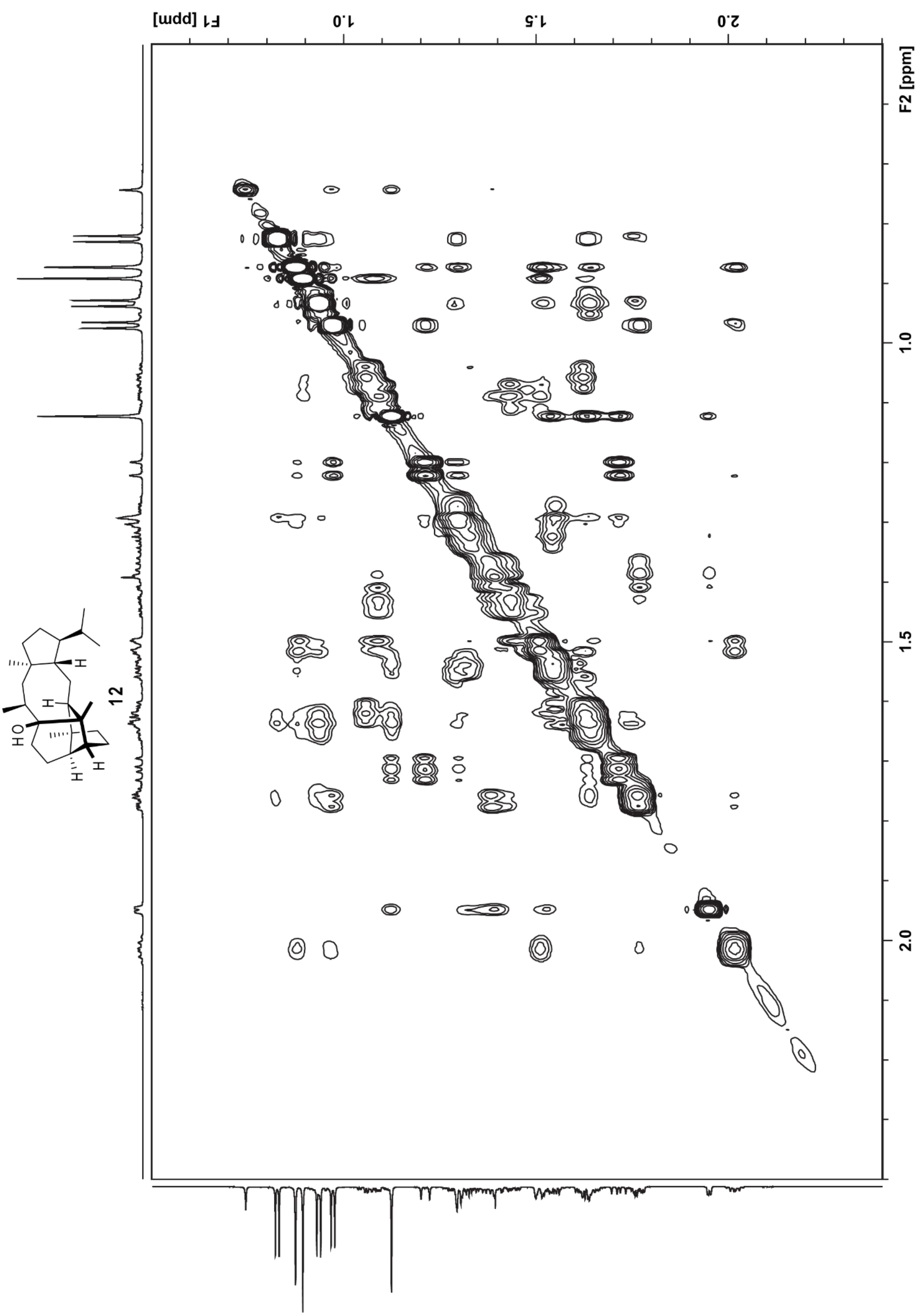

Figure S62. NOESY spectrum (C_6D_6) of **12**.

Incubation experiments with labeled substrates
Isotopic labeling experiments were performed with the precursors of GFPP (ca. 1.5 mg, in 1 mL 25 mM NH_4HCO_3), incubation buffer (5 mL), enzyme elution fractions, and the substrates and enzyme preparations as listed in Table S10. After incubation at 28 °C overnight, the products were extracted twice with C_6D_6 (600 µL and 300 µL), the extracts were dried with $MgSO_4$ and analyzed by NMR and/or GC/MS.

Table S10. Labeling experiments with SmTS1.

entry	substrates	enzymes	results shown in
1	GGPP + (1-^{13}C)IPP[10]	GFPPS, SmTS1	Figure S63
2	GGPP + (2-^{13}C)IPP[20]	GFPPS, SmTS1	Figure S64
3	GGPP + (3-^{13}C)IPP[10]	GFPPS, SmTS1	Figure S65
4	GGPP + (4-^{13}C)IPP[10]	GFPPS, SmTS1	Figure S66
5	(1-^{13}C)GGPP + IPP	GFPPS, SmTS1	Figure S67
6	(2-^{13}C)GGPP[10] + IPP	GFPPS, SmTS1	Figure S68
7	(3-^{13}C)GGPP + IPP	GFPPS, SmTS1	Figure S69
8	(4-^{13}C)GGPP + IPP	GFPPS, SmTS1	Figure S70
9	(1-^{13}C)FPP[21] + IPP	GFPPS, SmTS1	Figure S71
10	(2-^{13}C)FPP[21] + IPP	GFPPS, SmTS1	Figure S72
11	(3-^{13}C)FPP[21] + IPP	GFPPS, SmTS1	Figure S73
12	(4-^{13}C)FPP[21] + IPP	GFPPS, SmTS1	Figure S74
13	(5-^{13}C)FPP[21] + IPP	GFPPS, SmTS1	Figure S75
14	(6-^{13}C)FPP[21] + IPP	GFPPS, SmTS1	Figure S76
15	(7-^{13}C)FPP[21] + IPP	GFPPS, SmTS1	Figure S77
16	(6-^{13}C)FPP[21] + IPP	GFPPS, SmTS1	Figure S78
17	(9-^{13}C)FPP[21] + IPP	GFPPS, SmTS1	Figure S79
18	(10-^{13}C)FPP[21] + IPP	GFPPS, SmTS1	Figure S80
19	(11-^{13}C)FPP[21] + IPP	GFPPS, SmTS1	Figure S81
20	(12-^{13}C)FPP[21] + IPP	GFPPS, SmTS1	Figure S82
21	(9-^{13}C)GPP[22] + IPP	GFPPS, SmTS1	Figure S83
22	(14-^{13}C)FPP[21] + IPP	GFPPS, SmTS1	Figure S84
23	(15-^{13}C)FPP[21] + IPP	GFPPS, SmTS1	Figure S85
24	(20-^{13}C)GGPP[10] + IPP	GFPPS, SmTS1	Figure S86
25	GGPP + (5-^{13}C)IPP (Scheme S6)	GFPPS, SmTS1	Figure S87
26	(7-^{13}C)GPP[23] + (*E*)-(4-^{13}C,4-^{2}H)IPP[24]	GFPPS, SmTS1	Figures S88 – S94
27	(7-^{13}C)GPP[23] + (*Z*)-(4-^{13}C,4-^{2}H)IPP[24]	GFPPS, SmTS1	Figures S88 – S94
28	(*R*)-(1-^{13}C,1-^{2}H)GPP[10] + (2-^{13}C)IPP[20]	GFPPS, SmTS1	Figures S95, S96
29	(*S*)-(1-^{13}C,1-^{2}H)GPP[10] + (2-^{13}C)IPP[20]	GFPPS, SmTS1	Figures S95, S96
30	(3-^{13}C,2-^{2}H)GGPP[10] + IPP	GFPPS, SmTS1	Figure S97
31	GPP + (*Z*)-(4-^{2}H)IPP[25] + (2-^{13}C)IPP[20]	FPPS,[26] GFPPS, SmTS1	Figure S98
32	GPP + (3-^{13}C,4-$^{2}H_2$)IPP (Scheme S7)	GFPPS, SmTS1	Figures S99, S100
33	GPP + (*R*)-(1-^{13}C,1-^{2}H)IPP[2]	GFPPS, SmTS1	Figures S101 – S107
34	GPP + (*S*)-(1-^{13}C,1-^{2}H)IPP[2]	GFPPS, SmTS1	Figures S101 – S107
35	GPP + (*E*)-(4-^{13}C,4-^{2}H)IPP[24]	GFPPS, SmTS1	Figures S101 – S107
36	GPP + (*Z*)-(4-^{13}C,4-^{2}H)IPP[24]	GFPPS, SmTS1	Figures S101 – S107

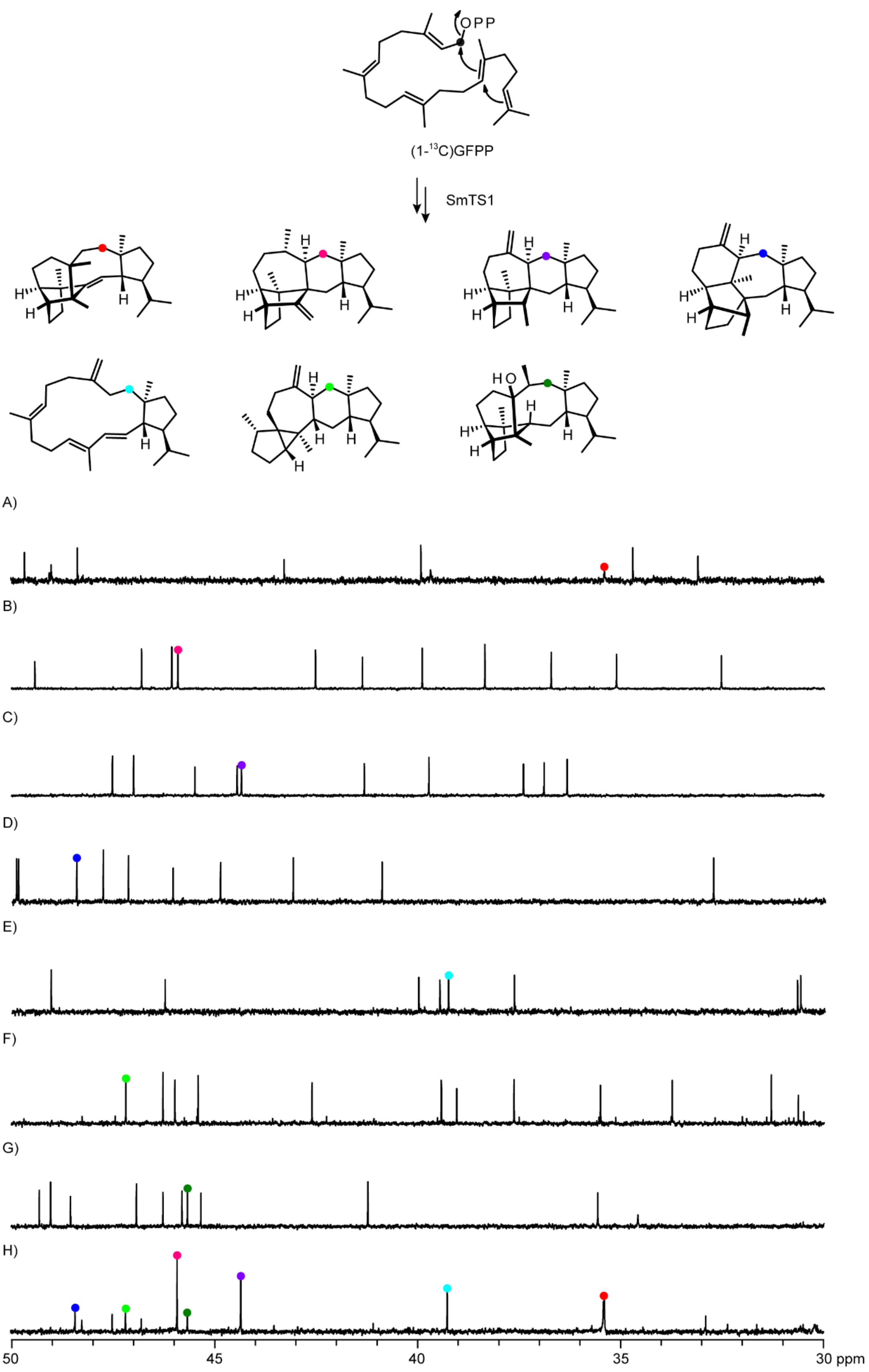

Figure S63. Enzymatic conversion of (1-^{13}C)GFPP with SmTS1. Coloured dots indicate labeled carbons and the corresponding peaks in the ^{13}C-NMR spectra. Figures A) – G) show the ^{13}C-NMR spectra of unlabeled **6** – **12**, H) shows the ^{13}C-NMR spectrum of the enzyme products from (1-^{13}C)GFPP.

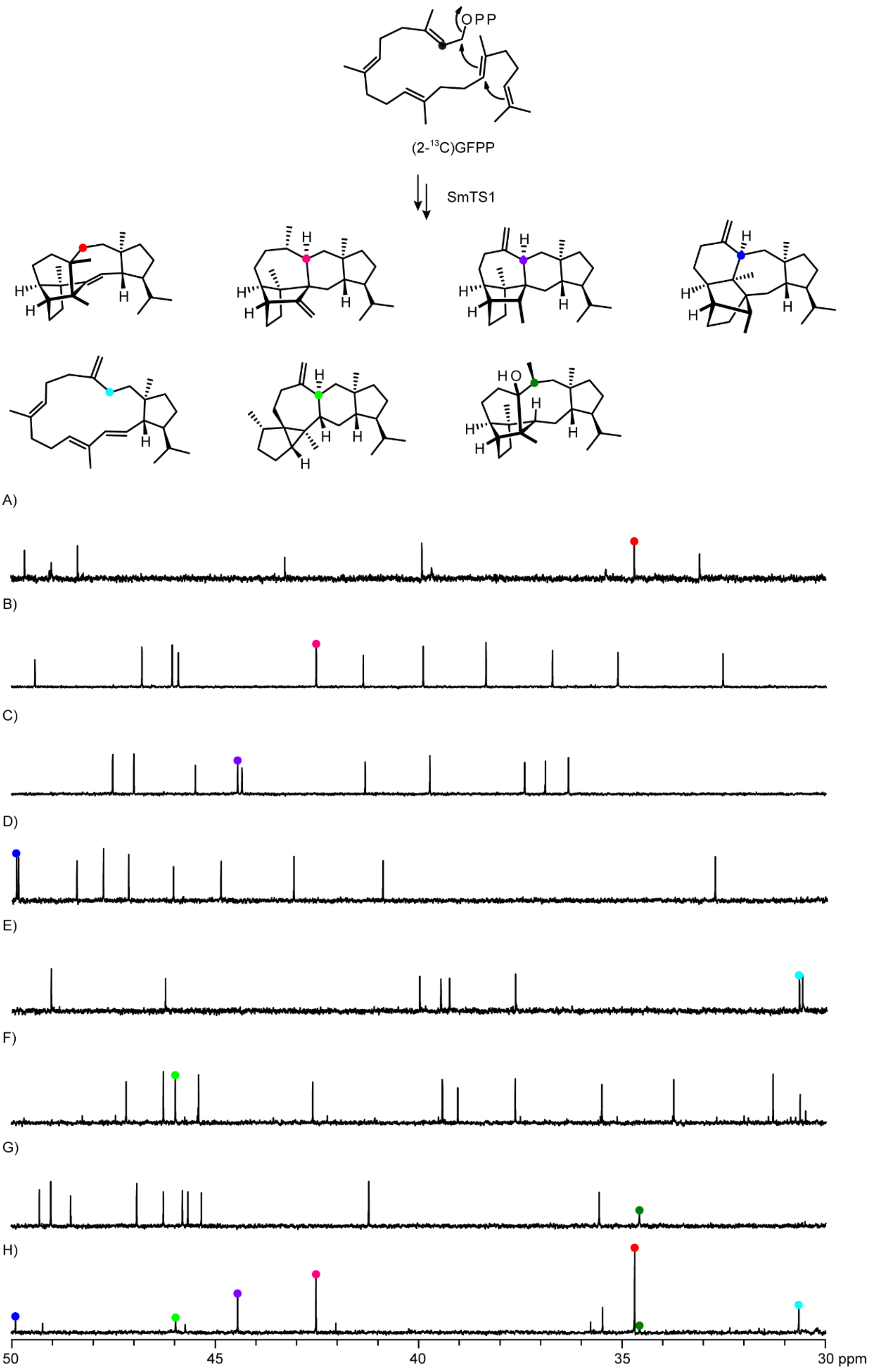

Figure S64. Enzymatic conversion of (2-^{13}C)GFPP with SmTS1. Coloured dots indicate labeled carbons and the corresponding peaks in the ^{13}C-NMR spectra. Figures A) – G) show the ^{13}C-NMR spectra of unlabeled **6** – **12**, H) shows the ^{13}C-NMR spectrum of the enzyme products from (2-^{13}C)GFPP.

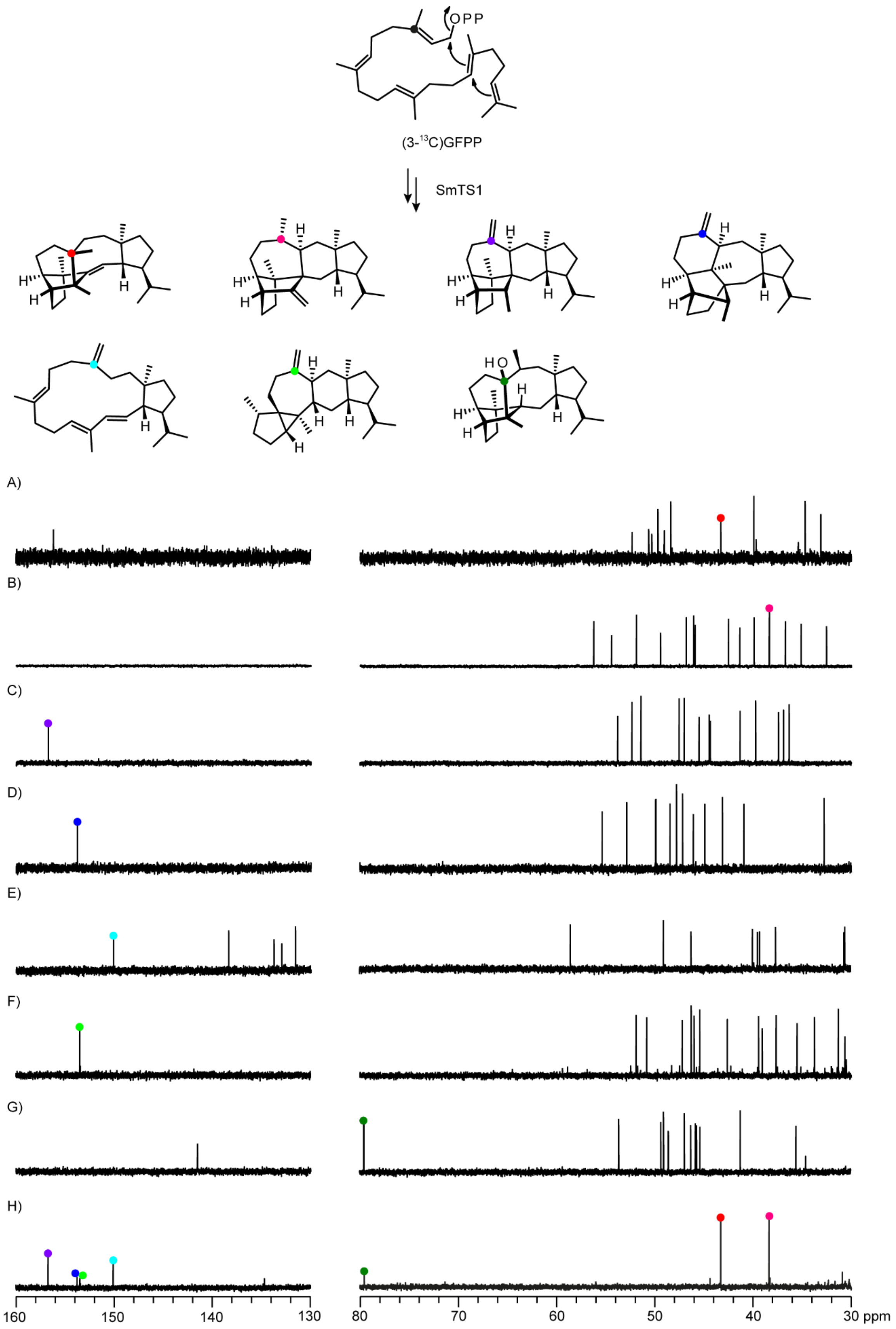

Figure S65. Enzymatic conversion of (3-^{13}C)GFPP with SmTS1. Coloured dots indicate labeled carbons and the corresponding peaks in the ^{13}C-NMR spectra. Figures A) – G) show the ^{13}C-NMR spectra of unlabeled **6** – **12**, H) shows the ^{13}C-NMR spectrum of the enzyme products from (3-^{13}C)GFPP.

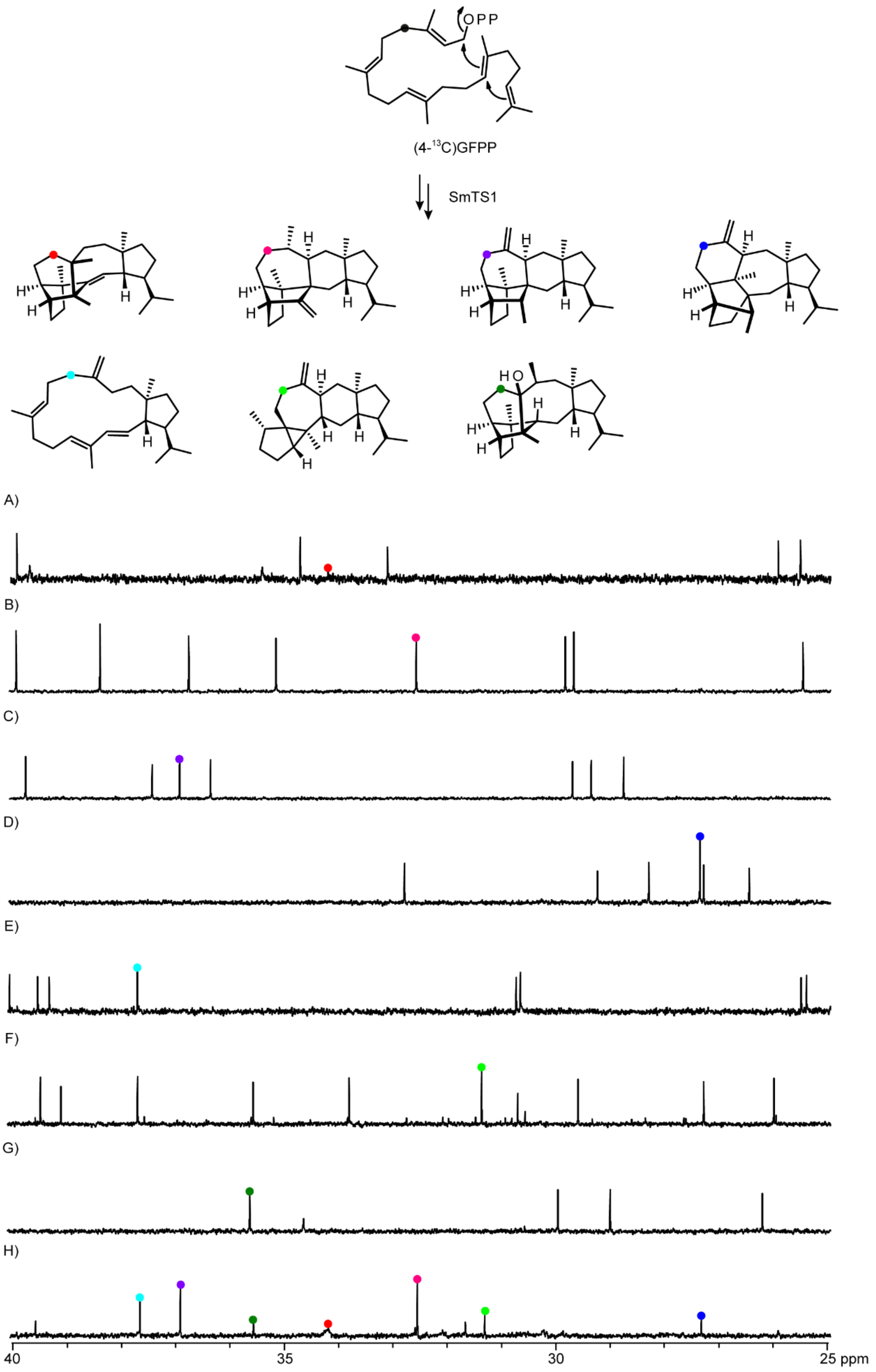

Figure S66. Enzymatic conversion of (4-^{13}C)GFPP with SmTS1. Coloured dots indicate labeled carbons and the corresponding peaks in the ^{13}C-NMR spectra. Figures A) – G) show the ^{13}C-NMR spectra of unlabeled **6** – **12**, H) shows the ^{13}C-NMR spectrum of the enzyme products from (4-^{13}C)GFPP.

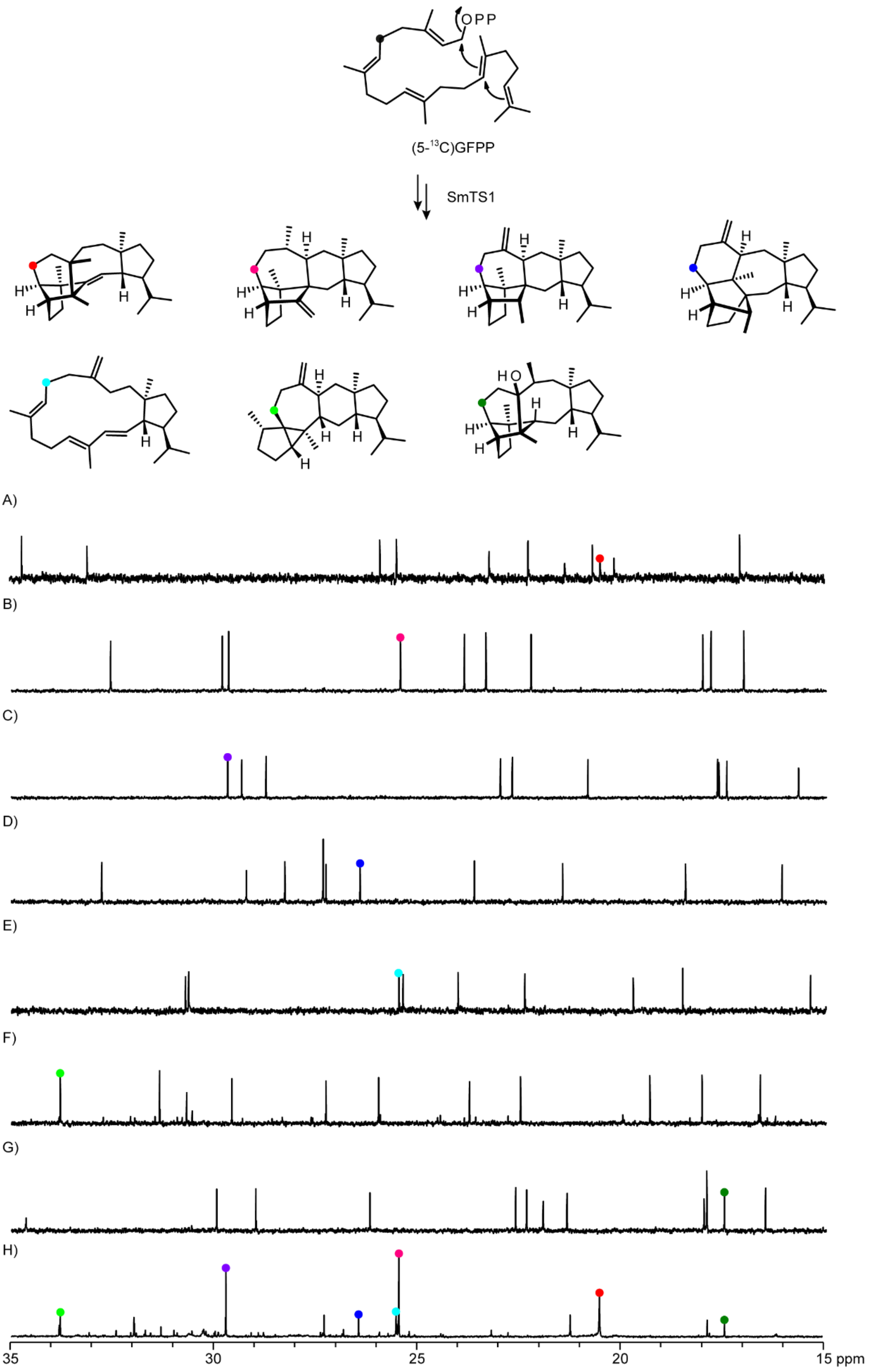

Figure S67. Enzymatic conversion of (5-^{13}C)GFPP with SmTS1. Coloured dots indicate labeled carbons and the corresponding peaks in the ^{13}C-NMR spectra. Figures A) – G) show the ^{13}C-NMR spectra of unlabeled **6** – **12**, H) shows the ^{13}C-NMR spectrum of the enzyme products from (5-^{13}C)GFPP.

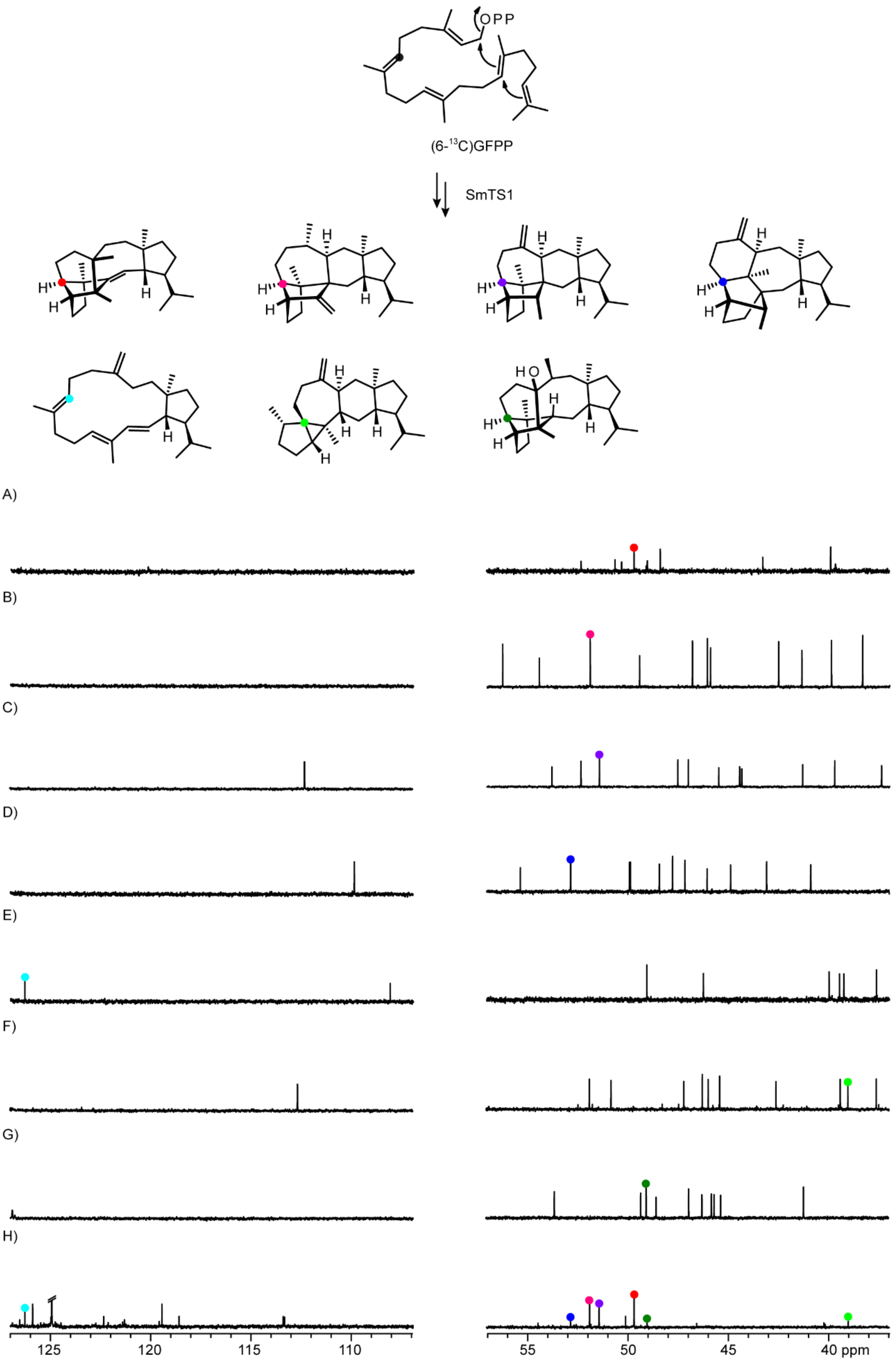

Figure S68. Enzymatic conversion of (6-^{13}C)GFPP with SmTS1. Coloured dots indicate labeled carbons and the corresponding peaks in the ^{13}C-NMR spectra. Figures A) – G) show the ^{13}C-NMR spectra of unlabeled **6** – **12**, H) shows the ^{13}C-NMR spectrum of the enzyme products from (6-^{13}C)GFPP.

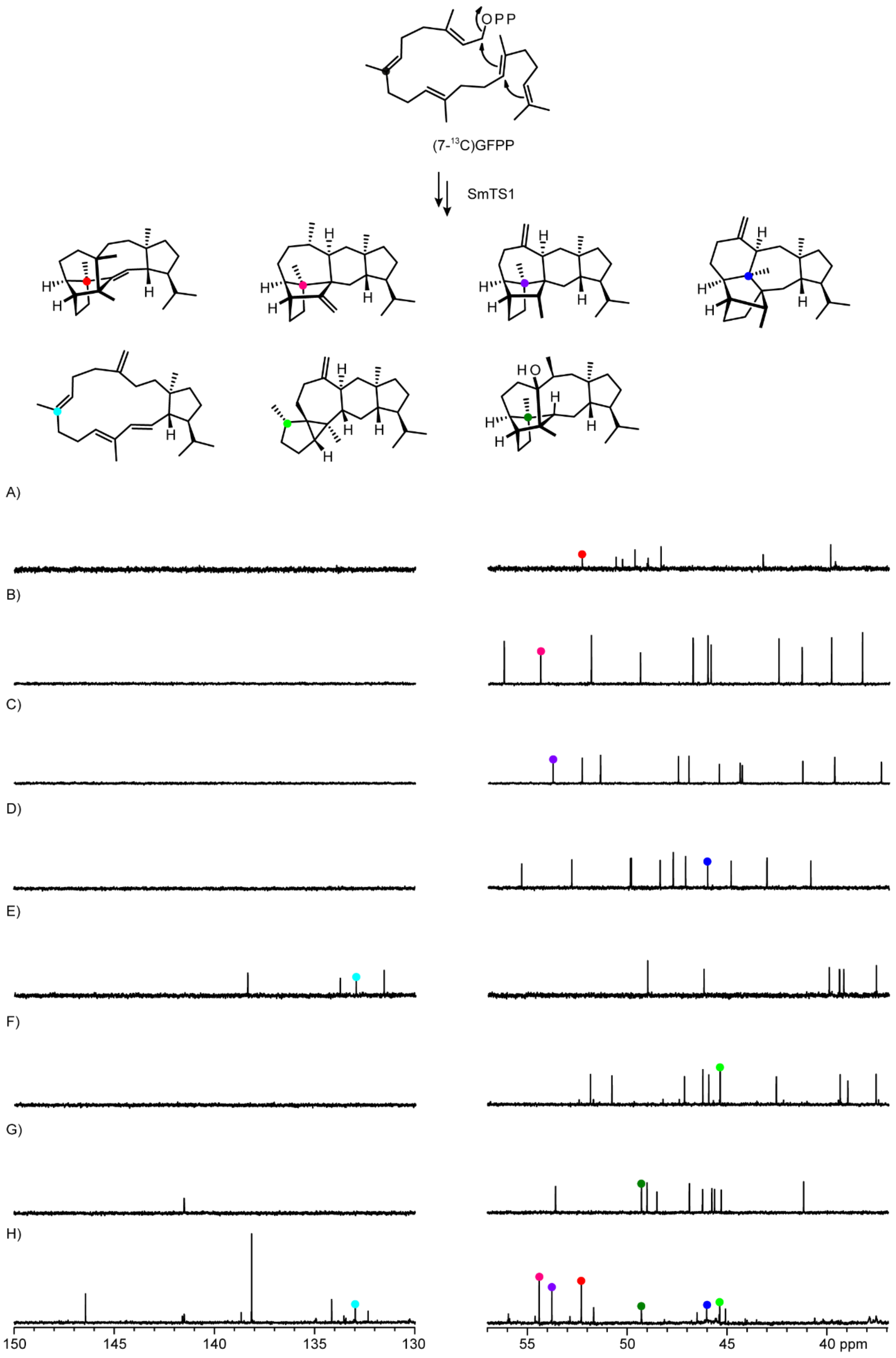

Figure S69. Enzymatic conversion of (7-^{13}C)GFPP with SmTS1. Coloured dots indicate labeled carbons and the corresponding peaks in the ^{13}C-NMR spectra. Figures A) – G) show the ^{13}C-NMR spectra of unlabeled **6** – **12**, H) shows the ^{13}C-NMR spectrum of the enzyme products from (7-^{13}C)GFPP.

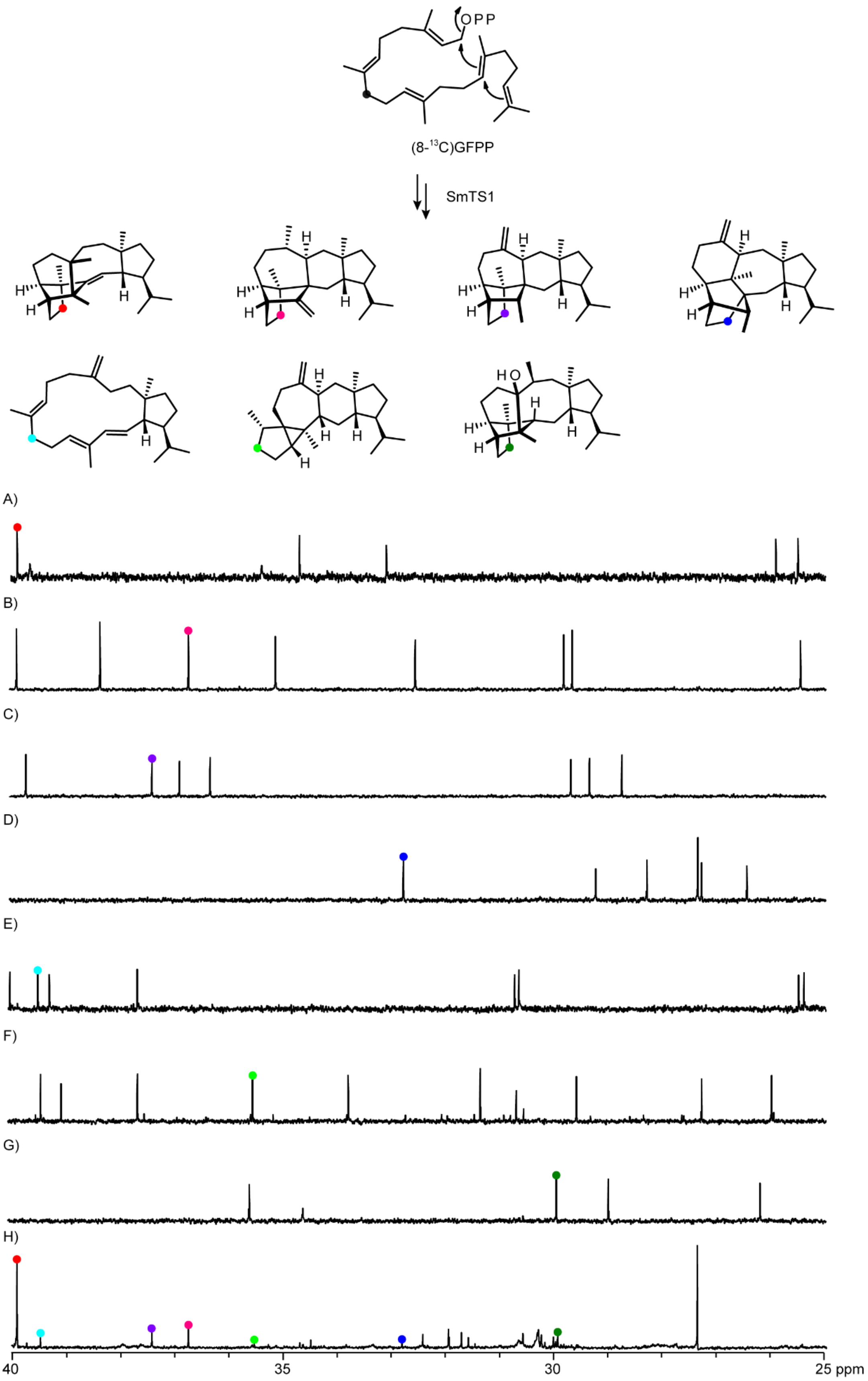

Figure S70. Enzymatic conversion of (8-^{13}C)GFPP with SmTS1. Coloured dots indicate labeled carbons and the corresponding peaks in the ^{13}C-NMR spectra. Figures A) – G) show the ^{13}C-NMR spectra of unlabeled **6** – **12**, H) shows the ^{13}C-NMR spectrum of the enzyme products from (8-^{13}C)GFPP.

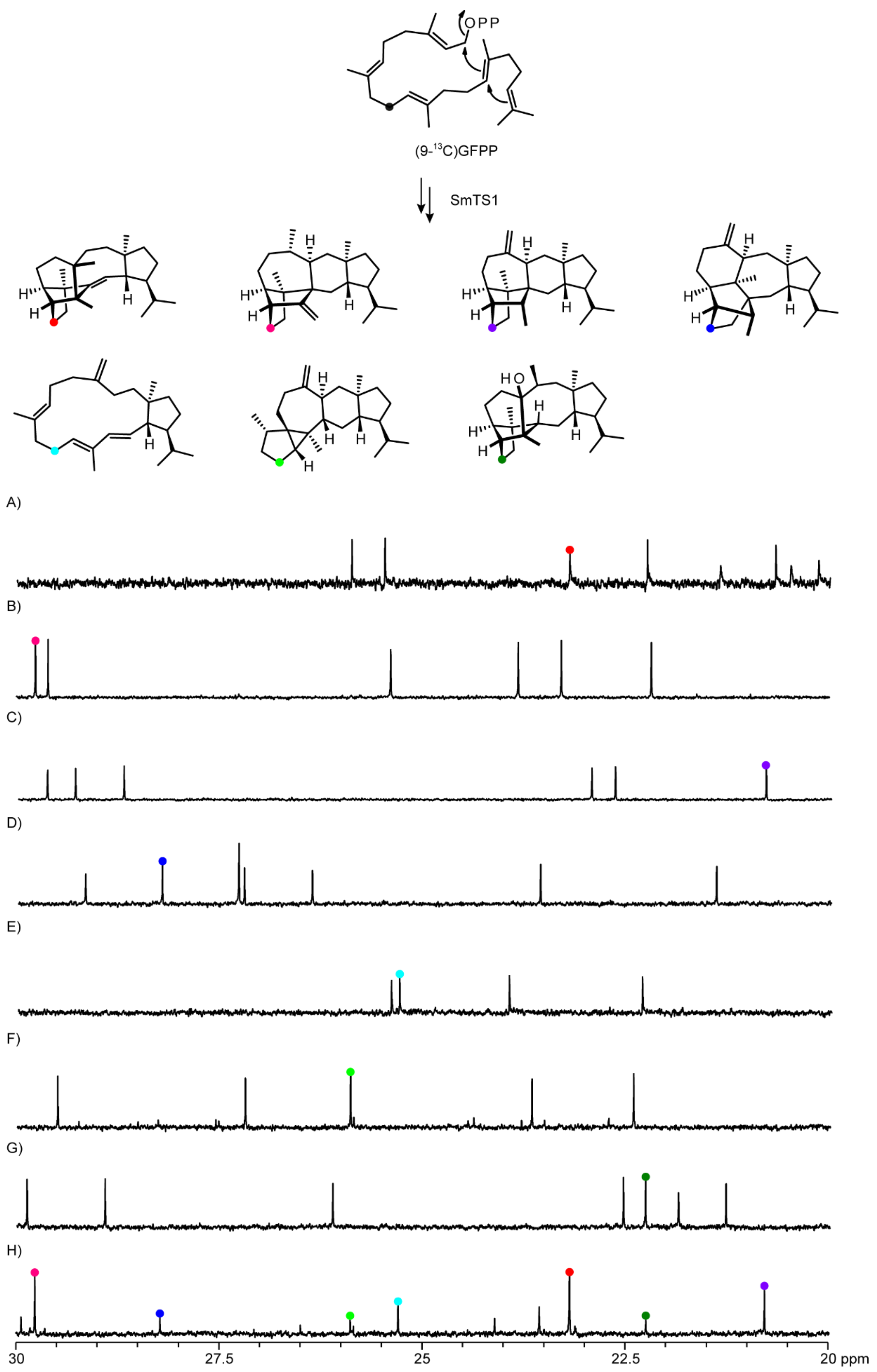

Figure S71. Enzymatic conversion of (9-^{13}C)GFPP with SmTS1. Coloured dots indicate labeled carbons and the corresponding peaks in the ^{13}C-NMR spectra. Figures A) – G) show the ^{13}C-NMR spectra of unlabeled **6** – **12**, H) shows the ^{13}C-NMR spectrum of the enzyme products from (9-^{13}C)GFPP.

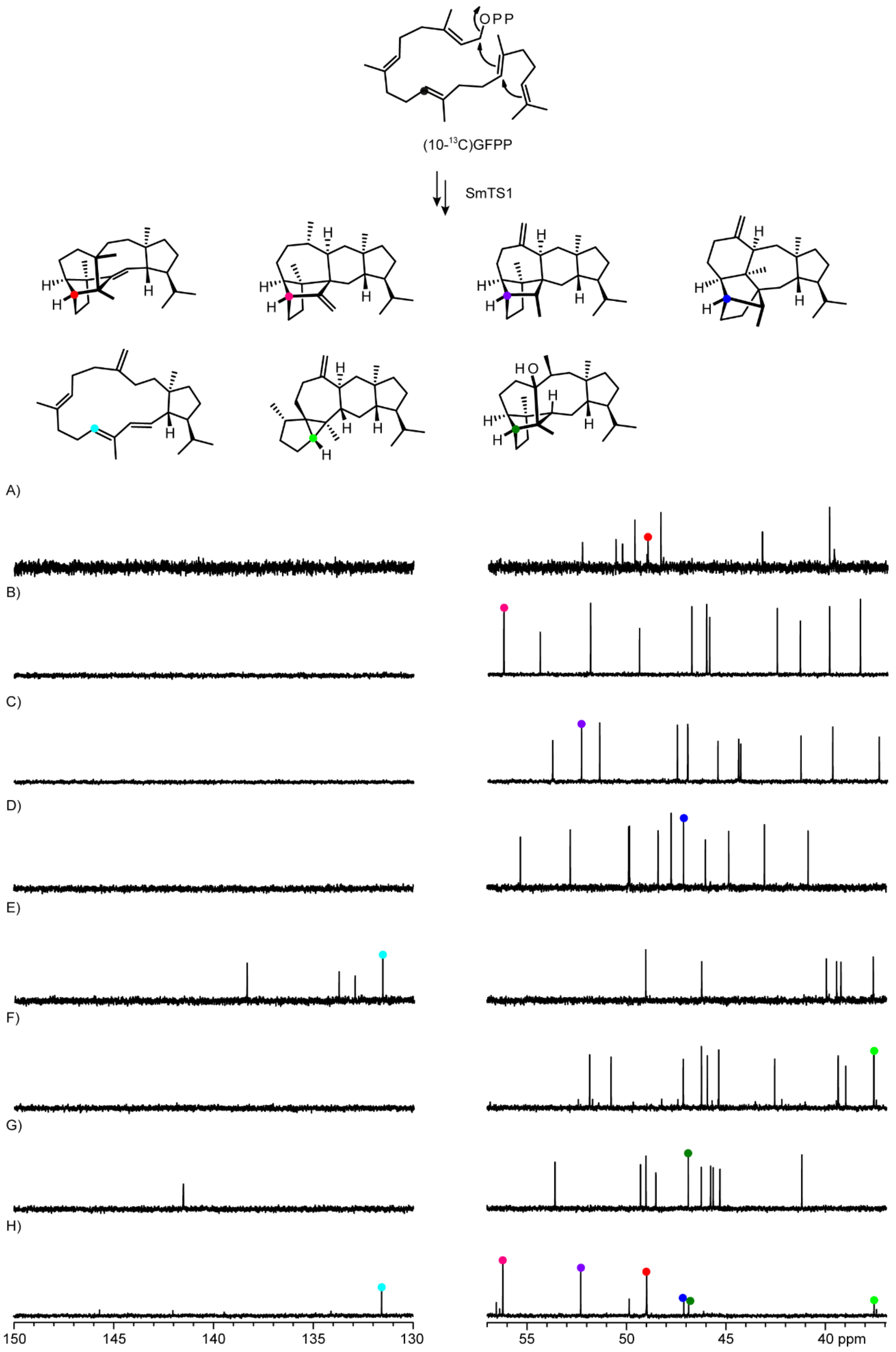

Figure S72. Enzymatic conversion of (10-^{13}C)GFPP with SmTS1. Coloured dots indicate labeled carbons and the corresponding peaks in the ^{13}C-NMR spectra. Figures A) – G) show the ^{13}C-NMR spectra of unlabeled **6** – **12**, H) shows the ^{13}C-NMR spectrum of the enzyme products from (10-^{13}C)GFPP.

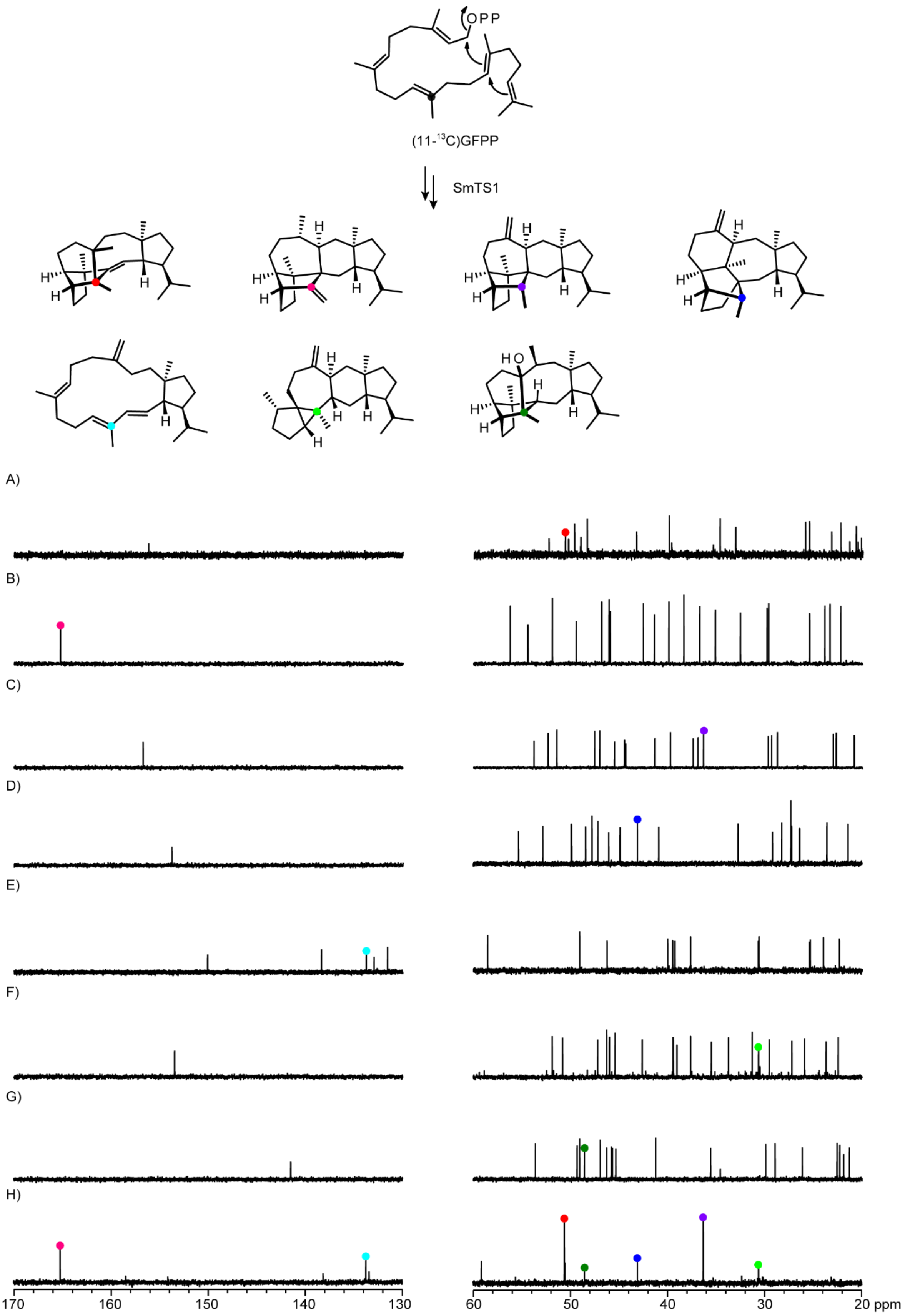

Figure S73. Enzymatic conversion of (11-^{13}C)GFPP with SmTS1. Coloured dots indicate labeled carbons and the corresponding peaks in the ^{13}C-NMR spectra. Figures A) – G) show the ^{13}C-NMR spectra of unlabeled **6** – **12**, H) shows the ^{13}C-NMR spectrum of the enzyme products from (11-^{13}C)GFPP.

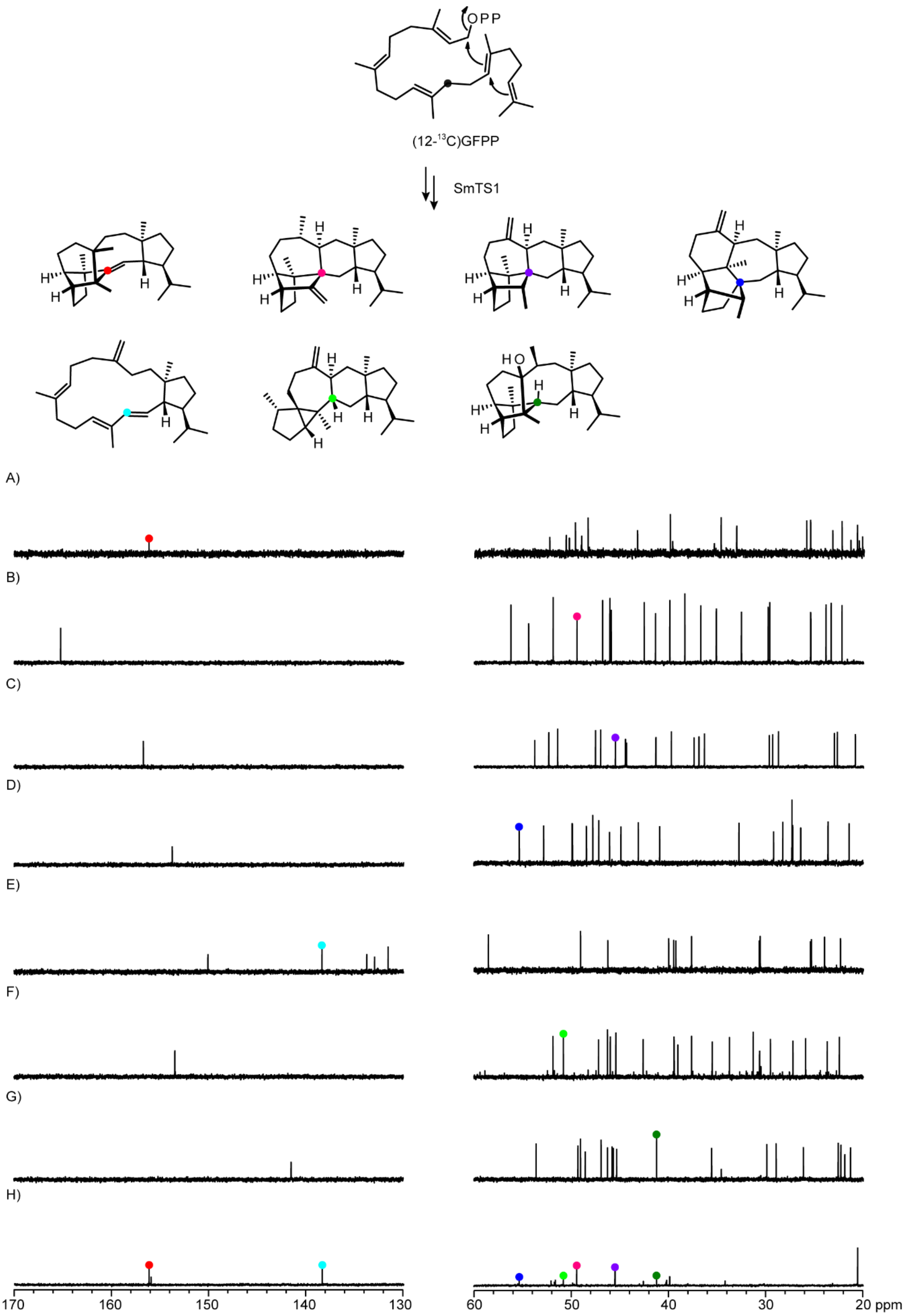

Figure S74. Enzymatic conversion of (12-^{13}C)GFPP with SmTS1. Coloured dots indicate labeled carbons and the corresponding peaks in the ^{13}C-NMR spectra. Figures A) – G) show the ^{13}C-NMR spectra of unlabeled **6** – **12**, H) shows the ^{13}C-NMR spectrum of the enzyme products from (12-^{13}C)GFPP.

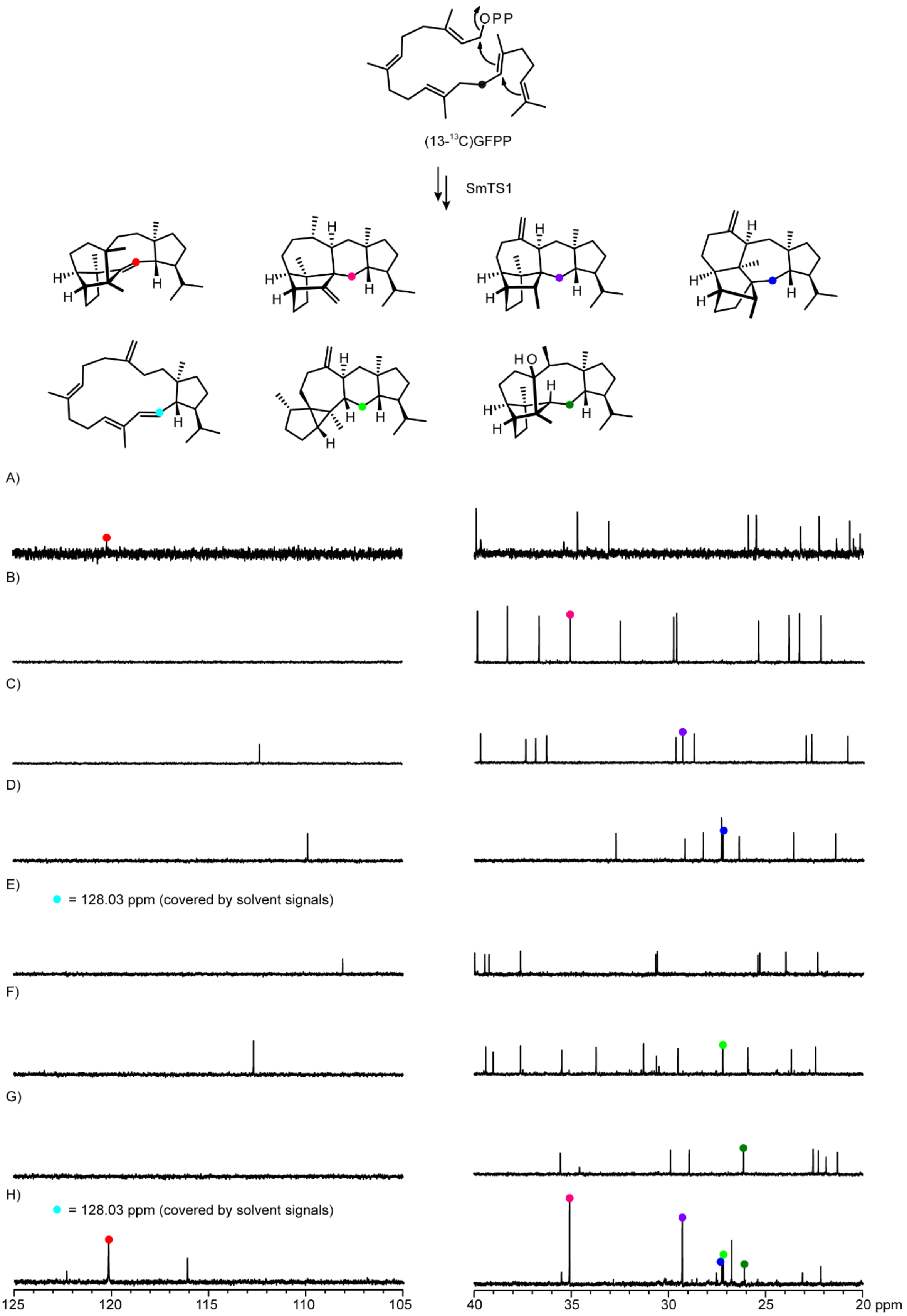

Figure S75. Enzymatic conversion of (13-^{13}C)GFPP with SmTS1. Coloured dots indicate labeled carbons and the corresponding peaks in the ^{13}C-NMR spectra. Figures A) – G) show the ^{13}C-NMR spectra of unlabeled **6** – **12**, H) shows the ^{13}C-NMR spectrum of the enzyme products from (13-^{13}C)GFPP.

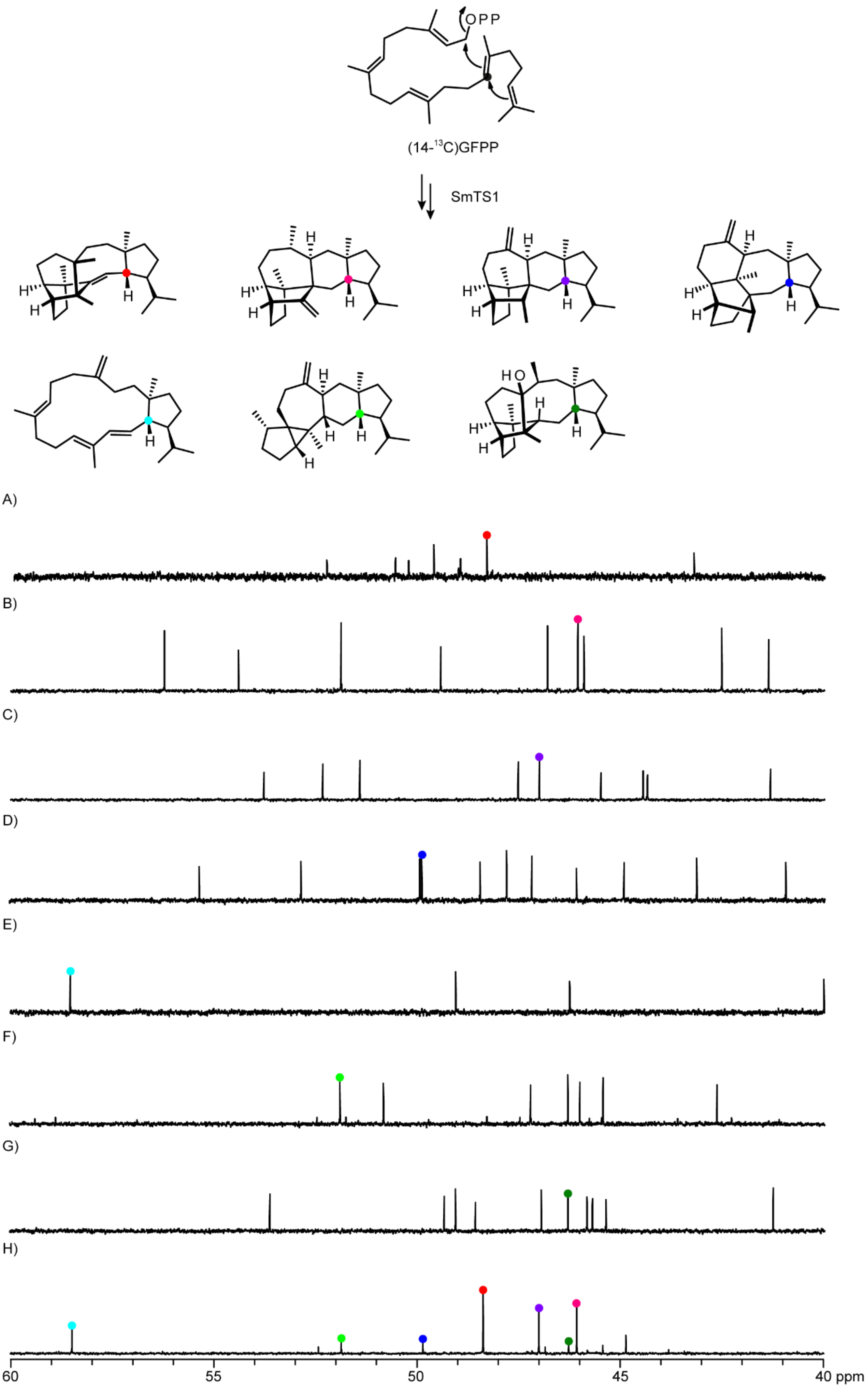

Figure S76. Enzymatic conversion of (14-^{13}C)GFPP with SmTS1. Coloured dots indicate labeled carbons and the corresponding peaks in the ^{13}C-NMR spectra. Figures A) – G) show the ^{13}C-NMR spectra of unlabeled **6** – **12**, H) shows the ^{13}C-NMR spectrum of the enzyme products from (14-^{13}C)GFPP.

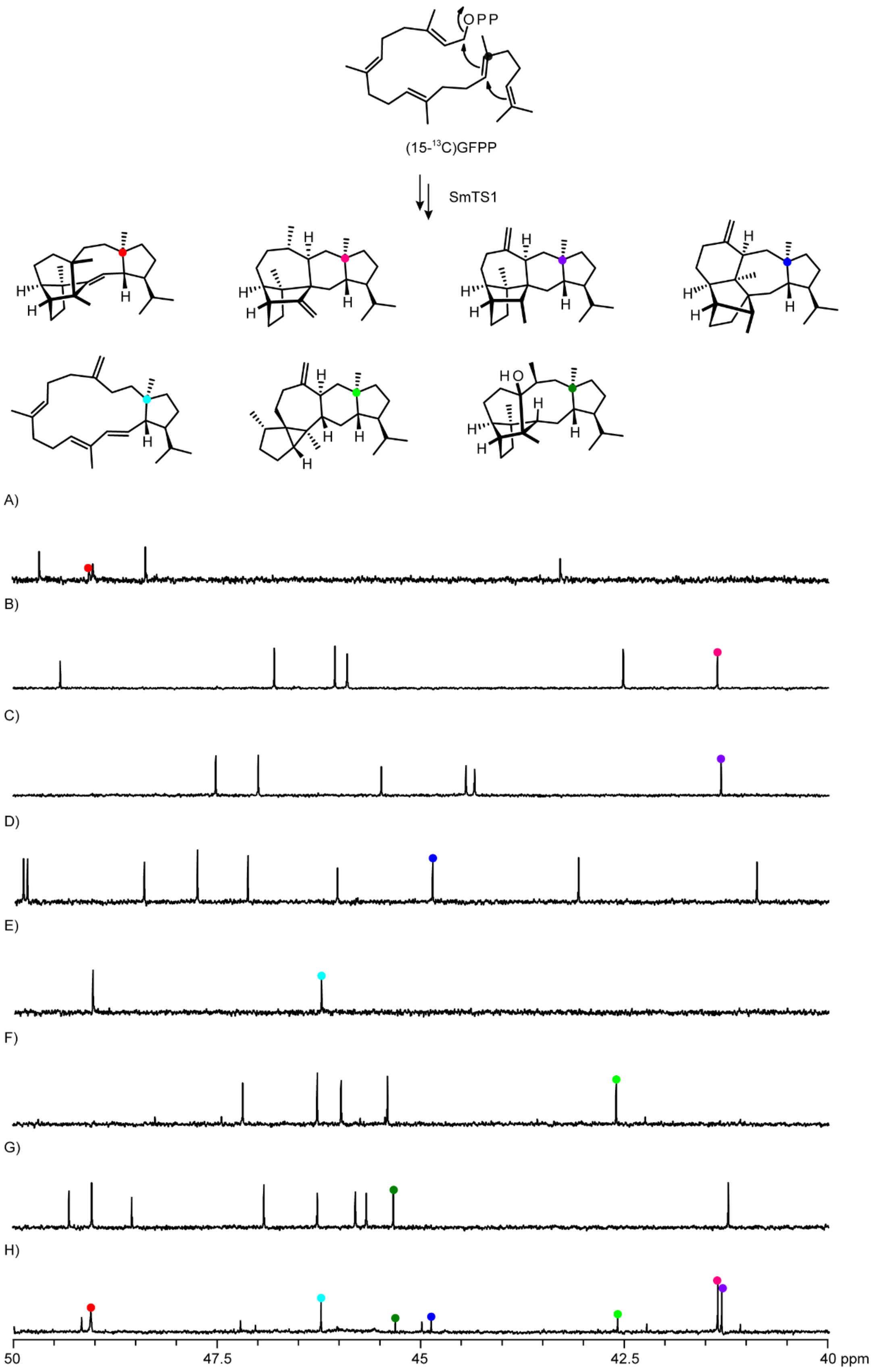

Figure S77. Enzymatic conversion of (15-^{13}C)GFPP with SmTS1. Coloured dots indicate labeled carbons and the corresponding peaks in the ^{13}C-NMR spectra. Figures A) – G) show the ^{13}C-NMR spectra of unlabeled **6** – **12**, H) shows the ^{13}C-NMR spectrum of the enzyme products from (15-^{13}C)GFPP.

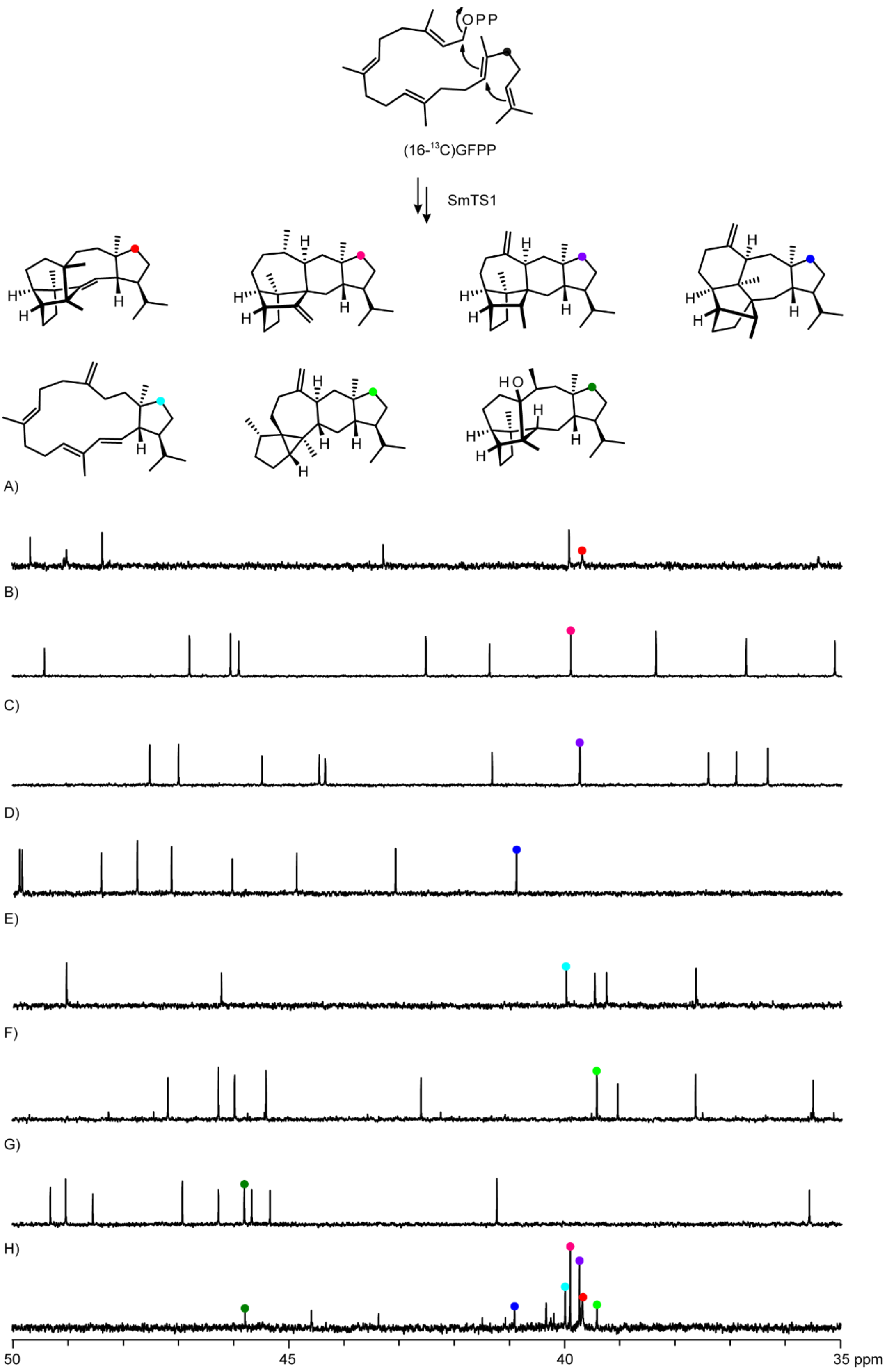

Figure S78. Enzymatic conversion of (16-^{13}C)GFPP with SmTS1. Coloured dots indicate labeled carbons and the corresponding peaks in the ^{13}C-NMR spectra. Figures A) – G) show the ^{13}C-NMR spectra of unlabeled **6** – **12**, H) shows the ^{13}C-NMR spectrum of the enzyme products from (16-^{13}C)GFPP.

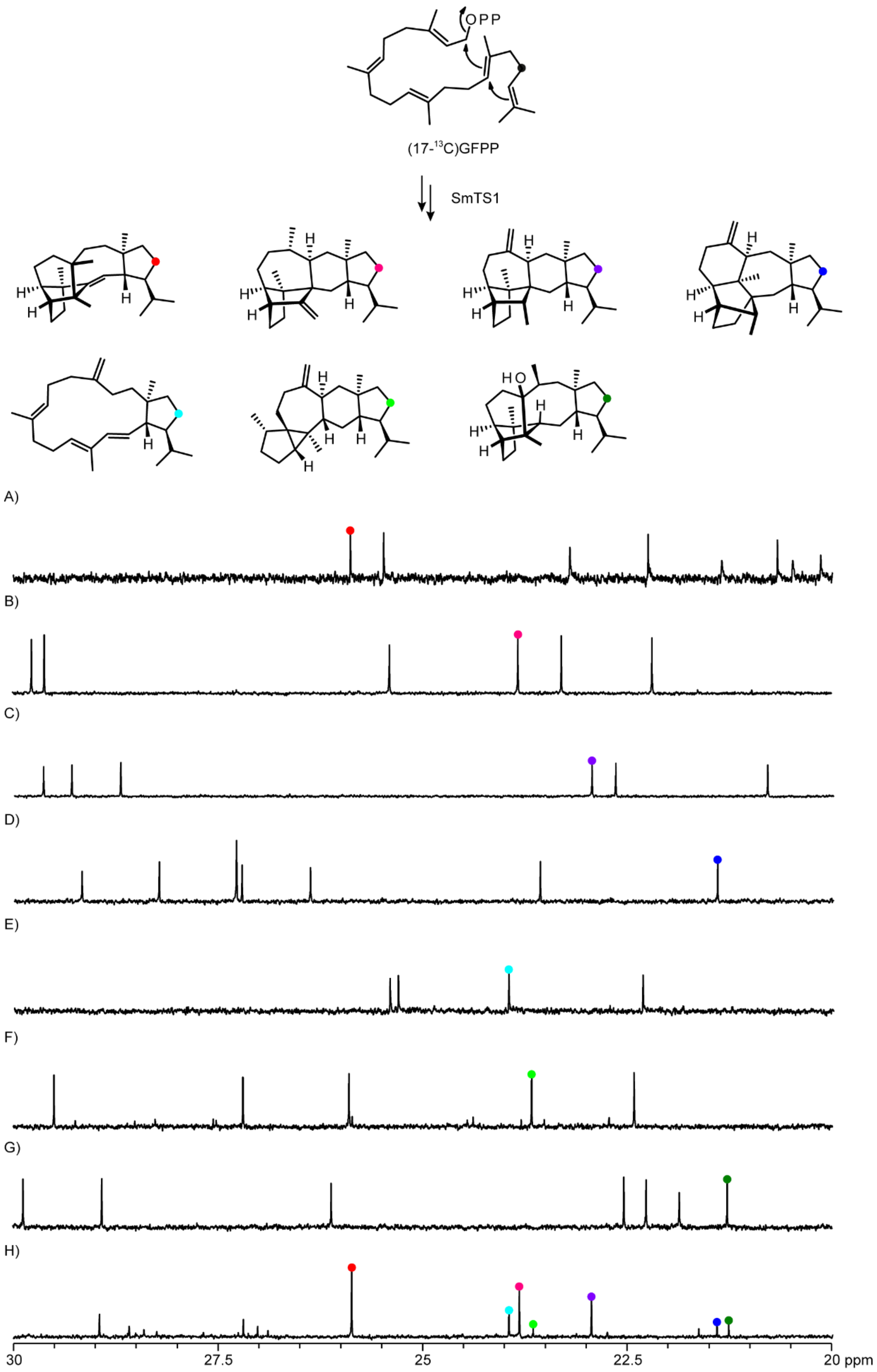

Figure S79. Enzymatic conversion of (17-^{13}C)GFPP with SmTS1. Coloured dots indicate labeled carbons and the corresponding peaks in the ^{13}C-NMR spectra. Figures A) – G) show the ^{13}C-NMR spectra of unlabeled **6** – **12**, H) shows the ^{13}C-NMR spectrum of the enzyme products from (17-^{13}C)GFPP.

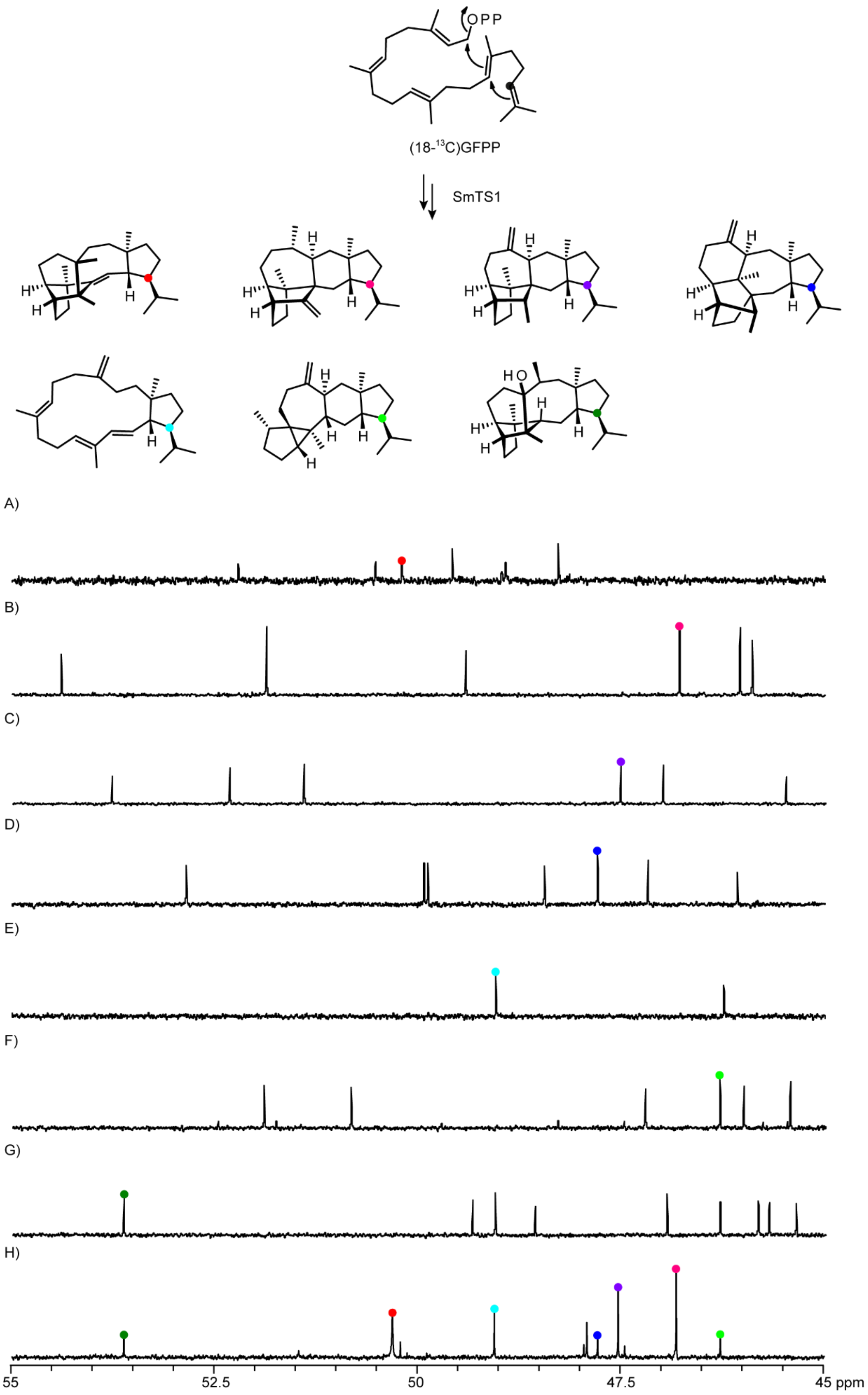

Figure S80. Enzymatic conversion of (18-^{13}C)GFPP with SmTS1. Coloured dots indicate labeled carbons and the corresponding peaks in the ^{13}C-NMR spectra. Figures A) – G) show the ^{13}C-NMR spectra of unlabeled **6** – **12**, H) shows the ^{13}C-NMR spectrum of the enzyme products from (18-^{13}C)GFPP.

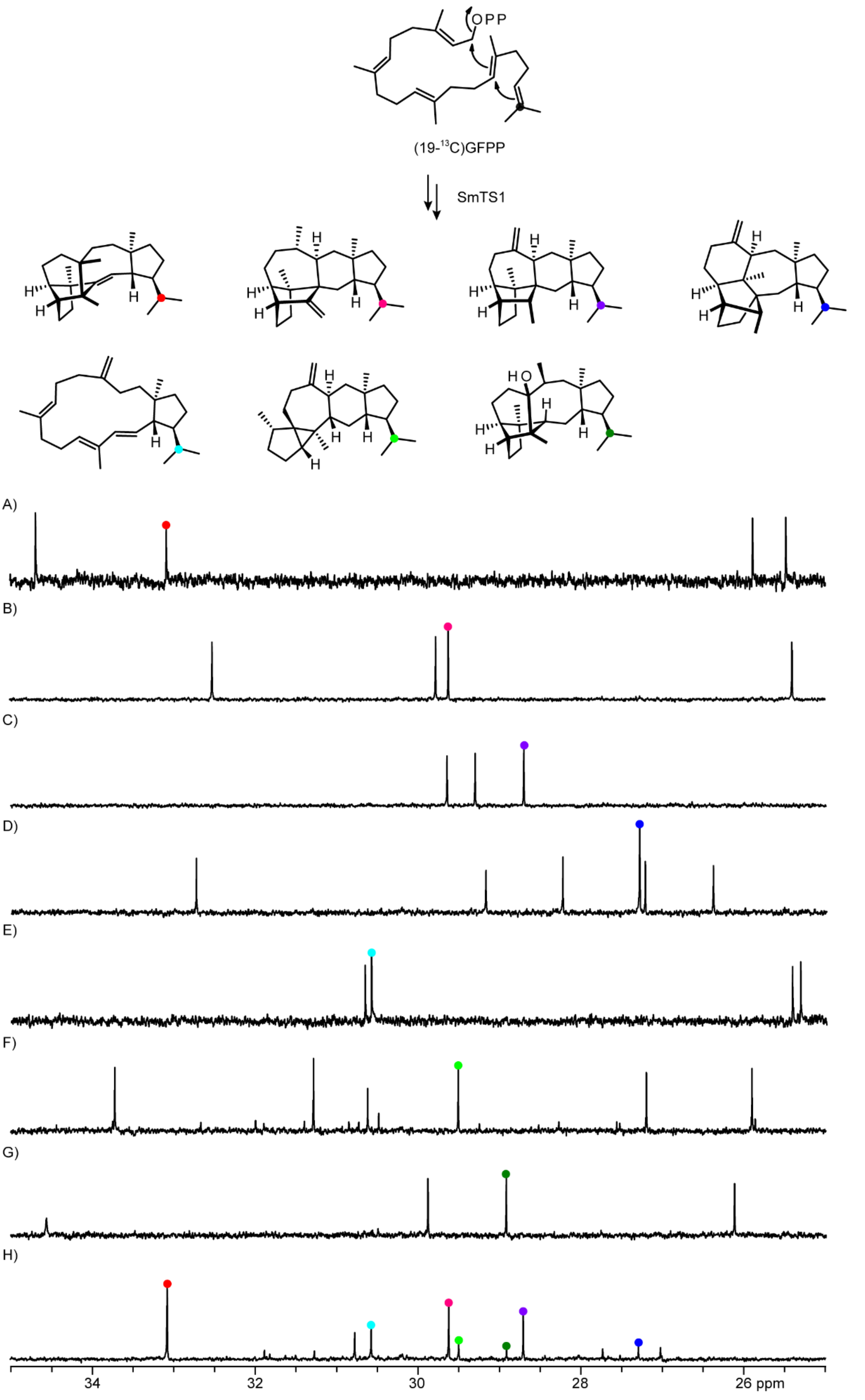

Figure S81. Enzymatic conversion of (19-^{13}C)GFPP with SmTS1. Coloured dots indicate labeled carbons and the corresponding peaks in the ^{13}C-NMR spectra. Figures A) – G) show the ^{13}C-NMR spectra of unlabeled **6** – **12**, H) shows the ^{13}C-NMR spectrum of the enzyme products from (19-^{13}C)GFPP.

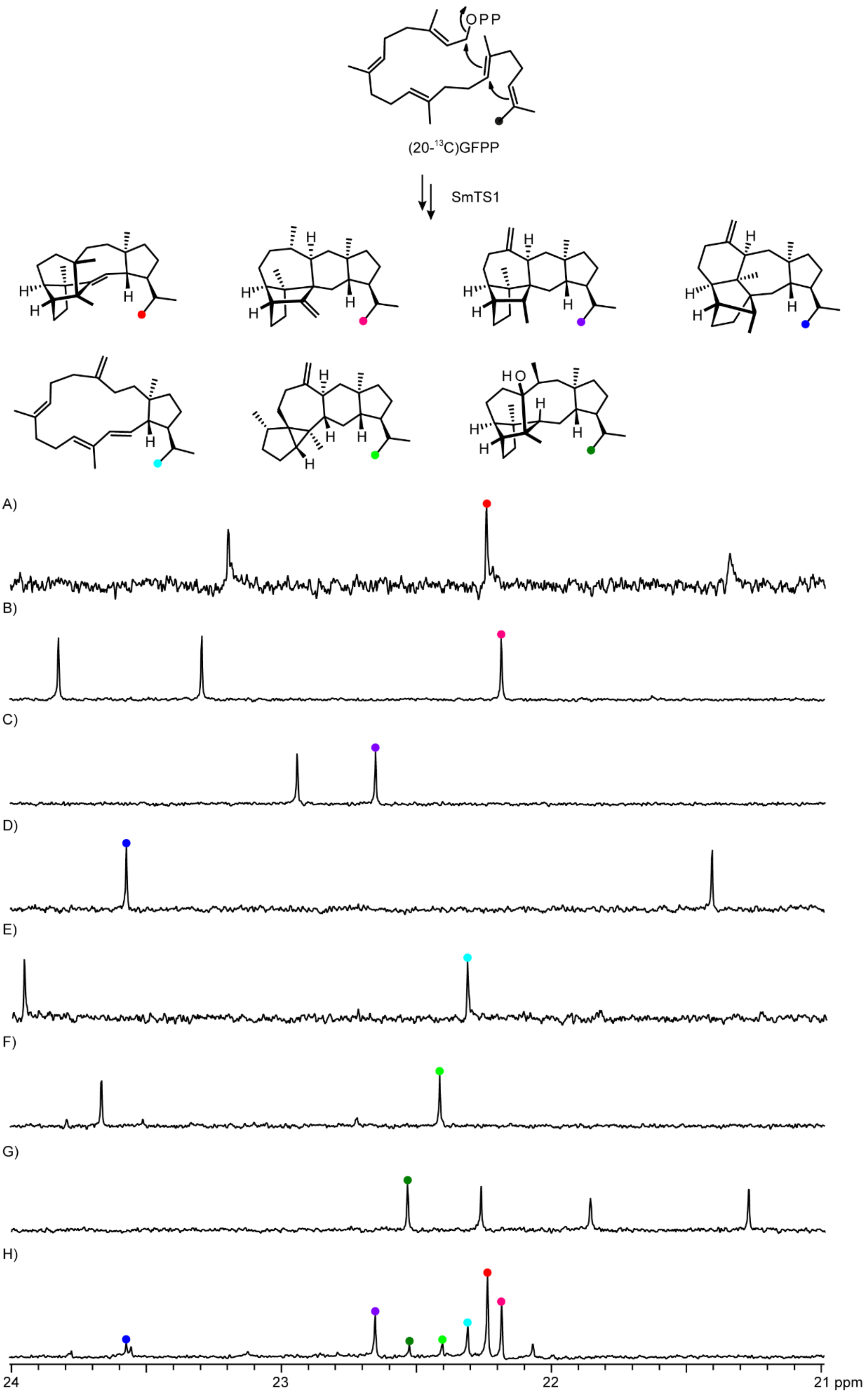

Figure S82. Enzymatic conversion of (20-^{13}C)GFPP with SmTS1. Coloured dots indicate labeled carbons and the corresponding peaks in the ^{13}C-NMR spectra. Figures A) – G) show the ^{13}C-NMR spectra of unlabeled **6** – **12**, H) shows the ^{13}C-NMR spectrum of the enzyme products from (20-^{13}C)GFPP.

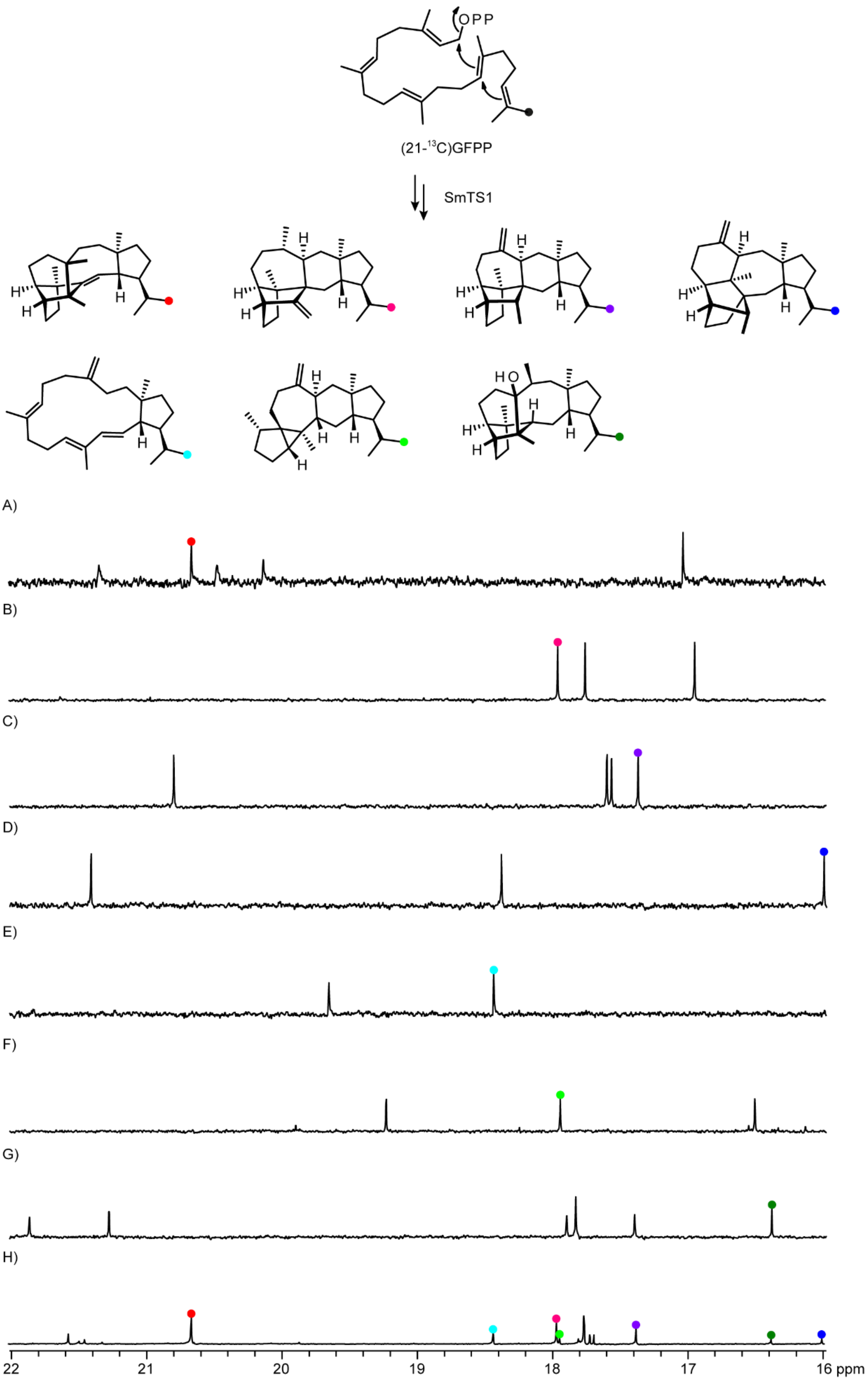

Figure S83. Enzymatic conversion of (21-^{13}C)GFPP with SmTS1. Coloured dots indicate labeled carbons and the corresponding peaks in the ^{13}C-NMR spectra. Figures A) – G) show the ^{13}C-NMR spectra of unlabeled **6** – **12**, H) shows the ^{13}C-NMR spectrum of the enzyme products from (21-^{13}C)GFPP.

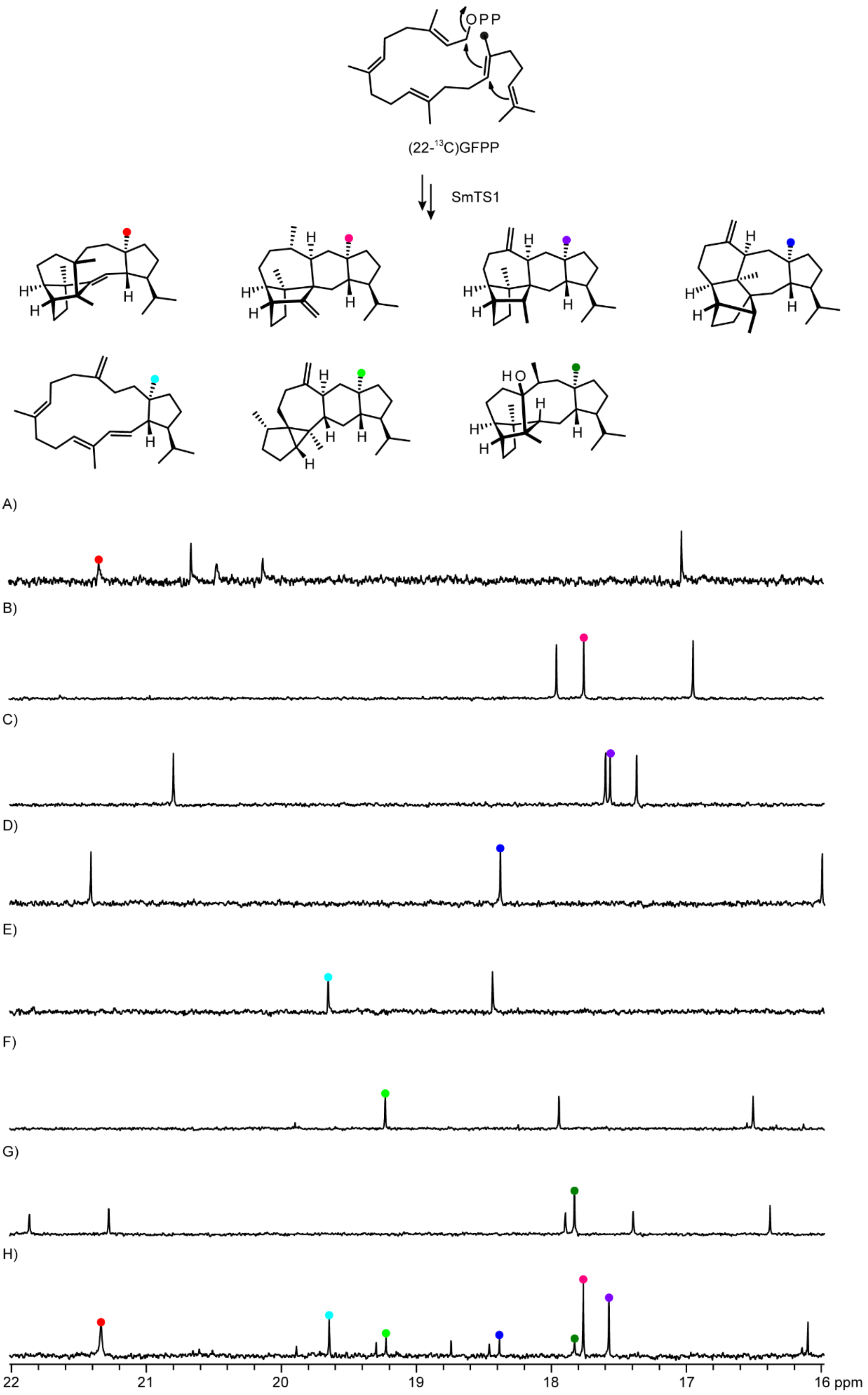

Figure S84. Enzymatic conversion of (22-^{13}C)GFPP with SmTS1. Coloured dots indicate labeled carbons and the corresponding peaks in the ^{13}C-NMR spectra. Figures A) – G) show the ^{13}C-NMR spectra of unlabeled **6** – **12**, H) shows the ^{13}C-NMR spectrum of the enzyme products from (22-^{13}C)GFPP.

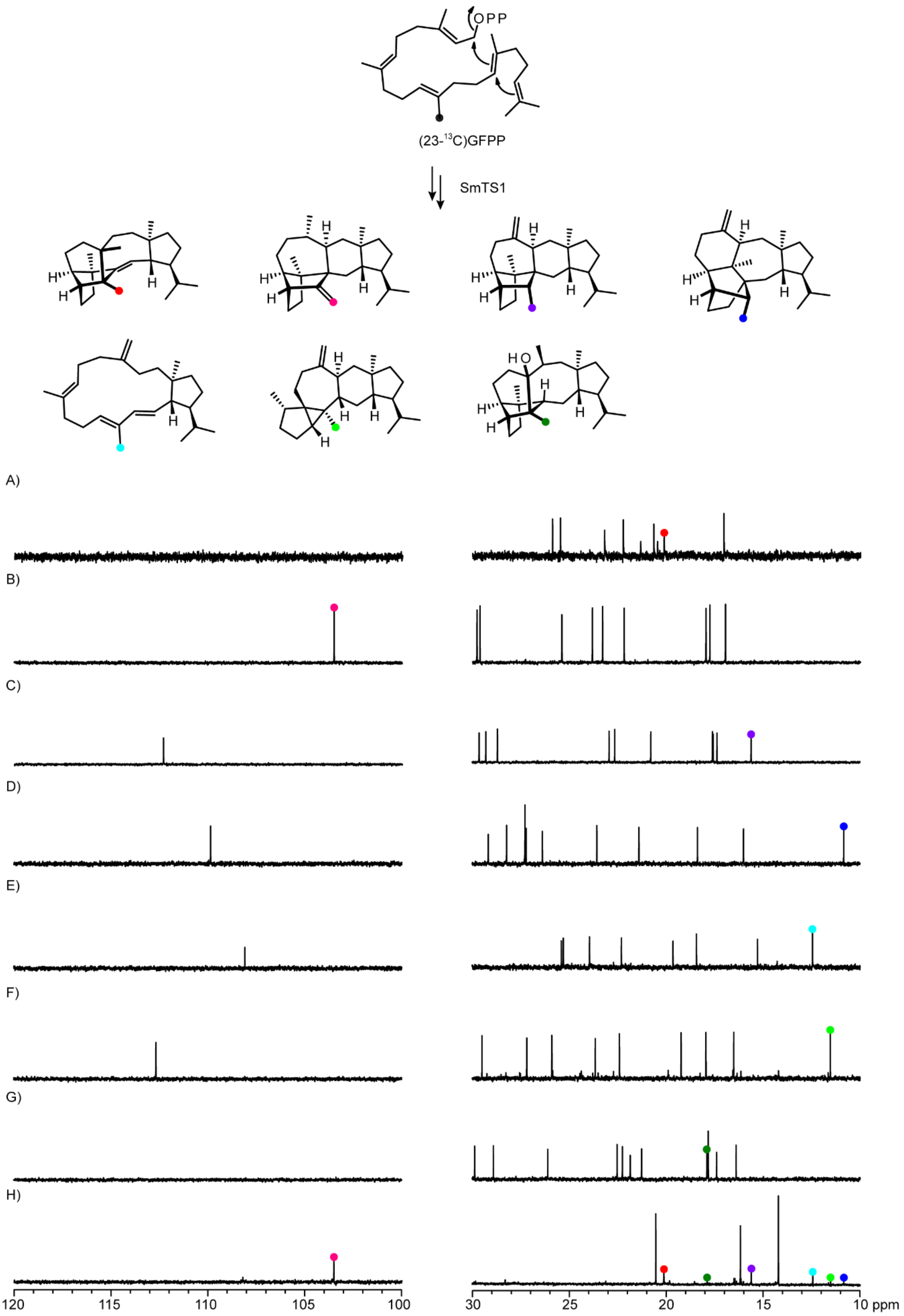

Figure S85. Enzymatic conversion of (23-^{13}C)GFPP with SmTS1. Coloured dots indicate labeled carbons and the corresponding peaks in the ^{13}C-NMR spectra. Figures A) – G) show the ^{13}C-NMR spectra of unlabeled **6** – **12**, H) shows the ^{13}C-NMR spectrum of the enzyme products from (23-^{13}C)GFPP.

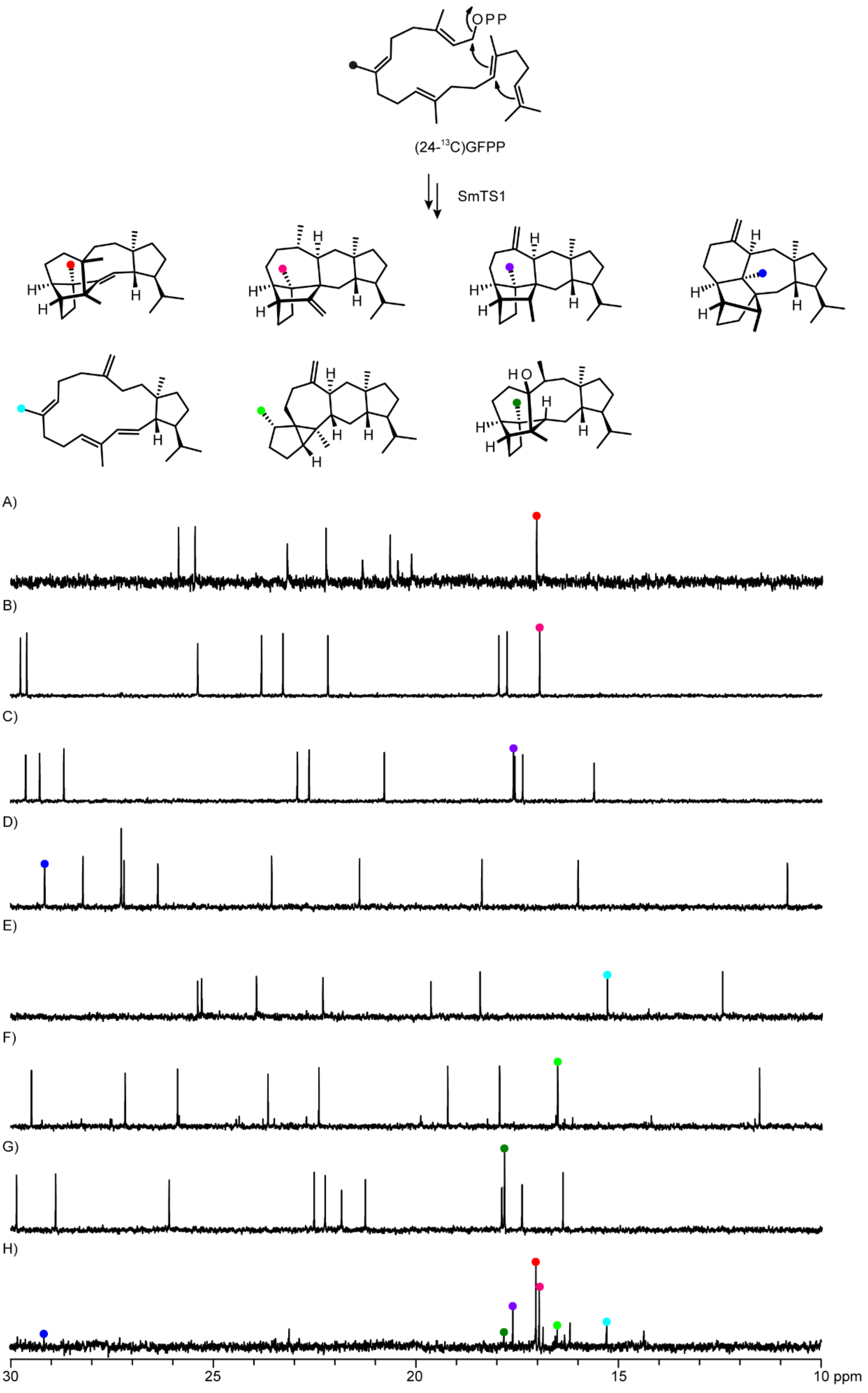

Figure S86. Enzymatic conversion of (24-^{13}C)GFPP with SmTS1. Coloured dots indicate labeled carbons and the corresponding peaks in the ^{13}C-NMR spectra. Figures A) – G) show the ^{13}C-NMR spectra of unlabeled **6** – **12**, H) shows the ^{13}C-NMR spectrum of the enzyme products from (24-^{13}C)GFPP.

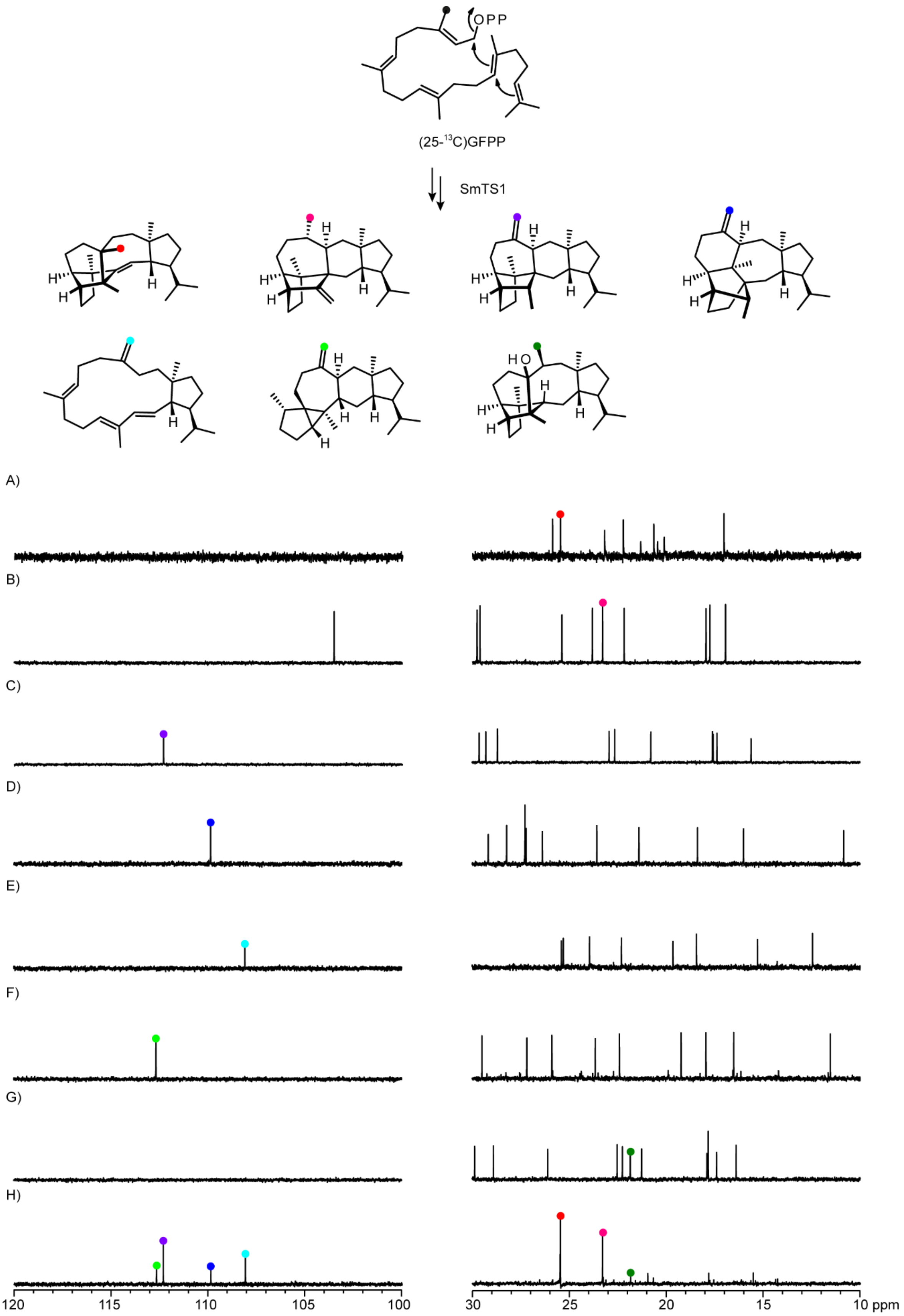

Figure S87. Enzymatic conversion of (25-^{13}C)GFPP with SmTS1. Coloured dots indicate labeled carbons and the corresponding peaks in the ^{13}C-NMR spectra. Figures A) – G) show the ^{13}C-NMR spectra of unlabeled **6** – **12**, H) shows the ^{13}C-NMR spectrum of the enzyme products from (25-^{13}C)GFPP.

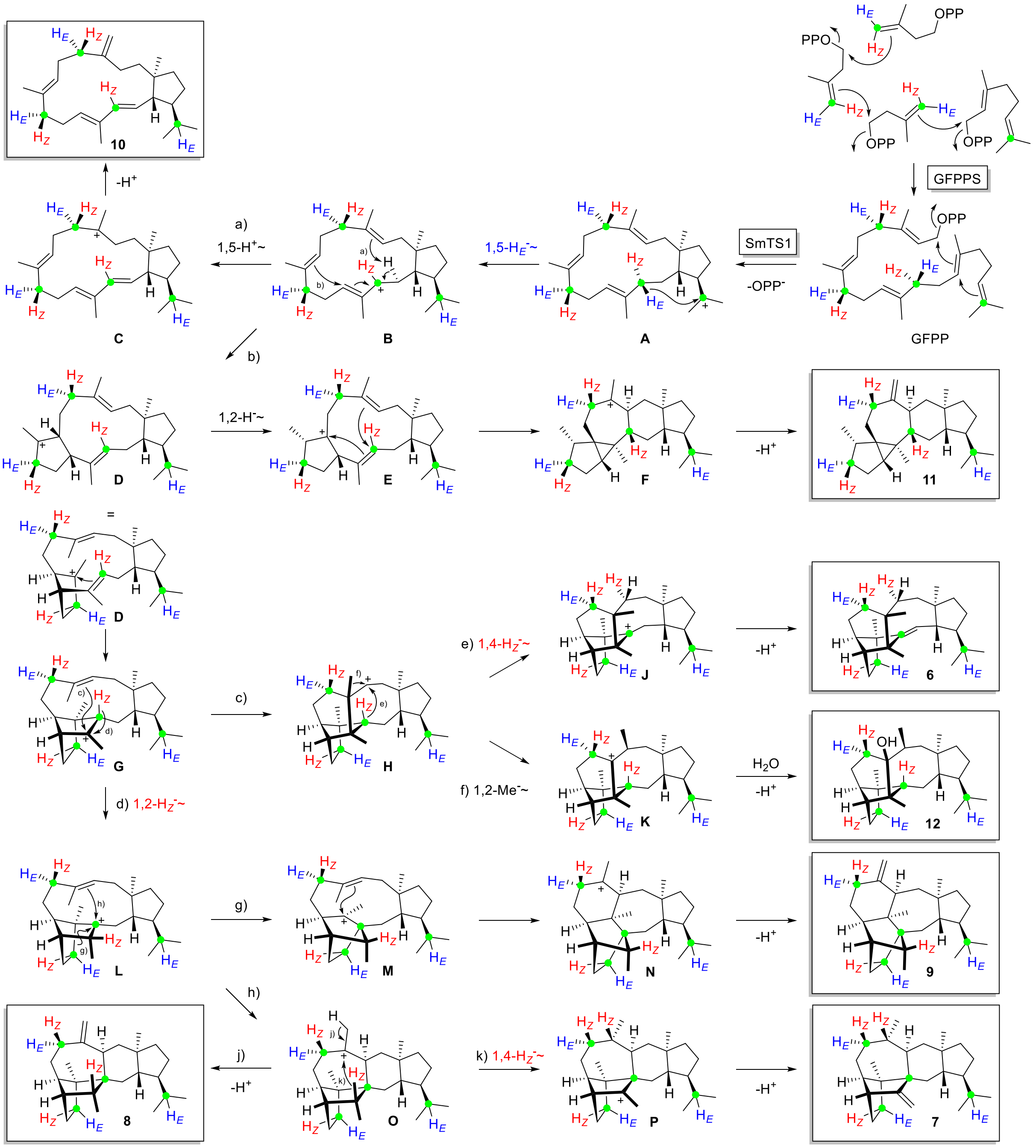

Scheme S1. Biosynthesis of labeled **6 – 12** from (7-^{13}C)GPP and (*E*)- or (*Z*)-(4-^{13}C,4-^{2}H)IPP with GFPPS and SmTS1.

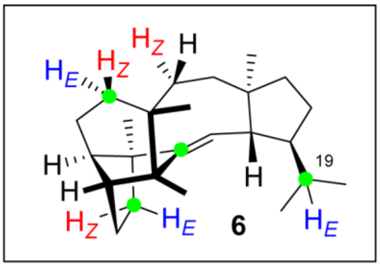

← Scheme S1

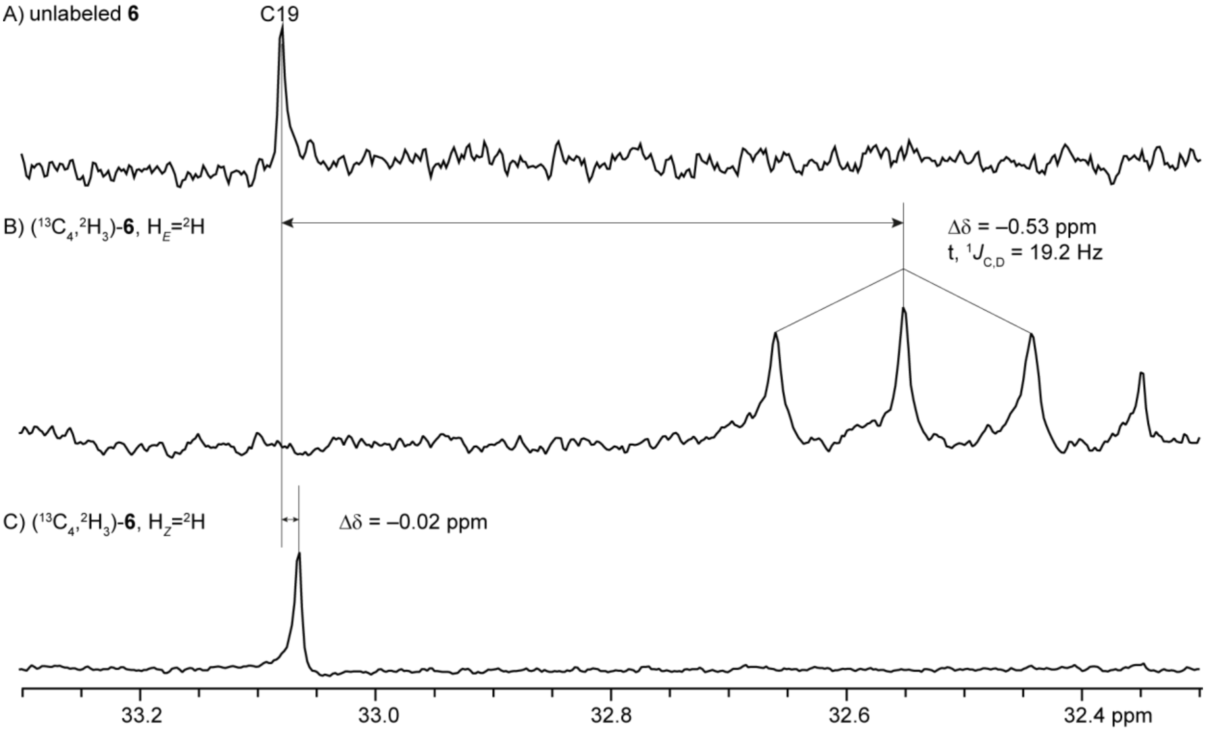

Figure S88. 1,5-Hydride shift from **A** to **B** in the formation of compound **6**. A) ^{13}C-NMR signal for C19 of unlabeled **6**, B) ^{13}C-NMR signal for deuterated C19 of labeled **6** obtained from (7-^{13}C)GPP with (*E*)-(4-^{13}C,4-^{2}H)IPP, C) ^{13}C-NMR signal for non-deuterated C19 of labeled **6** obtained from (7-^{13}C)GPP with (*Z*)-(4-^{13}C,4-^{2}H)IPP. The slightly upfield shifted triplet in B) is indicative for a direct ^{13}C-^{2}H bond and supports the proposed 1,5-hydride shift.

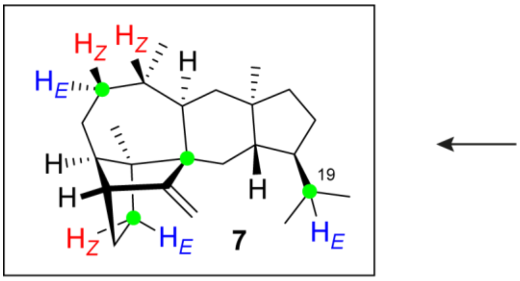

Scheme S1

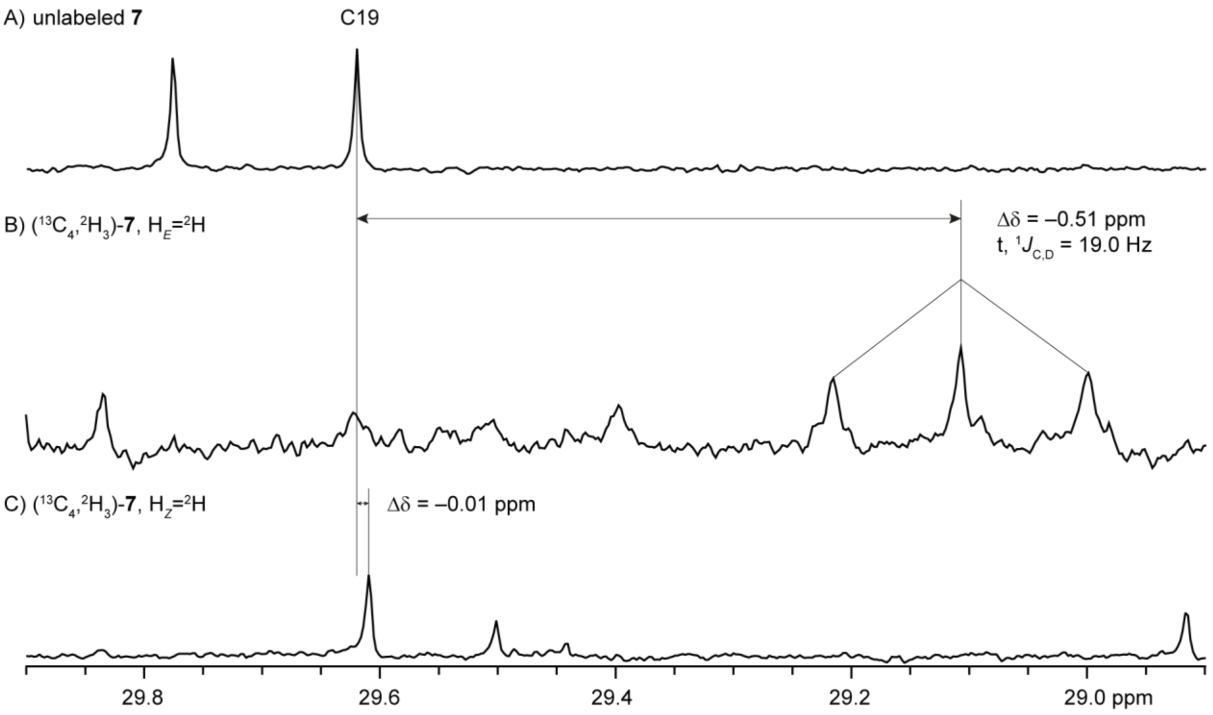

Figure S89. 1,5-Hydride shift from **A** to **B** in the formation of compound **7**. A) ^{13}C-NMR signal for C19 of unlabeled **7**, B) ^{13}C-NMR signal for deuterated C19 of labeled **7** obtained from (7-^{13}C)GPP with (*E*)-(4-^{13}C,4-^{2}H)IPP, C) ^{13}C-NMR signal for non-deuterated C19 of labeled **7** obtained from (7-^{13}C)GPP with (*Z*)-(4-^{13}C,4-^{2}H)IPP. The slightly upfield shifted triplet in B) is indicative for a direct ^{13}C-^{2}H bond and supports the proposed 1,5-hydride shift.

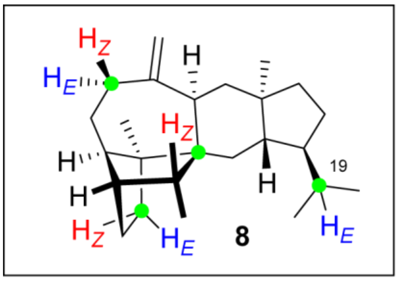

← Scheme S1

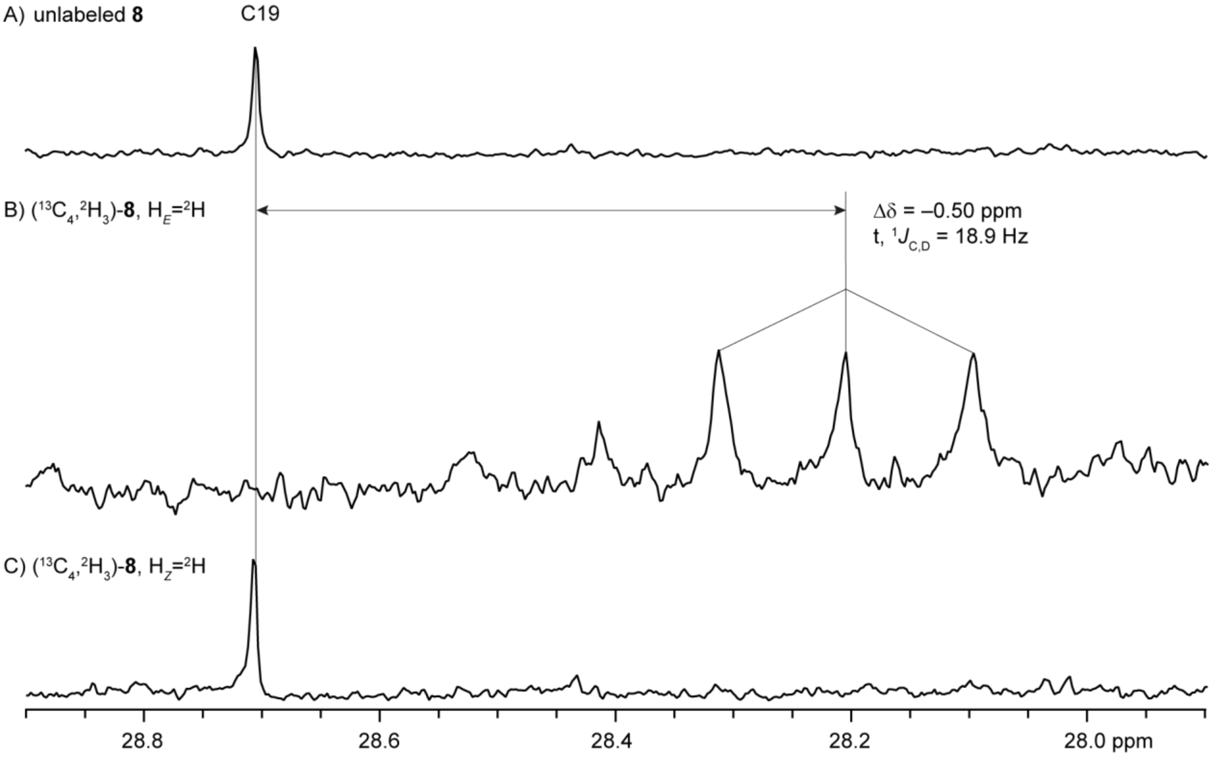

Figure S90. 1,5-Hydride shifts from **A** to **B** in the formation of compound **8**, A) ^{13}C-NMR signal for C19 of unlabeled **8**, B) ^{13}C-NMR signal for deuterated C19 of labeled **8** obtained from (7-^{13}C)GPP with (*E*)-(4-^{13}C,4-^{2}H)IPP, C) ^{13}C-NMR signal for non-deuterated C19 of labeled **8** obtained from (7-^{13}C)GPP with (*Z*)-(4-^{13}C,4-^{2}H)IPP. The slightly upfield shifted triplet in B) is indicative for a direct ^{13}C-^{2}H bond and supports the proposed 1,5-hydride shift.

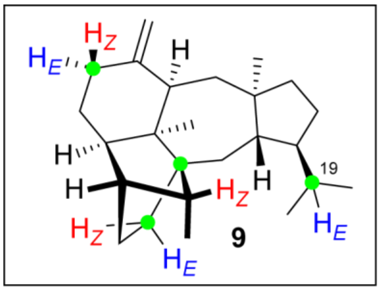

← Scheme S1

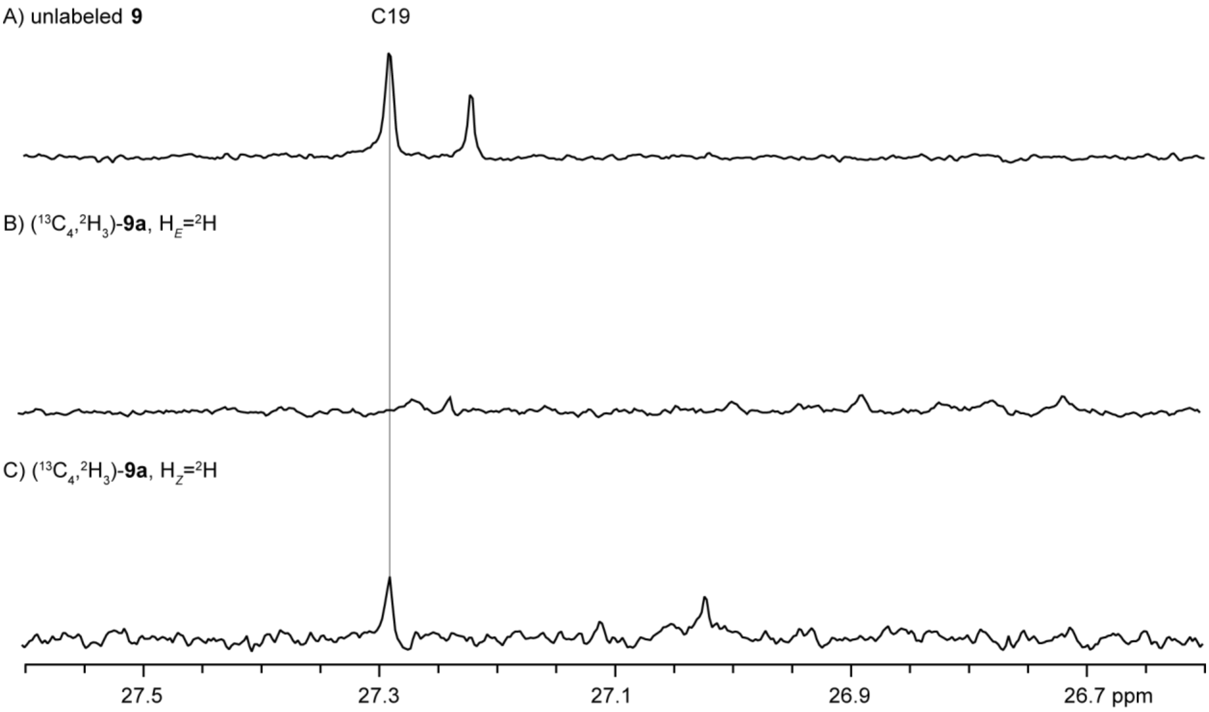

Figure S91. 1,5-Hydride shift from **A** to **B** in the formation of compound **9**, A) ^{13}C-NMR signal for C19 of unlabeled **9**, B) ^{13}C-NMR spectrum for deuterated C19 of labeled **9** obtained from (7-^{13}C)GPP with (*E*)-(4-^{13}C,4-^{2}H)IPP, C) ^{13}C-NMR signal for non-deuterated C19 of labeled **9** obtained from (7-^{13}C)GPP with (*Z*)-(4-^{13}C,4-^{2}H)IPP. As compound **9** was a minor product, the expected triplet signal in B) could not be observed. However, the presence of a signal for C19 in C) together with the absence in B) indicates deuteration of C19 for H_E = ^{2}H and thus supports the 1,5-hydride shift.

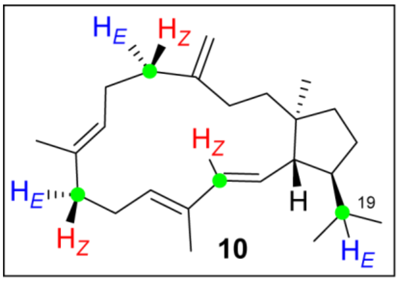

Scheme S1

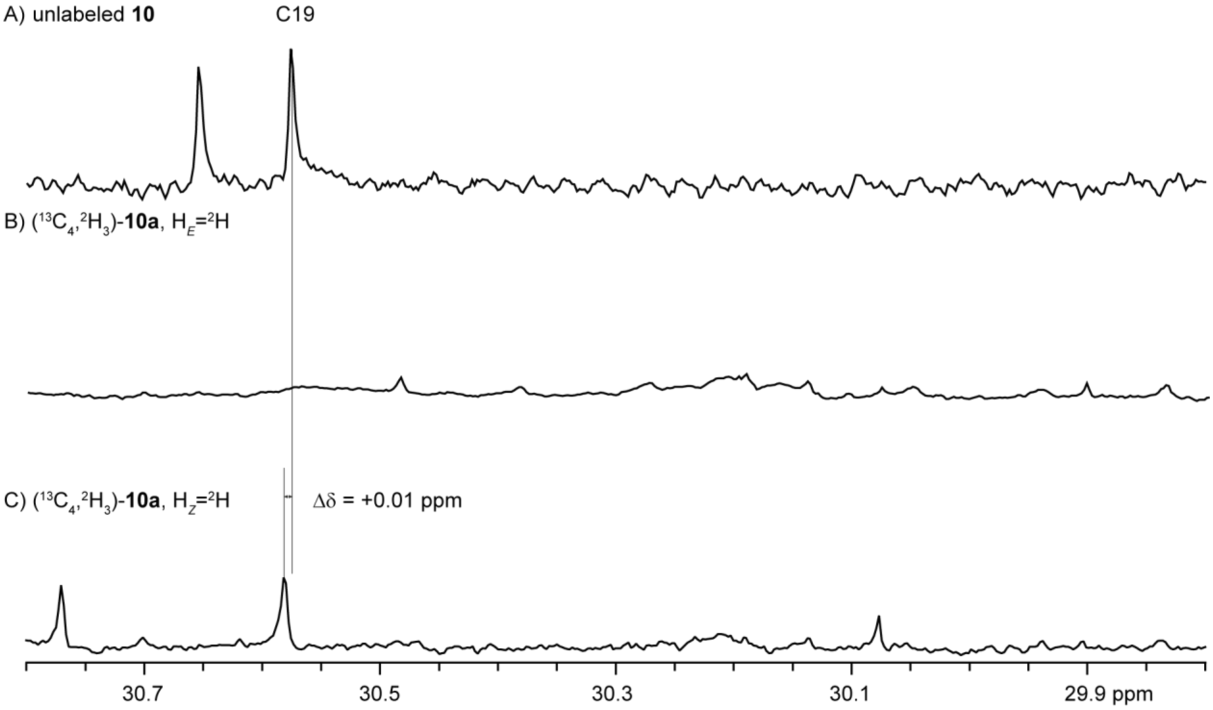

Figure S92. 1,5-Hydride shifts from **A** to **B** in the formation of compound **10**, A) ^{13}C-NMR signal for C19 of unlabeled **10**, B) ^{13}C-NMR spectrum for deuterated C19 of labeled **10** obtained from (7-^{13}C)GPP with (*E*)-(4-^{13}C,4-^{2}H)IPP, C) ^{13}C-NMR signal for non-deuterated C19 of labeled **10** obtained from (7-^{13}C)GPP with (*Z*)-(4-^{13}C,4-^{2}H)IPP. As compound **10** was a minor product, the expected triplet signal in B) could not be observed. However, the presence of a signal for C19 in C) together with the absence in B) indicates deuteration of C19 for H_E = ^{2}H and thus supports the 1,5-hydride shift.

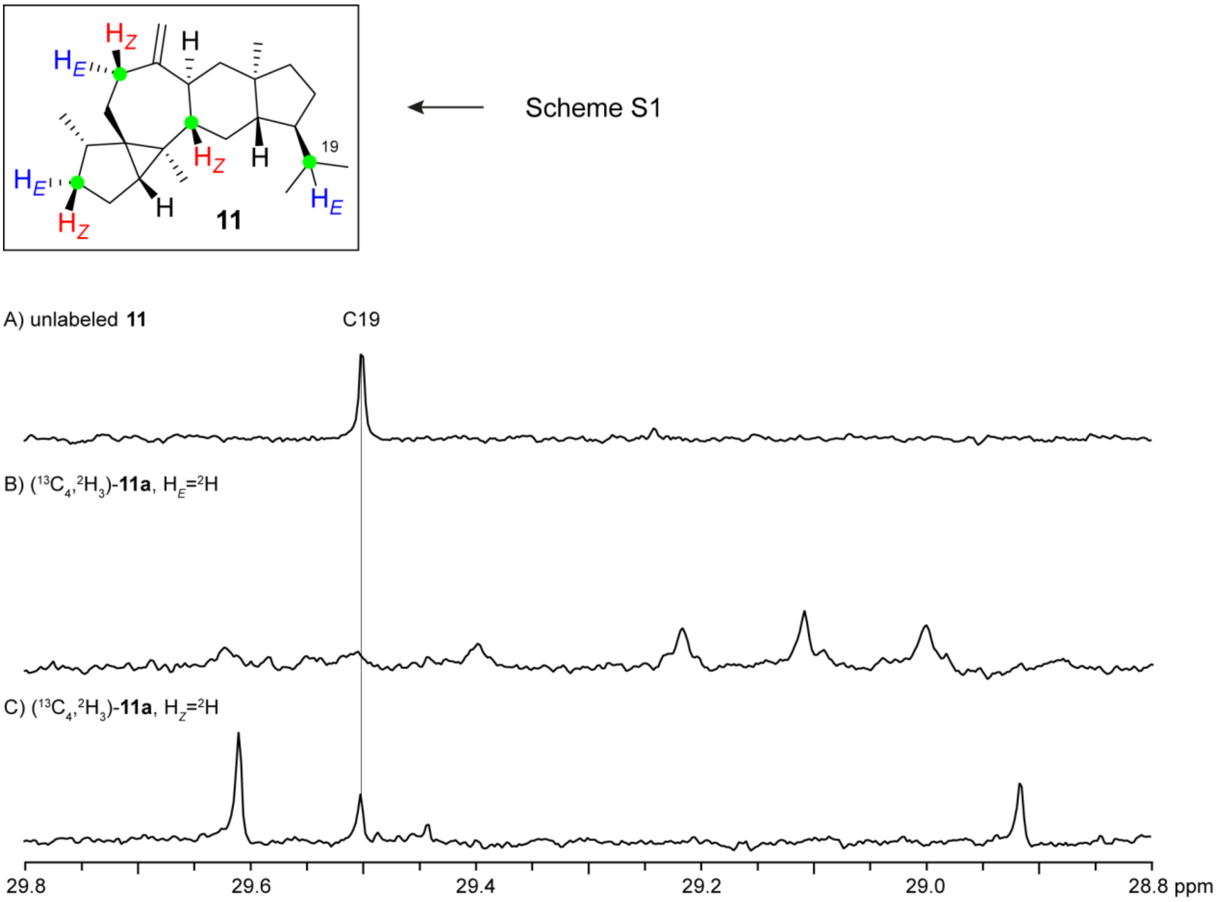

Figure S93. 1,5-Hydride shift from **A** to **B** in the formation of compound **11**, A) ^{13}C-NMR signal for C19 of unlabeled **11**, B) ^{13}C-NMR spectrum for deuterated C19 of labeled **11** obtained from (7-^{13}C)GPP with (*E*)-(4-^{13}C,4-^{2}H)IPP, C) ^{13}C-NMR signal for non-deuterated C19 of labeled **11** obtained from (7-^{13}C)GPP with (*Z*)-(4-^{13}C,4-^{2}H)IPP. As compound **11** was a minor product, the expected triplet signal in B) could not be observed (the triplet that is visible at 29.1 ppm has a smaler $\Delta\delta$ = –0.40 than expected (ca. $\Delta\delta$ = –0.5) and originates from another compound in the sample). However, the presence of a signal for C19 in C) together with the absence in B) indicates deuteration of C19 for H_E = ^{2}H and thus supports the 1,5-hydride shift.

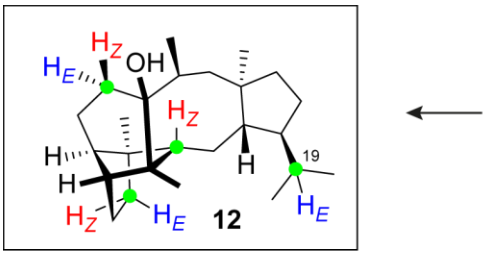

Scheme S1

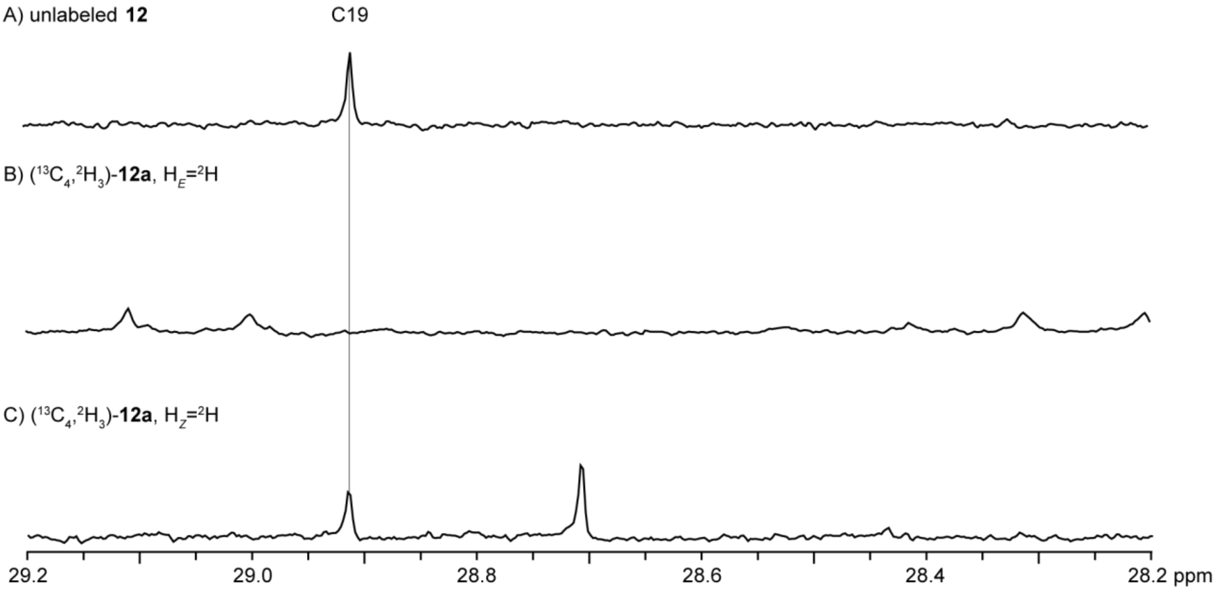

Figure S94. 1,5-Hydride shift from **A** to **B** in the formation of compound **12**, A) ^{13}C-NMR signal for C19 of unlabeled **12**, B) ^{13}C-NMR spectrum for deuterated C19 of labeled **12** obtained from (7-^{13}C)GPP with (*E*)-(4-^{13}C,4-^{2}H)IPP, C) ^{13}C-NMR signal for non-deuterated C19 of labeled **12** obtained from (7-^{13}C)GPP with (*Z*)-(4-^{13}C,4-^{2}H)IPP. As compound **12** was a minor product, the expected triplet signal in B) could not be observed. However, the presence of a signal for C19 in C) together with the absence in B) indicates deuteration of C19 for H_E = ^{2}H and thus supports the 1,5-hydride shift.

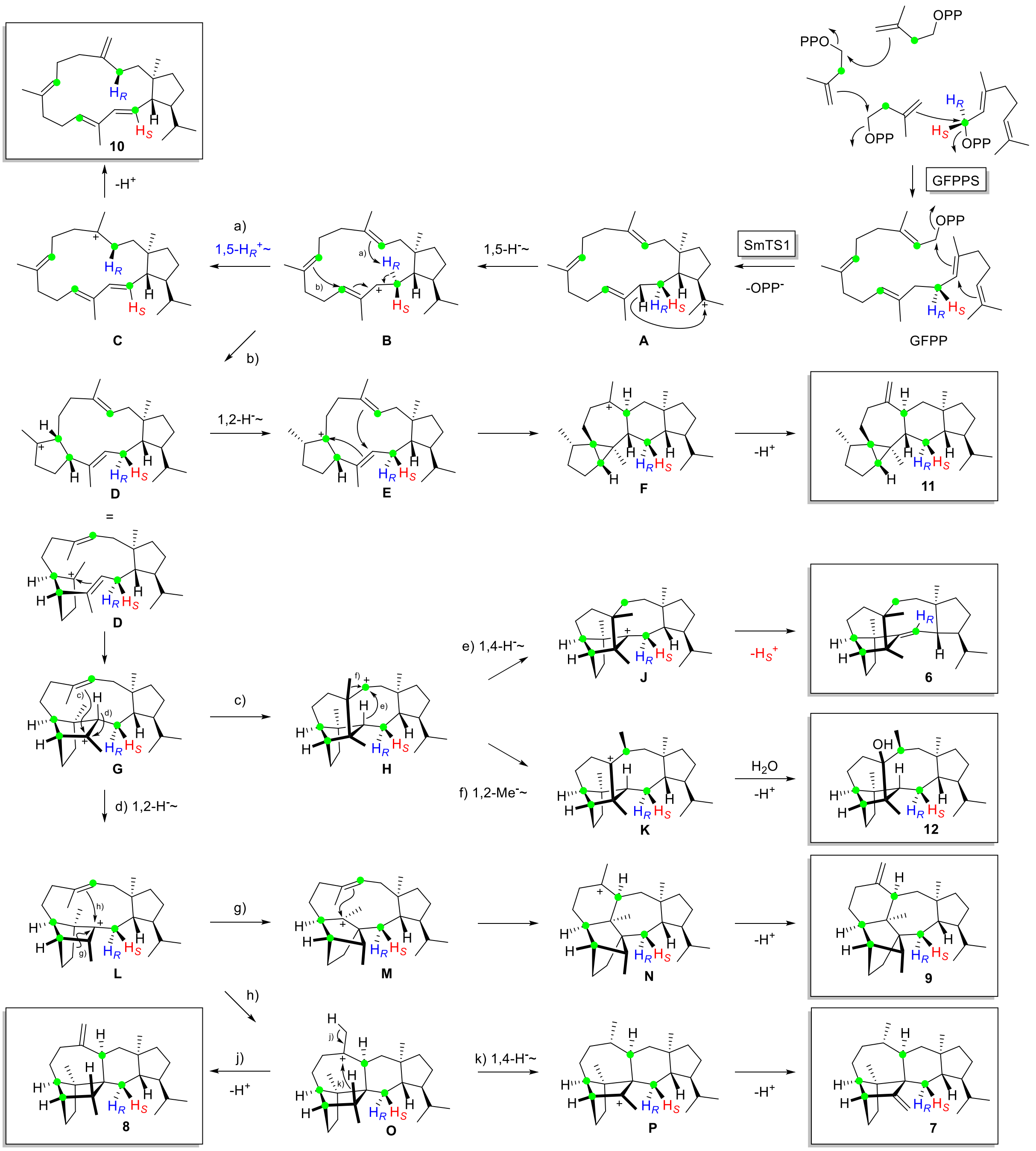

Scheme S2. Biosynthesis of labeled **6 – 12** from (*R*)- or (*S*)-(1-^{13}C,1-^{2}H)GPP and (2-^{13}C)IPP with GFPPS and SmTS1.

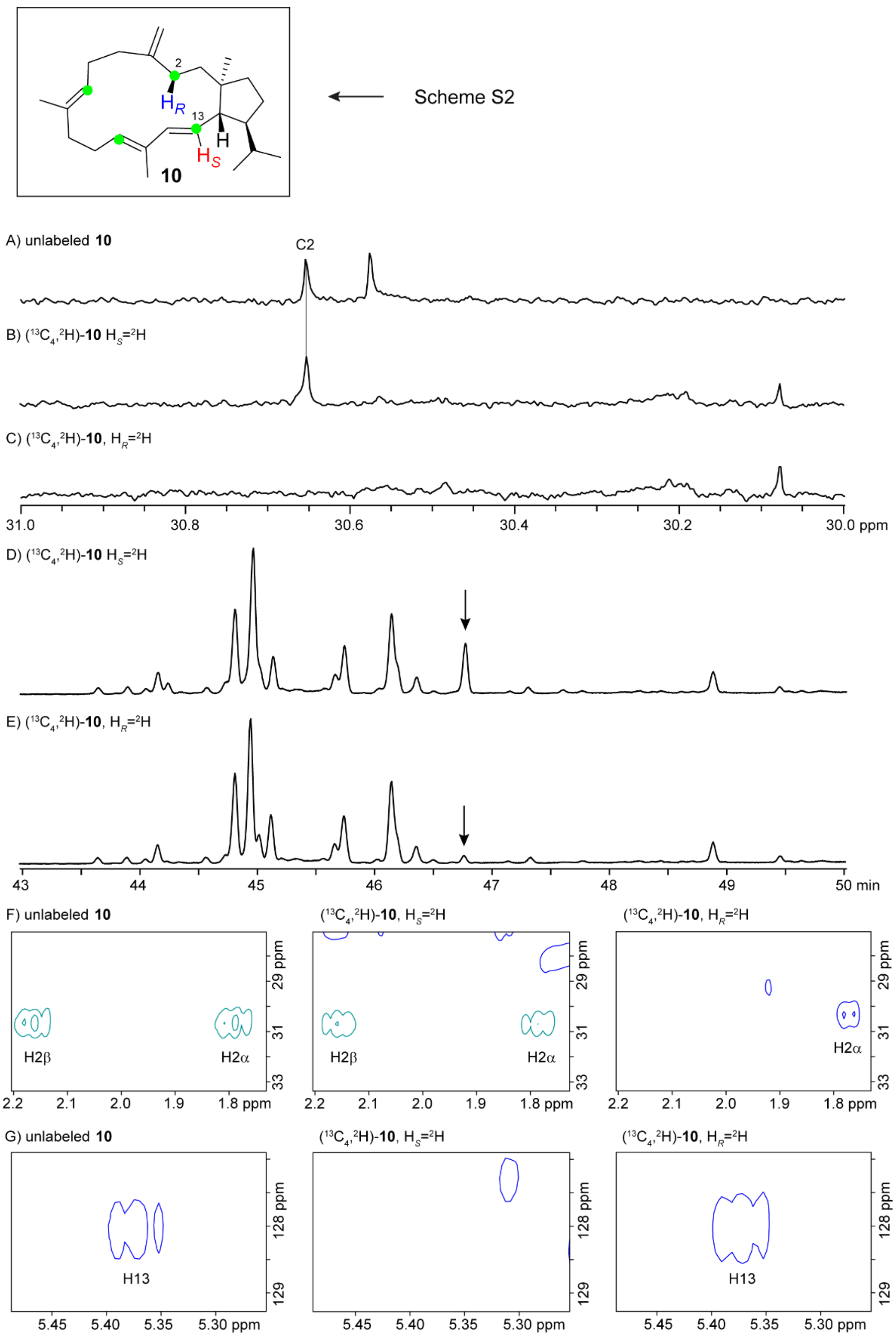

Figure S95. 1,5-Hydride shift from **B** to **C** in the formation of compound **10**. A) ^{13}C NMR signal for C2 of unlabeled **10**, B) ^{13}C NMR signal for non-deuterated C2 of labeled **10** obtained from (*S*)-(1-^{13}C,1-^2H)GPP with (2-^{13}C)IPP, C) the ^{13}C NMR signal for deuterated C2 of labeled **10** obtained from (*R*)-(1-^{13}C,1-^2H)GPP with (2-^{13}C)IPP could not be observed, D) total ion chromatogram of the products obtained from (*S*)-(1-^{13}C,1-^2H)GPP and (2-^{13}C)IPP, E) from (*R*)-(1-^{13}C,1-^2H)GPP and (2-^{13}C)IPP (peak for **10** is marked by arrow), F) HSQC signals for C2 of unlabeled **10**, labeled **10** (H_S=^2H) and labeled **10** (H_R=^2H, one crosspeak missing, indicating deuteration at C2), G) HSQC signals for C13 of unlabeled **10**, labeled **10** (H_S=^2H, crosspeak missing, indicating deuteration at C13) and labeled **10** (H_R=^2H).

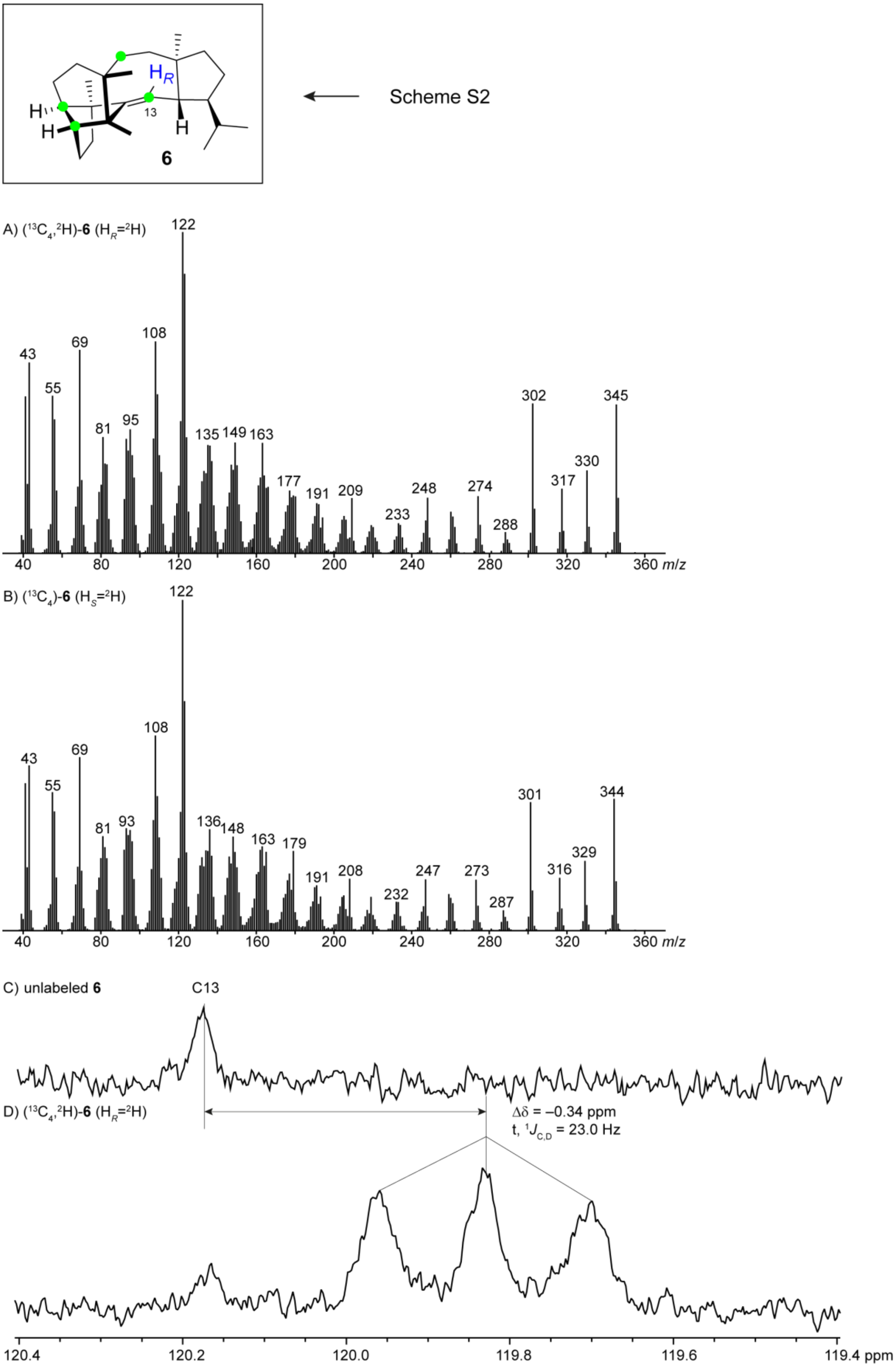

Figure S96. The specific loss of the 1-*pro*-*S* proton of GPP in the final deprotonation step to compound **6**. A) Mass spectrum of compound **6** obtained from (*R*)-(1-^{13}C,1-^{2}H)GPP and (2-^{13}C)IPP, B) mass spectrum of compound **6** obtained from (*S*)-(1-^{13}C,1-^{2}H)GPP and (2-^{13}C)IPP, C) ^{13}C NMR spectrum for C13 of unlabeled **6**, D) ^{13}C NMR spectrum for deuterated C13 of labeled **6** obtained from (*R*)-(1-^{13}C,1-^{2}H)GPP and (2-^{13}C)IPP.

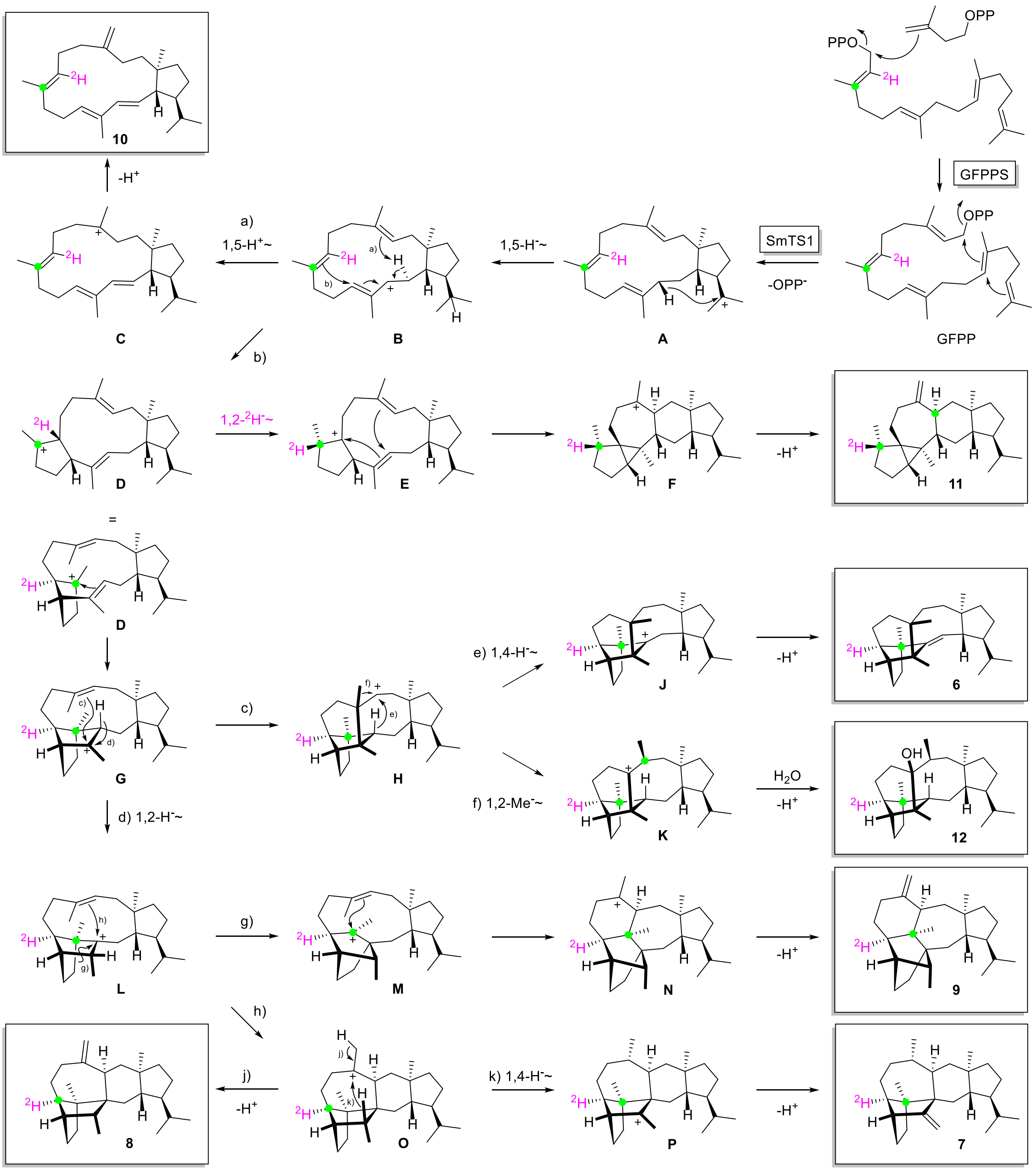

Scheme S3. Biosynthesis of **6 – 12** from (3-^{13}C,2-^{2}H)GGPP and IPP with GFPPS and SmTS1.

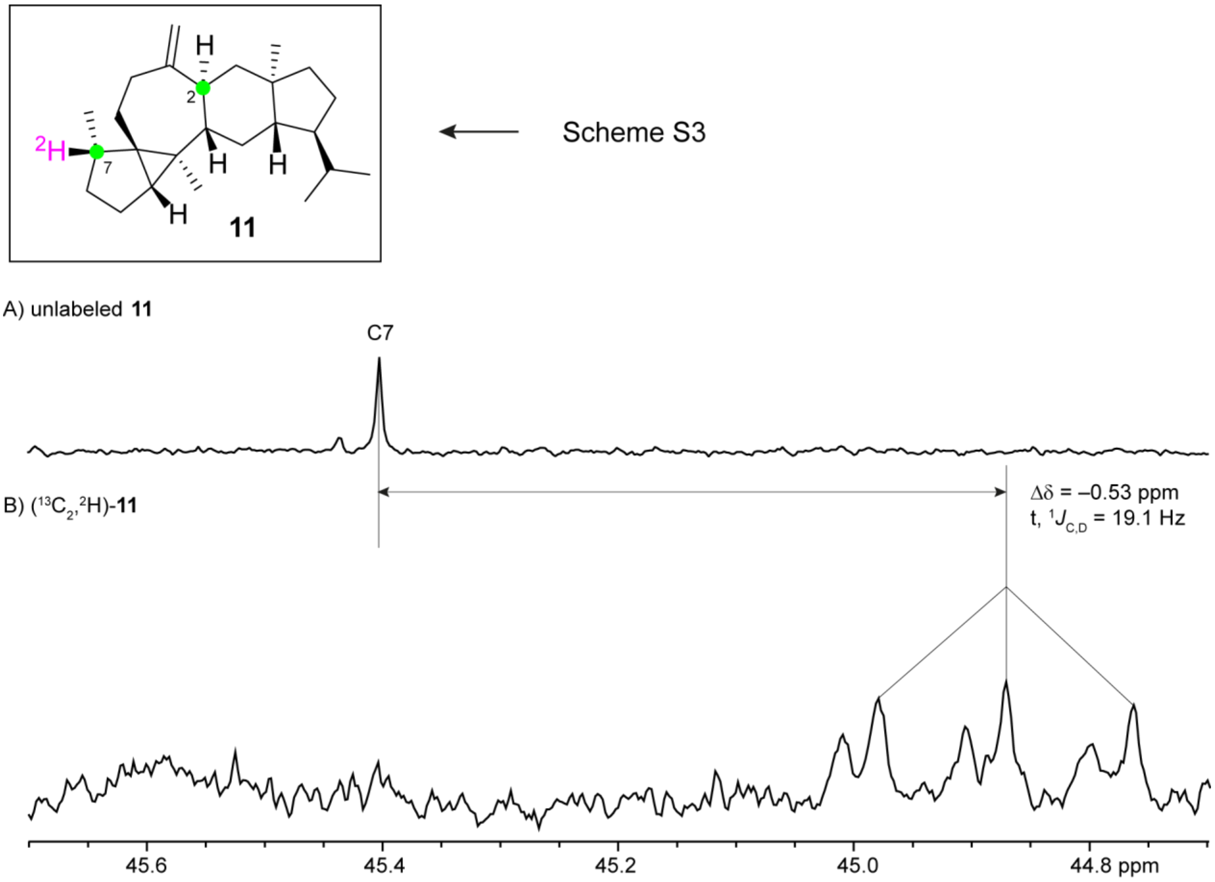

Figure S97. The 1,2-hydride shift from **D** to **E** in the formation of compound **11**. A) ^{13}C NMR spectrum for C7 of unlabeled **11**, B) ^{13}C NMR spectrum for deuterated C7 of **11** obtained from (3-^{13}C,2-^2H)GGPP and IPP.

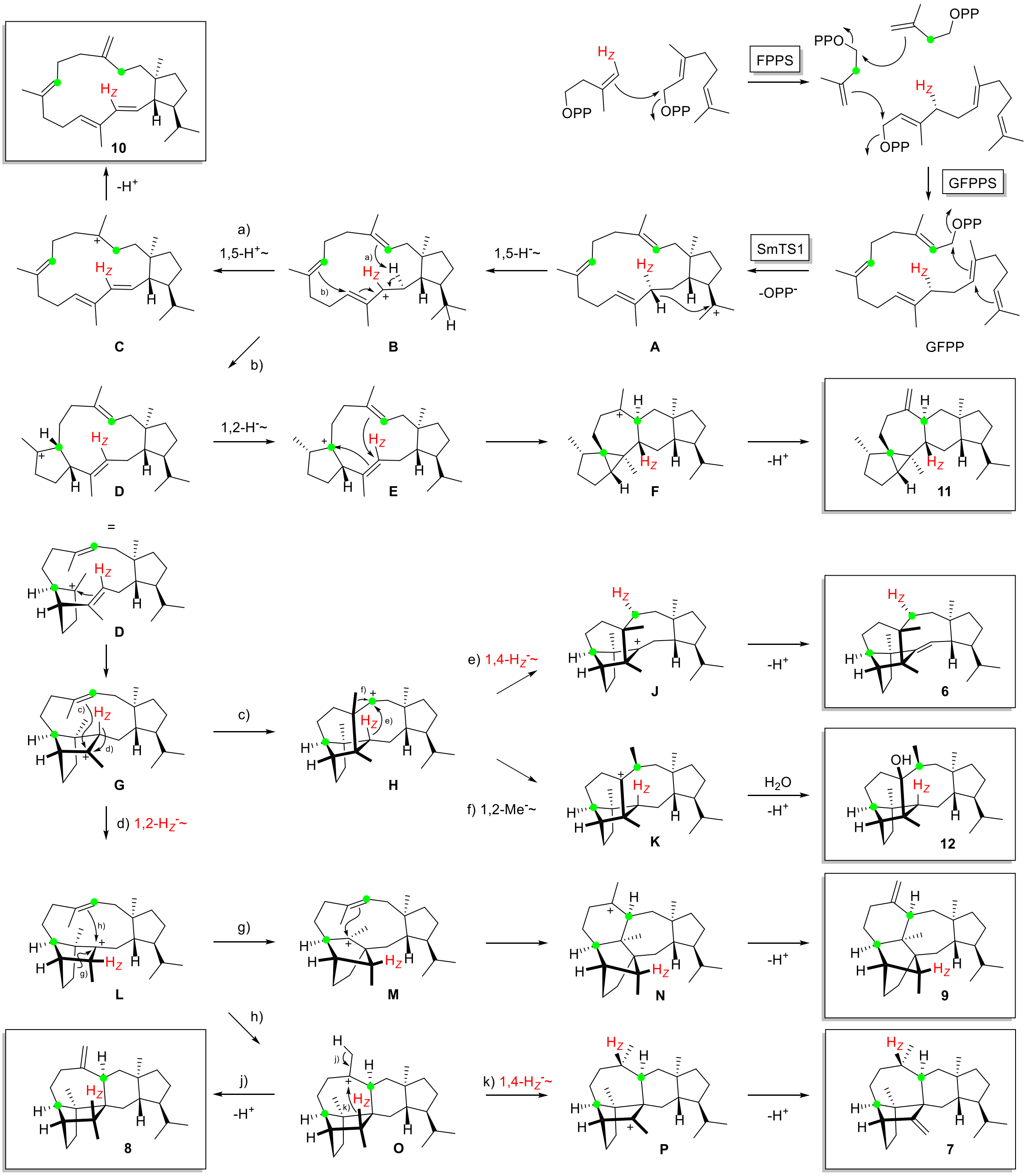

Scheme S4a. Biosynthesis of **6** – **12** from GPP, (*Z*)-(4-^{2}H)IPP and (2-^{13}C)IPP with FPPS, GFPPS and SmTS1, showing the formation of the target isotopomer of **6**.

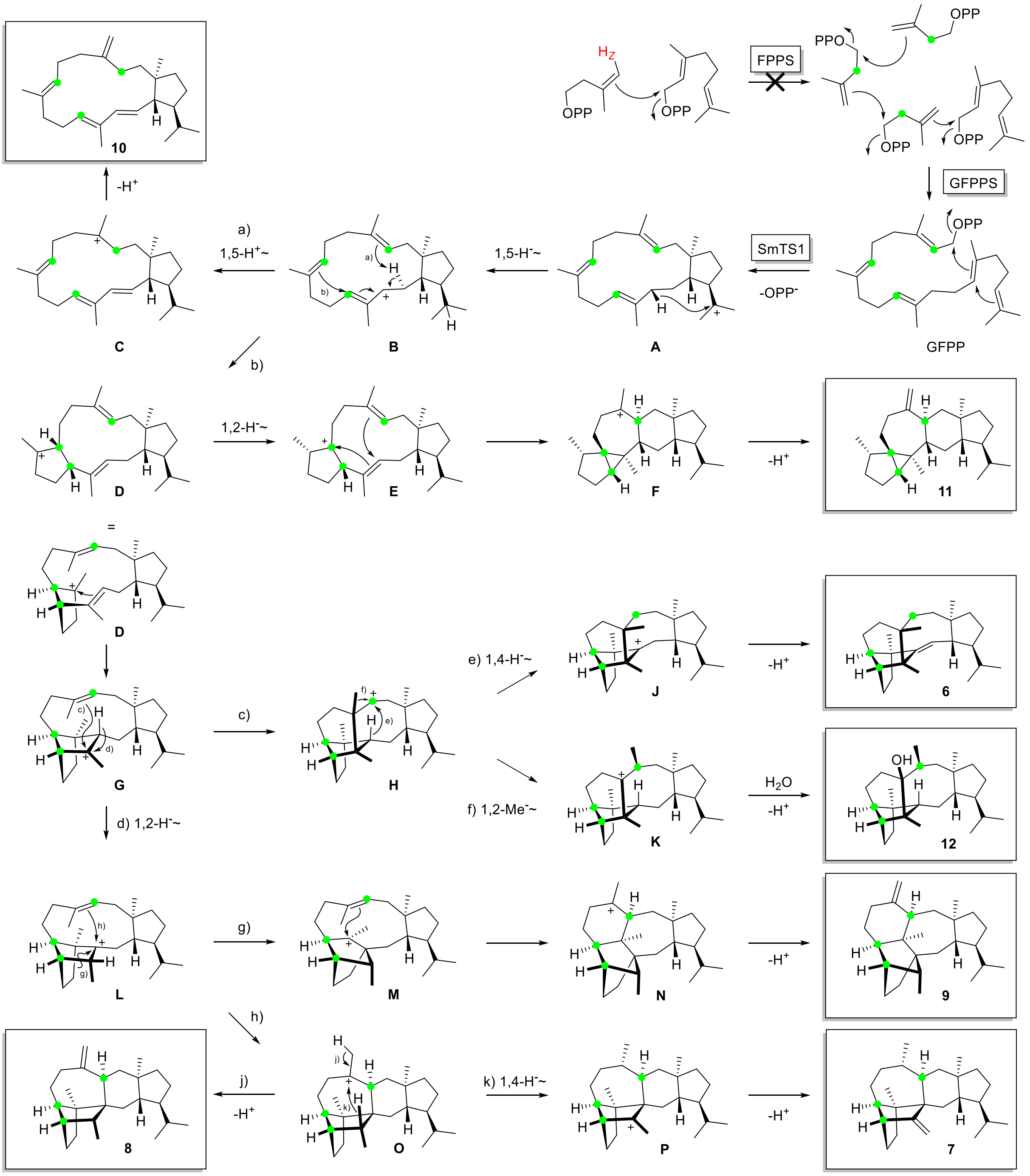

Scheme S4b. Biosynthesis of **6** – **12** from GPP, (*Z*)-(4-^{2}H)IPP and (2-^{13}C)IPP with FPPS, GFPPS and SmTS1. First, the incubation of GPP and (*Z*)-(4-^{2}H)IPP with FPPS was performed, followed by addition of (2-^{13}C)IPP, GFPPS and SmTS1. In side reactions, after the first step unreacted GPP can be elongated with the later added (2-^{13}C)IPP to form other (e. g. as shown here non-deuterated) isotopomers of **6**. Further isotopomers are possible by statistical incorporation of (*Z*)-(4-^{2}H)IPP and (2-^{13}C)IPP.

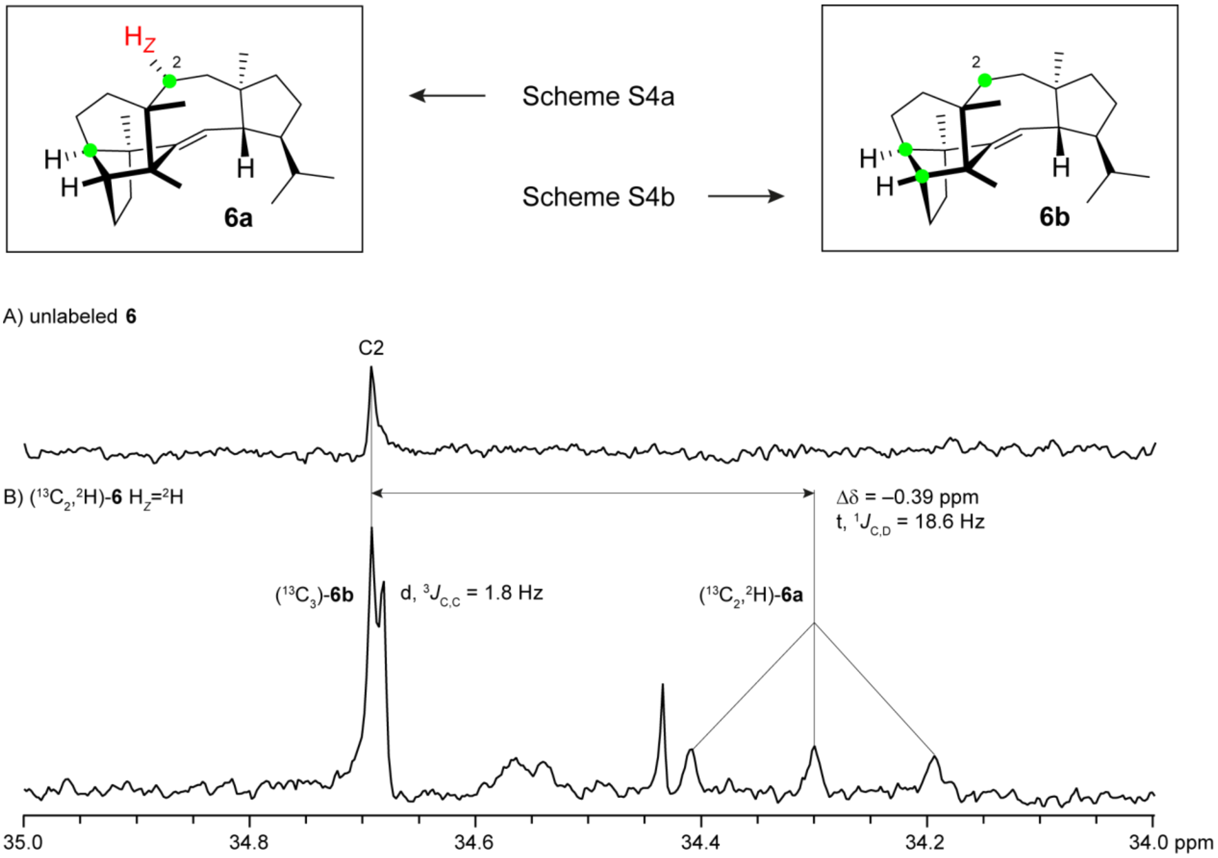

Figure S98. The 1,4-hydride shift from **H** to **J** in the formation of compound **6**. A) ^{13}C NMR spectrum for C2 of unlabeled **6**, B) ^{13}C NMR spectrum of labeled **6** obtained from GPP, (*Z*)-(4-^{2}H)IPP and (2-^{13}C)IPP. The upfield shifted triplet for C2 of **6a** supports the 1,4-hydride shift. Additional signals for C2 of other isotopomers such as **6b** are also observed.

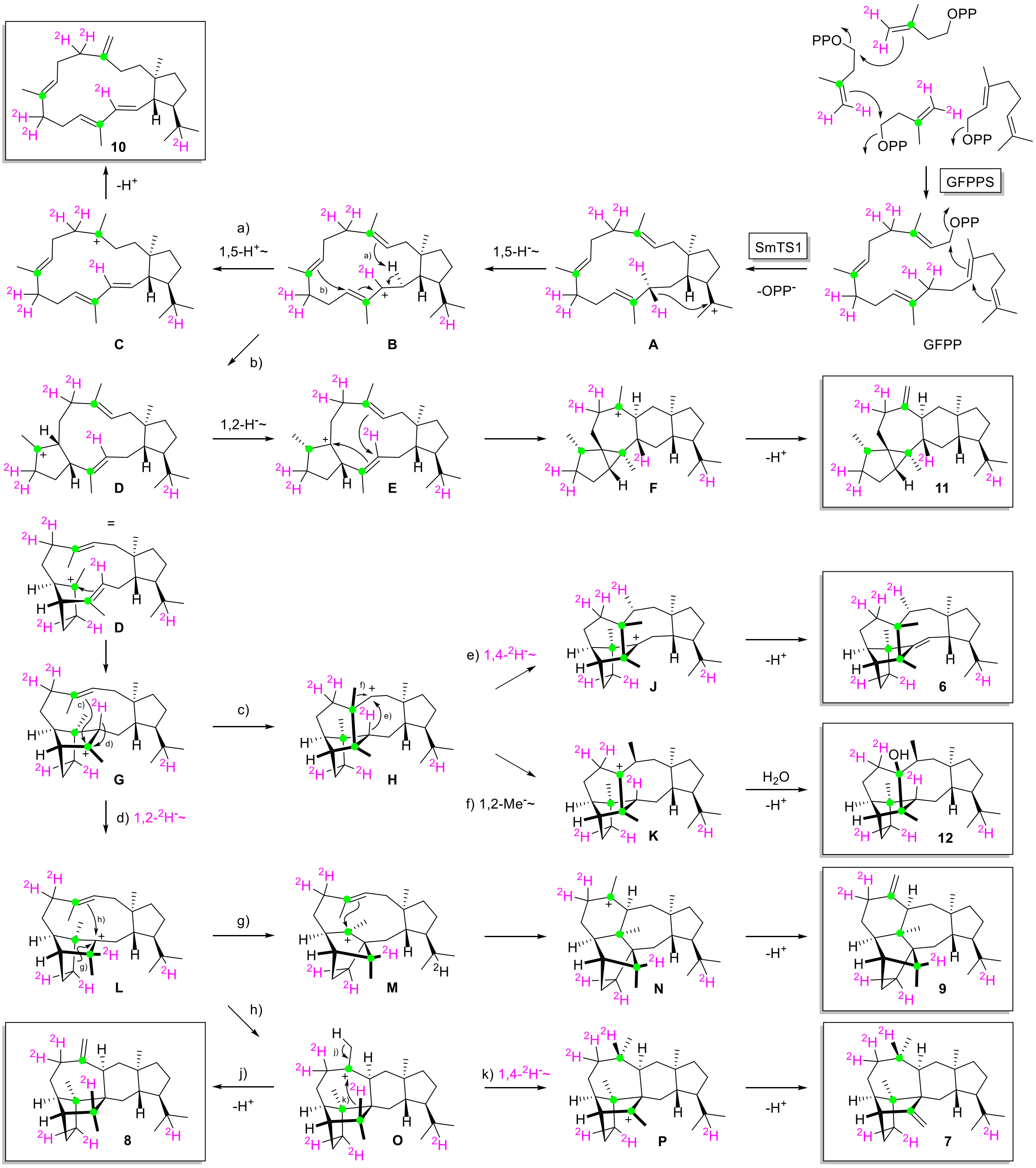

Scheme S5. Biosynthesis of **6 – 12** from GPP and (3-^{13}C,4-$^{2}H_2$)IPP with GFPPS and SmTS1.

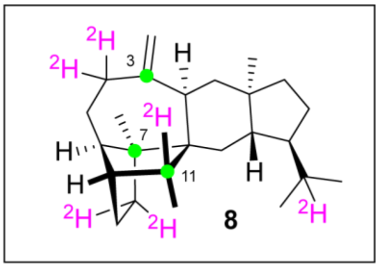

← Scheme S5

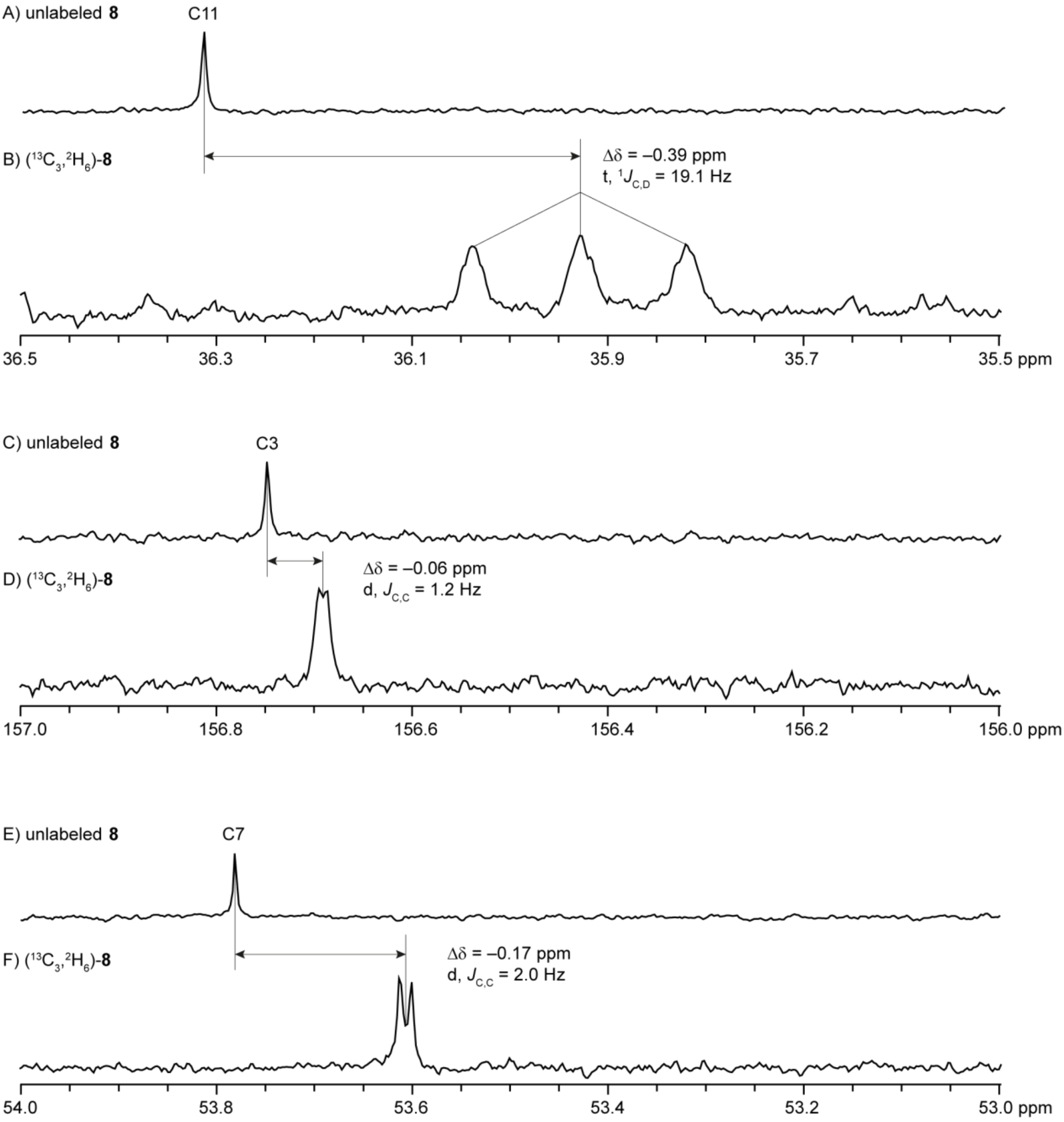

Figure S99. The 1,2-hydride shift from **G** to **L** in the formation of compound **8**. ^{13}C NMR spectra showing the signals for C11 of A) unlabeled **8** and B) labeled **8**, for C3 of C) unlabeled **8** and D) labeled **8**, and for C7 of E) unlabeled **8** and F) labeled **8**. Labeled **8** was obtained from GPP and (3-^{13}C,4-^{2}H$_2$)IPP. The upfield shifted triplet in B) supports the 1,2-hydride shift. The upfield shifts in D) and F) are a result of double deuteration at the neighboring carbon.

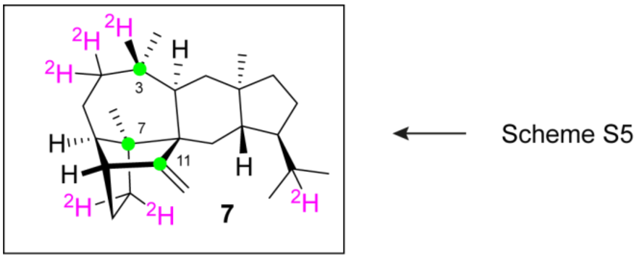

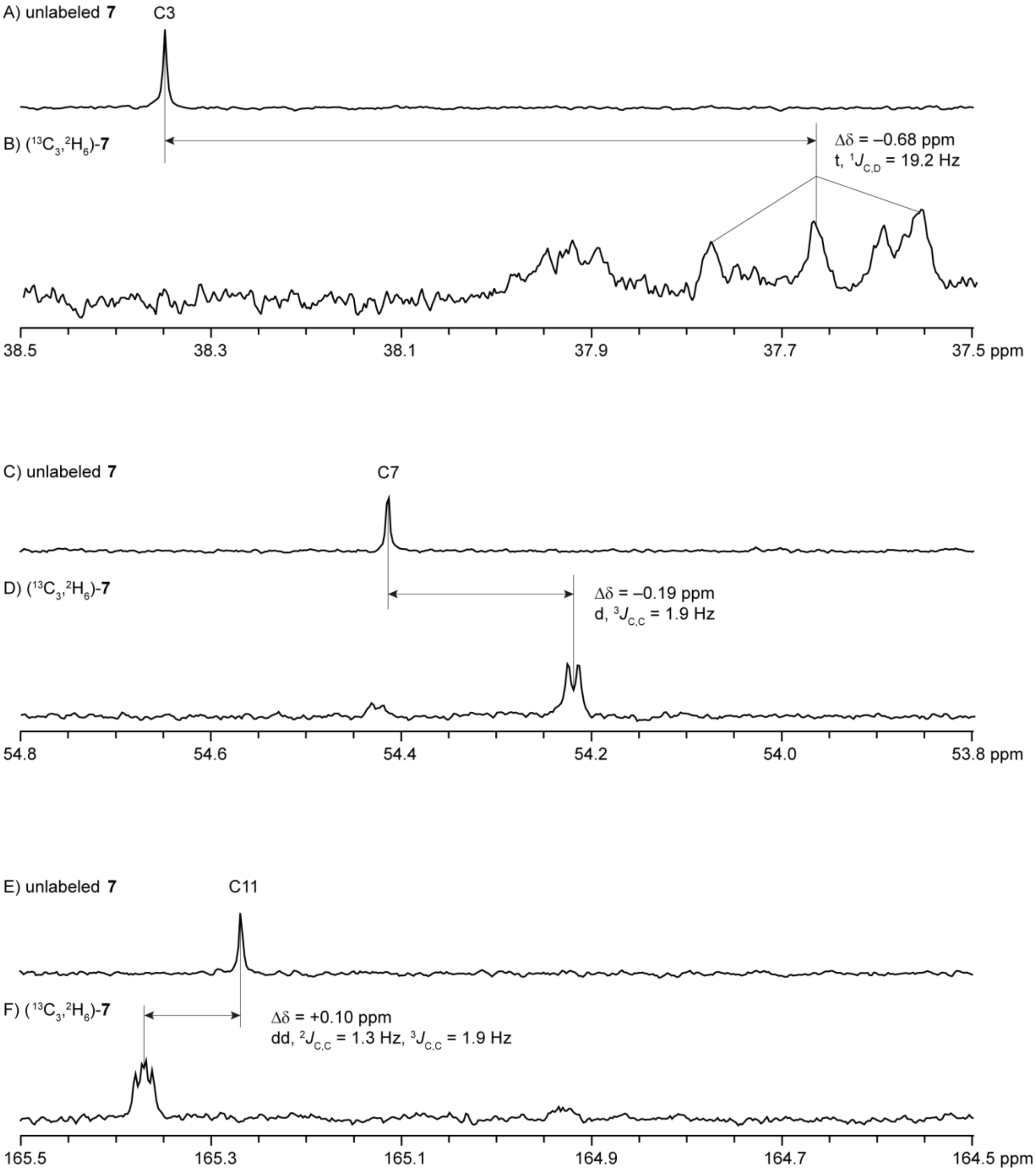

Figure S100. The 1,2-hydride shift from **G** to **L** in the formation of compound **7**. ^{13}C NMR spectra showing the signal for C3 of A) unlabeled **7** and B) labeled **7**, for C7 of C) unlabeled **7** and D) labeled **7**, and for C11 of E) unlabeled **7** and F) labeled **7**. Labeled **7** was obtained from GPP and (3-^{13}C,4-$^{2}H_2$)IPP. The upfield shifted triplet in B) supports the 1,2-hydride shift. The $\Delta\delta$ = –0.68 is a result of one directly bound deuterium at C3 and two deuterium atoms at the neigboring carbon C4.

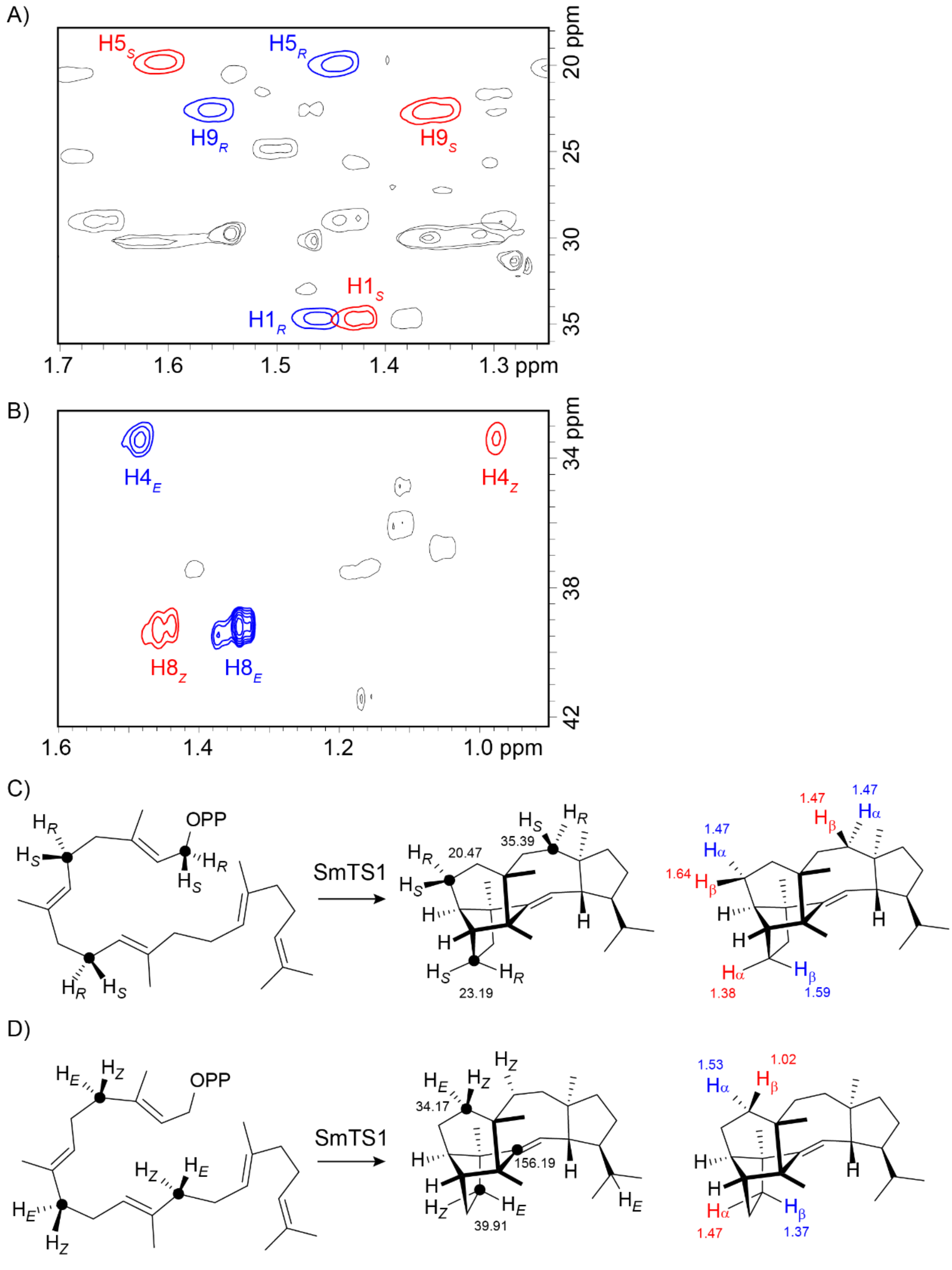

Figure S101. Determination of the absolute configuration of **6**. A) Overlaid HSQC spectra from two labeling experiments using GPP with (*S*)- or (*R*)-(1-^{13}C,1-^{2}H)IPP. The experiment with (*S*)-(1-^{13}C,1-^{2}H)IPP resulted in vanished crosspeaks for $H1_S$, $H5_S$ and $H9_S$, but crosspeaks for $H1_R$, $H5_R$ and $H9_R$ were detected (blue), while in the experiment with (*R*)-(1-^{13}C,1-^{2}H)IPP the red crosspeaks were observed. B) Overlaid HSQC spectra from two labeling experiments using GPP with (*Z*)- or (*E*)-(4-^{13}C,4-^{2}H)IPP. The experiment with (*Z*)-(4-^{13}C,4-^{2}H)IPP resulted in vanished crosspeaks for $H4_Z$ and $H8_Z$, but crosspeaks for $H4_E$ and $H8_E$ were detected (blue), while in the experiment with (*E*)-(4-^{13}C,4-^{2}H)IPP the red crosspeaks were observed. Figures C) and D) show the GFPP isotopomers with the stereogenic anchors at the labeled carbons of known absolute configuration and their conversion into labeled **6**, which together with the NOESY based assignments of diastereotopic hydrogens at C1, C4, C5, C8 and C9 indicates the absolute configuration of **6** as shown.

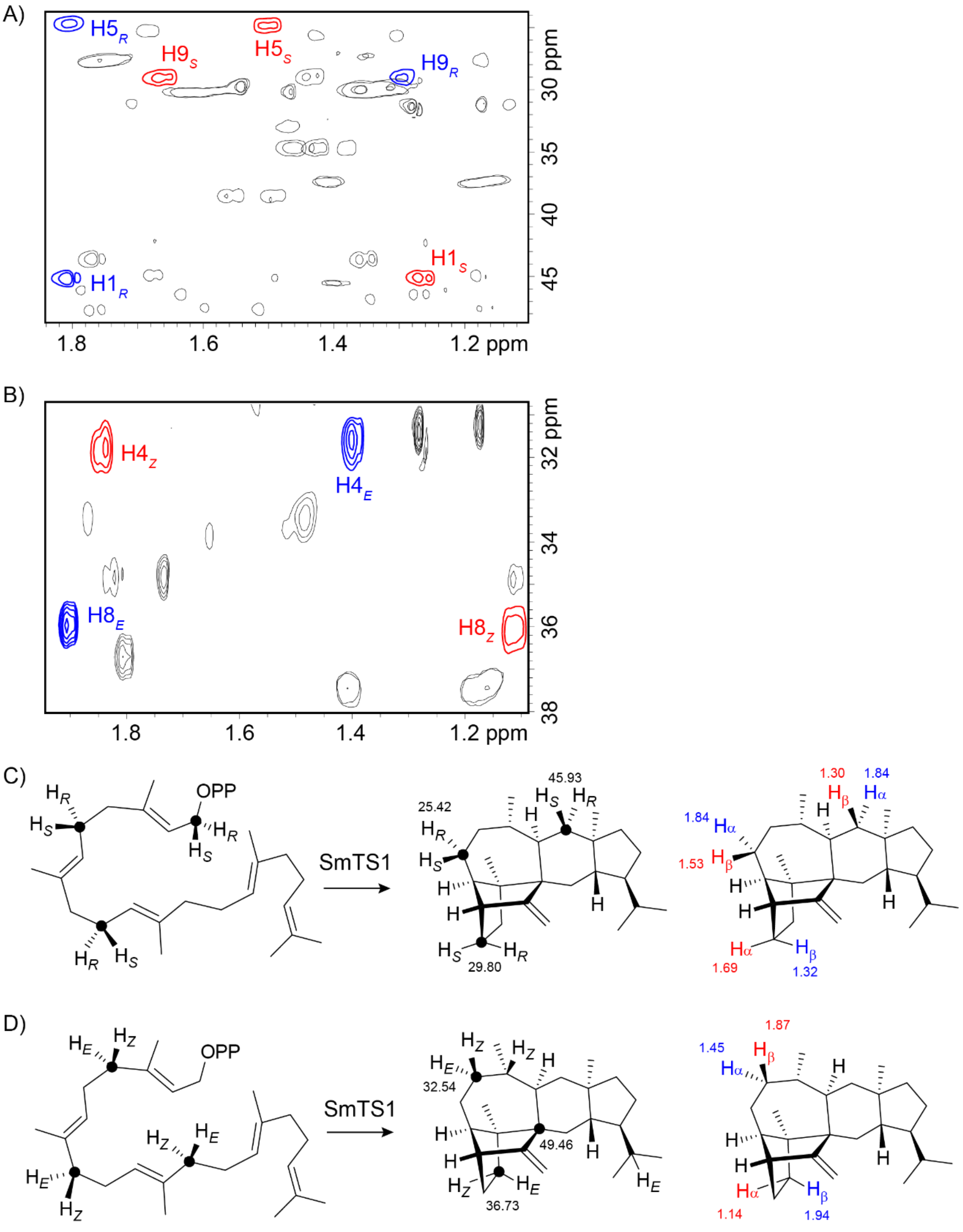

Figure S102. Determination of the absolute configuration of **7**. A) Overlaid HSQC spectra from two labeling experiments using GPP with (*S*)- or (*R*)-(1-^{13}C,1-^{2}H)IPP. The experiment with (*S*)-(1-^{13}C,1-^{2}H)IPP resulted in vanished crosspeaks for $H1_S$, $H5_S$ and $H9_S$, but crosspeaks for $H1_R$, $H5_R$ and $H9_R$ were detected (blue), while in the experiment with (*R*)-(1-^{13}C,1-^{2}H)IPP the red crosspeaks were observed. B) Overlaid HSQC spectra from two labeling experiments using GPP with (*Z*)- or (*E*)-(4-^{13}C,4-^{2}H)IPP. The experiment with (*Z*)-(4-^{13}C,4-^{2}H)IPP resulted in vanished crosspeaks for $H4_Z$ and $H8_Z$, but crosspeaks for $H4_E$ and $H8_E$ were detected (blue), while in the experiment with (*E*)-(4-^{13}C,4-^{2}H)IPP the red crosspeaks were observed. Figures C) and D) show the GFPP isotopomers with the stereogenic anchors at the labeled carbons of known absolute configuration and their conversion into labeled **7**, which together with the NOESY based assignments of diastereotopic hydrogens at C1, C4, C5, C8 and C9 indicates the absolute configuration of **7** as shown.

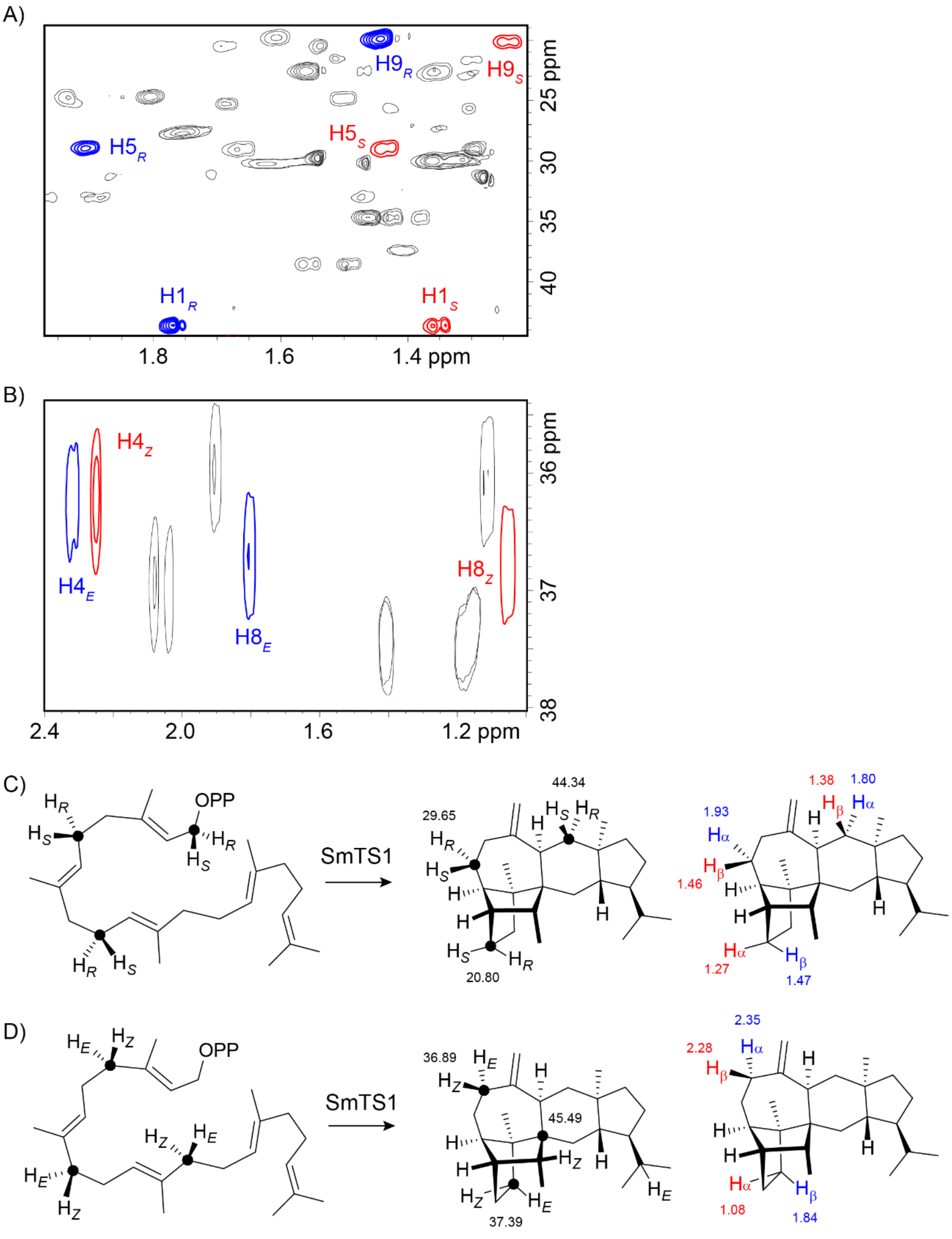

Figure S103. Determination of the absolute configuration of **8**. A) Overlaid HSQC spectra from two labeling experiments using GPP with (*S*)- or (*R*)-(1-^{13}C,1-^{2}H)IPP. The experiment with (*S*)-(1-^{13}C,1-^{2}H)IPP resulted in vanished crosspeaks for H1$_S$, H5$_S$ and H9$_S$, but crosspeaks for H1$_R$, H5$_R$ and H9$_R$ were detected (blue), while in the experiment with (*R*)-(1-^{13}C,1-^{2}H)IPP the red crosspeaks were observed. B) Overlaid HSQC spectra from two labeling experiments using GPP with (*Z*)- or (*E*)-(4-^{13}C,4-^{2}H)IPP. The experiment with (*Z*)-(4-^{13}C,4-^{2}H)IPP resulted in vanished crosspeaks for H4$_Z$ and H8$_Z$, but crosspeaks for H4$_E$ and H8$_E$ were detected (blue), while in the experiment with (*E*)-(4-^{13}C,4-^{2}H)IPP the red crosspeaks were observed. Figures C) and D) show the GFPP isotopomers with the stereogenic anchors at the labeled carbons of known absolute configuration and their conversion into labeled **8**, which together with the NOESY based assignments of diastereotopic hydrogens at C1, C4, C5, C8 and C9 indicates the absolute configuration of **8** as shown.

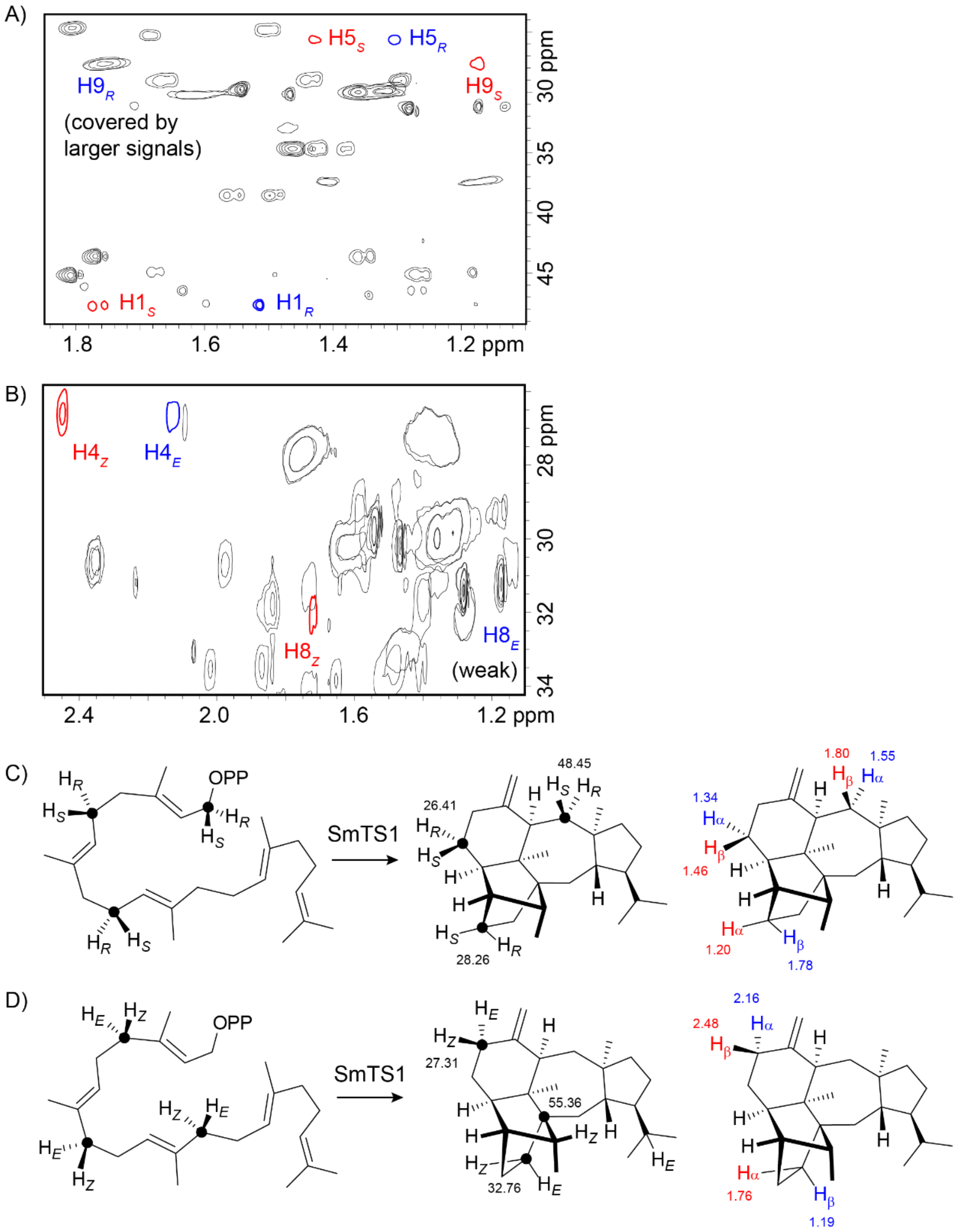

Figure S104. Determination of the absolute configuration of **9**. A) Overlaid HSQC spectra from two labeling experiments using GPP with (*S*)- or (*R*)-(1-^{13}C,1-^{2}H)IPP. The experiment with (*S*)-(1-^{13}C,1-^{2}H)IPP resulted in vanished crosspeaks for H1$_S$, H5$_S$ and H9$_S$, but crosspeaks for H1$_R$ and H5$_R$ were detected (blue, signal for H9$_R$ was covered), while in the experiment with (*R*)-(1-^{13}C,1-^{2}H)IPP the red crosspeaks were observed. B) Overlaid HSQC spectra from two labeling experiments using GPP with (*Z*)- or (*E*)-(4-^{13}C,4-^{2}H)IPP. The experiment with (*Z*)-(4-^{13}C,4-^{2}H)IPP resulted in vanished crosspeaks for H4$_Z$ and H8$_Z$, but the crosspeaks for H4$_E$ was detected (blue, H8$_E$ was weak), while in the experiment with (*E*)-(4-^{13}C,4-^{2}H)IPP the red crosspeaks were observed. Figures C) and D) show the GFPP isotopomers with the stereogenic anchors at the labeled carbons of known absolute configuration and their conversion into labeled **8**, which together with the NOESY based assignments of diastereotopic hydrogens at C1, C4, C5, C8 and C9 indicates the absolute configuration of **8** as shown.

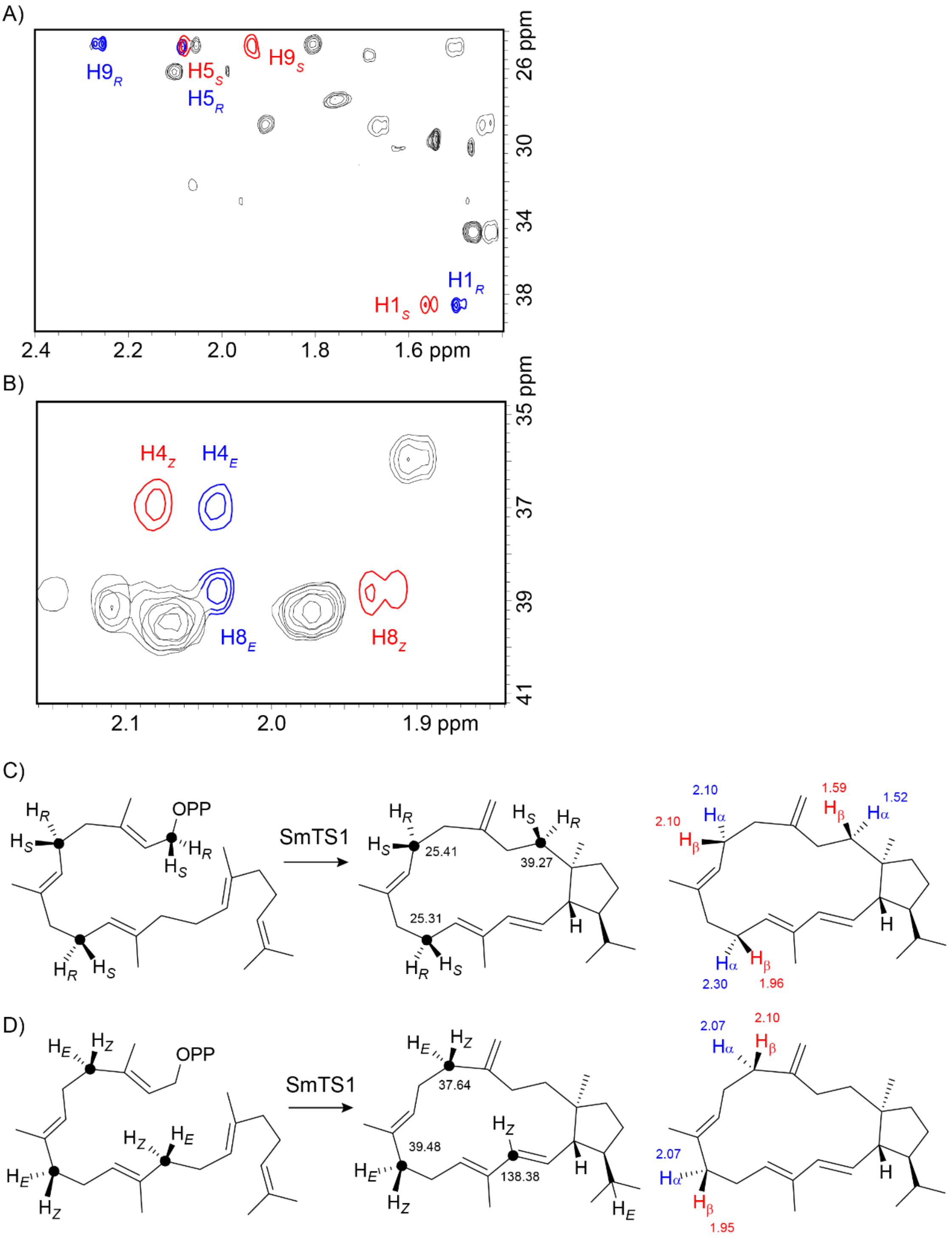

Figure S105. Determination of the absolute configuration of **10**. A) Overlaid HSQC spectra from two labeling experiments using GPP with (*S*)- or (*R*)-(1-^{13}C,1-^{2}H)IPP. The experiment with (*S*)-(1-^{13}C,1-^{2}H)IPP resulted in vanished crosspeaks for $H1_S$, $H5_S$ and $H9_S$, but crosspeaks for $H1_R$, $H5_R$ and $H9_R$ were detected (blue), while in the experiment with (*R*)-(1-^{13}C,1-^{2}H)IPP the red crosspeaks were observed. B) Overlaid HSQC spectra from two labeling experiments using GPP with (*Z*)- or (*E*)-(4-^{13}C,4-^{2}H)IPP. The experiment with (*Z*)-(4-^{13}C,4-^{2}H)IPP resulted in vanished crosspeaks for $H4_Z$ and $H8_Z$, but crosspeaks for $H4_E$ and $H8_E$ were detected (blue), while in the experiment with (*E*)-(4-^{13}C,4-^{2}H)IPP the red crosspeaks were observed. Figures C) and D) show the GFPP isotopomers with the stereogenic anchors at the labeled carbons of known absolute configuration and their conversion into labeled **10**, which together with the NOESY based assignments of diastereotopic hydrogens at C1, C4, C5, C8 and C9 indicates the absolute configuration of **10** as shown.

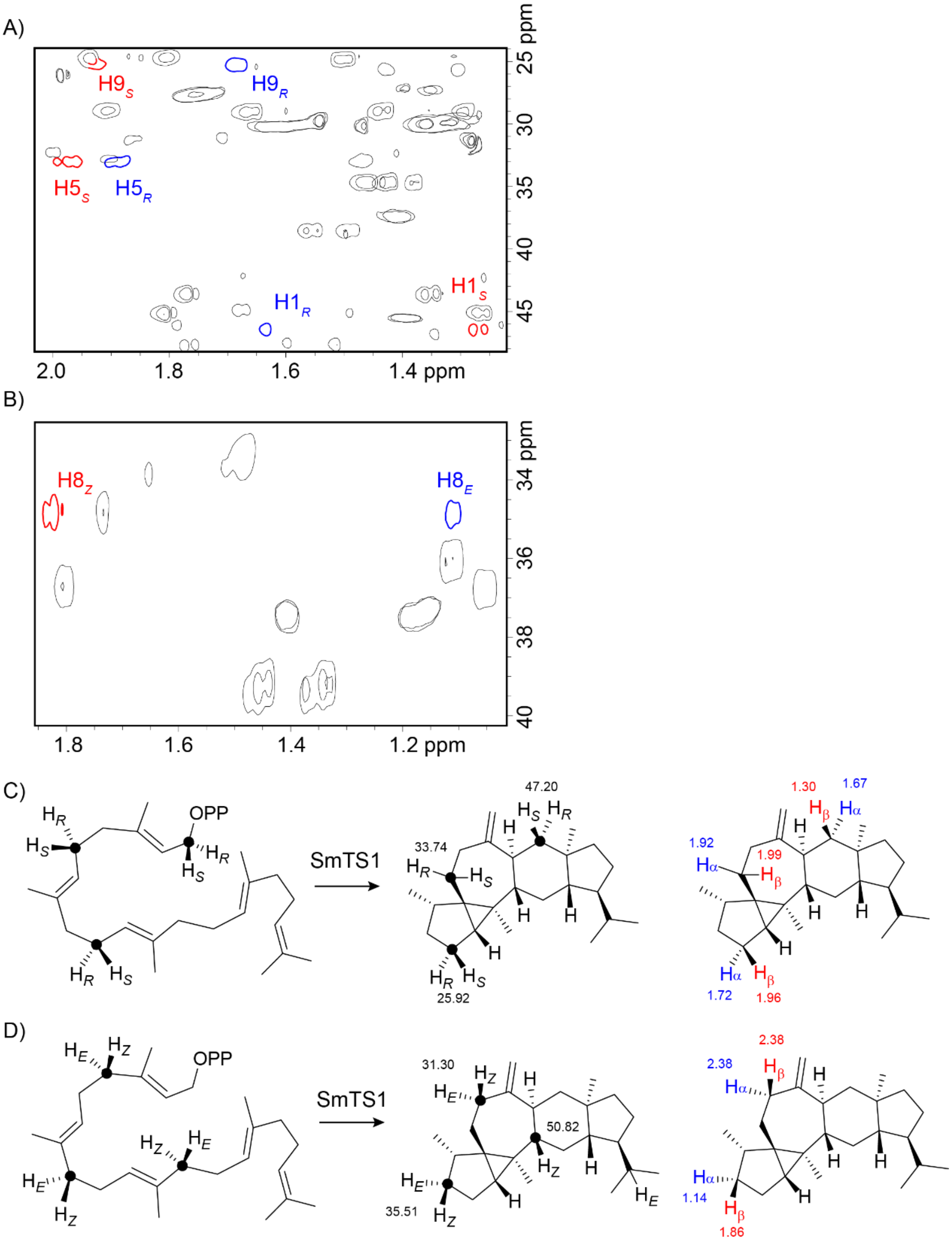

Figure S106. Determination of the absolute configuration of **11**. A) Overlaid HSQC spectra from two labeling experiments using GPP with (*S*)- or (*R*)-(1-^{13}C,1-^{2}H)IPP. The experiment with (*S*)-(1-^{13}C,1-^{2}H)IPP resulted in vanished crosspeaks for H1$_S$, H5$_S$ and H9$_S$, but crosspeaks for H1$_R$, H5$_R$ and H9$_R$ were detected (blue), while in the experiment with (*R*)-(1-^{13}C,1-^{2}H)IPP the red crosspeaks were observed. B) Overlaid HSQC spectra from two labeling experiments using GPP with (*Z*)- or (*E*)-(4-^{13}C,4-^{2}H)IPP. The experiment with (*Z*)-(4-^{13}C,4-^{2}H)IPP resulted in vanished crosspeaks for H4$_Z$ and H8$_Z$, but crosspeaks for H4$_E$ and H8$_E$ were detected (blue), while in the experiment with (*E*)-(4-^{13}C,4-^{2}H)IPP the red crosspeaks were observed. Figures C) and D) show the GFPP isotopomers with the stereogenic anchors at the labeled carbons of known absolute configuration and their conversion into labeled **11**, which together with the NOESY based assignments of diastereotopic hydrogens at C1, C4, C5, C8 and C9 indicates the absolute configuration of **11** as shown.

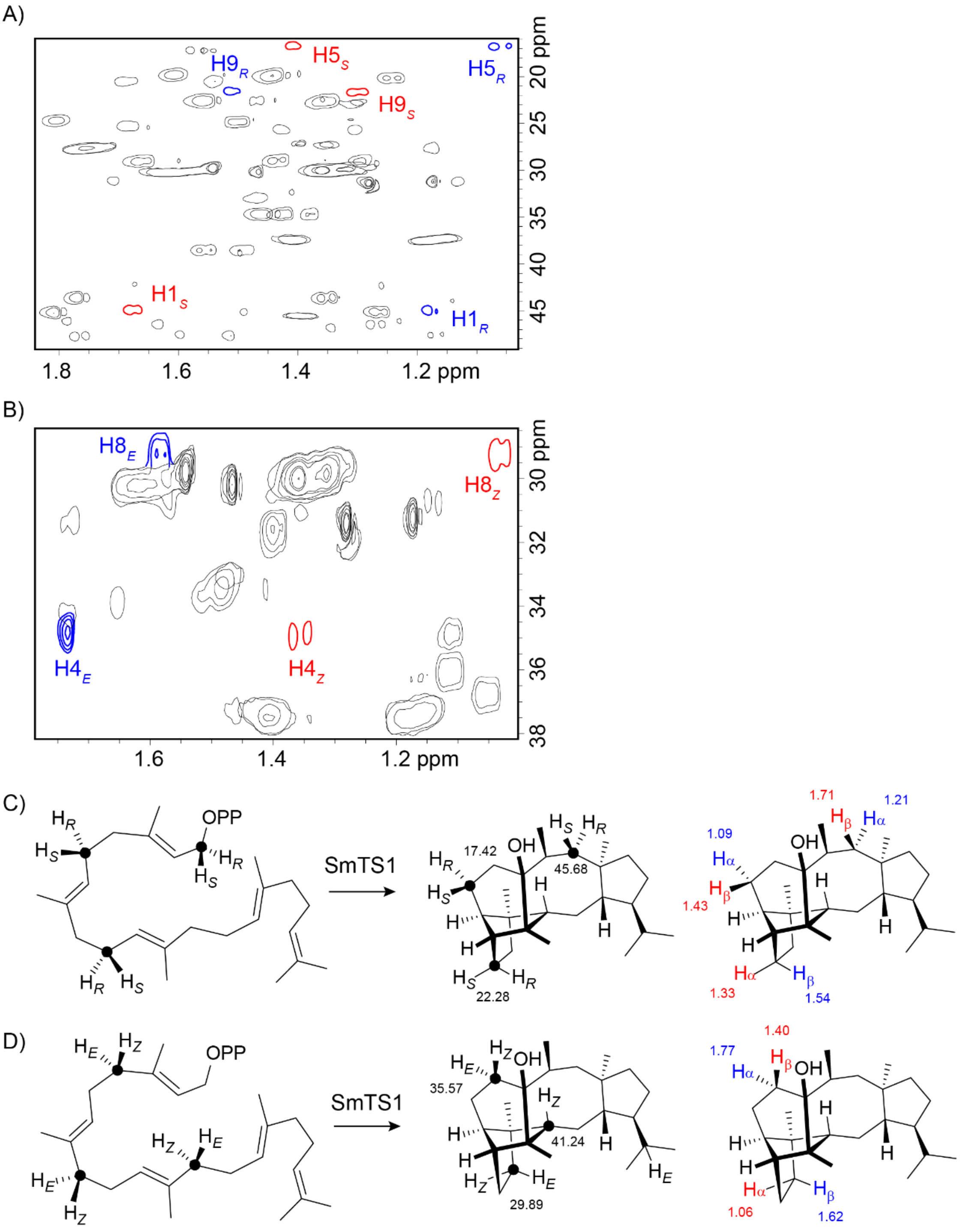

Figure S107. Determination of the absolute configuration of **12**. A) Overlaid HSQC spectra from two labeling experiments using GPP with (*S*)- or (*R*)-(1-^{13}C,1-^{2}H)IPP. The experiment with (*S*)-(1-^{13}C,1-^{2}H)IPP resulted in vanished crosspeaks for $H1_S$, $H5_S$ and $H9_S$, but crosspeaks for $H1_R$, $H5_R$ and $H9_R$ were detected (blue), while in the experiment with (*R*)-(1-^{13}C,1-^{2}H)IPP the red crosspeaks were observed. B) Overlaid HSQC spectra from two labeling experiments using GPP with (*Z*)- or (*E*)-(4-^{13}C,4-^{2}H)IPP. The experiment with (*Z*)-(4-^{13}C,4-^{2}H)IPP resulted in vanished crosspeaks for $H4_Z$ and $H8_Z$, but crosspeaks for $H4_E$ and $H8_E$ were detected (blue), while in the experiment with (*E*)-(4-^{13}C,4-^{2}H)IPP the red crosspeaks were observed. Figures C) and D) show the GFPP isotopomers with the stereogenic anchors at the labeled carbons of known absolute configuration and their conversion into labeled **12**, which together with the NOESY based assignments of diastereotopic hydrogens at C1, C4, C5, C8 and C9 indicates the absolute configuration of **12** as shown.

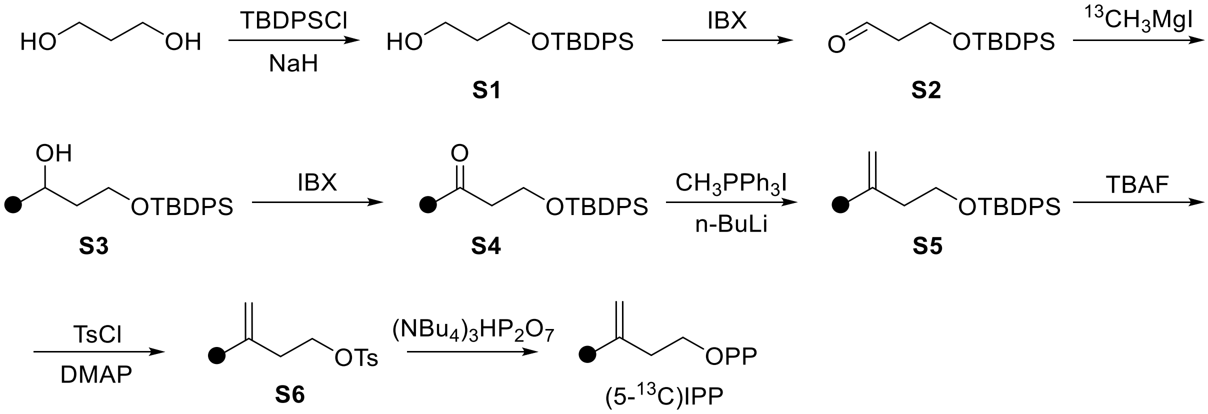

Scheme S6. The synthetic route towards (5-^{13}C)IPP.

Synthesis of (5-^{13}C)IPP

3-((*tert*-Butyldiphenylsilyl)oxy)propanol (S1)

To a suspension of NaH (60% in mineral oil, 1.0 g, 25.0 mmol, 1.0 eq) in THF (25 mL) was slowly added 1,3-propanediol (1.9 g, 25.0 mmol, in 12.5 mL THF) at 0 °C. The reaction mixture was stirred for 45 min at room temperature. Then TBDPSCl (6.87 g, 25 mmol, 1.0 eq, in 12.5 mL THF) was added dropwise, and the reaction mixture was stirred for 1.5 h at room temperature. The reaction was quenched by adding sat. $NaHCO_3$ (75 ml) at 0 °C, followed by extraction with Et_2O (3 x 100 mL). The combined organic layers were dried with $MgSO_4$ and concentrated under reduced pressure. The product **S1** (6.4 g, 20.4 mmol, 82%) was obtained via silica gel chromatography [cyclohexane/EA (4/1), R_f =0.40] as a colorless oil.

3-((*tert*-Butyldiphenylsilyl)oxy)propanol S1. ^{1}H NMR (400 MHz, $CDCl_3$): δ = 7.72 – 7.66 (m, 4H, 4 x CH), 7.47 – 7.38 (m, 6H, 6 x CH), 3.89 – 3.82 (m, 4H, 2 x CH_2), 2.33 (s, 1H, OH), 1.82 (p, $^3J_{H,H}$ = 5.7 Hz, 2H, CH_2), 1.07 (s, 9H, 3 x CH_3). ^{13}C NMR (101 MHz, $CDCl_3$): δ = 135.67 (4 x CH), 133.38 (2 x C_q), 129.90 (2 x CH), 127.88 (4 x CH), 63.37 (CH_2), 62.04 (CH_2), 34.41 (CH_2), 26.97 (3 x CH_3), 19.22 (C_q). GC (HP-5MS): *I* = 2200. MS (EI, 70 eV): *m*/*z* (%) = 257 (28), 239 (1), 229 (10), 211 (5), 199 (100), 179 (96), 167 (2), 149 (6), 139 (10), 121 (7), 117 (7), 105 (7), 91 (11), 77 (14), 57 (2), 45 (4).

3-((*tert*-Butyldiphenylsilyl)oxy)propanal (S2)

To a solution of IBX (6.72 g, 24.4 mmol, 1.2 eq) in DMSO (62.5 mL) was added **S1** (6.4 g, 20.4 mmol, in 12.5 mL DMSO) dropwise at room temperature. The reaction mixture was stirred overnight. Et_2O (100 mL) was added, followed by cooling to 0 °C and addition of sat. $NaHCO_3$ (200 mL). The organic layer was separated and the aqueous layer was extracted with Et_2O (2 x 100 mL). The combined organic layers were dried with $MgSO_4$ and concentrated under reduced pressure. Purification via silica gel chromatography [cyclohexane/EA (10/1), R_f =0.46] afforded **S2** (5.63 g, 18.0 mmol, 88%) as a colorless oil.

3-((*tert*-Butyldiphenylsilyl)oxy)propanal S2. ^{1}H NMR (400 MHz, $CDCl_3$): δ = 9.82 (t, $^3J_{H,H}$ = 2.1 Hz, 1H, CH), 7.69 – 7.64 (m, 4H, 4x CH), 7.45 – 7.38 (m, 6H, 6 x CH), 4.03 (t, $^3J_{H,H}$ = 6.0 Hz, 2H, CH_2), 2.61 (td, $^3J_{H,H}$ = 6.0, 2.2 Hz, 2H, CH_2), 1.05 (s, 9H, 3 x CH_3). ^{13}C NMR (101 MHz, $CDCl_3$): δ = 202.03 (CH), 135.68 (4 x CH), 133.38 (2 x C_q), 129.95 (2 x CH), 127.90 (4 x CH), 58.44 (CH_2), 46.52 (CH_2), 26.89 (3 x CH_3), 19.29 (C_q). GC (HP-5MS): *I* = 2135. MS (EI, 70 eV): *m*/*z* (%) = 255 (75), 225 (79), 211 (22), 199 (25), 183 (100), 177 (62), 147 (10), 135 (8), 117 (72), 105 (22), 100 (5), 91 (12), 77 (20), 57 (4), 45 (9).

(1-^{13}C)-4-((*tert*-Butyldiphenylsilyl)oxy)butan-2-ol (S3)

Magnesium turnings (0.38 g, 15.84 mmol, 1.1 eq) were covered with Et_2O (5 mL) in an oven-dried flask and a small piece of iodine was added. $^{13}CH_3I$ (2.06 g, 14.42 mmol, 1.0 eq) was added dropwise at a rate to maintain the reaction. The reaction mixture was stirred for 1.5 h at

room temperature. After cooling to 0 °C, **S2** (4.5 g, 14.40 mmol, in 20 mL Et_2O) was added, followed by stirring at room temperature for 4 h. The reaction was quenched by the addition of sat. NH_4Cl (100 mL) and extracted with Et_2O (3 x 100 mL). The combined organic layers were dried with $MgSO_4$ and concentrated under reduced pressure. Through silica gel chromatography [cyclohexane/EA (4/1), R_f =0.44], **S3** (3.5 g, 10.65 mmol, 74%) was obtained as a colorless oil.

(1-^{13}C)-4-((*tert*-Butyldiphenylsilyl)oxy)butan-2-ol S3. ^{1}H NMR (400 MHz, C_6D_6): δ = 7.78 – 7.73 (m, 4H, 4 x CH), 7.25 – 7.19 (m, 6H, 6 x CH), 4.02 – 3.88 (m, 1H, CH), 3.83 – 3.66 (m, 2H, CH_2), 2.42 (t, $^3J_{H,H}$ = 3.8 Hz, $^3J_{C,H}$ = 3.8 Hz, 1H, OH), 1.67 – 1.35 (m, 2H, CH_2), 1.12 (s, 9H, 3 x CH_3), 1.08 (dd, $^1J_{C,H}$ = 125.1 Hz, $^3J_{H,H}$ = 6.2 Hz, 3H, CH_3). ^{13}C NMR (101 MHz, C_6D_6): δ = 136.00 (4 x CH), 133.85 (2 x C_q), 130.09 (2 x CH), 128.16 (4 x CH), 66.72 (d, $^1J_{C,C}$ = 39.1 Hz, CH), 63.07 (d, $^3J_{C,C}$ = 3.9 Hz, CH_2), 41.22 (CH_2), 27.07 (3 x CH_3), 23.97 ($^{13}CH_3$), 19.35 (C_q). GC (HP-5MS): *I* = 2199. MS (EI, 70 eV): *m*/*z* (%) = 272 (4), 254 (4), 229 (40), 211 (7), 199 (100), 181 (7), 167 (3), 151 (4), 139 (14), 121 (3), 105 (3), 91 (5), 77 (7), 56 (3), 45 (2).

(1-^{13}C)-4-((*tert*-Butyldiphenylsilyl)oxy)butan-2-one (S4)

Following the same procedure as for the preparation of **S2**, **S3** (3.5 g, 10.65 mmol) was converted into **S4** (2.8 g, 8.55 mmol, 80 %) as a colorless oil.

(1-^{13}C)-4-((*tert*-Butyldiphenylsilyl)oxy)butan-2-one S4. TLC [cyclohexane/EA (10/1)]: R_f = 0.34. ^{1}H NMR (400 MHz, $CDCl_3$): δ = 7.68 – 7.64 (m, 4H, 4 x CH), 7.44 – 7.36 (m, 6H, 6 x CH), 3.94 (t, $^3J_{H,H}$ = 6.3 Hz, 2H, CH_2), 2.64 (t, $^3J_{H,H}$ = 6.2 Hz, 2H, CH_2), 2.19 (d, $^1J_{C,H}$ = 127.2 Hz, 3H, CH_3), 1.04 (s, 9H, 3 x CH_3). ^{13}C NMR (101 MHz, $CDCl_3$): δ = 207.98 (d, $^1J_{C,C}$ = 40.4 Hz, C_q), 135.68 (4 x CH), 133.56 (2 x C_q), 129.85 (2 x CH), 127.84 (4 x CH), 59.86 (CH_2), 46.49 (d, $^2J_{C,C}$ = 13.5 Hz, CH_2), 30.85 ($^{13}CH_3$), 26.92 (3 x CH_3), 19.29 (C_q). GC (HP-5MS): *I* = 2209. MS (EI, 70 eV): *m*/*z* (%) = 270 (90), 240 (73), 222 (6), 211 (10), 199 (80), 192 (100), 181 (20), 174 (16), 165 (20), 162 (23), 152 (5), 139 (25), 121 (13), 114 (11), 105 (13), 91 (8), 77 (27), 57 (3), 45 (11).

***tert*-Butyl((3-((^{13}C)methyl)but-3-en-1-yl)oxy)diphenylsilane (S5)**

To a suspension of CH_3PPh_3I (6.93 g, 17.14 mmol, 2.0 eq) in THF (80 mL) was added n-BuLi (1.6 M in hexane, 10.71 mL, 17.14 mmol, 2.0 eq) dropwise at 0 °C. The reaction mixture was stirred at the same temperature for 1 h. After cooling the mixture to –78 °C, **S4** (2.8 g, 8.55 mmol) was added dropwise, followed by stirring overnight without further cooling. The reaction was quenched by the addition of water and extracted with Et_2O (3 x 100 mL). The combined organic layers were dried with $MgSO_4$ and the solvent was removed under reduced pressure. The product **S5** (1.78 g, 5.46 mmol, 64%) was obtained via silica gel chromatography [cyclohexane/EA (100/1): R_f =0.37].

***tert*-Butyl((3-((^{13}C)methyl)but-3-en-1-yl)oxy)diphenylsilane S5.** ^{1}H NMR (500 MHz, $CDCl_3$): δ = 7.71 – 7.68 (m, 4H, 4 x CH), 7.45 – 7.35 (m, 6H, 6 x CH), 4.80 – 4.65 (m, 2H, CH_2), 3.78 (t, $^3J_{H,H}$ = 6.9 Hz, 2H, CH_2), 2.34 – 2.25 (m, 2H, CH_2), 1.70 (d, $^1J_{C,H}$ = 125.7 Hz, 3H, CH_3), 1.07 (s, 9H, 3 x CH_3). ^{13}C NMR (126 MHz, $CDCl_3$): δ = 143.12 (d, $^1J_{C,C}$ = 41.7 Hz, C_q), 135.74 (4 x CH), 134.15 (2 x C_q), 129.68 (2 x CH), 127.74 (4 x CH), 111.84 (d, $^2J_{C,C}$ = 2.8 Hz, =CH_2), 62.90 (d, $^3J_{C,C}$ = 1.5 Hz, CH_2), 41.02 (d, $^2J_{C,C}$ = 3.6 Hz, CH_2), 27.00 (3 x CH_3), 22.90 ($^{13}CH_3$), 19.36 (C_q). GC (HP-5MS): *I* = 2083. MS (EI, 70 eV): *m*/*z* (%) = 268 (68), 250 (1), 238 (17), 225 (37), 211 (6), 199 (14), 190 (100), 183 (21), 160 (32), 135 (18), 122 (8), 112 (7), 105 (19), 91 (5), 77 (15), 68 (1), 53 (2), 45 (6).

3-((^{13}C)Methyl)but-3-en-1-yl 4-methylbenzenesulfonate (S6)

To a solution of **S5** (1.78 g, 5.46 mmol) in THF (26 mL) was added TBAF (1 M in THF, 6.60 mL, 6.60 mmol, 1.2 eq) dropwise at 0 °C. The reaction mixture was stirred for 2 h at room temperature and then quenched by the addition of water and extracted with Et_2O (3 x 50 mL). The combined organic layers were dried with $MgSO_4$. The solvent was removed carefully under reduced pressure (600 mbar, 40 °C, 40 min). The residue containing the product alcohol was dissolved in CH_2Cl_2 (48 mL) and the solution was cooled to °C, followed by addition of DMAP (2.17 g, 17.79 mmol, 3.3 eq). TsCl (2.61 g, 13.69 mmol, 2.5 eq, suspended in 10 mL CH_2Cl_2)

was added dropwise. After stirring the mixture overnight at room temperature, the reaction was quenched by the addition of sat. NH_4Cl (100 mL), followed by extraction with Et_2O (3 x 100 mL). The organic layers were combined and dried with $MgSO_4$, and the solvent was removed by vacuum evaporation. Via silica gel chromatography [cyclohexane/EA (5/1): R_f =0.44], **S6** (0.60 g, 2.48 mmol, 45%) was obtained as a colorless oil.

3-((^13^C)Methyl)but-3-en-1-yl 4-methylbenzenesulfonate S6. ^{1}H NMR (499 MHz, $CDCl_3$): δ = 7.81 – 7.77 (m, 2H, 2 x CH), 7.36 – 7.32 (m, 2H, 2 x CH), 4.81 - 4.72 (m, 1H, 1/2 x CH_2), 4.72 – 4.61 (m, 1H, 1/2 x CH_2), 4.13 (t, $^3J_{H,H}$ = 6.9 Hz, 2H, CH_2), 2.45 (s, 3H, CH_3), 2.37 – 2.32 (m, 2H, CH_2), 1.66 (d, $^1J_{C,H}$ = 126.1Hz, 3H, CH_3). ^{13}C NMR (126 MHz, $CDCl_3$): δ = 144.85 (C_q), 140.27 (d, $^1J_{C,C}$ = 42.2 Hz, C_q), 133.33 (C_q), 129.94 (2 x CH), 128.05 (2 x CH), 113.23 (d, $^2J_{C,C}$ = 2.7 Hz, C_q), 68.67 (d, $^3J_{C,C}$ = 1.7 Hz, CH_2), 36.90 (d, $^2J_{C,C}$ = 4.0 Hz, CH_2), 22.48 ($^{13}CH_3$), 21.79 (CH_3). GC (HP-5MS): *I* = 1832. MS (EI, 70 eV): *m*/*z* (%) = 173 (4), 155 (26), 139 (1), 107 (1), 91 (54), 77 (2), 69 (100), 56 (6), 41 (5).

Trisammonium (5-^{13}C)isopentenyl diphosphate

$(NBu_4)_3HP_2O_7$ (1.13 g, 1.25 mmol, 3.0 eq) was added to acetonitrile (1 mL), followed by the dropwise addition of **S6** (0.10 g, 0.42 mmol, in 1 mL acetonitrile). The reaction mixture was stirred overnight, and the solvent was removed under reduced pressure. The residue was loaded onto an ion exchange resin column (DOWEX® 50W-X8, 100-200 mesh, $NH4^+$ form), followed by elution with two column volumes of elution buffer (25 mM NH_4HCO_3 in 2% iPrOH/H2O). Lyophilization gave the crude product which was dissolved in aqueous NH_4HCO_3 (0.1 M, 3 mL), followed by extraction with acetonitrile/iPrOH (1/1, 3x 7 mL).[27] The extracts were combined and evaporated under reduced pressure. The residue was freeze-dried to afford (5-^{13}C)IPP (107 mg, 0.36 mmol, 86%) as a colorless powder.

Trisammonium (5-^{13}C)isopentenyl diphosphate (5-^{13}C)IPP. ^{1}H NMR (500 MHz, D_2O): δ = 4.89 – 4.80 (m, 2H, =CH_2), 4.05 (q, $^3J_{H,H}$= 6.7 Hz, CH_2), 2.41 – 2.35 (m, 2H, CH_2), 1.76 (d, $^1J_{C,H}$ = 126.1 Hz, 3H, CH_3). ^{13}C NMR (126 MHz, D_2O): δ = 143.89 (d, $^1J_{C,C}$= 41.4 Hz, C_q), 111.56 (d, $^2J_{C,C}$ = 2.7 Hz, CH_2), 64.26 (d, $^2J_{P,C}$ = 5.3 Hz, CH_2), 37.91 (dd, $^2J_{C,C}$ = 7.6, $^3J_{P,C}$ =3.8 Hz, CH_2), 21.73 ($^{13}CH_3$). ^{31}P NMR (202 MHz, D_2O): δ = –7.78 (d, $^2J_{P,P}$ = 15.9 Hz, 1P), –10.47 (d, $^2J_{P,P}$ = 16.0 Hz, 1P).

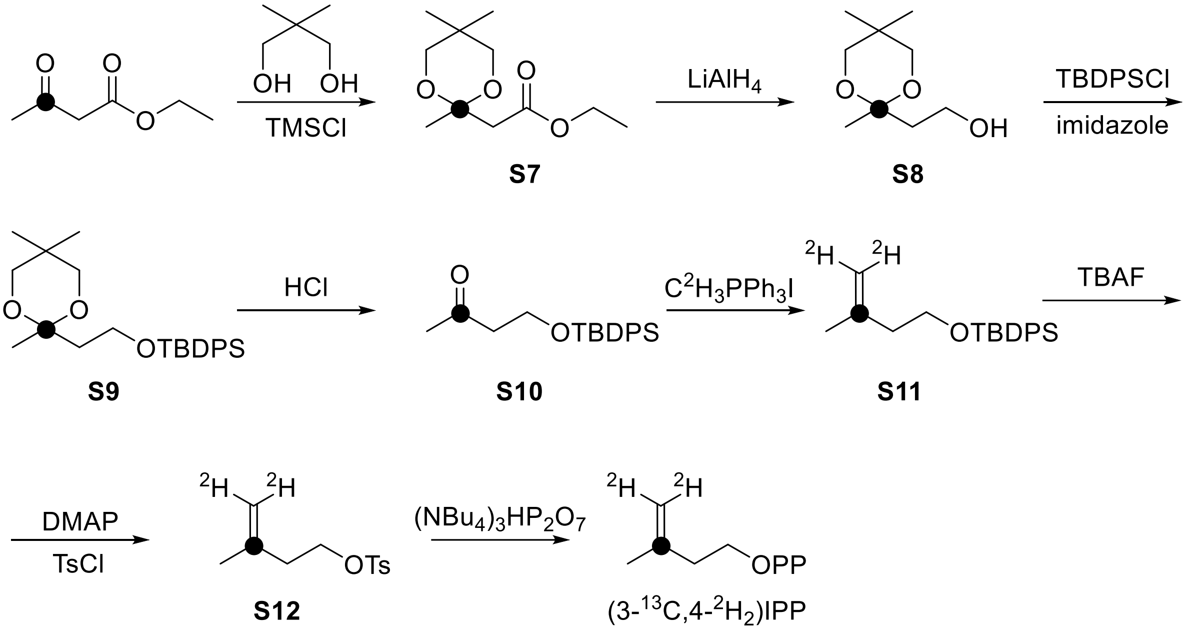

Scheme S7. The synthetic route towards (3-^{13}C,4-$^{2}H_2$)IPP.

Synthesis of (3-^{13}C,4-$^{2}H_2$)IPP

Ethyl 2-((2-^{13}C)-2,5,5-trimethyl-1,3-dioxan-2-yl)acetate (S7)

Following a published procedure,[28] (3-^{13}C)acetoacetate (1.00 g, 7.63 mmol) and neopentyl glycol (1.74 g, 16.72 mmol, 2.2 eq) were dissolved in CH_2Cl_2 (40 mL) at room temperature. TMSCl (3.63 g, 33.44 mmol, 4.4 eq) was added dropwise, followed by stirring the reaction mixture under reflux overnight. The reaction mixture was cooled to room temperature and then quenched by the addition of a mixture of sat. $NaHCO_3$ (60 mL) and ice-water (100 mL). The product was extracted with Et_2O (3 x 80 mL), the combined organic layers were dried with $MgSO_4$, and the solvent was removed under reduced pressure. Via silica gel chromatography [cyclohexane/EA (5/1), R_f=0.36], **S7** (1.44 g, 6.63 mmol, 87%) was obtained as a colourless oil.

Ethyl 2-((2-^{13}C)-2,5,5-trimethyl-1,3-dioxan-2-yl)acetate S7. ^{1}H NMR (300 MHz, C_6D_6): δ = 3.98 (q, $^{3}J_{H,H}$ = 7.1 Hz, 2H, CH_2), 3.43 – 3.21 (m, 4H, 2 x CH_2), 2.78 (d, $^{3}J_{H,H}$ = 5.9 Hz, 2H, CH_2), 1.68 (d, $^{2}J_{C,H}$ = 4.8 Hz, 3H, CH_3), 0.96 (t, $^{3}J_{H,H}$ = 7.1 Hz, 3H, CH_3), 0.76 (s, 3H, CH_3), 0.67 (s, 3H, CH_3). ^{13}C NMR (75 MHz, C_6D_6): δ = 169.13 (C_q), 97.79 ($^{13}C_q$), 70.55 (d, $^{2}J_{C,C}$ = 2.1 Hz, 2 x CH_2), 60.26 (CH_2) , 41.75 (d, $^{1}J_{C,C}$ = 44.1 Hz, CH_2), 29.76 (d, $^{3}J_{C,C}$ = 2.4 Hz, C_q), 23.32 (d, $^{1}J_{C,C}$ = 48.0 Hz, CH_3), 22.60 (CH_3), 22.43 (CH_3), 14.23 (CH_3). GC (HP-5MS): *I* = 1340. MS (EI, 70 eV): *m*/*z* (%) = 202 (32), 130 (100), 116 (16), 104 (12), 86 (28), 69 (52), 56 (32), 44 (81).

2-((2-^{13}C)-2,5,5-Trimethyl-1,3-dioxan-2-yl)ethan-1-ol (S8)

$LiAlH_4$ (0.25 g, 6.63 mmol, 1.0 eq) was suspended in THF (50 mL) and the suspension was cooled to 0 °C. Then **S7** (1.44 g, 6.63 mmol, in 5 mL THF) was added dropwise, and the mixture was stirred overnight without further cooling. After cooling the reaction mixture to 0 °C, water (2.5 mL) was carefully added to quench the reaction, followed by the addition of $MgSO_4$ for drying the solvent. The solids were removed by filtration, and the filtrate was concentrated under reduced pressure to afford **S8** (1.15 g, 6.56 mmol, 99%) without further purification.

2-((2-^{13}C)-2,5,5-Trimethyl-1,3-dioxan-2-yl)ethan-1-ol S8. ^{1}H NMR (300 MHz, C_6D_6): δ = 4.00 – 3.87 (m, 2H, CH_2), 3.34 – 3.02 (m, 4H, 2 x CH_2), 2.81 (t, $^{3}J_{H,H}$ = 5.7 Hz, 1H, OH), 1.94 – 1.78 (m, 2H, CH_2), 1.17 (d, $^{2}J_{C,H}$ = 4.4 Hz, 3H, CH_3), 0.96 (s, 3H, CH_3), 0.40 (s, 3H, CH_3). ^{13}C NMR (75 MHz, C_6D_6): δ = 100.10 ($^{13}C_q$), 70.23 (d, $^{2}J_{C,C}$ = 2.1 Hz, 2 x CH_2), 58.79 (d, $^{2}J_{C,C}$ = 2.0 Hz,

CH_2), 42.48 (d, $^1J_{C,C}$ = 46.9 Hz, CH_2), 29.75 (d, $^3J_{C,C}$ = 2.4 Hz, C_q), 22.81 (CH_3), 22.03 (CH_3), 19.08 (d, $^1J_{C,C}$ = 45.9 Hz, CH_3). GC (HP-5MS): *I* = 1236. MS (EI, 70 eV): *m*/*z* (%) = 160 (42), 130 (56), 90 (24), 74 (39), 69 (45), 56 (46), 44 (100), 41 (47), 31 (23).

tert-Butyldiphenyl(2-((2-^{13}C)-2,5,5-trimethyl-1,3-dioxan-2-yl)ethoxy)silane (S9)
Compound **S8** (1.15 g, 6.56 mmol) and imidazole (0.63 g, 9.24 mmol) were added into DCM (40 mL). After the solids were dissolved, TBDPSCl (2.20 g, 7,92 mmol, 1.2 eq, in 2 mL DCM) was added dropwise, and the reaction mixture was stirred overnight. The reaction was quenched by adding water (80 mL) and extracted with Et_2O (3 x 80 mL). The combined organic layers were washed with brine and dried with $MgSO_4$. The solvent was removed under reduced pressure and the product **S9** (2.25 g, 82.4%) was obtained via silica gel chromatography [cyclohexane/EA (20/1), R_f=0.25] as a colorless oil.
tert-Butyldiphenyl(2-((2-^{13}C)-2,5,5-trimethyl-1,3-dioxan-2-yl)ethoxy)silane S9. ^{1}H NMR (300 MHz, C_6D_6): δ = 7.86 – 7.77 (m, 4H, 4 x CH), 7.27-7.16 (m, 6H, 6 x CH), 4.07 (td, $^3J_{H,H}$ = 7.3 Hz, $^3J_{C,H}$ = 2.5 Hz, 2H, CH_2), 3.21 (m, 4H, 2 x CH_2), 2.24 (td, $^3J_{H,H}$ = 7.4 Hz, $^2J_{C,H}$ = 4.9 Hz, 2H,CH_2), 1.34 (d, $^2J_{C,H}$ = 4.5 Hz, 3H, CH_3), 1.19 (s, 9H, 3 x CH_3), 0.72 (s, 3H, CH_3), 0.65 (s, 3H, CH_3). ^{13}C NMR (75 MHz, C_6D_6): δ = 136.07 (4 x CH), 134.46 (2 x C_q), 129.92 (2 x CH), 128.09 (4 x CH), 98.24 ($^{13}C_q$), 70.20 (d, $^2J_{C,C}$ = 2.1 Hz, 2 x CH_2), 60.57 (CH_2), 29.79 (d, *J* = 2.5 Hz, C_q), 27.18 (3 x CH_3), 22.69 (CH_3), 22.54 (CH_3), 19.47 (C_q). GC (HP-5MS): *I* = 2585. MS (EI, 70 eV): *m*/*z* (%) = 398 (4), 356 (1), 283 (3), 270 (100), 252 (2), 240 (42), 225 (6), 211 (6), 199 (61), 192 (48), 181 (13), 174 (5), 165 (9), 162 (8), 148 (5), 139 (14), 135 (12), 130 (41), 121 (6), 105 (9), 91 (6), 77 (11), 69 (24), 56 (28), 44 (28).

(2-^{13}C)-4-((_tert_-Butyldiphenylsilyl)oxy)butan-2-one (S10)
Compound **S9** (2.25 g, 5.44 mmol) was dissolved in MeOH (34 mL), followed by the dropwise addition of HCl (1 M, 2.6 mL, 2.6 mmol, 0.48 eq). After stirring the reaction mixture at room temperature for 30 min, the reaction was quenched the by addition of $NaHCO_3$ (5%, 56 mL). The product was extracted with Et_2O (3 x 60 mL). The combined organic layers were dried with $MgSO_4$ and concentrated under reduced pressure. Purification by silica gel chromatography [cyclohexane/EA (20/1), R_f=0.20] afforded **S10** (1.37 g, 4.18 mmol, 77%) as a colorless oil.
(2-^{13}C)-4-((_tert_-Butyldiphenylsilyl)oxy)butan-2-one S10. ^{1}H NMR (300 MHz, C_6D_6): δ = 7.79 – 7.73 (m, 4H, 4 x CH), 7.25 – 7.20 (m, 6H, 6 x CH), 3.85 (td, $^3J_{H,H}$ = 6.1 Hz, $^3J_{C,H}$ = 4.4 Hz, 2H, CH_2), 2.19 (q, $^3J_{H,H}$ = 5.9 Hz, $^2J_{C,H}$ = 5.9 Hz, 2H, CH_2), 1.70 (d, $^2J_{C,H}$ = 5.9 Hz, 3H, CH_3), 1.14 (s, 9H, 3 x CH_3). ^{13}C NMR (75 MHz, C_6D_6) : δ = 205.06 ($^{13}C_q$), 136.01 (4 x CH), 133.98 (2 x C_q), 130.07 (2 x C_q), 128.13 (4 x CH), 59.98 (d, $^2J_{C,C}$ = 2.0 Hz, CH_2), 46.03 (d, $^1J_{C,C}$ = 39.7 Hz, CH_2), 30.08 (d, $^1J_{C,C}$ = 40.6 Hz, CH_3), 27.05 (3 x CH_3) , 19.42 (C_q). GC (HP-5MS): *I* = 2204. MS (EI, 70 eV): *m*/*z* (%) = 270 (60), 240 (44), 199 (100), 192 (36), 181 (17), 174 (6), 162 (8), 152 (7), 139 (9), 121 (8), 114 (4), 105 (10), 91 (6), 77 (22), 71 (4), 57 (21), 44 (25), 41 (18).

tert-Butyl(((3-^{13}C,4-$^2H_2$)-3-methylbut-3-en-1-yl)oxy)diphenylsilane (S11)
CD_3PPh_3I (3.07 g, 7.53 mmol, 1.8 eq) was added to THF (40 mL) and the mixture was cooled to 0 °C. Then n-BuLi (1.6 M in hexane, 4.7 mL, 7.53 mmol, 1.8 eq) was added dropwise, followed by stirring the mixture at 0 °C for 1 h. The reaction mixture was cooled to –78 °C, **S10** (1.37 g, 4.18 mmol) was added dropwise, and stirring was continued overnight without further cooling. The reaction was poured onto ice-water (150 mL), and the product was extracted with Et_2O (3 x 70 mL). The combined organic layers were dried with $MgSO_4$ and concentrated under reduced pressure. The product **S11** (1.16 g, 3.54 mmol, 85%) was obtained as a colorless oil via silica gel chromatography [cyclohexane/EA (40/1), R_f=0.40].
tert-Butyl(((3-^{13}C,4-$^2H_2$)-3-methylbut-3-en-1-yl)oxy)diphenylsilane S11. ^{1}H NMR (700 MHz, C_6D_6): δ = 7.80 – 7.77 (m, 4H, 4 x CH), 7.24 – 7.20 (m, 6H, 6 x CH), 3.77 (td, $^3J_{H,H}$ = 6.7 Hz, $^3J_{C,H}$ = 3.6 Hz, 2H, CH_2), 2.24 (td, $^3J_{H,H}$ = 6.7 Hz, $^2J_{C,H}$ = 5.9 Hz, 2H, CH_2), 1.58 (d, $^2J_{C,H}$ = 6.3 Hz, 3H, CH_3), 1.18 (s, 9H, 3 x CH_3). ^{13}C NMR (176 MHz, C_6D_6): δ = 142.77 ($^{13}C_q$), 136.04 (4 x CH), 134.39 (2 x C_q), 129.96 (2 x CH), 128.07 (4 x CH), 63.04 (d, $^2J_{C,C}$ = 1.8 Hz, CH_2), 41.16 (d, $^1J_{C,C}$ = 41.1 Hz, CH_2), 27.13 (3 x CH_3), 22.65 (d, $^1J_{C,C}$ = 42.3 Hz, CH_3), 19.51 (C_q). GC (HP-5MS): *I* = 2078. MS (EI, 70 eV): *m*/*z* (%) = 270 (100), 240 (25), 225 (45), 211 (11),

199 (22), 192 (86), 181 (41), 162 (27), 155 (4), 147 (5), 135 (25), 121 (9), 114 (5), 105 (28), 91 (9), 77 (16), 57 (42), 41 (30).

(3-^{13}C,4-^{2}H$_2$)-3-Methylbut-3-en-1-yl-4-methylbenzenesulfonate (S12)

Compound **S11** (1.16 g, 3.54 mmol) was dissolved in THF (15 mL) and the solution was cooled to 0 °C. TBAF (1 M in THF, 4.25 mL, 4.25 mmol) was added dropwise, and the reaction mixture was stirred at room temperature for 1.5 h. The reaction mixture was poured onto ice-water, followed by extraction with Et_2O (3 x 60 mL). The combined organic layers were then with $MgSO_4$ and concentrated carefully under reduced pressure (600 mbar, 40 °C, 40 min) to afford the crude alcohol which was used for next step directly.

CH_2Cl_2 (40 mL) and DMAP (1.43 g, 11.68 mmol, 3.3 eq) were added to the residue and the solution was cooled to 0 °C. TsCl (1.68 g, 8.85 mmol, 2.5 eq, suspended in 8 mL DCM) was added dropwise and the reaction mixture was stirred overnight without further cooling. The reaction was quenched by pouring onto a mixture of sat. NH_4HCO_3 (60 mL) and ice-water (100 mL). The product was extracted with Et_2O (3 x 60 mL) and the combined extracts were dried with $MgSO_4$. The solvent was removed under reduced pressure. The product **S12** (0.68 g, 2.79 mmol, 79%) was obtained as colorless oil via silica gel chromatography [pentane/Et_2O(5/1), R_f = 0.44].

(3-^{13}C,4-^{2}H$_2$)-3-Methylbut-3-en-1-yl-4-methylbenzenesulfonate S12. ^{1}H NMR (700 MHz, C_6D_6): δ = 7.76 – 7.73 (m, 2H, 2 x CH), 6.69 – 6.65 (m, 2H, 2 x CH), 3.93 (td, $^3J_{H,H}$ = 6.7 Hz, $^3J_{C,H}$ = 3.8 Hz, 2H, CH_2), 1.98 (q, $^3J_{H,H}$ = 6.5 Hz, 2H, CH_2), 1.82 (s, 3H, CH_3), 1.36 (d, $^2J_{C,H}$ = 6.3 Hz, 3H, CH_3). ^{13}C NMR (176 MHz, C_6D_6): δ = 144.15 (C_q), 140.31 ($^{13}C_q$), 134.56 (C_q), 129.78 (2 x CH), 128.18 (2 x C_q), 68.23 (d, $^2J_{C,C}$ = 1.8 Hz, CH_2), 36.83 (d, $^1J_{C,C}$ = 41.1 Hz, CH_2), 22.02 (d, $^1J_{C,C}$ = 41.2 Hz, CH_2), 21.13 (CH_3). GC (HP-5MS): *I* = 1824. MS (EI, 70 eV): *m*/*z* (%) = 173 (5), 155 (43), 134 (3), 107 (5), 91 (94), 71 (100), 65 (40), 58 (13), 41 (13).

Trisammonium (3-^{13}C,4-^{2}H$_2$)isopentenyl diphosphate (3-^{13}C, 4-^{2}H$_2$)IPP

Following the same procedure as described above for the preparation of (5-^{13}C)IPP, **S12** (0.28 g, 1.15 mmol) was converted into (3-^{13}C, 4-^{2}H$_2$)IPP (310 mg, 1.03 mmol, 90%) as a colorless powder.

(3-^{13}C, 4-^{2}H$_2$)IPP. ^{1}H NMR (499 MHz, D_2O): δ = 4.02 – 3.92 (m, 2H, CH_2), 2.31 (q, $^3J_{H,H}$ = 6.5 Hz, 2H, CH_2), 1.69 (d, $^2J_{C,H}$ = 6.3 Hz, 3H, CH_3). ^{13}C NMR (126 MHz, D_2O): δ = 143.72 ($^{13}C_q$), 64.07 (dd, $^2J_{P,C}$ = 5.7 Hz, $^2J_{C,C}$ = 1.9 Hz, CH_2), 37.77 (dd, $^1J_{C,C}$ = 41.8 Hz, $^3J_{P,C}$ = 7.6 Hz, CH_2), 21.57 (d, $^1J_{C,C}$ = 41.3 Hz, CH_3). ^{31}P NMR (202 MHz, D_2O): δ = –7.12 (d, $^2J_{P,P}$ = 21.7 Hz, 1P), –10.54 (d, $^2J_{P,P}$ = 21.8 Hz, 1P).

References

[1] K. Grob, F. Zürcher, *J. Chromatogr.* **1976**, *117*, 285.
[2] J. Rinkel, J. S. Dickschat, *Org. Lett.* **2019**, *21*, 2426.
[3] S.-Y. Kim, P. Zhao, M. Igarashi, R. Sawa, T. Tomita, M. Nishiyama, T. Kuzuyama, *Chem. Biol.* **2009**, *16*, 736.
[4] C. Nakano, T. Tezuka, S. Horinouchi, Y. Ohnishi, *J. Antibiot.* **2012**, *65*, 551.
[5] P. Rabe, J. S. Dickschat, *Angew. Chem. Int. Ed.* **2013**, *52*, 1810.
[6] W. K. W. Chou, I. Fanizza, T. Uchiyama, M. Komatsu, H. Ikeda, D. E. Cane, *J. Am. Chem. Soc.* **2010**, *132*, 8850.
[7] D. E. Cane, J. K. Sohng, C. R. Lamberson, S. M. Rudnicki, Z. Wu, M. D. Lloyd, J. S. Oliver, B. R. Hubbard, *Biochemistry* **1994**, *33*, 5846.
[8] P. Rabe, M. Samborskyy, P. F. Leadlay, J. S. Dickschat, *Org. Biomol. Chem.* **2017**, *15*, 2353.
[9] P. Rabe, J. Rinkel, T. A. Klapschinski, L. Barra, J. S. Dickschat, *Org. Biomol. Chem.* **2016**, *14*, 158.
[10] P. Rabe, J. Rinkel, E. Dolja, T. Schmitz, B. Nubbemeyer, T. H. Luu, J. S. Dickschat, *Angew. Chem. Int. Ed.* **2017**, *56*, 2776.
[11] P. Baer, P. Rabe, K. Fischer, C. A. Citron, T. A. Klapschinski, M. Groll, J. S. Dickschat, *Angew. Chem. Int. Ed.* **2014**, *53*, 7652.
[12] C. Nakano, S. Horinouchi, Y. Ohnishi, *J. Biol. Chem.* **2011**, *286*, 27980.
[13] J. Rinkel, J. S. Dickschat, *Org. Lett*. **2019**, *21*, 9442.
[14] X. Lin, R. Hopson, D. E. Cane, *J. Am. Chem. Soc*. **2006**, *128*, 6022.
[15] B. Neumann, A. Pospiech, H. U. Schairer, *Trends Genet.* **1992**, *8*, 332.
[16] J. S. Dickschat, K. A. K. Pahirulzaman, P. Rabe, T. A. Klapschinski, *ChemBioChem* **2014**, *15*, 810.
[17] R. D. Giets, R. H. Schiestl, *Nat. Protoc.* **2007**, *2*, 31.
[18] M. M. Bradford, *Anal. Biochem.* **1976**, *72*, 248.
[19] G. R. Fulmer, A. J. M. Miller, N. H. Sherden, H. E. Gottlieb, A. Nudelman, B. M. Stoltz, J. E. Bercaw, K. I. Goldberg, *Organometallics* **2010**, *29*, 2176.
[20] J. Rinkel, L. Lauterbach, J. S. Dickschat, *Angew. Chem. Int. Ed.* **2019**, *58*, 452.
[21] P. Rabe, L. Barra, J. Rinkel, R. Riclea, C. A. Citron, T. A. Klapschinski, A. Janusko, J. S. Dickschat, *Angew. Chem. Int. Ed.* **2015**, *54*, 13448.
[22] G. Bian, J. Rinkel, Z. Wang, L. Lauterbach, A. Hou, Y. Yuan, Z. Deng, T. Liu, J. S. Dickschat, *Angew. Chem. Int. Ed.* **2018**, *57*, 15887.
[23] T. Mitsuhashi, J. Rinkel, M. Okada, I. Abe, J. S. Dickschat, *Chem. Eur. J*. **2017**, *23*, 10053.
[24] L. Lauterbach, J. Rinkel, J. S. Dickschat, *Angew. Chem. Int. Ed.* **2018**, *57*, 8280.
[25] J. Rinkel, L. Lauterbach, P. Rabe, J. S. Dickschat, *Angew. Chem. Int. Ed.* **2018**, *57*, 3238.
[26] P. Rabe, J. Rinkel, B. Nubbemeyer, T. G. Köllner, F. Chen, J. S. Dickschat, *Angew. Chem. Int. Ed.* **2016**, *55*, 15420.
[27] V. J. Davisson, A. B. Woodside, T. R. Neal, K. E. Stremler, M. Muehlbacher, C. D. Poulter, *J. Org. Chem.* **1986**, *51*, 4768.
[28] L. Barra, B. Schulz, J. S. Dickschat, *ChemBioChem* **2014**, *15*, 2379.
